# Supplementary material for: Electrochemical oxidative difunctionalization of diazo compounds with two different nucleophiles
Source: Nat Commun. 2023 Mar 17;14:1476. doi: 10.1038/s41467-023-37032-8 (PMC10020561; doi:10.1038/s41467-023-37032-8)
Supplement: Supplementary file 1 — Supplementary Information [file 41467_2023_37032_MOESM1_ESM.pdf]

Supplementary Information

Electrochemical Oxidative Difunctionalization of Diazo Compounds with Two Different Nucleophiles

Dongfeng Yang[1,*], Zhipeng Guan[1,*], Yanan Peng[1], Shuxiang Zhu[1], Pengjie Wang[1], Zhiliang Huang[1], Hesham Alhumade[2,3], Dong Gu[1,✉], Hong Yi[1,4,✉], Aiwen Lei[1,5,✉]

[1] The Institute for Advanced Studies (IAS) and College of Chemistry and Molecular Sciences, Wuhan University, Wuhan, 430072, Hubei (P. R. China)

[2] K. A. CARE Energy Research and Innovation Center, King Abdulaziz University, Jeddah 21589, Saudi Arabia

[3] Department of Chemical and Materials Engineering, Faculty of Engineering, Center of Research Excellence in Renewable Energy and Power Systems, King Abdulaziz University, Jeddah 21589, Saudi Arabia

[4] Shenzhen Research Institute, Wuhan University, Shenzhen, 518057, Guangdong (P. R. China).

[5] Chemical and Materials Engineering Department, Faculty of Engineering, King Abdulaziz University, Jeddah 21589, Saudi Arabia

[*]These authors contributed equally: Dongfeng Yang, Zhipeng Guan.

[✉]Corresponding author. Email: DGu@whu.edu.cn; hong.yi@whu.edu.cn; aiwenlei@whu.edu.cn

Table of Contents

1 Supplementary Methods **3**
1.1 General information **3**
1.2 Experimental set-up **4**
2 Supplementary Discussion **5**
2.1 Optimization of the reaction conditions **5**
2.2 General procedure for electrochemical experiments **5**
2.3 Gram-scale experiments **5**
2.4 Intramolecular cyclization reaction. **6**
2.5 Derivatization of product **7**
3 Supplementary Notes **8**
3.1 Control experiments **8**
3.2 General procedure for the cyclic voltammetry (CV) experiments **9**
3.3 General procedure for the electron paramagnetic resonance (EPR) experiment **10**
3.4 Detail descriptions for products **11**
3.5 NMR spectra **25**

1 Supplementary Methods

1.1 General information

All glassware was oven dried at 110 °C for hours and cooled down under vacuum. All reactions were performed using standard three-necked bottle under argon atmosphere. 4-Fluorothiophenol (98%), Ethyl diazoacetate (98%) were purchased from Energy Chemical Inc. Tetraethylammonium tetrafluoroborate was purchased from Bidepharm. Unless otherwise noted, all reagents were purchased from commercial suppliers and used without further purification. The instrument for electrolysis was dual display potentiostat (DJS-292B) (made in China). Cyclic voltammograms were obtained on a CHI 605E potentiostat. The anodic electrode was carbon rod and cathodic electrode was platinum sheet (1.5 cm × 1.5 cm ×1 mm). These electrodes were commercially available from GaossUnion and Huanqiujinxin, China. Thin layer chromatography (TLC) employed glass 0.25 mm silica gel plates. Flash chromatography columns were packed with 200-300 mesh silica gel in petroleum (bp. 60-90 °C). GC-MS spectra were recorded on a Varian GC-MS 3900-2100T. The known compounds were characterized by ^{1}H NMR, ^{13}C NMR, ^{31}P NMR and ^{19}F NMR. ^{1}H, ^{19}F, ^{31}P and ^{13}C NMR data were recorded with ADVANCE III 400 MHz with tetramethylsilane as an internal standard. High resolution mass spectra (HRMS) were measured with a Waters Micromass GCT instrument. All chemical shifts (δ) were reported in ppm and coupling constants (*J*) in Hz. All chemical shifts were reported relative to tetramethylsilane (0 ppm for ^{1}H) and $CDCl_3$ (77.16 ppm for ^{13}C) in $CDCl_3$, respectively.

1.2 Experimental set-up

Carbon rod electrode (ϕ 6mm), platinum plate electrode (1.5 cm × 1.5 cm × 1 mm), rubber plug, undivided three-neck bottle were used in the experimental device.

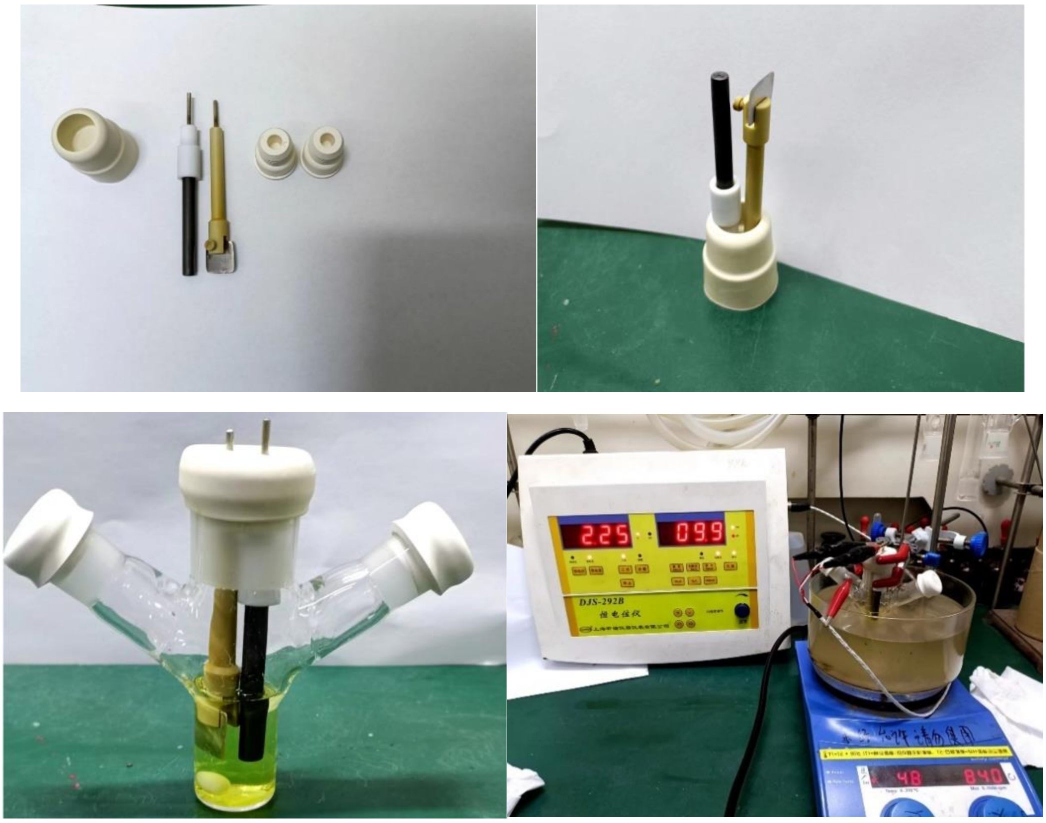

Supplementary Figure 1. Some photos of electrolysis device.

2 Supplementary Discussion

2.1 Optimization of the reaction conditions

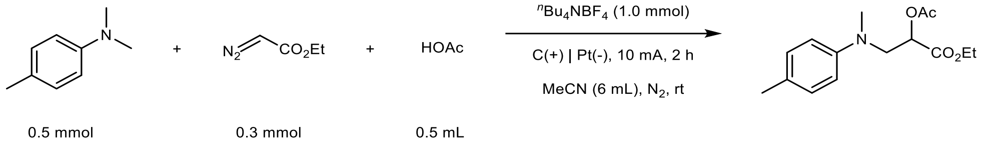

Entry	Variation from the standard conditions	Yield (%)[a]
1	none	69
2	CH_3CN/DCM (3/3, 6 mL)	48
3	DCM/HOAc (4/2, 6 mL)	28
4	Acetone/THF (4/2, 6 mL)	38
5	0.3 mmol *N*,*N*,4-trimethylaniline	51
6	HOAc (0.25 mL)	39
7	HOAc (1.0 mL)	50
8	20 mA, 1.3 h	49
9	5 mA, 3 h	45

Supplementary Table 1. Standard conditions: *N*, *N*, 4-trimethylaniline (0.5 mmol), ethyl diazoacetate (0.3 mmol), HOAc (0.5 mL), ^{n}Bu$_4$NBF$_4$ (1.0 mmol), CH_3CN (6.0 mL) in an undivided cell with carbon rod as anode, platinum as cathode (1.5 × 1.5 cm^2), constant current = 10 mA, rt, N_2, 2 h. [a]Isolated yield.

2.2 General procedure for electrochemical experiments

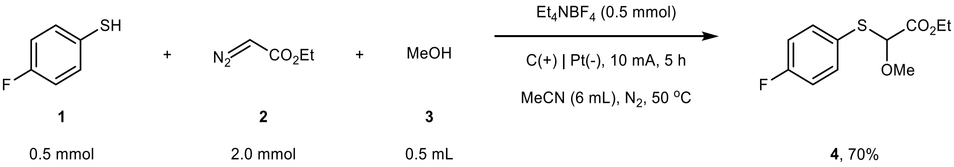

Supplementary Figure 2. General procedure for electrochemical experiments.

In an oven-dried undivided three-necked bottle (10 mL) equipped with a stir bar, Et_4NBF_4 (0.5 mmol) was added. The bottle was equipped with carbon rod as the anode and platinum plate (1.5 cm × 1.5 cm × 1 mm) as the cathode. Under nitrogen atmosphere, 4-fluorothiophenol (**1**, 0.5 mmol), ethyl diazoacetate (**2**, 2.0 mmol), MeOH (**3**, 0.5 mL) and MeCN (6.0 mL) were injected respectively into the tube via syringes. The reaction mixture was stirred and electrolyzed at a constant current of 10 mA for 5 h at 50 °C. The reaction mixture was washed with water and extracted with EA (10 mL × 3). The organic layers were combined, dried over Na_2SO_4 and concentrated in vacuum. The pure product **4** was purified by silica gel flash column chromatography (PE/EA) in 70% yield.

2.3 Gram-scale experiments

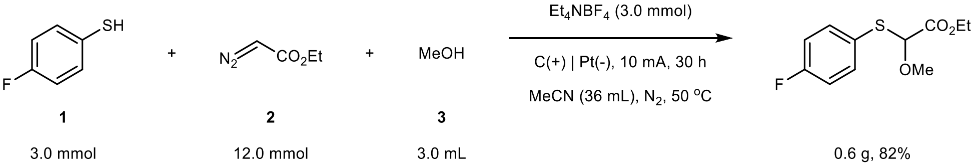

Supplementary Figure 3. Gram-scale experiments.

In an oven-dried undivided three-necked bottle (50 mL) equipped with a stir bar, Et_4NBF_4 (0.5 mmol) was added. The bottle was equipped with carbon rod as the anode and platinum plate (1.5 cm × 1.5 cm × 1 mm) as the cathode. Under nitrogen atmosphere, 4-fluorothiophenol (**1**, 3.0 mmol)，ethyl diazoacetate (**2**, 12.0 mmol), MeOH (**3**, 3.0 mL) and MeCN (36.0 mL) were injected respectively into the tube via syringes. The reaction mixture was stirred and electrolyzed at a constant current of 10 mA for 30 h at 50 °C. The reaction mixture was washed with water and extracted with EA (10 mL × 3). The organic layers were combined, dried over Na_2SO_4 and concentrated in vacuum. The pure product **4** was purified by silica gel flash column chromatography (PE/EA) in 82% yield.

2.4 Intramolecular cyclization reaction.

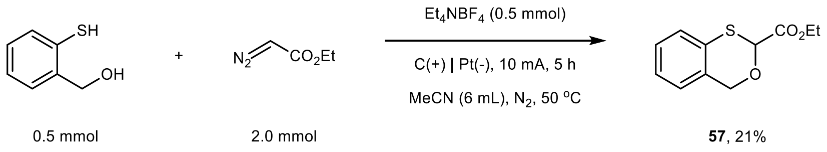

Supplementary Figure 4. Intramolecular cyclization of (2-mercaptophenyl)methanol.

In an oven-dried undivided three-necked bottle (10 mL) equipped with a stir bar, Et_4NBF_4 (0.5 mmol) was added. The bottle was equipped with carbon rod as the anode and platinum plate (1.5 cm × 1.5 cm × 1 mm) as the cathode. Under nitrogen atmosphere, (2-mercaptophenyl)methanol (0.5 mmol)， ethyl diazoacetate (2.0 mmol) and MeCN (6.0 mL) were injected respectively into the tube via syringes. The reaction mixture was stirred and electrolyzed at a constant current of 10 mA for 5 h at 50 °C. The reaction mixture was washed with water and extracted with EA (10 mL × 3). The organic layers were combined, dried over Na_2SO_4 and concentrated in vacuum. The pure product **57** was purified by silica gel flash column chromatography (PE/EA) in 21% yield.

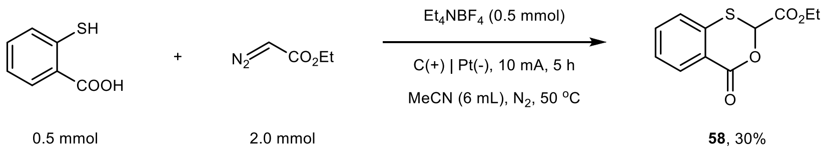

Supplementary Figure 5. Intramolecular cyclization of 2-mercaptobenzoic acid.

In an oven-dried undivided three-necked bottle (10 mL) equipped with a stir bar, Et_4NBF_4 (0.5 mmol) was added. The bottle was equipped with carbon rod as the anode and platinum plate (1.5 cm × 1.5 cm × 1 mm) as the cathode. Under nitrogen atmosphere, 2-mercaptobenzoic acid (0.5 mmol)， ethyl diazoacetate (2.0 mmol) and MeCN (6.0 mL) were injected respectively into the tube via syringes. The reaction mixture was stirred and electrolyzed at a constant current of 10 mA for 5 h at 50 °C. The reaction mixture was washed with water and extracted with EA (10 mL × 3). The organic layers were combined, dried over Na_2SO_4 and concentrated in vacuum. The pure product **58** was purified by silica gel flash column chromatography (PE/EA) in 30% yield.

2.5 Derivatization of product.

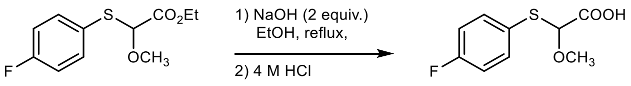

4, 0.2 mmol　　59, 75%

Supplementary Figure 6. Derivatization of product to acid.

A stirring bar was added to the dry Schlenk tube, added **4** (0.2 mmol), NaOH (0.4 mmol), EtOH (4 mL) and then the solution was stirred for until the product disappeared at 80 °C. The aqueous mixture was adjusted to pH =1 with concentrated HCl (4 M × 2 mL) and extracted with ethyl acetate (10 mL × 3). After filtration over Na_2SO_4 and the pure product was purified by flash column chromatography (PE/EA/HOAc) on silica gel resulted in **59** (Yield: 75%)

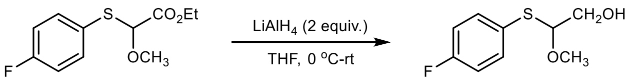

4, 0.2 mmol　　60, 80%

Supplementary Figure 7. Derivatization of product to alcohol.

A stirring bar was added to the dry Schlenk tube for anhydrous and oxygen-free operation. Under nitrogen atmosphere, added **4** (0.2 mmol), dry THF (2 mL). $LiAlH_4$ (0.4 mmol) was added slowly to the THF solution at 0 ºC and then the solution was stirred for two hours at room temperature. After that, a solution of NaOH (10% in water) was added carefully until a white solid precipitated. After filtration over Na_2SO_4 and the pure product was purified by flash column chromatography (PE/EA) on silica gel resulted in **60** (Yield: 80%).

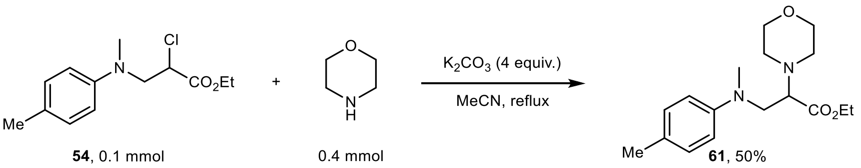

54, 0.1 mmol　　0.4 mmol　　61, 50%

Supplementary Figure 8. Synthesis of morpholine derivative by product derivatization.

A stirring bar was added to the dry Schlenk tube, added **54** (0.1 mmol), K_2CO_3 (0.4 mmol), morpholine (0.4 mmol), MeCN (4 mL) and then the solution was stirred for until the product disappeared at 90 ºC. The reaction mixture was washed with water and extracted with EA (10 mL × 3). The organic layers were combined, dried over Na_2SO_4 and concentrated in vacuum. The pure product **61** was purified by silica gel flash column chromatography (PE/EA) in 50% yield.

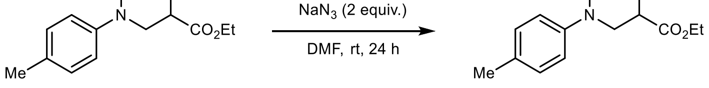

54, 0.1 mmol　　62, 61%

Supplementary Figure 9. Synthesis of azide derivative by product derivatization.

A stirring bar was added to the dry Schlenk tube for anhydrous, NaN_3 (0.2 mmol) was added. Under nitrogen atmosphere, **54** (0.1 mmol), DMF (2.0 mL) were injected respectively into the tube via syringes. Then the solution was stirred for 24 hours at room temperature. The reaction mixture was

washed with water and extracted with EA (10 mL × 3). The organic layers were combined, dried over Na_2SO_4 and concentrated in vacuum. The pure product **62** was purified by silica gel flash column chromatography (PE/EA) in 61% yield.

3 Supplementary Notes

3.1 Control experiments

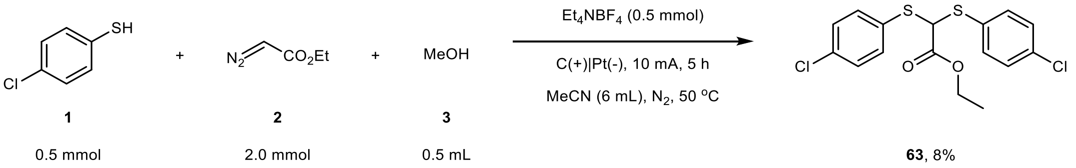

Supplementary Figure 10. Determination of ethyl 2,2-bis ((4-chlorophenyl) thio) acetate.

In an oven-dried undivided three-necked bottle (10 mL) equipped with a stir bar, Et_4NBF_4 (0.5 mmol) was added. The bottle was equipped with carbon rod as the anode and platinum plate (1.5 cm × 1.5 cm × 1 mm) as the cathode. Under nitrogen atmosphere, 4-Chlorothiophenol (**1**, 0.5 mmol), ethyl diazoacetate (**2**, 2.0 mmol), MeOH (**3**, 0.5 mL) and MeCN (6.0 mL) were injected respectively into the tube via syringes. The reaction mixture was stirred and electrolyzed at a constant current of 10 mA for 5 h at 50 °C. The reaction mixture was washed with water and extracted with EA (10 mL × 3). The organic layers were combined, dried over Na_2SO_4 and concentrated in vacuum. The pure product **63** was purified by silica gel flash column chromatography (PE/EA) in 8% yield.

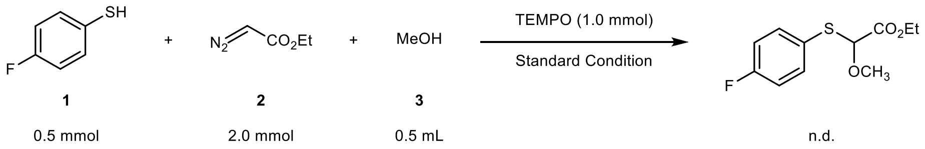

Supplementary Figure 11. TEMPO capture radical experiment.

In an oven-dried undivided three-necked bottle (10 mL) equipped with a stir bar, Et_4NBF_4 (0.5 mmol), TEMPO (1.0 mmol) were added. The bottle was equipped with carbon rod as the anode and platinum plate (1.5 cm × 1.5 cm × 1 mm) as the cathode. Under nitrogen atmosphere, 4-fluorothiophenol (**1**, 0.5 mmol)，ethyl diazoacetate (**2**, 2.0 mmol), MeOH (**3**, 0.5 mL) and MeCN (6.0 mL) were injected respectively into the tube via syringes. The reaction mixture was stirred and electrolyzed at a constant current of 10 mA for 5 h at 50 °C. When the reaction finished, product was undetected by GC-MS.

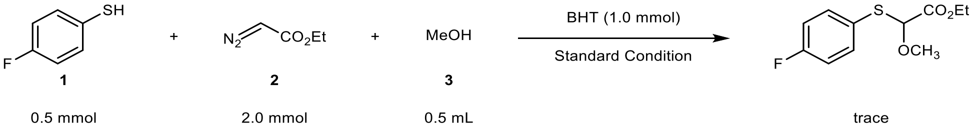

Supplementary Figure 12. BHT capture radical experiment.

In an oven-dried undivided three-necked bottle (10 mL) equipped with a stir bar, Et_4NBF_4 (0.5 mmol), BHT (1.0 mmol) were added. The bottle was equipped with carbon rod as the anode, platinum plate (1.5 cm × 1.5 cm × 1 mm) as the cathode. Under nitrogen atmosphere, 4-

fluorothiophenol (**1**, 0.5 mmol), ethyl diazoacetate (**2**, 2.0 mmol), MeOH (**3**, 0.5 mL) and MeCN (6.0 mL) were injected respectively into the tube via syringes. The reaction mixture was stirred and electrolyzed at a constant current of 10 mA for 5 h at 50 °C. When the reaction finished, only trace product was detected by GC-MS.

3.2 General procedure for the cyclic voltammetry (CV) experiments

Cyclic voltammetry was performed in a three-electrode cell connected to a schlenk line at room temperature. The working electrode was a steady glassy carbon disk electrode, the counter electrode a platinum wire. The reference was Ag/AgCl electrode submerged in saturated aqueous KCl solution, and separated from reaction by a salt bridge. The scan rate was 0.1 V/s.

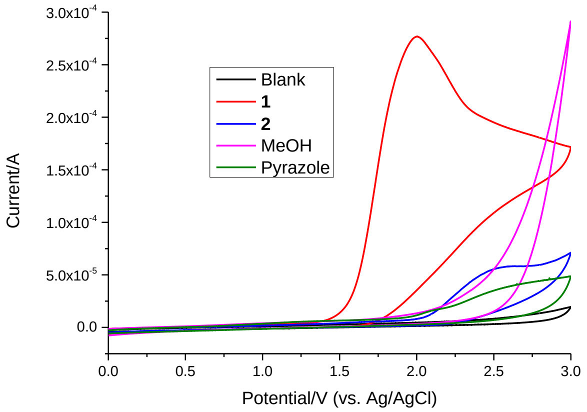

Supplementary Figure 13. The cyclic voltammogram (CV) experiments. Et_4NBF_4 (0.2 mmol), **1** (0.1 mmol), **2** (0.1 mmol), MeOH (0.5 mL), Pyrazole (0.1 mmol), in MeCN (12 mL).

3.3 General procedure for the electron paramagnetic resonance (EPR) experiment

In an oven-dried undivided three-necked bottle (25 mL) equipped with a stir bar, thiol (0.3 mmol), and $^{n}Bu_4NBF_4$ (0.5 mmol) were combined and added. The bottle was equipped with graphite rod (ϕ 6 mm, about 18 mm immersion depth in solution) as the anode and platinum plate (1.5 cm × 1.5 cm × 1 mm) as the cathode and was then charged with nitrogen. Under the protection of N_2, DMPO (30 μL) and MeCN (6.0 mL) were injected respectively into the tubes via syringes. After 5 minutes, the solution sample was taken out into a small tube and analyzed by EPR. EPR spectra was recorded at room temperature on EPR spectrometer operated at 9.823307 GHz. Typical spectrometer parameters are shown as follows, scan range: 100 G; center field set: 3505.202 G; time constant: 163.84 ms; scan time: 30.72 s; modulation amplitude: 1.0 G; modulation frequency: 100 kHz; receiver gain: 1.00×10^4; microwave power: 21.49 mW. The difference between fitting results and experiment results may be caused by linear distortion. The difference between fitting results and experiment results may be caused by linear distortion.

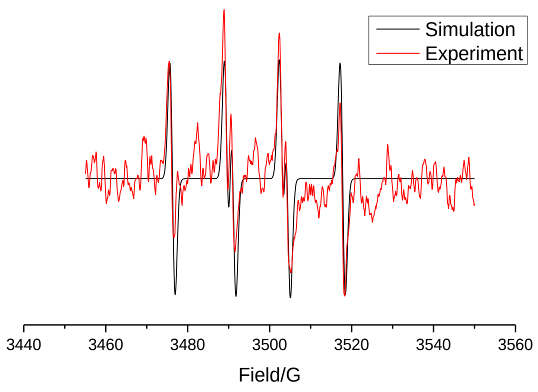

Supplementary Figure 14. EPR experiment.

3.4 Detail descriptions for products

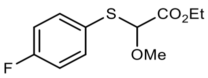

Ethyl 2-((4-fluorophenyl)thio)-2-methoxyacetate (**4**). Colorless liquid was obtained in 70% isolated yield. ^1H NMR (400 MHz, $CDCl_3$) δ 7.51–7.44 (m, 2H), 7.05–6.99 (m, 2H), 5.03 (s, 1H), 4.12 (q, J = 7.2 Hz, 2H), 3.59 (s, 3H), 1.19 (t, J = 7.2 Hz, 3H). ^{13}C NMR (101 MHz, $CDCl_3$) δ 167.15, 163.40 (d, $J_{C\text{-}F}$ = 249.47 Hz), 136.91, 136.82, 125.84 (d, $J_{C\text{-}F}$ = 3.03 Hz), 116.25, 116.03, 86.43, 61.71, 56.13, 14.11. ^{19}F NMR (377 MHz, $CDCl_3$) δ -111.98. HRMS (ESI) calculated for $C_{11}H_{14}O_3SF$ $[M+H]^+$ 245.0642 found 245.0634.

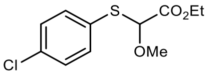

Ethyl 2-((4-chlorophenyl)thio)-2-methoxyacetate (**5**). Colorless liquid was obtained in 65% isolated yield. ^1H NMR (400 MHz, $CDCl_3$) δ 7.42 (d, J = 9.0 Hz, 2H), 7.28 (d, J = 8.0 Hz, 2H), 5.06 (s, 1H), 4.13 (q, J = 7.2 Hz, 2H), 3.58 (s, 3H), 1.19 (t, J = 7.2 Hz, 3H). ^{13}C NMR (101 MHz, $CDCl_3$) δ 167.10, 135.58, 135.19, 129.48, 129.18, 86.31, 61.81, 56.16, 14.12. HRMS (ESI) calculated for $C_{11}H_{14}O_3SCl$ $[M+H]^+$ 261.0347 found 261.0340.

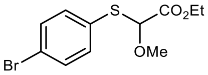

Ethyl 2-((4-bromophenyl)thio)-2-methoxyacetate (**6**). Colorless liquid was obtained in 75% isolated yield. ^1H NMR (400 MHz, $CDCl_3$) δ 7.46–7.42 (m, 2H), 7.37–7.34 (m, 2H), 5.07 (s, 1H), 4.13 (q, J = 7.2 Hz, 2H), 3.58 (s, 3H), 1.19 (t, J = 7.2 Hz, 3H). ^{13}C NMR (101 MHz, $CDCl_3$) δ 167.06, 135.71, 132.10, 130.13, 123.32, 86.18, 61.81, 56.14, 14.11. HRMS (ESI) calculated for $C_{11}H_{14}O_3SBr$ $[M+H]^+$ 304.9842 found 304.9842.

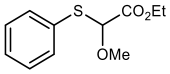

Ethyl 2-methoxy-2-(phenylthio)acetate (**7**). Colorless liquid was obtained in 55% isolated yield. ^1H NMR (400 MHz, $CDCl_3$) δ 7.51–7.48 (m, 2H), 7.34–7.30 (m, 3H), 5.10 (s, 1H), 4.12 (q, J = 7.2 Hz, 2H), 3.58 (s, 3H), 1.17 (t, J = 7.2 Hz, 3H). ^{13}C NMR (101 MHz, $CDCl_3$) δ 167.38, 134.28, 131.17, 128.99, 128.73, 86.61, 61.69, 56.07, 14.07. HRMS (ESI) calculated for $C_{11}H_{15}O_3S$ $[M+H]^+$ 227.0736 found 227.0729.

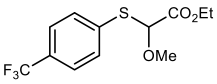

Ethyl 2-methoxy-2-((4-(trifluoromethyl)phenyl)thio)acetate (**8**). Colorless liquid was obtained in 72% isolated yield. ^1H NMR (400 MHz, $CDCl_3$) δ 7.63–7.55 (m, 4H), 5.18 (s, 1H), 4.15 (q, J = 7.2 Hz, 2H), 3.60 (s, 3H), 1.18 (t, J = 7.2 Hz, 3H). ^{13}C NMR (101 MHz, $CDCl_3$) δ 167.04, 136.59, 133.26, 130.34 (q, $J_{C\text{-}F}$ = 32.32 Hz), 125.77 (q, $J_{C\text{-}F}$ = 4.04 Hz), 124.00 (q, $J_{C\text{-}F}$ = 272.7 Hz), 85.94, 62.02, 56.18, 14.06. ^{19}F NMR (377 MHz, $CDCl_3$) δ -62.77. HRMS (ESI) calculated for $C_{12}H_{14}O_3F_3S$ $[M+H]^+$ 295.0610 found 295.0604.

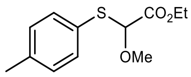

Ethyl 2-methoxy-2-(p-tolylthio)acetate (**9**). Colorless liquid was obtained in 63% isolated yield. ^{1}H NMR (400 MHz, $CDCl_3$) δ 7.40–7.36 (m, 2H), 7.12 (d, J = 8.0 Hz, 2H), 5.03 (s, 1H), 4.13 (q, J = 7.2 Hz, 2H), 3.58 (s, 3H), 2.33 (s, 3H), 1.19 (t, J = 7.2 Hz, 3H). ^{13}C NMR (101 MHz, $CDCl_3$) δ 167.44, 139.05, 134.68, 129.79, 127.25, 86.69, 61.61, 56.06, 21.26, 14.10. HRMS (ESI) calculated for $C_{12}H_{17}O_3S$ $[M+H]^+$ 241.0893 found 241.0891.

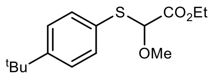

(4-(tert-butyl)phenyl)(ethoxy(methoxy)methyl)sulfane (**10**). Colorless liquid was obtained in 56% isolated yield. ^{1}H NMR (400 MHz, $CDCl_3$) δ 7.44–7.41 (m, 2H), 7.35–7.32 (m, 2H), 5.06 (s, 1H), 4.12 (q, J = 7.2 Hz, 2H), 3.59 (s, 3H), 1.30 (s, 9H), 1.16 (t, J = 7.2 Hz, 3H). ^{13}C NMR (101 MHz, $CDCl_3$) δ 167.57, 152.11, 134.43, 127.41, 126.11, 86.78, 61.66, 56.09, 34.71, 31.28, 14.08. HRMS (ESI) calculated for $C_{15}H_{23}O_3S$ $[M+H]^+$ 283.1362 found 283.1360.

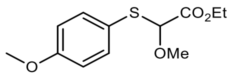

Ethyl 2-methoxy-2-((4-methoxyphenyl)thio)acetate (**11**). Colorless liquid was obtained in 50% isolated yield. ^{1}H NMR (400 MHz, $CDCl_3$) δ 7.43–7.40 (m, 2H), 6.87–6.83 (m, 2H), 4.98 (s, 1H), 4.13 (q, J = 8.0 Hz, 2H), 3.79 (s, 3H), 3.58 (s, 3H), 1.20 (t, J = 8.0 Hz, 3H). ^{13}C NMR (101 MHz, $CDCl_3$) δ 167.49, 160.56, 136.85, 121.00, 114.60, 86.79, 61.62, 56.13, 55.38, 14.17. HRMS (ESI) calculated for $C_{12}H_{17}O_4S$ $[M]^+$ 256.0756 found 256.0769.

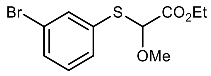

Ethyl 2-((3-bromophenyl)thio)-2-methoxyacetate (**12**). Colorless liquid was obtained in 60% isolated yield. ^{1}H NMR (400 MHz, $CDCl_3$) δ 7.65 (t, J = 4.0 Hz, 1H), 7.46–7.42 (m, 2H), 7.19 (t, J = 8.0 Hz, 1H), 5.11 (s, 1H), 4.15 (q, J = 8.0 Hz, 2H), 3.58 (s, 3H), 1.20 (t, J = 8.0 Hz, 3H). ^{13}C NMR (101 MHz, $CDCl_3$) δ 167.06, 136.43, 133.38, 132.59, 131.73, 130.27, 122.49, 86.37, 61.89, 56.16, 14.10. HRMS (ESI) calculated for $C_{11}H_{14}O_3SBr$ $[M+H]^+$ 304.9842 found 304.9860.

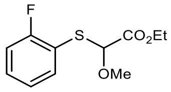

Ethyl 2-((2-fluorophenyl)thio)-2-methoxyacetate (**13**). Colorless liquid was obtained in 52% isolated yield. ^{1}H NMR (400 MHz, $CDCl_3$) δ 7.56–7.51 (m, 1H), 7.40–7.35 (m, 1H), 7.16–7.10 (m, 2H), 5.09 (s, 1H), 4.19 (q, J = 7.2 Hz, 2H), 3.63 (s, 3H), 1.24 (t, J = 8.0 Hz, 3H). ^{13}C NMR (101 MHz, $CDCl_3$) δ 167.27, 163.08 (d, J_{C-F} = 248.46 Hz), 137.60, 131.50 (d, J_{C-F} = 8.08 Hz), 124.61 (d, J_{C-F} = 4.04 Hz), 117.48 (d, J_{C-F} = 16.16 Hz), 116.04 (d, J_{C-F} = 23.23 Hz), 85.81, 61.87, 56.31, 14.05. ^{19}F NMR (377 MHz, $CDCl_3$) δ -106.65. HRMS (ESI) calculated for $C_{11}H_{14}O_3SF$ $[M+H]^+$ 245.0642 found 245.0636.

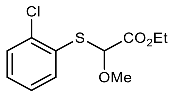

Ethyl 2-((2-chlorophenyl)thio)-2-methoxyacetate (**14**). Colorless liquid was obtained in 56% isolated yield. ^{1}H NMR (400 MHz, $CDCl_3$) δ 7.63–7.61 (m, 1H), 7.43–7.41 (m, 1H), 7.26–7.21 (m, 2H), 5.19 (s, 1H), 4.21–4.14 (m, 2H), 3.60 (s, 3H), 1.22 (t, J = 7.2 Hz, 3H). ^{13}C NMR (101 MHz, $CDCl_3$) δ 167.16, 137.27, 135.55, 130.84, 129.97, 129.67, 127.26, 85.71, 61.96, 56.26, 14.01. HRMS (ESI) calculated for $C_{11}H_{14}O_3SCl$ $[M+H]^+$ 261.0347 found 261.0340.

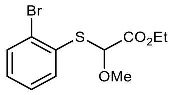

Ethyl 2-((2-bromophenyl)thio)-2-methoxyacetate (**15**). Colorless liquid was obtained in 55% isolated yield. ^{1}H NMR (400 MHz, $CDCl_3$) δ 7.65–7.58 (m, 2H), 7.30–7.26 (m, 1H), 7.17–7.13 (m, 1H), 5.21 (s, 1H), 4.23–4.14 (m, 2H), 3.60 (s, 3H), 1.22 (t, J = 8.0 Hz, 3H). ^{13}C NMR (101 MHz, $CDCl_3$) δ 167.12, 134.91, 133.26, 133.24, 129.57, 127.92, 127.50, 85.87, 62.02, 56.29, 14.04. HRMS (ESI) calculated for $C_{11}H_{14}O_3SBr$ $[M+H]^+$ 304.9842 found 304.9862.

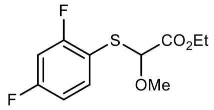

Ethyl 2-((2,4-difluorophenyl)thio)-2-methoxyacetate (**16**). Colorless liquid was obtained in 65% isolated yield. ^{1}H NMR (400 MHz, $CDCl_3$) δ 7.52–7.45 (m, 1H), 6.89–6.85 (m, 2H), 4.99 (s, 1H), 4.17 (q, J = 8.0 Hz, 2H), 3.60 (s, 3H), 1.24 (t, J = 8.0 Hz, 3H). ^{13}C NMR (101 MHz, $CDCl_3$) δ 167.07, 164.15(dd, $J_{C\text{-}F}$ = 11.11, 12.12 Hz), 163.69 (dd, $J_{C\text{-}F}$ = 12.12, 13.13 Hz), 139.13 (dd, $J_{C\text{-}F}$ = 2.02, 1.01 Hz), 112.59 (dd, $J_{C\text{-}F}$ = 4.04, 4.04 Hz), 112.10 (dd, $J_{C\text{-}F}$ = 4.04, 4.04 Hz), 104.66 (t, $J_{C\text{-}F}$ = 27.27 Hz), 85.65, 61.88, 56.34, 56.33, 14.05. ^{19}F NMR (377 MHz, $CDCl_3$) δ -101.33, -101.36, -106.42, -106.45. HRMS (ESI) calculated for $C_{11}H_{13}O_3F_2S$ $[M+H]^+$ 263.0548 found 263.0540.

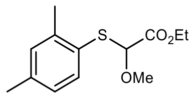

Ethyl 2-((2,4-dimethylphenyl)thio)-2-methoxyacetate (**17**). Colorless liquid was obtained in 48% isolated yield. ^{1}H NMR (400 MHz, $CDCl_3$) δ 7.39 (d, J = 4.0 Hz, 1H), 7.06–7.02 (m, 1H), 6.96 (d, J = 7.9 Hz, 1H), 5.03 (s, 1H), 4.16–4.06 (m, 2H), 3.57 (s, 3H), 2.43 (s, 3H), 2.29 (s, 3H), 1.19 (t, J = 7.2 Hz, 3H). ^{13}C NMR (101 MHz, $CDCl_3$) δ 167.58, 141.63, 139.17, 135.81, 131.36, 127.37, 127.14, 87.00, 61.66, 56.25, 21.18, 21.11, 14.03. HRMS (ESI) calculated for $C_{13}H_{18}O_4S$ $[M]^+$ 254.0965 found 254.0971.

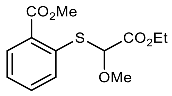

Methyl 2-((2-ethoxy-1-methoxy-2-oxoethyl)thio)benzoate (**18**). Colorless liquid was obtained in 44% isolated yield. ^{1}H NMR (400 MHz, $CDCl_3$) δ 7.86 (dd, J = 4.0, 4.0 Hz, 1H), 7.76 (dd, J = 4.0, 4.0 Hz, 1H), 7.47–7.42 (m, 1H), 7.30–7.26 (m, 1H), 5.29 (s, 1H), 4.20–4.14 (m, 2H), 3.92 (s, 3H), 3.58 (s, 3H), 1.18 (t, J = 7.2 Hz, 3H). ^{13}C NMR (101 MHz, $CDCl_3$) δ 167.64, 167.20, 136.12, 132.26,

131.16, 131.03, 130.69, 126.52, 85.11, 55.99, 52.43, 14.07. HRMS (ESI) calculated for $C_{13}H_{17}O_5S$ $[M+H]^+$ 285.0791 found 285.0788

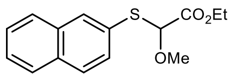

Ethyl 2-methoxy-2-(naphthalen-2-ylthio)acetate (**19**). Colorless liquid was obtained in 40% isolated yield. ^{1}H NMR (400 MHz, $CDCl_3$) δ 8.02 (d, *J* = 2.0 Hz, 1H), 7.82–7.77 (m, 3H), 7.56 (dd, *J* = 4.0, 2.0 Hz, 1H), 7.51–7.46 (m, 2H), 5.18 (s, 1H), 4.11 (q, *J* = 8.0 Hz, 2H), 3.63 (s, 3H), 1.12 (t, *J* = 8.0 Hz, 3H). ^{13}C NMR (101 MHz, $CDCl_3$) δ 167.51, 133.68, 133.56, 133.08, 130.93, 128.73, 128.62, 127.83, 127.80, 126.85, 126.65, 86.89, 76.84, 61.83, 56.27, 14.10. HRMS (ESI) calculated for $C_{15}H_{16}O_3S$ $[M]^+$ 276.0810 found 276.0815

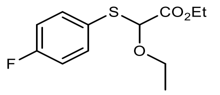

Ethyl 2-ethoxy-2-((4-fluorophenyl)thio)acetate (**20**). Colorless liquid was obtained in 55% isolated yield. ^{1}H NMR (400 MHz, $CDCl_3$) δ 7.52–7.48 (m, 2H), 7.06–7.01 (m, 2H), 5.13 (s, 1H), 4.16 (q, *J* = 7.2 Hz, 2H), 4.07–4.00 (m, 1H), 3.68–3.60 (m, 1H), 1.32 (t, *J* = 8.0 Hz, 3H), 1.22 (t, *J* = 7.2 Hz, 3H). ^{13}C NMR (101 MHz, $CDCl_3$) δ 167.43, 163.26 (d, $J_{C\text{-}F}$ = 249.47 Hz), 136.79, 136.71, 126.22 (d, $J_{C\text{-}F}$ = 4.04 Hz), 116.25, 116.03, 84.78, 84.76, 64.45, 61.67, 14.71, 14.16. ^{19}F NMR (377 MHz, $CDCl_3$) δ -112.24. HRMS (ESI) calculated for $C_{12}H_{16}O_3SF$ $[M+H]^+$ 259.0799 found 259.0795.

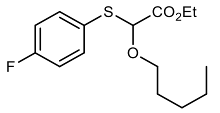

(ethoxy(pentyloxy)methyl)(4-fluorophenyl)sulfane (**21**). Colorless liquid was obtained in 50% isolated yield. ^{1}H NMR (400 MHz, $CDCl_3$) δ 7.53–7.48 (m, 2H), 7.07–7.01 (m, 2H), 5.13 (s, 1H), 4.16 (q, *J* = 7.2 Hz, 2H), 3.96–3.90 (m, 1H), 3.59–3.53 (m, 1H), 1.73–1.66 (m, 2H), 1.39–1.34 (m, 4H), 1.23 (t, *J* = 8.0 Hz, 3H), 0.95–0.90 (m, 3H). ^{13}C NMR (101 MHz, $CDCl_3$) δ 167.47, 163.34 (d, $J_{C\text{-}F}$ = 250.48 Hz), 136.69, 136.61, 126.38 (d, $J_{C\text{-}F}$ = 3.03 Hz), 116.24, 116.02, 85.03, 69.18, 61.67, 28.85, 28.30, 22.53, 14.19, 14.10. ^{19}F NMR (377 MHz, $CDCl_3$) δ -112.34. HRMS (ESI) calculated for $C_{15}H_{22}O_3SF$ $[M+H]^+$ 301.1268 found 301.1261.

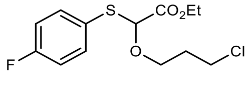

(ethoxy(2-methoxyethoxy)methyl)(4-fluorophenyl)sulfane (**22**). Colorless liquid was obtained in 36% isolated yield. ^{1}H NMR (400 MHz, $CDCl_3$) δ 7.54–7.45 (m, 2H), 7.06–6.99 (m, 2H), 5.12 (s, 1H), 4.18–4.12 (m, 2H), 4.12–4.06 (m, 1H), 3.69–3.63 (m, 3H), 2.15–2.09 (m, 2H), 1.22 (t, *J* = 7.1 Hz, 3H). ^{13}C NMR (101 MHz, $CDCl_3$) δ 167.26, 163.44 (d, $J_{C\text{-}F}$ = 250.48 Hz), 136.79, 136.70, 126.11(d, $J_{C\text{-}F}$ = 3.03 Hz), 116.36, 116.15, 85.20, 65.41, 61.81, 41.73, 32.24, 14.20. ^{19}F NMR (377 MHz, $CDCl_3$) δ -111.98. HRMS (ESI) calculated for $C_{13}H_{16}O_3SClF$ $[M]^+$ 306.0487 found 306.0487.

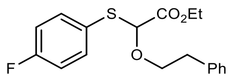

(ethoxy(pentyloxy)methyl)(4-fluorophenyl)sulfane (**23**). Colorless liquid was obtained in 45% isolated yield. ^{1}H NMR (400 MHz, $CDCl_3$) δ 7.33–7.28 (m, 4H), 7.25–7.22 (m, 3H), 6.96–6.91 (m,

2H), 5.09 (s, 1H), 4.16–4.08 (m, 3H), 3.81–3.74 (m, 1H), 2.98 (t, J = 7.0 Hz, 2H), 1.18 (t, J = 7.2 Hz, 3H). ^{13}C NMR (101 MHz, $CDCl_3$) δ 167.26, 163.31(d, $J_{C\text{-}F}$ = 249.47 Hz), 138.33, 136.69, 136.60, 129.20, 128.57, 126.58, 126.08 (d, $J_{C\text{-}F}$ = 3.03 Hz), 116.19, 115.97, 85.02, 69.64, 61.70, 35.86, 14.17. ^{19}F NMR (377 MHz, $CDCl_3$) δ -112.26. HRMS (ESI) calculated for $C_{18}H_{20}O_3SF$ $[M+H]^+$ 335.1112 found 335.1112

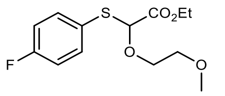

(ethoxy(2-methoxyethoxy)methyl)(4-fluorophenyl)sulfane (**24**). Colorless liquid was obtained in 68% isolated yield. ^{1}H NMR (400 MHz, $CDCl_3$) δ 7.53–7.48 (m, 2H), 7.04–6.98 (m, 2H), 5.26 (s, 1H), 4.13 (q, J = 7.2 Hz, 2H), 4.08–4.03 (m, 1H), 3.85–3.80 (m, 1H), 3.65–3.59 (m, 2H), 3.37 (s, 3H), 1.20 (t, J = 7.2 Hz, 3H). ^{13}C NMR (101 MHz, $CDCl_3$) δ 167.30, 163.37 (d, $J_{C\text{-}F}$ = 249.47 Hz), 162.13, 136.73, 136.65, 126.21 (d, $J_{C\text{-}F}$ = 4.04 Hz), 116.23, 116.01, 85.26, 71.66, 67.64, 61.68, 59.11, 14.17. ^{19}F NMR (377 MHz, $CDCl_3$) δ -112.26. HRMS (ESI) calculated for $C_{13}H_{18}O_4SF$ $[M+H]^+$ 289.0904 found 289.0902.

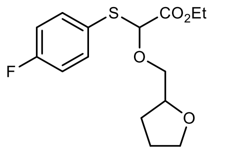

Ethyl 2-((4-fluorophenyl)thio)-2-((tetrahydrofuran-2-yl)methoxy)acetate (**25**). Colorless liquid was obtained in 41% isolated yield. ^{1}H NMR (400 MHz, $CDCl_3$) δ 7.54–7.49 (m, 2H), 7.03–6.99 (m, 2H), 5.26 (s, 1H), 4.13 (q, J = 7.2 Hz, 3H), 3.91–3.84 (m, 2H), 3.80–3.74 (m, 1H), 3.71–3.67 (m, 1H), 2.01–1.85 (m, 3H), 1.76–1.70 (m, 1H), 1.19 (t, J = 7.2 Hz, 3H). ^{13}C NMR (101 MHz, $CDCl_3$) δ 167.36, 164.39 (d, $J_{C\text{-}F}$ = 250.48 Hz), 136.73, 136.64, 126.27 (d, $J_{C\text{-}F}$ = 3.03 Hz), 116.26, 116.05, 85.28, 77.52, 70.58, 68.55, 61.70, 28.19, 25.85, 14.20. ^{19}F NMR (377 MHz, $CDCl_3$) δ -112.33. HRMS (ESI) calculated for $C_{15}H_{20}O_4SF$ $[M+H]^+$ 305.1061 found 305.1052.

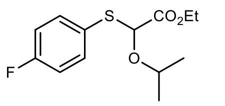

(ethoxy(isopropoxy)methyl)(4-fluorophenyl)sulfane (**26**). Colorless liquid was obtained in 44% isolated yield. ^{1}H NMR (400 MHz, $CDCl_3$) δ 7.53–7.47 (m, 2H), 7.04–6.98 (m, 2H), 5.16 (s, 1H), 4.16–4.10 (m, 3H), 1.27 (d, J = 6.2 Hz, 3H), 1.22–1.18 (m, 6H). ^{13}C NMR (101 MHz, $CDCl_3$) δ 167.85, 163.36 (d, $J_{C\text{-}F}$ = 250.48 Hz), 136.83, 136.74, 126.41 (d, $J_{C\text{-}F}$ = 4.04 Hz), 116.20, 115.99, 82.72, 82.71, 70.73, 61.64, 22.69, 20.99, 14.17. ^{19}F NMR (377 MHz, $CDCl_3$) δ -112.34. HRMS (ESI) calculated for $C_{13}H_{18}O_3SF$ $[M+H]^+$ 273.0955 found 273.0955.

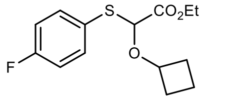

Ethyl 2-cyclobutoxy-2-((4-fluorophenyl)thio)acetate (**27**). Colorless liquid was obtained in 40% isolated yield. ^{1}H NMR (400 MHz, $CDCl_3$) δ 7.51–7.45 (m, 2H), 7.04–6.99 (m, 2H), 5.05 (s, 1H), 4.48–4.41 (m, 1H), 4.14 (q, J = 7.2 Hz, 2H), 2.31–2.13 (m, 3H), 2.03–1.93 (m, 1H), 1.75–1.67 (m, 1H), 1.57–1.50 (m, 1H), 1.21 (t, J = 7.2 Hz, 3H). ^{13}C NMR (101 MHz, $CDCl_3$) δ 167.52, 163.37 (d,

$J_{C\text{-}F}$ = 250.48 Hz), 136.79, 136.71, 126.29 (d, $J_{C\text{-}F}$ = 3.03 Hz), 116.27, 116.06, 82.53, 82.51, 71.54, 61.72, 30.42, 30.00, 14.17, 12.63. ^{19}F NMR (377 MHz, $CDCl_3$) δ -112.20. HRMS (ESI) calculated for $C_{14}H_{18}O_3SF$ $[M+H]^+$ 285.0955 found 285.0952.

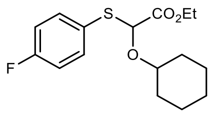

((cyclohexyloxy)(ethoxy)methyl)(4-fluorophenyl)sulfane (**28**). Colorless liquid was obtained in 44% isolated yield. ^{1}H NMR (400 MHz, $CDCl_3$) δ 7.54–7.48 (m, 2H), 7.04–6.98 (m, 2H), 5.22 (s, 1H), 4.13 (q, J = 7.2 Hz, 2H), 3.79–3.73 (m, 1H), 1.94–1.87 (m, 2H), 1.79–1.72 (m, 2H), 1.57–1.53 (m, 1H), 1.50–1.40 (m, 1H), 1.35–1.23 (m, 4H), 1.20 (t, J = 7.2 Hz, 3H). ^{13}C NMR (101 MHz, $CDCl_3$) δ 167.92, 163.35 (d, $J_{C\text{-}F}$ = 250.48 Hz), 136.80, 136.72, 126.50 (d, $J_{C\text{-}F}$ = 3.03 Hz), 116.20, 115.98, 82.41, 76.61, 61.63, 32.67, 31.14, 25.61, 24.33, 24.10, 14.20. ^{19}F NMR (377 MHz, $CDCl_3$) δ -112.39. HRMS (ESI) calculated for $C_{16}H_{22}O_3SF$ $[M+H]^+$ 313.1268 found 313.1288.

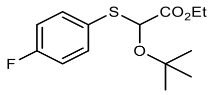

Ethyl 2-(tert-butoxy)-2-((4-fluorophenyl)thio)acetate (**29**). Colorless liquid was obtained in 32% isolated yield. ^{1}H NMR (400 MHz, $CDCl_3$) δ 7.58–7.55 (m, 2H), 7.04–6.99 (m, 2H), 5.18 (s, 1H), 4.15 (q, J = 7.2 Hz, 2H), 1.23 (t, J = 7.2 Hz, 3H), 1.18 (s, 9H). ^{13}C NMR (101 MHz, $CDCl_3$) δ 169.22, 164.45 (d, $J_{C\text{-}F}$ = 249.47 Hz), 137.48, 137.40, 126.77 (d, $J_{C\text{-}F}$ = 3.03 Hz), 116.14, 115.92, 78.87, 61.61, 27.90, 14.19. ^{19}F NMR (377 MHz, $CDCl_3$) δ -112.19. HRMS (ESI) calculated for $C_{14}H_{20}O_3SF$ $[M+H]^+$ 287.1112 found 287.1112.

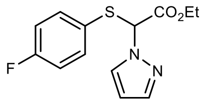

Ethyl 2-((4-fluorophenyl)thio)-2-(1H-pyrazol-1-yl)acetate (**30**). Colorless liquid was obtained in 70% isolated yield. ^{1}H NMR (400 MHz, $CDCl_3$) δ 7.72 (d, J = 2.5 Hz, 1H), 7.41 (d, J = 1.8 Hz, 1H), 7.21–7.16 (m, 2H), 6.91–6.84 (m, 2H), 6.24 (t, J = 2.2 Hz, 1H), 6.06 (s, 1H), 4.17 (q, J = 7.2 Hz, 2H), 1.21 (t, J = 7.2 Hz, 3H). ^{13}C NMR (101 MHz, $CDCl_3$) δ 166.12, 163.61 (d, $J_{C\text{-}F}$ = 249.47 Hz), 140.12, 136.70, 136.62, 129.55, 125.75 (d, $J_{C\text{-}F}$ = 3.03 Hz), 116.56, 116.34, 107.21, 69.14, 62.79, 14.03. ^{19}F NMR (377 MHz, $CDCl_3$) δ -110.76. HRMS (ESI) calculated for $C_{13}H_{14}O_2N_2SF$ $[M+H]^+$ 281.0755 found 281.0745.

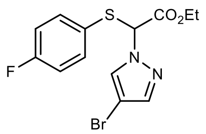

Ethyl 2-(4-bromo-1H-pyrazol-1-yl)-2-((4-fluorophenyl)thio)acetate (**31**). Colorless liquid was obtained in 55% isolated yield. ^{1}H NMR (400 MHz, $CDCl_3$) δ 7.86 (s, 1H), 7.48 (s, 1H), 7.30–7.26 (m, 2H), 7.02–6.97 (m, 2H), 6.07 (s, 1H), 4.26 (q, J = 7.2 Hz, 2H), 1.31 (t, J = 7.2 Hz, 3H). ^{13}C NMR (101 MHz, $CDCl_3$) δ 165.81, 163.81 (d, $J_{C\text{-}F}$ = 251.49 Hz), 145.09, 136.84, 136.76, 134.08, 125.40 (d, $J_{C\text{-}F}$ = 4.04 Hz), 116.79, 116.57, 69.50, 63.07, 58.24, 14.10. ^{19}F NMR (377 MHz, $CDCl_3$) δ -110.19. HRMS (ESI) calculated for $C_{13}H_{13}O_2N_2SFBr$ $[M+H]^+$ 358.9860 found 358.9850.

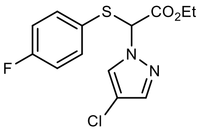

Ethyl 2-(4-chloro-1H-pyrazol-1-yl)-2-((4-fluorophenyl)thio)acetate (**32**). Colorless liquid was obtained in 55% isolated yield. ^{1}H NMR (400 MHz, $CDCl_3$) δ 7.83 (s, 1H), 7.39 (s, 1H), 7.32–7.28 (m, 2H), 7.03–6.97 (m, 2H), 6.02 (s, 1H), 4.27 (q, *J* = 7.2 Hz, 2H), 1.31 (t, *J* = 7.2 Hz, 3H). ^{13}C NMR (101 MHz, $CDCl_3$) δ 165.77, 163.82 (d, $J_{C\text{-}F}$ = 252.5 Hz), 138.65, 136.80, 136.71, 127.57, 125.40 (d, $J_{C\text{-}F}$ = 3.03 Hz), 116.83, 116.61, 111.80, 69.97, 63.09, 14.10. ^{19}F NMR (377 MHz, $CDCl_3$) δ -110.21. HRMS (ESI) calculated for $C_{13}H_{13}O_2N_2SFCl$ $[M+H]^+$ 315.0365 found 315.0360.

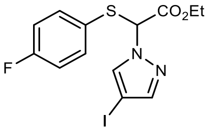

Ethyl 2-((4-fluorophenyl)thio)-2-(4-iodo-1H-pyrazol-1-yl)acetate (**33**). Colorless liquid was obtained in 41% isolated yield. ^{1}H NMR (400 MHz, $CDCl_3$) δ 7.86 (s, 1H), 7.48 (s, 1H), 7.30–7.25 (m, 2H), 7.02–6.97 (m, 2H), 6.07 (s, 1H), 4.27 (q, *J* = 7.2 Hz, 2H), 1.31 (t, *J* = 7.2 Hz, 3H). ^{13}C NMR (101 MHz, $CDCl_3$) δ 165.80, 163.79 (d, $J_{C\text{-}F}$ = 251.49 Hz), 145.07, 136.83, 136.75, 134.07, 125.38 (d, $J_{C\text{-}F}$ = 3.03 Hz), 116.78, 116.56, 69.48, 63.06, 58.24, 14.09. ^{19}F NMR (377 MHz, $CDCl_3$) δ -110.20. HRMS (ESI) calculated for $C_{13}H_{13}O_2N_2SFI$ $[M+H]^+$ 406.9721 found 406.9714.

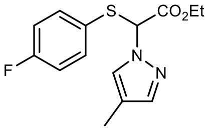

Ethyl 2-((4-fluorophenyl)thio)-2-(4-methyl-1H-pyrazol-1-yl)acetate (**34**). Colorless liquid was obtained in 48% isolated yield. ^{1}H NMR (400 MHz, $CDCl_3$) δ 7.58 (s, 1H), 7.30–7.28 (m, 2H), 7.27 (s, 1H), 6.99–6.94 (m, 2H), 6.04 (s, 1H), 4.25 (q, *J* = 7.2 Hz, 2H), 2.08 (s, 3H), 1.30 (t, *J* = 7.2 Hz, 3H). ^{13}C NMR (101 MHz, $CDCl_3$) δ 166.39, 163.61 (d, $J_{C\text{-}F}$ = 251.49 Hz), 140.88, 136.54, 136.46, 128.00, 126.17 (d, $J_{C\text{-}F}$ = 4.04 Hz), 117.96, 116.59, 116.37, 69.31, 62.80, 14.11, 9.18. ^{19}F NMR (377 MHz, $CDCl_3$) δ -111.08. HRMS (ESI) calculated for $C_{14}H_{16}O_2N_2SF$ $[M+H]^+$ 295.0911 found 295.0908.

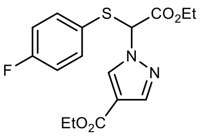

Ethyl 1-(2-ethoxy-1-((4-fluorophenyl)thio)-2-oxoethyl)-1H-pyrazole-4-carboxylate (**35**). Colorless liquid was obtained in 37% isolated yield. ^{1}H NMR (400 MHz, $CDCl_3$) δ 8.31 (s, 1H), 7.88 (s, 1H), 7.33–7.29 (m, 2H), 7.03–6.97 (m, 2H), 6.08 (s, 1H), 4.29 (dq, *J* = 9.2, 7.2 Hz, 4H), 1.33 (dt, *J* = 14.4, 7.2 Hz, 6H). ^{13}C NMR (101 MHz, $CDCl_3$) δ 165.63, 163.87 (d, $J_{C\text{-}F}$ = 252.5 Hz), 162.70, 141.48, 136.81, 136.73, 133.19, 125.25 (d, $J_{C\text{-}F}$ = 3.03 Hz), 116.93, 116.71, 69.52, 63.22, 60.52, 14.46, 14.09. ^{19}F NMR (377 MHz, $CDCl_3$) δ -110.03. HRMS (ESI) calculated for $C_{16}H_{18}O_4N_2SF^+$ $[M+H]^+$ 358.0966 found 358.0958.

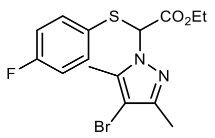

Ethyl 2-(4-bromo-3,5-dimethyl-1H-pyrazol-1-yl)-2-((4-fluorophenyl)thio)acetate (**36**). Colorless liquid was obtained in 43% isolated yield. ^{1}H NMR (400 MHz, $CDCl_3$) δ 7.28–7.25 (m, 2H), 6.98–6.94 (m, 2H), 5.98 (s, 1H), 4.29 (q, J = 7.2 Hz, 2H), 2.18 (s, 3H), 2.01 (s, 3H), 1.29 (t, J = 7.2 Hz, 3H). ^{13}C NMR (101 MHz, $CDCl_3$) δ 165.70, 163.66 (d, $J_{C\text{-}F}$ = 251.49 Hz), 147.83, 138.69, 137.32, 137.23, 125.94 (d, $J_{C\text{-}F}$ = 4.04 Hz), 116.52, 116.30, 96.08, 69.05, 63.17, 14.09, 12.46, 10.55. ^{19}F NMR (377 MHz, $CDCl_3$) δ -110.79. HRMS (ESI) calculated for $C_{15}H_{17}O_2N_2SFBr^+$ $[M+H]^+$ 387.0173 found 387.0167.

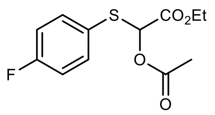

Ethyl 2-acetoxy-2-((4-fluorophenyl)thio)acetate (**37**). Colorless liquid was obtained in 55% isolated yield. ^{1}H NMR (400 MHz, $CDCl_3$) δ 7.56–7.52 (m, 2H), 7.07–7.02 (m, 2H), 6.13 (s, 1H), 4.13 (q, J = 7.2 Hz, 2H), 2.18 (s, 3H), 1.21 (t, J = 7.2 Hz, 3H). ^{13}C NMR (101 MHz, $CDCl_3$) δ 169.78, 165.88, 163.74 (d, $J_{C\text{-}F}$ = 251.49 Hz), 137.14, 137.06, 124.97 (d, $J_{C\text{-}F}$ = 4.04 Hz), 116.49, 116.27, 76.65, 62.26, 20.86, 14.10. ^{19}F NMR (377 MHz, $CDCl_3$) δ -110.93. HRMS (ESI) calculated for $C_{12}H_{13}O_4SF$ $[M]^+$ 272.0513 found 272.0512.

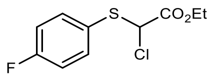

Ethyl 2-chloro-2-((4-fluorophenyl)thio)acetate (**38**). Colorless liquid was obtained in 37% isolated yield. ^{1}H NMR (400 MHz, $CDCl_3$) δ 7.61–7.57 (m, 2H), 7.12–7.06 (m, 2H), 5.45 (s, 1H), 4.23 (q, J = 7.1 Hz, 2H), 1.29 (t, J = 7.1 Hz, 3H). ^{13}C NMR (101 MHz, CDCl3) δ 165.89, 164.07 (d, $J_{C\text{-}F}$ = 251.49 Hz), 137.35, 137.26, 125.28 (d, $J_{C\text{-}F}$ = 4.04 Hz), 116.73, 116.52, 64.86, 63.08, 14.06. ^{19}F NMR (377 MHz, $CDCl_3$) δ -110.22. HRMS (ESI) calculated for $C_{10}H_{11}O_2SFCl$ $[M]^+$ 248.0069 found 248.0070.

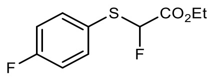

Ethyl 2-fluoro-2-((4-fluorophenyl)thio)acetate (**39**). Colorless liquid was obtained in 30% isolated yield. 1H NMR (400 MHz, $CDCl_3$) δ 7.59–7.52 (m, 2H), 7.10–7.02 (m, 2H), 6.01 (d, J = 51.6 Hz, 1H), 4.14 (q, J = 7.1 Hz, 2H), 1.20 (t, J = 7.2 Hz, 3H). ^{13}C NMR (101 MHz, $CDCl_3$) δ 165.38 (d, $J_{C\text{-}F}$ = 29.29 Hz), 163.93 (d, $J_{C\text{-}F}$ = 251.49 Hz), 137.05 (d, $J_{C\text{-}F}$ = 2.02 Hz), 136.96 (d, $J_{C\text{-}F}$ = 2.02 Hz), 124.57, 116.66, 116.44, 94.21 (d, $J_{C\text{-}F}$ = 233.31 Hz), 62.48, 14.11. ^{19}F NMR (377 MHz, $CDCl_3$) δ -110.68, -159.15. HRMS (ESI) calculated for $C_{10}H_{11}O_2SF_2$ $[M]^+$ 232.0364 found 232.0366.

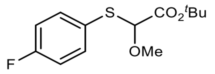

Tert-butyl 2-((4-fluorophenyl)thio)-2-methoxyacetate (**40**). Colorless liquid was obtained in 68% isolated yield. ^{1}H NMR (400 MHz, $CDCl_3$) δ 7.51–7.47 (m, 2H), 7.03–6.98 (m, 2H), 4.90 (s, 1H),

3.56 (s, 3H), 1.39 (s, 9H). ^{13}C NMR (101 MHz, $CDCl_3$) δ 166.15, 163.17 (d, $J_{C\text{-}F}$ = 249.47 Hz), 136.43, 136.35, 126.56 (d, $J_{C\text{-}F}$ = 4.04 Hz), 116.09, 115.88, 86.71, 82.58, 56.01, 27.91. ^{19}F NMR (377 MHz, $CDCl_3$) δ -112.60. HRMS (ESI) calculated for $C_{13}H_{18}O_3SF$ $[M+H]^+$ 273.0955 found 273.0956.

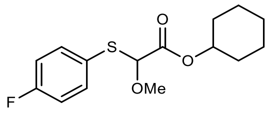

Cyclohexyl 2-((4-fluorophenyl)thio)-2-methoxyacetate (**41**). Colorless liquid was obtained in 87% isolated yield. ^{1}H NMR (400 MHz, $CDCl_3$) δ 7.51–7.43 (m, 2H), 7.04–6.98 (m, 2H), 5.00 (s, 1H), 4.78–4.72 (m, 1H), 3.58 (s, 3H), 1.82–1.65 (m, 4H), 1.56–1.50 (m, 1H), 1.43–1.22 (m, 5H). ^{13}C NMR (101 MHz, $CDCl_3$) δ 166.69, 163.36 (d, $J_{C\text{-}F}$ = 249.47 Hz), 136.68, 136.60, 126.15 (d, $J_{C\text{-}F}$ = 3.03 Hz), 116.27, 116.05, 86.44, 74.35, 56.16, 31.56, 31.45, 25.32, 23.77. ^{19}F NMR (377 MHz, $CDCl_3$) δ -112.26. HRMS (ESI) calculated for $C_{15}H_{20}O_3SF$ $[M+H]^+$ 299.1112 found 299.1114.

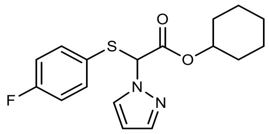

Cyclohexyl 2-((4-fluorophenyl)thio)-2-(1H-pyrazol-1-yl)acetate (**42**). Colorless liquid was obtained in 50% isolated yield. ^{1}H NMR (400 MHz, $CDCl_3$) δ 7.82 (d, *J* = 2.5 Hz, 1H), 7.50 (d, *J* = 1.8 Hz, 1H), 7.33–7.24 (m, 2H), 7.03–6.92 (m, 2H), 6.33 (t, *J* = 2.2 Hz, 1H), 6.14 (s, 1H), 4.93–4.82 (m, 1H), 1.88–1.79 (m, 2H), 1.78–1.67 (m, 2H), 1.57–1.47 (m, 3H), 1.47–1.25 (m, 4H). ^{13}C NMR (101 MHz, $CDCl_3$) δ 165.55, 163.59 (d, $J_{C\text{-}F}$ = 249.47 Hz), 140.06, 136.63, 136.55, 129.56, 125.95 (d, $J_{C\text{-}F}$ = 3.03 Hz), 116.55, 116.33, 107.16, 75.47, 69.38 (d, $J_{C\text{-}F}$ = 2.02 Hz) 31.22, 31.19, 25.25, 23.40. ^{19}F NMR (377 MHz, $CDCl_3$) δ -110.96. HRMS (ESI) calculated for $C_{17}H_{20}O_2N_2SF^+$ $[M+H]^+$ 335.1224 found 335.1216.

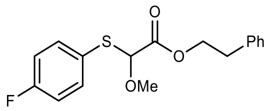

Phenethyl 2-((4-fluorophenyl)thio)-2-methoxyacetate (**43**). Colorless liquid was obtained in 61% isolated yield.^{1}H NMR (400 MHz, $CDCl_3$) δ 7.36–7.20 (m, 4H), 7.26–7.24 (m, 1H), 7.22–7.20 (m, 2H), 6.99–6.95 (m, 2H), 5.01 (s, 1H), 4.28–4.24 (m, 2H), 3.56 (s, 3H), 2.88 (t, *J* = 8.0 Hz, 2H). ^{13}C NMR (101 MHz, $CDCl_3$) δ 167.20, 163.44 (d, $J_{C\text{-}F}$ = 249.47 Hz), 137.40, 136.93, 136.85, 129.09, 128.68, 126.86, 125. 84 (d, $J_{C\text{-}F}$ = 3.03 Hz), 116.33, 116.11, 86.38, 66.16, 56.18, 34.97. ^{19}F NMR (377 MHz, $CDCl_3$) δ -111.87. HRMS (ESI) calculated for $C_{17}H_{17}O_3SF$ $[M]^+$ 320.0877 found 320.0876.

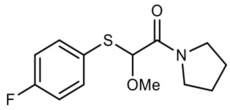

2-((4-fluorophenyl)thio)-2-methoxy-1-(pyrrolidin-1-yl)ethan-1-one (**44**). Colorless liquid was obtained in 52% isolated yield. ^{1}H NMR (400 MHz, $CDCl_3$) δ 7.52–7.41 (m, 2H), 7.03–6.95 (m, 2H), 5.22 (s, 1H), 3.67–3.59 (m, 1H), 3.51 (s, 3H), 3.49–3.37 (m, 3H), 1.97–1.80 (m, 4H). ^{13}C NMR (101 MHz, $CDCl_3$) δ 164.77, 163.03 (d, $J_{C\text{-}F}$ = 249.47 Hz), 136.16, 136.08, 127.13 (d, $J_{C\text{-}F}$ = 4.04

Hz), 116.13, 115.92, 88.40, 55.26, 46.36, 46.31, 26.24, 23.80. ^{19}F NMR (377 MHz, $CDCl_3$) δ -112.71. HRMS (ESI) calculated for $C_{13}H_{17}O_2NSF$ $[M+H]^+$ 270.0959 found 270.0956.

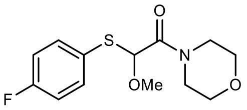

2-((4-fluorophenyl)thio)-2-methoxy-1-morpholinoethan-1-one (**45**). Colorless liquid was obtained in 55% isolated yield. ^{1}H NMR (400 MHz, $CDCl_3$) δ 7.58–7.41 (m, 2H), 7.08–6.99 (m, 2H), 5.32 (s, 1H), 3.74–3.67 (m, 4H), 3.66–3.54 (m, 4H), 3.50 (s, 3H). ^{13}C NMR (101 MHz, $CDCl_3$) δ 165.34, 163.18 (d, $J_{C\text{-}F}$ = 250.48 Hz), 135.95, 135.86, 127.87 (d, $J_{C\text{-}F}$ = 3.03 Hz), 116.50, 116.28, 89.83, 66.98, 66.80, 55.69, 46.41, 42.76.^{19}F NMR (377 MHz, $CDCl_3$) δ -112.41. HRMS (ESI) calculated for $C_{13}H_{17}O_3SNF^+$ $[M+H]^+$ 286.0908 found 286.0913.

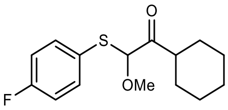

1-cyclohexyl-2-((4-fluorophenyl)thio)-2-methoxyethan-1-one (**46**). Colorless liquid was obtained in 60% isolated yield. ^{1}H NMR (400 MHz, $CDCl_3$) δ 7.43–7.39 (m, 2H), 7.02–6.96 (m, 2H), 5.05 (s, 1H), 3.59 (s, 3H), 2.72–2.66 (m, 1H), 1.77–1.72 (m, 3H), 1.67–1.64 (m, 1H), 1.41–1.16 (m, 6H). ^{13}C NMR (101 MHz, $CDCl_3$) δ 204.18, 163.22 (d, $J_{C\text{-}F}$ = 250.48 Hz), 136.47, 136.38, 125.96 (d, $J_{C\text{-}F}$ = 3.03 Hz), 116.33, 116.11, 90.94, 56.24, 46.63, 29.49, 28.08, 25.85, 25.74, 25.43. ^{19}F NMR (377 MHz, $CDCl_3$) δ -112.23. HRMS (ESI) calculated for $C_{15}H_{20}O_2SF$ $[M+H]^+$ 283.1163 found 283.1162.

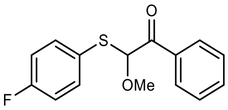

2-((4-fluorophenyl)thio)-2-methoxy-1-phenylethan-1-one (**47**). Colorless liquid was obtained in 60% isolated yield. ^{1}H NMR (400 MHz, $CDCl_3$) δ 7.95–7.92 (m, 2H), 7.60–7.58 (m, 1H), 7.49–7.43 (m, 2H), 7.33–7.28 (m, 2H), 7.00–6.94 (m, 2H), 5.83 (s, 1H), 3.69 (s, 3H). ^{13}C NMR (101 MHz, $CDCl_3$) δ 190.26, 163.49 (d, $J_{C\text{-}F}$ = 250.48 Hz), 137.05, 136.96, 134.41, 133.60, 128.89, 128.68, 125.61 (d, $J_{C\text{-}F}$ = 3.03 Hz), 116.42, 116.21, 89.35, 56.25. ^{19}F NMR (377 MHz, $CDCl_3$) δ -111.74. HRMS (ESI) calculated for $C_{15}H_{14}O_2SF$ $[M+H]^+$ 277.0693 found 277.0693.

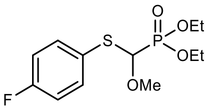

Diethyl (((4-fluorophenyl)thio)(methoxy)methyl)phosphonate (**48**). Colorless liquid was obtained in 68% isolated yield. ^{1}H NMR (400 MHz, $CDCl_3$) δ 7.59–7.56 (m, 2H), 7.05–7.01 (m, 2H), 4.81 (dd, J = 10.6, 1.6 Hz, 1H), 4.24–4.14 (m, 4H), 3.57 (s, 3H), 1.35–1.30 (m, 6H). ^{13}C NMR (101 MHz, $CDCl_3$) δ 163.05 (d, $J_{C\text{-}F}$ = 249.47 Hz), 135.86, 135.78, 128.79 (d, $J_{C\text{-}F}$ = 3.03 Hz), 116.34, 116.12, 87.61, 85.79, 63.67, 63.61, 63.54, 57.27, 57.16, 16.57, 16.51. ^{19}F NMR (377 MHz, $CDCl_3$) δ -112.78. ^{31}P NMR (162 MHz, $CDCl_3$) δ 15.37. HRMS (ESI) calculated for $C_{12}H_{19}O_4SPF$ $[M+H]^+$ 309.0720 found 309.0714.

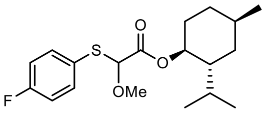

(*1S, 2R, 4R*)-2-isopropyl-4-methylcyclohexyl 2-((4-fluorophenyl)thio)-2-methoxyacetate (**49**). Colorless liquid was obtained in 61% isolated yield. ^{1}H NMR (400 MHz, $CDCl_3$) δ 7.54–7.46 (m, 2H), 7.08–6.99 (m, 2H), 5.02 (s, 1H), 4.74–4.64 (m, 1H), 3.61 (s, 3H), 2.03–1.88 (m, 2H), 1.73–1.66 (m, 2H), 1.63 (d, *J* = 1.6 Hz, 1H), 1.51–1.39 (m, 2H), 1.10–0.99 (m, 1H), 0.94–0.88 (m, 7H), 0.77–0.71 (m, 3H). ^{13}C NMR (101 MHz, $CDCl_3$) δ 166.92, 163.26 (d, J_{C-F} = 250.48 Hz), 136.21, 136.13, 126.45 (d, J_{C-F} = 3.03 Hz), 116.28, 116.06, 86.45, 76.01, 56.15, 46.97, 40.62, 34.19, 31.45, 25.92, 23.17, 22.06, 20.98, 16.18. ^{19}F NMR (377 MHz, $CDCl_3$) δ -112.67. HRMS (ESI) calculated for $C_{19}H_{28}O_3SF^+$ [M+H] 355.1738 found 355.1748.

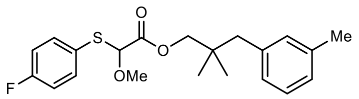

2, 2-dimethyl-3-(m-tolyl)propyl 2-((4-fluorophenyl)thio)-2-methoxyacetate (**50**). Colorless liquid was obtained in 62% isolated yield. ^{1}H NMR (400 MHz, $CDCl_3$) δ 7.46–7.36 (m, 2H), 7.08 (t, *J* = 7.5 Hz, 1H), 6.98–6.91 (m, 3H), 6.88–6.81 (m, 2H), 5.02 (s, 1H), 3.71 (s, 2H), 3.53 (s, 3H), 2.46 (s, 2H), 2.25 (s, 3H), 0.82 (s, 6H). ^{13}C NMR (101 MHz, $CDCl_3$) δ 167.38, 163.36 (d, J_{C-F} = 250.48 Hz), 137.88, 137.54, 136.50, 136.41, 131.43, 127.93, 127.68, 127.05, 126.26 (d, J_{C-F} = 4.04 Hz), 116.42, 116.20, 86.35, 72.84, 56.14, 44.90, 35.07, 24.50, 24.28, 21.55. HRMS (ESI) calculated for $C_{21}H_{25}O_3SF$ $[M]^+$ 376.1503 found 376.1492.

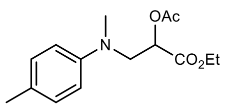

Ethyl 2-acetoxy-3-(methyl(p-tolyl)amino)propanoate (**51**). Colorless liquid was obtained in 69% isolated yield. ^{1}H NMR (400 MHz, $CDCl_3$) δ 7.06–7.04 (m, 2H), 6.71–6.67 (m, 2H), 5.27 (dd, *J* = 8.3, 3.8 Hz, 1H), 4.23–4.14 (m, 2H), 3.81 (dd, *J* = 15.4, 4.0 Hz, 1H), 3.70 (dd, *J* = 15.4, 8.3 Hz, 1H), 2.97 (s, 3H), 2.25 (s, 3H), 2.07 (s, 3H), 1.27 (t, *J* = 7.2, 3H). ^{13}C NMR (101 MHz, $CDCl_3$) δ 170.37, 169.25, 146.57, 129.87, 126.55, 112.84, 71.30, 61.78, 54.06, 39.22, 20.77, 20.34, 14.22. HRMS (ESI) calculated for $C_{15}H_{22}O_4N^+$ $[M+H]^+$ 280.1543 found 280.1535.

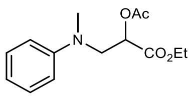

Ethyl 2-acetoxy-3-(methyl(phenyl)amino)propanoate (**52**). Colorless liquid was obtained in 47% isolated yield. ^{1}H NMR (400 MHz, $CDCl_3$) δ 7.26–7.22 (m, 2H), 6.78–6.72 (m, 3H), 5.28 (dd, *J* = 8.3, 4.0 Hz, 1H), 4.23–4.14 (m, 2H), 3.85 (dd, *J* = 15.4, 4.0 Hz, 1H), 3.75 (dd, *J* = 15.4, 8.4 Hz, 1H), 3.00 (s, 3H), 2.07 (s, 3H), 1.27 (d, *J* = 7.2 Hz, 3H). ^{13}C NMR (101 MHz, $CDCl_3$) δ 170.37, 169.16, 148.55, 129.36, 117.28, 112.51, 71.22, 61.83, 53.65, 39.09, 20.75, 14.20. HRMS (ESI) calculated for $C_{14}H_{20}O_4N$ $[M+H]^+$ 266.1387 found 266.1380.

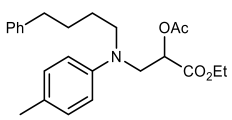

Ethyl 2-acetoxy-3-((4-phenylbutyl)(p-tolyl)amino)propanoate (**53**). Colorless liquid was obtained in 48% isolated yield. ^{1}H NMR (400 MHz, $CDCl_3$) δ 7.30–7.25 (m, 2H), 7.20–7.16 (m, 3H), 7.05–7.01 (m, 2H), 6.66–6.63 (m, 2H), 5.21 (dd, *J* = 8.6, 3.8 Hz, 1H), 4.23–4.15 (m, 2H), 3.79

(dd, J = 15.4, 3.8 Hz, 1H), 3.63 (dd, J = 15.4, 8.6 Hz, 1H), 3.32 (t, J = 7.0 Hz, 2H), 2.63 (t, J = 7.0 Hz, 2H), 2.24 (s, 3H), 2.04 (s, 3H), 1.67–1.60 (m, 4H), 1.26 (t, J = 7.2Hz, 3H). ^{13}C NMR (101 MHz, $CDCl_3$) δ 170.39, 169.30, 145.20, 142.33, 129.97, 128.49, 128.47, 126.20, 125.94, 112.89, 71.37, 61.76, 52.53, 51.69, 35.90, 29.02, 26.70, 20.73, 20.30, 14.23. HRMS (ESI) calculated for $C_{24}H_{32}O_4N$ $[M+H]^+$ 398.2326 found 398.2319.

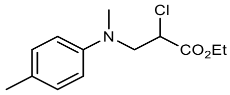

Ethyl 2-chloro-3-(methyl(p-tolyl)amino)propanoate (**54**). Colorless liquid was obtained in 73% isolated yield. ^{1}H NMR (400 MHz, $CDCl_3$) δ 7.08–7.04 (m, 2H), 6.67–6.63 (m, 2H), 4.47 (dd, J = 8.2, 6.2 Hz, 1H), 4.23–4.14 (m, 2H), 3.97 (dd, J = 15.0, 8.1 Hz, 1H), 3.69 (dd, J = 15.0, 6.0 Hz, 1H), 2.98 (s, 3H), 2.26 (s, 3H), 1.26 (t, J = 7.2 Hz, 3H). ^{13}C NMR (101 MHz, $CDCl_3$) δ 169.27, 146.05, 130.01, 126.83, 112.60, 62.40, 57.18, 53.42, 39.64, 20.34, 14.09. HRMS (ESI) calculated for $C_{13}H_{19}O_2NCl$ $[M+H]^+$ 256.1099 found 256.1095.

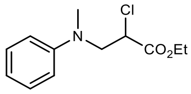

Ethyl 2-chloro-3-(methyl(phenyl)amino)propanoate (**55**). Colorless liquid was obtained in 47% isolated yield. ^{1}H NMR (400 MHz, $CDCl_3$) δ 7.28–7.24 (m, 2H), 6.78–6.71 (m, 3H), 4.49 (dd, J = 8.0, 6.1 Hz, 1H), 4.23–4.13 (m, 2H), 4.01 (dd, J = 15.2, 8.0 Hz, 1H), 3.72 (dd, J = 15.1, 6.1 Hz, 1H), 3.01 (s, 3H), 1.26 (t, J = 7.1 Hz, 3H). ^{13}C NMR (101 MHz, $CDCl_3$) δ 169.15, 148.06, 129.48, 117.51, 112.29, 62.43, 56.84, 53.36, 39.51, 14.06. HRMS (ESI) calculated for $C_{12}H_{16}O_2NCl$ $[M+H]^+$ 242.0942 found 242.0944.

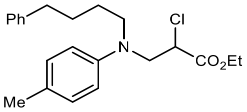

Ethyl 2-chloro-3-((4-phenylbutyl)(p-tolyl)amino)propanoate (**56**). Colorless liquid was obtained in 55% isolated yield. ^{1}H NMR (400 MHz, $CDCl_3$) δ 7.29–7.24 (m, 2H), 7.19–7.14 (m, 3H), 7.05 (d, J = 8.3 Hz, 2H), 6.65–6.58 (m, 2H), 4.41 (dd, J = 8.4, 5.7 Hz, 1H), 4.16 (p, J = 7.1 Hz, 2H), 3.91 (dd, J = 15.0, 8.3 Hz, 1H), 3.69–3.60 (m, 1H), 3.34 (m, 2H), 2.61 (t, J = 7.3 Hz, 2H), 2.25 (s, 3H), 1.66–1.55 (m, 4H), 1.24 (t, J = 7.1 Hz, 3H). ^{13}C NMR (101 MHz, $CDCl_3$) δ 169.33, 144.73, 142.31, 130.10, 128.49, 128.46, 126.75, 125.92, 113.23, 62.33, 55.66, 53.20, 52.07, 35.83, 28.95, 26.58, 20.33, 14.09. HRMS (ESI) calculated for $C_{22}H_{29}O_2NCl$ $[M+H]^+$ 374.1881 found 374.1880.

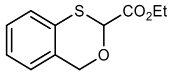

Ethyl 4H-benzo[d][1,3]oxathiine-2-carboxylate (**57**). Colorless liquid was obtained in 21% isolated yield. ^{1}H NMR (400 MHz, $CDCl_3$) δ 7.23–7.18 (m, 2H), 7.17–7.12 (m, 1H), 7.08 (d, J = 7.5 Hz, 1H), 5.68 (s, 1H), 5.07–4.87 (m, 2H), 4.30 (q, J = 7.1 Hz, 2H), 1.32 (t, J = 7.1 Hz, 3H). ^{13}C NMR (101 MHz, CDCl3) δ 167.95, 131.37, 130.75, 128.03, 127.88, 126.25, 125.72, 75.96, 68.96, 62.40, 14.23. HRMS (ESI) calculated for $C_{11}H_{13}O_3S$ $[M+H]^+$ 225.0580 found 225.0582.

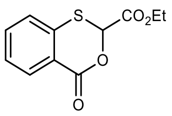

Ethyl 4-oxo-4H-benzo[d][1,3]oxathiine-2-carboxylate (**58**). Colorless liquid was obtained in 30% isolated yield. ^{1}H NMR (400 MHz, $CDCl_3$) δ 8.19 (dd, *J* = 7.9, 1.5 Hz, 1H), 7.50 (td, *J* = 7.6, 1.5 Hz, 1H), 7.37–7.32 (m, 1H), 7.30–7.26 (m, 1H), 5.94 (s, 1H), 4.29–4.16 (m, 2H), 1.22 (t, *J* = 7.1 Hz, 3H). ^{13}C NMR (101 MHz, $CDCl_3$) δ 166.35, 161.86, 134.11, 133.69, 132.17, 127.58, 127.46, 124.43, 75.41, 63.24, 14.00. HRMS (ESI) calculated for $C_{11}H_{11}O_4S$ $[M+H]^+$ 239.0373 found 239.0372.

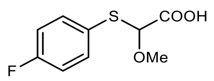

2-((4-fluorophenyl)thio)-2-methoxyacetic acid (**59**). Colorless liquid was obtained in 75% isolated yield. ^{1}H NMR (400 MHz, $CDCl_3$) δ 7.50–7.47 (m, 2H), 7.05–7.00 (m, 2H), 5.04 (s, 1H), 3.63 (s, 3H). ^{13}C NMR (101 MHz, $CDCl_3$) δ 171.34, 163.72 (d, $J_{C\text{-}F}$ = 250.48 Hz), 137.39, 137.31, 124.96 (d, $J_{C\text{-}F}$ = 4.04 Hz), 116.58, 116.36, 85.85, 56.29. ^{19}F NMR (377 MHz, $CDCl_3$) δ -111.18. HRMS (ESI) calculated for $C_9H_9O_3FS^+$ $[M]^+$ 216.0251 found 216.049.

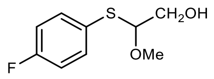

2-((4-fluorophenyl)thio)-2-methoxyethan-1-ol (**60**). Colorless liquid was obtained in 80% isolated yield. ^{1}H NMR (400 MHz, $CDCl_3$) δ 7.47–7.43 (m, 2H), 7.04–6.99 (m, 2H), 4.56–4.53 (m, 1H), 3.64–3.60 (m, 2H), 3.58 (s, 3H), 2.16–2.07 (m, 1H). ^{13}C NMR (101 MHz, $CDCl_3$) δ 163.11 (d, $J_{C\text{-}F}$ = 249.47 Hz), 136.87, 136.78, 126.22 (d, $J_{C\text{-}F}$ = 4.04 Hz), 116.32, 116.10, 90.86, 64.42, 56.75. ^{19}F NMR (377 MHz, $CDCl_3$) δ -112.79. HRMS (ESI) calculated for $C_9H_{12}O_2FS$ $[M+H]^+$ 203.0537 found 203.0537.

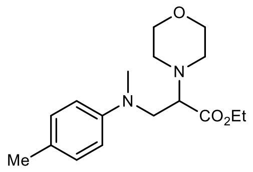

Ethyl 3-(methyl(p-tolyl)amino)-2-morpholinopropanoate (**61**). Colorless liquid was obtained in 50% isolated yield. ^{1}H NMR (400 MHz, $CDCl_3$) δ 7.09–7.00 (m, 2H), 6.71–6.62 (m, 2H), 4.21–4.09 (m, 2H), 3.78–3.72 (m, 1H), 3.72–3.61 (m, 4H), 3.55–3.45 (m, 2H), 2.92 (s, 3H), 2.74–2.67 (m, 2H), 2.62–2.55 (m, 2H), 2.25 (s, 3H), 1.25 (t, *J* = 7.1 Hz, 3H). ^{13}C NMR (101 MHz, $CDCl_3$) δ 171.11, 147.10, 129.83, 126.08, 112.91, 67.48, 65.41, 60.61, 53.03, 50.51, 39.56, 20.34, 14.60. HRMS (ESI) calculated for $C_{17}H_{26}O_3N_2$ $[M+H]^+$ 307.2016 found 307.2008.

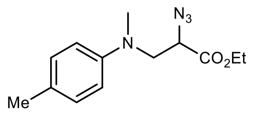

Ethyl 2-azido-3-(methyl(p-tolyl)amino)propanoate (**62**). Colorless liquid was obtained in 61% isolated yield. ^{1}H NMR (400 MHz, $CDCl_3$) δ 7.18–7.03 (m, 2H), 6.79–6.67 (m, 2H), 4.39–4.16 (m, 3H), 3.94 (dd, *J* = 15.1, 4.8 Hz, 1H), 3.55 (dd, *J* = 15.1, 8.2 Hz, 1H), 3.04 (s, 3H), 2.31 (s, 3H), 1.35 (t, *J* = 7.2 Hz, 3H). ^{13}C NMR (101 MHz, $CDCl_3$) δ 169.47, 146.09, 130.02, 126.83, 112.79, 62.19,

60.50, 54.88, 39.61, 20.34, 14.26. HRMS (ESI) calculated for $C_{13}H_{18}O_2N_4$ $[M+H]^+$ 262.1503 found 263.1495.

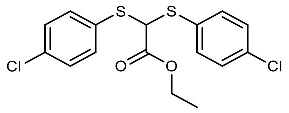

Ethyl 2,2-bis((4-chlorophenyl)thio)acetate (**63**). Colorless liquid was obtained in 8% isolated yield. ^1H NMR (400 MHz, $CDCl_3$) δ 7.43–7.40 (m, 4H), 7.32–7.29 (m, 4H), 4.75 (s, 1H), 4.16 (q, J = 7.2 Hz, 2H), 1.21 (t, J = 7.2 Hz, 3H). ^{13}C NMR (101 MHz, $CDCl_3$) δ 168.24, 135.40, 135.05, 130.85, 129.45, 62.52, 58.52, 14.12. HRMS (ESI) calculated for $C_{16}H_{14}O_2Cl_2S_2Na$ $[M+Na]^+$ 394.9704 found 394.9696.

3.5 NMR spectra

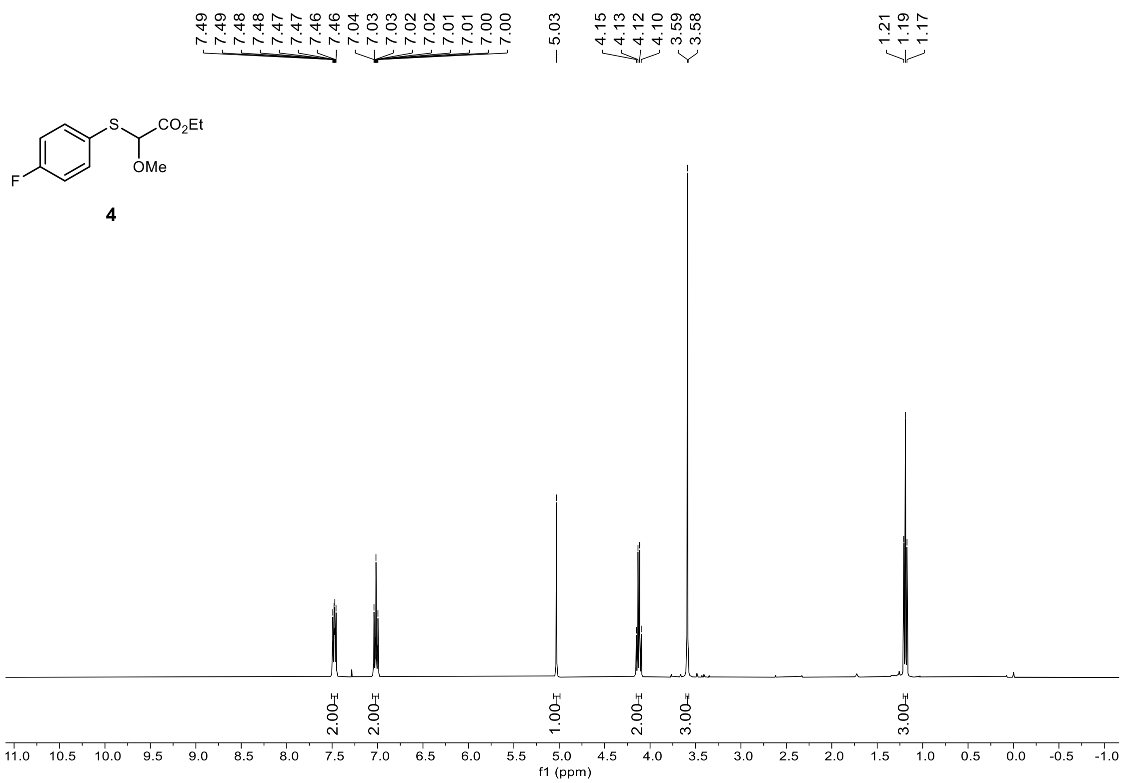

Supplementary Figure 15. ^{1}H NMR spectrum of compound **4** (400 MHz, r.t., $CDCl_3$)

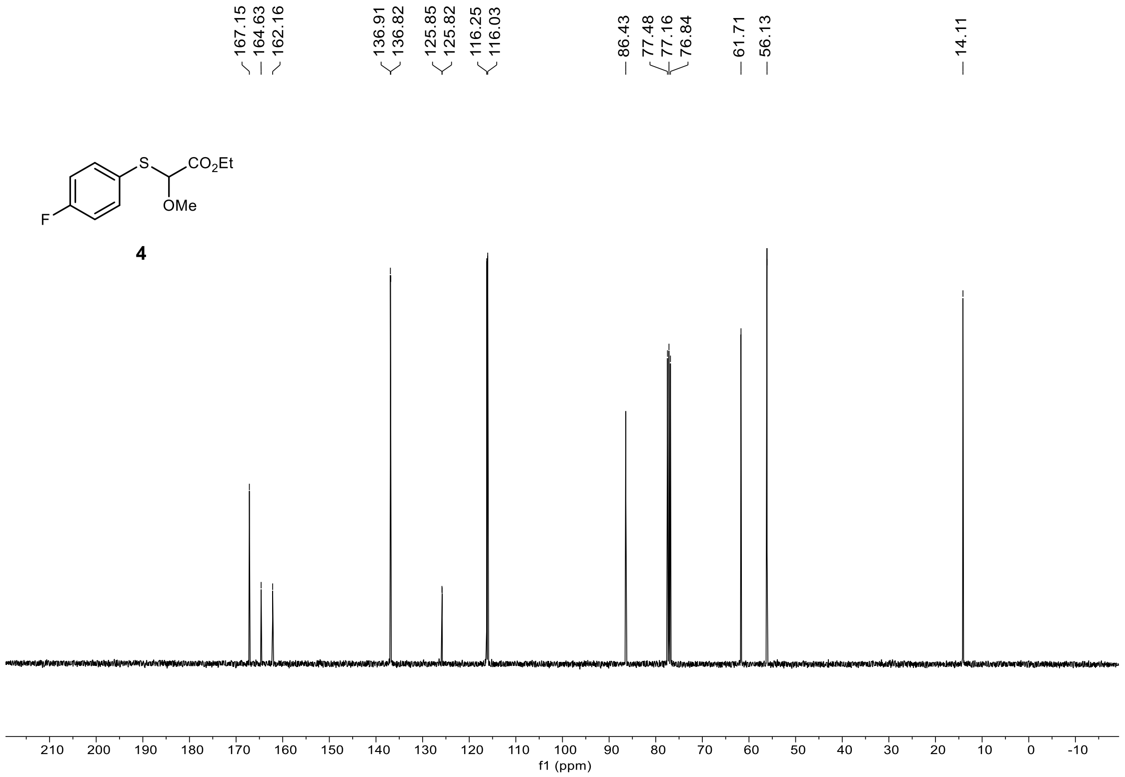

Supplementary Figure 16. ^{13}C NMR spectrum of compound **4** (101 MHz, r.t., $CDCl_3$)

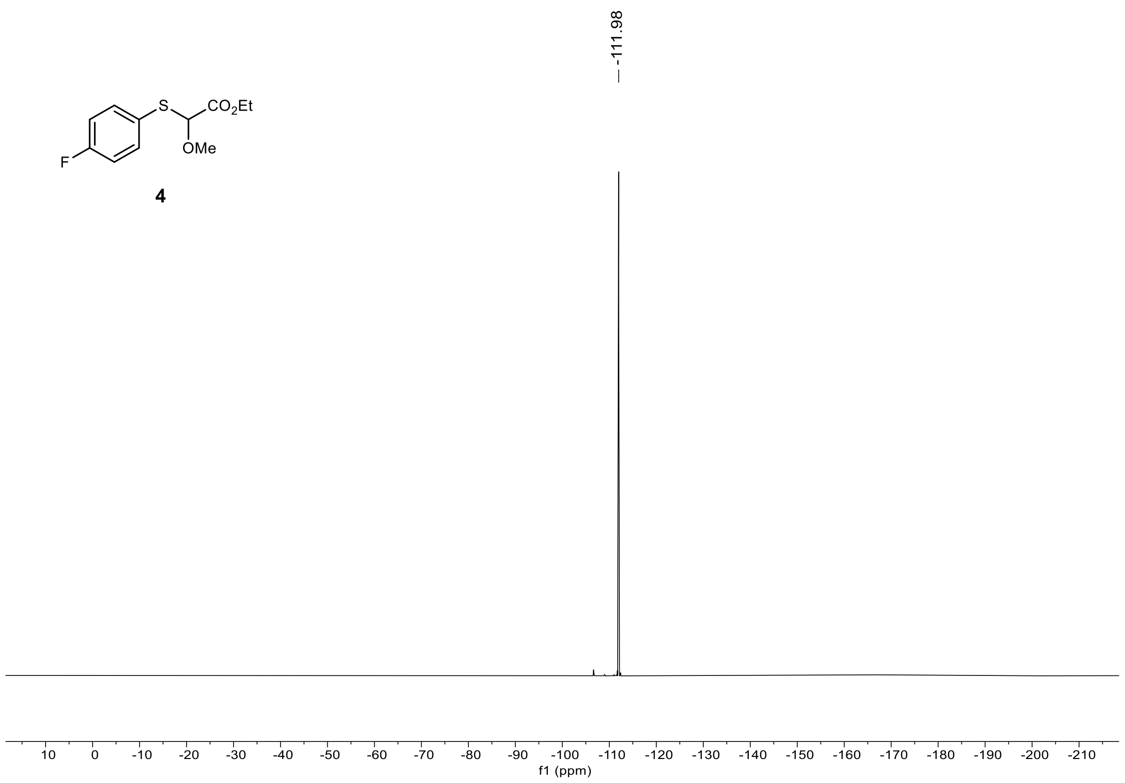

Supplementary Figure 17. **^{19}F NMR** spectrum of compound **4** (377 MHz, r.t., $CDCl_3$)

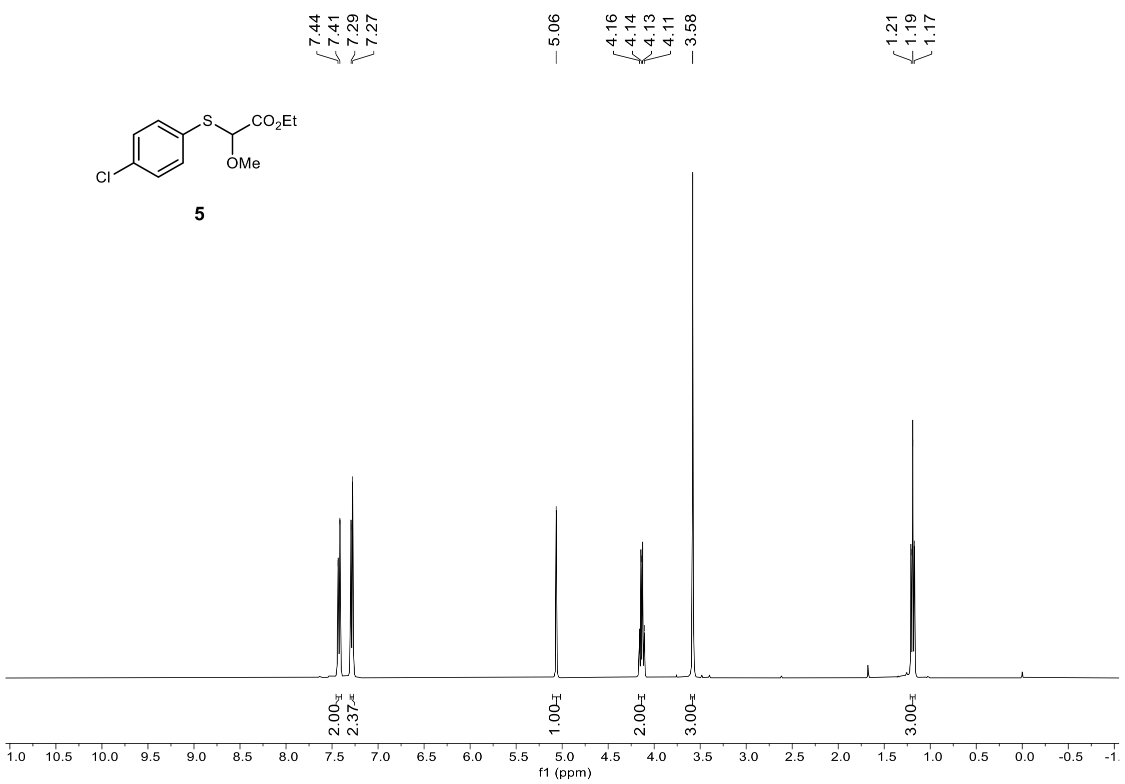

Supplementary Figure 18. ^{1}**H NMR** spectrum of compound **5** (400 MHz, r.t., $CDCl_3$)

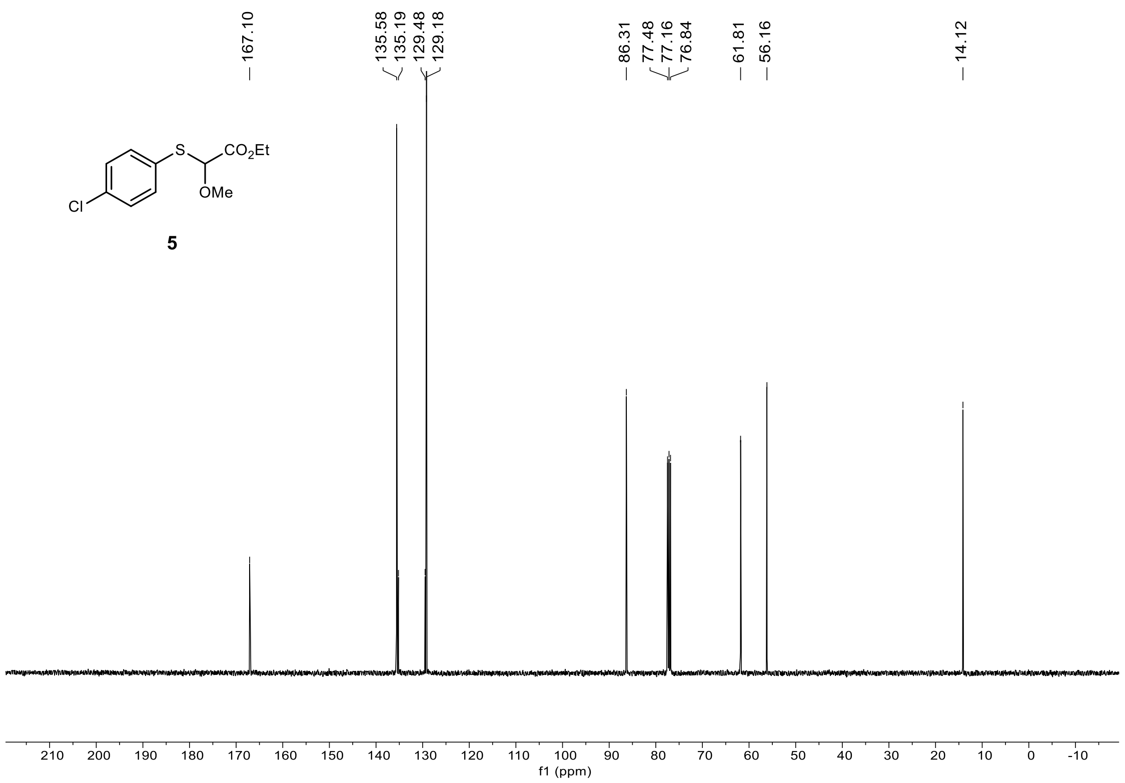

Supplementary Figure 19. ^{13}**C NMR** spectrum of compound **5** (101 MHz, r.t., $CDCl_3$)

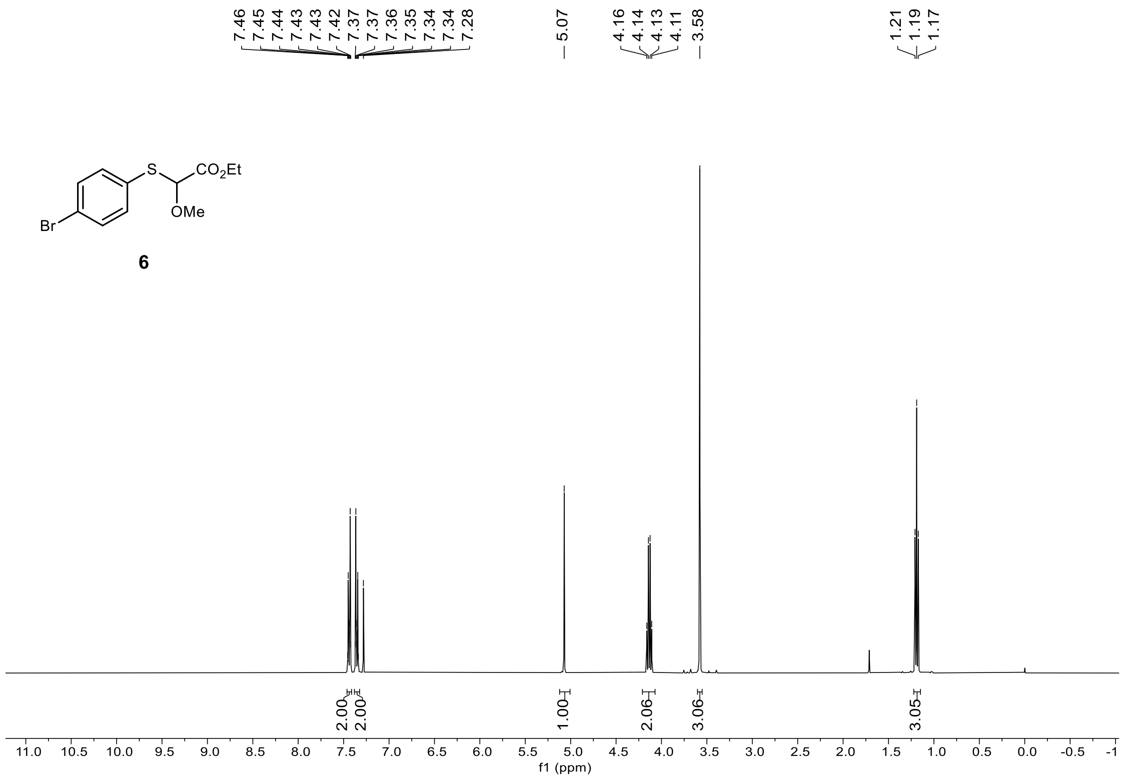

Supplementary Figure 20. ^{1}**H NMR** spectrum of compound **6** (400 MHz, r.t., $CDCl_3$)

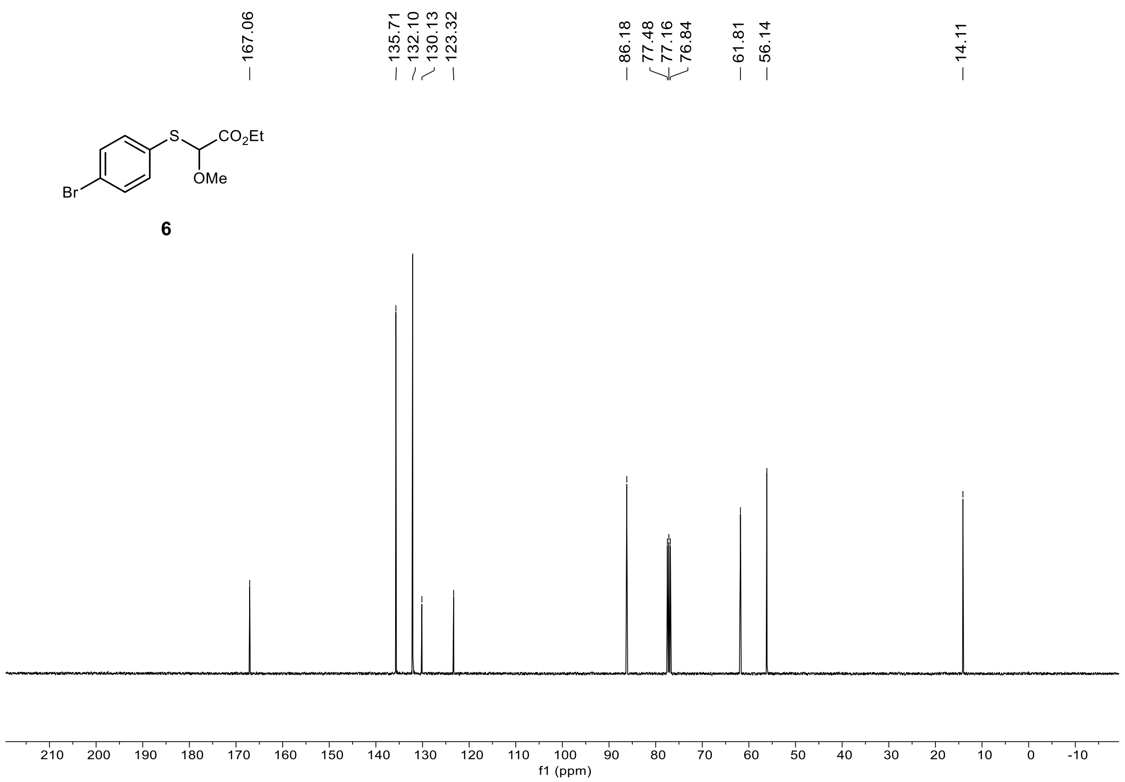

Supplementary Figure 21. ^{13}**C NMR** spectrum of compound **6** (101 MHz, r.t., $CDCl_3$)

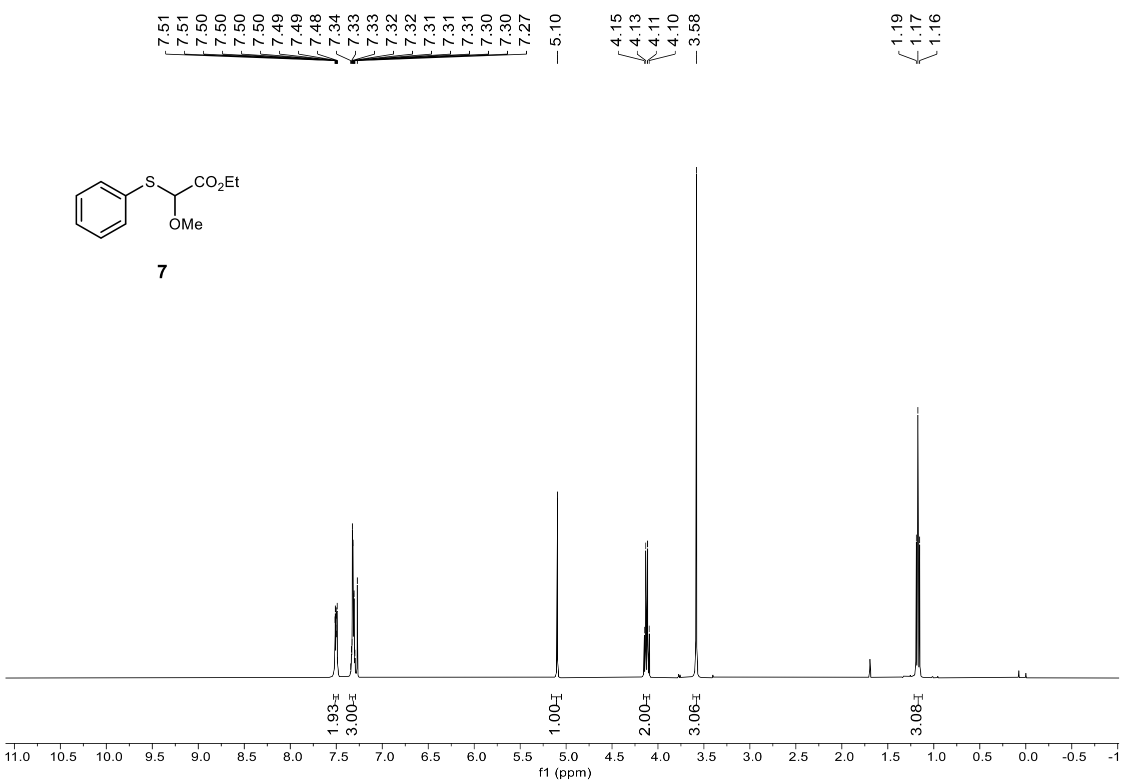

Supplementary Figure 22. ^{1}**H NMR** spectrum of compound **7** (400 MHz, r.t., $CDCl_3$)

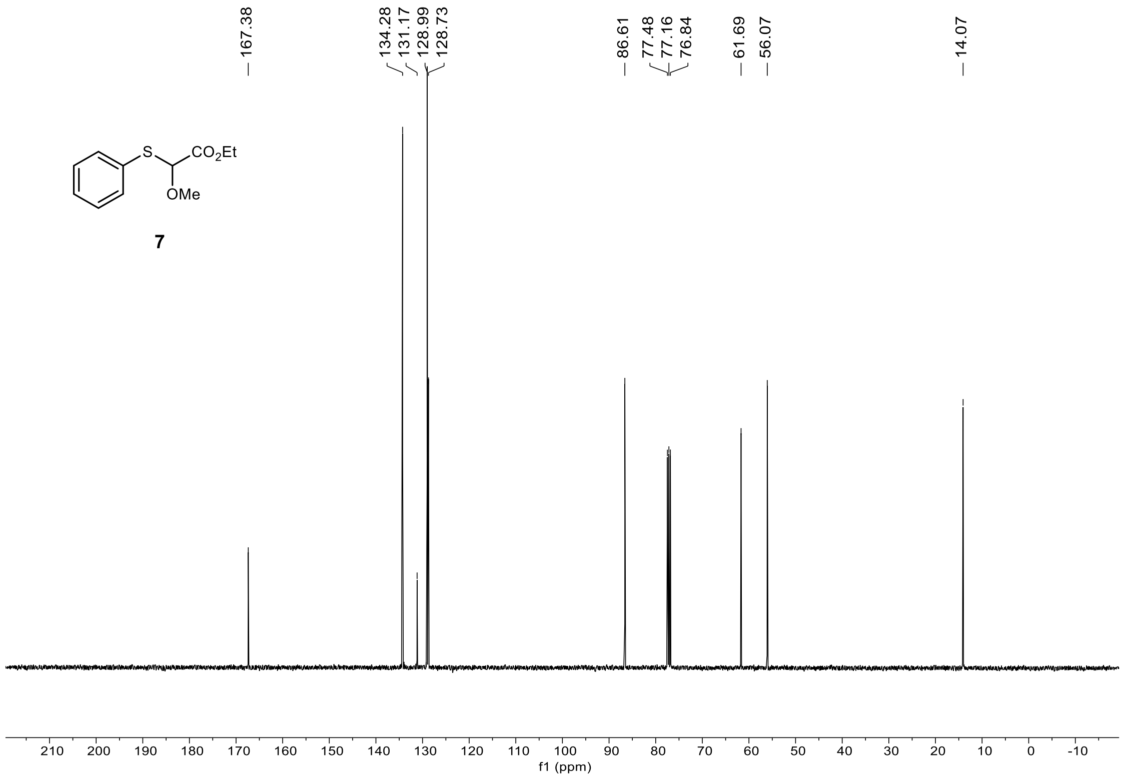

Supplementary Figure 23. ^{13}**C NMR** spectrum of compound **7** (101 MHz, r.t., $CDCl_3$)

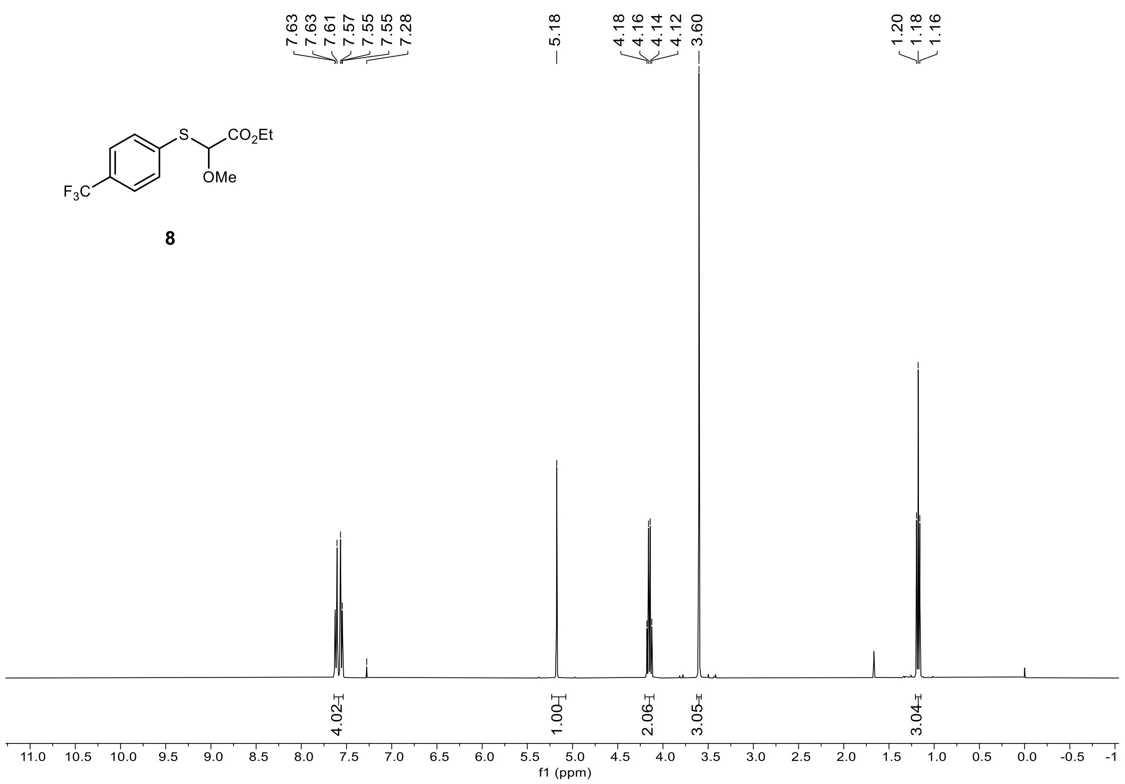

Supplementary Figure 24. ^{1}**H NMR** spectrum of compound **8** (400 MHz, r.t., $CDCl_3$)

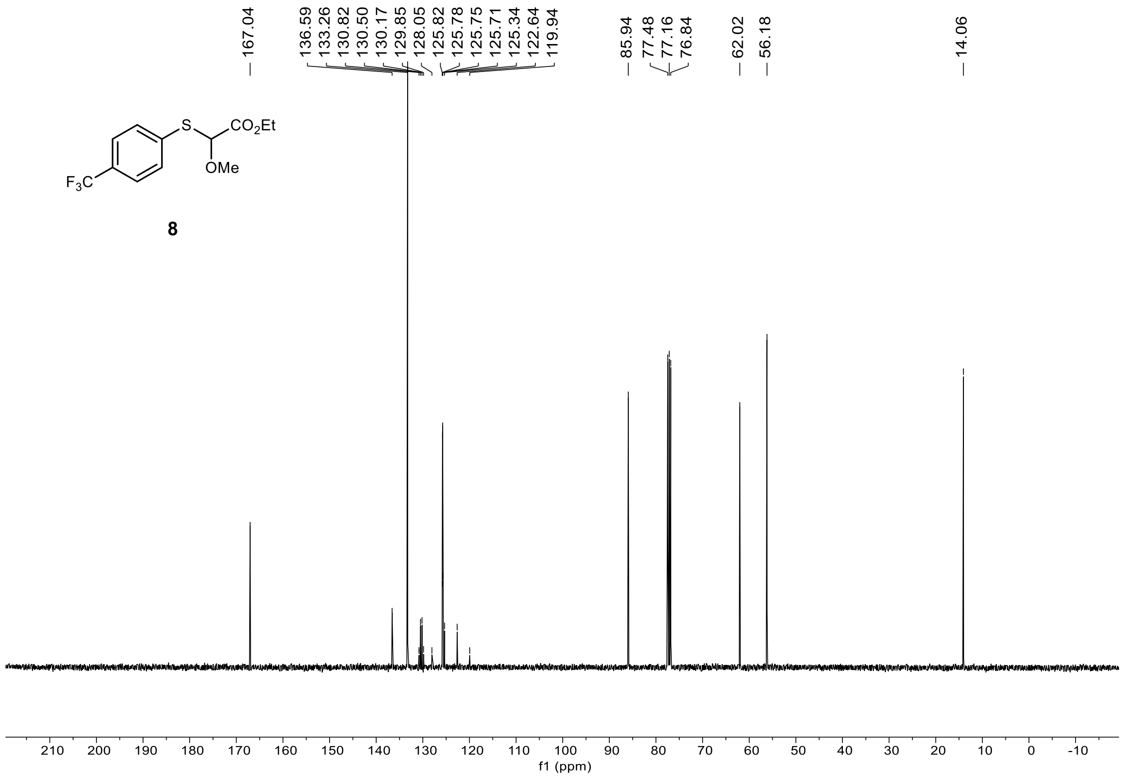

Supplementary Figure 25. ^{13}**C NMR** spectrum of compound **8** (101 MHz, r.t., $CDCl_3$)

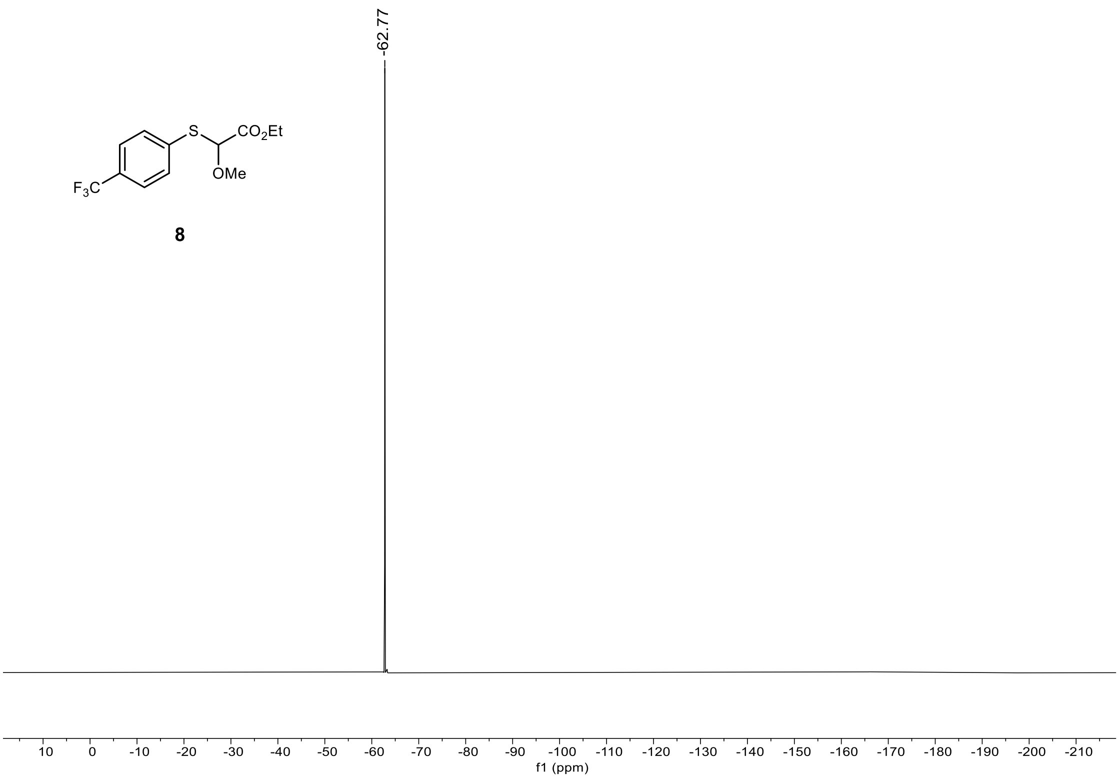

Supplementary Figure 26. ^{19}**F NMR** spectrum of compound **8** (377 MHz, r.t., $CDCl_3$)

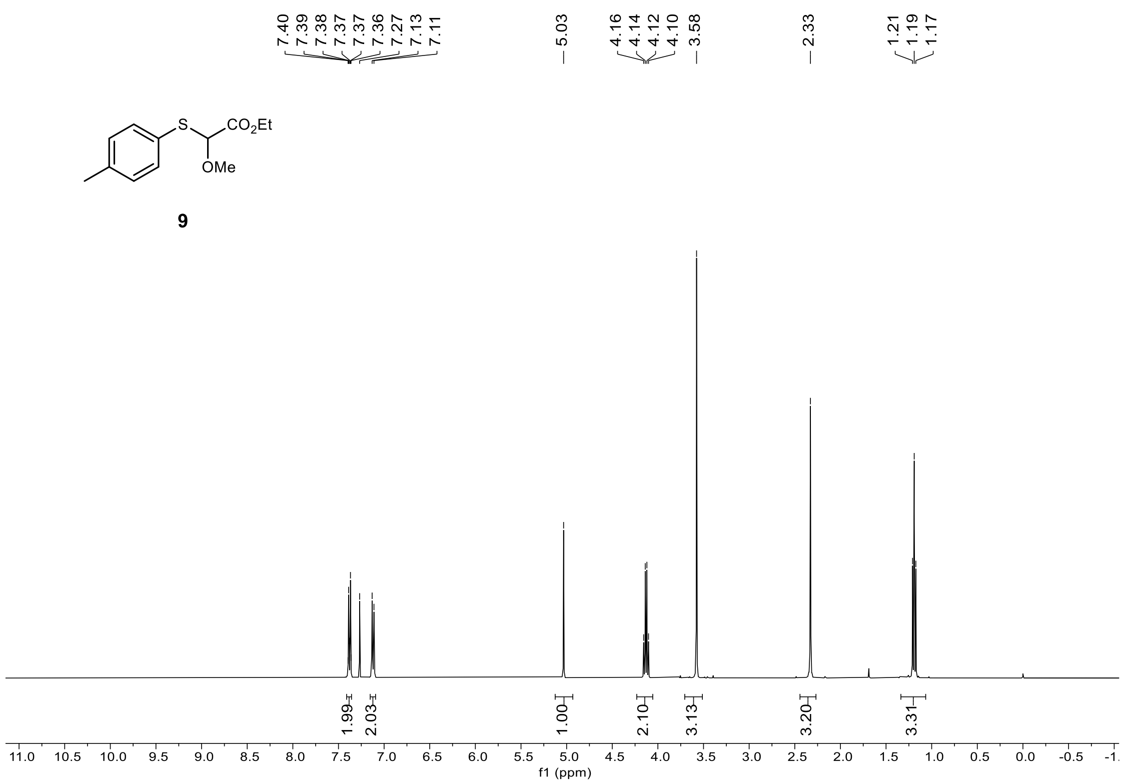

Supplementary Figure 27. ^{1}H NMR spectrum of compound **9** (400 MHz, r.t., $CDCl_3$)

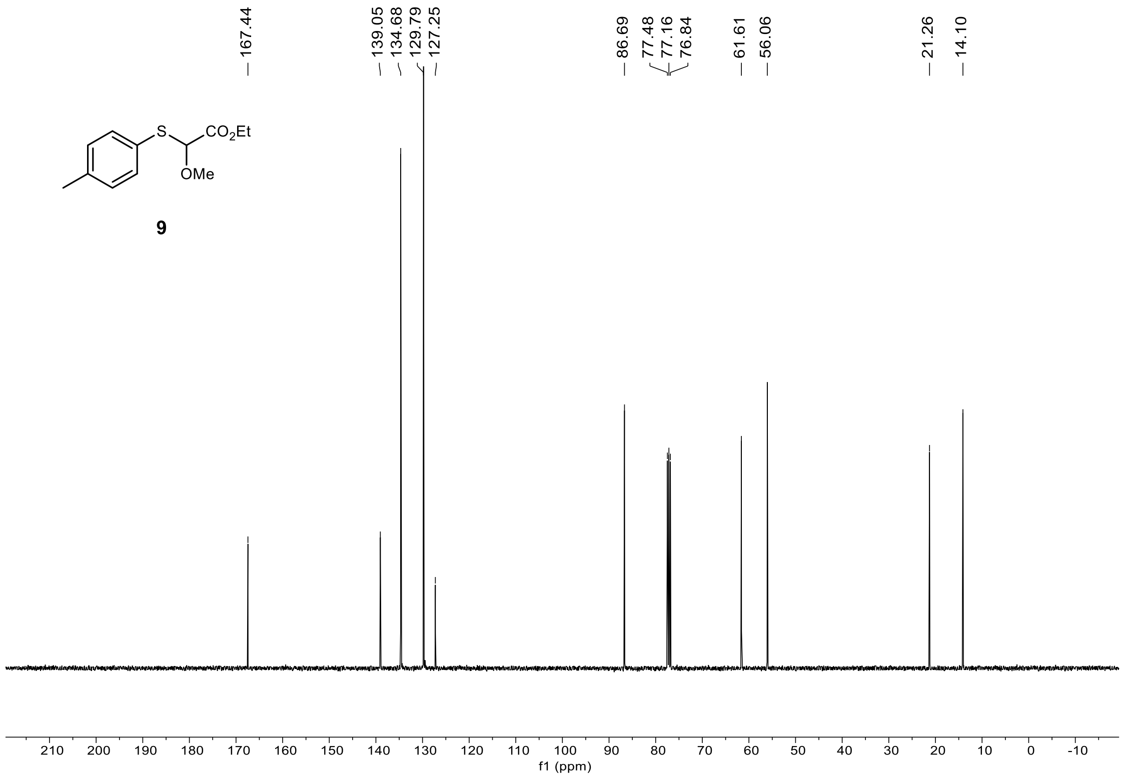

Supplementary Figure 28. ^{13}C NMR spectrum of compound **9** (101 MHz, r.t., $CDCl_3$)

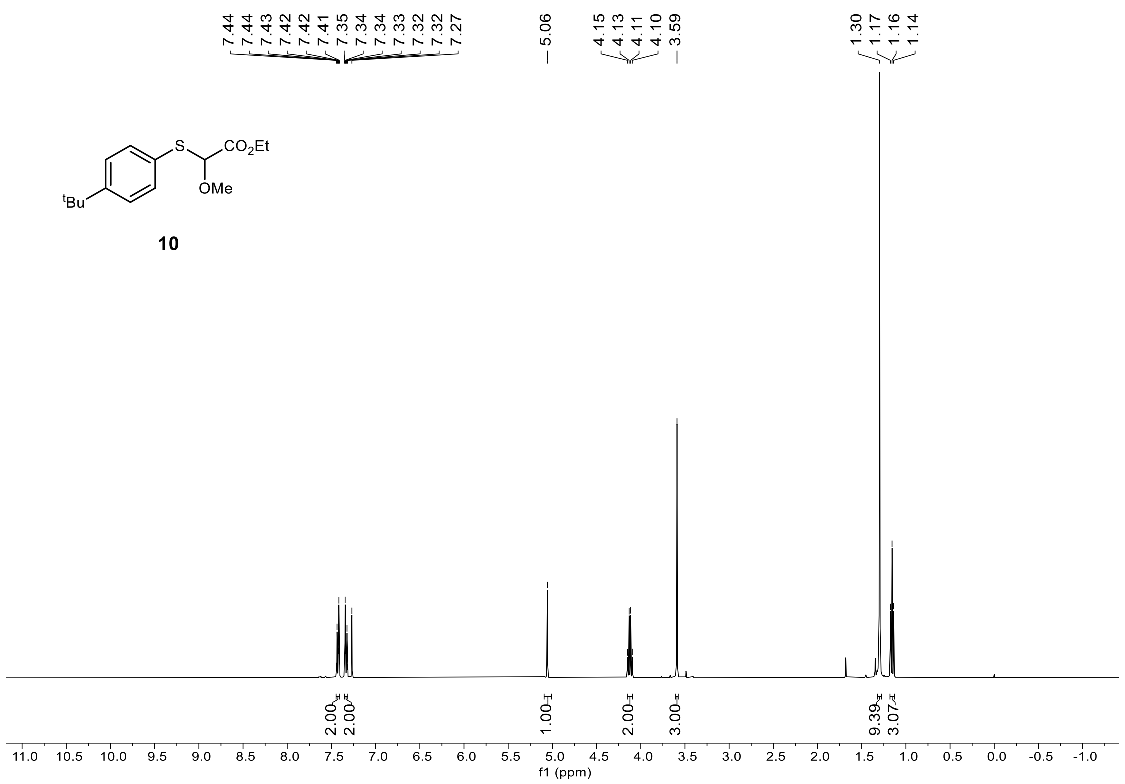

Supplementary Figure 29. ^{1}**H NMR** spectrum of compound **10** (400 MHz, r.t., $CDCl_3$)

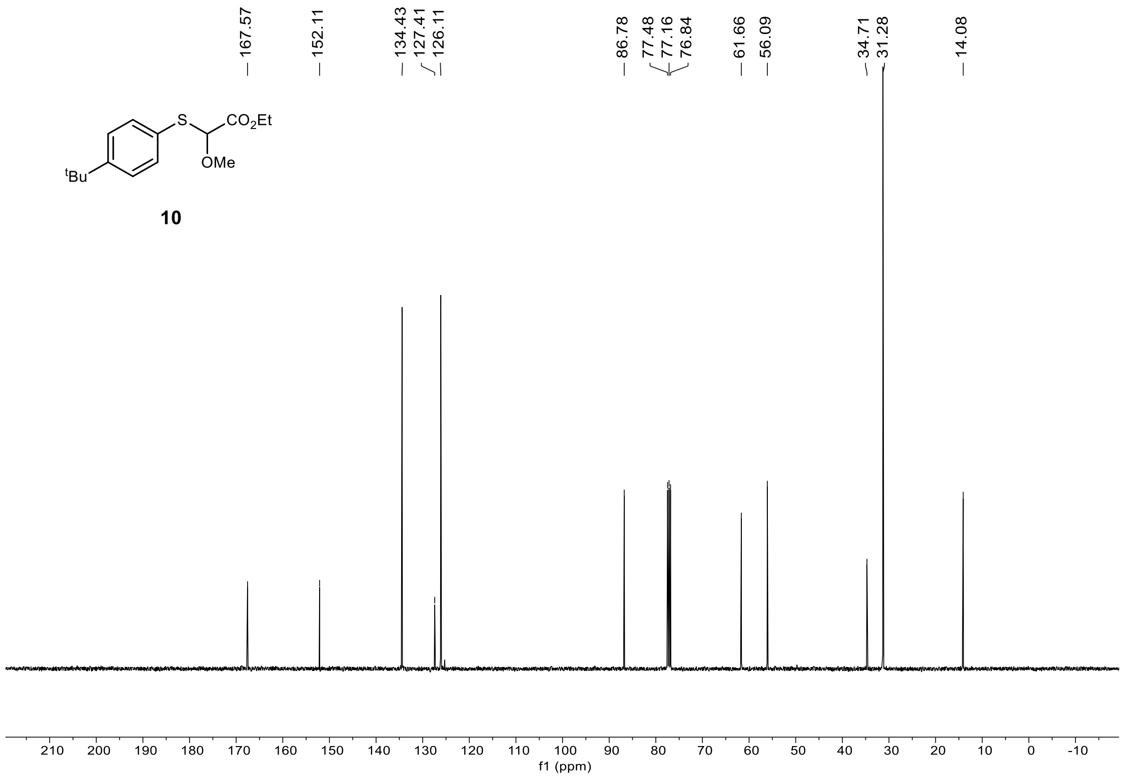

Supplementary Figure 30. ^{13}**C NMR** spectrum of compound **10** (101 MHz, r.t., $CDCl_3$)

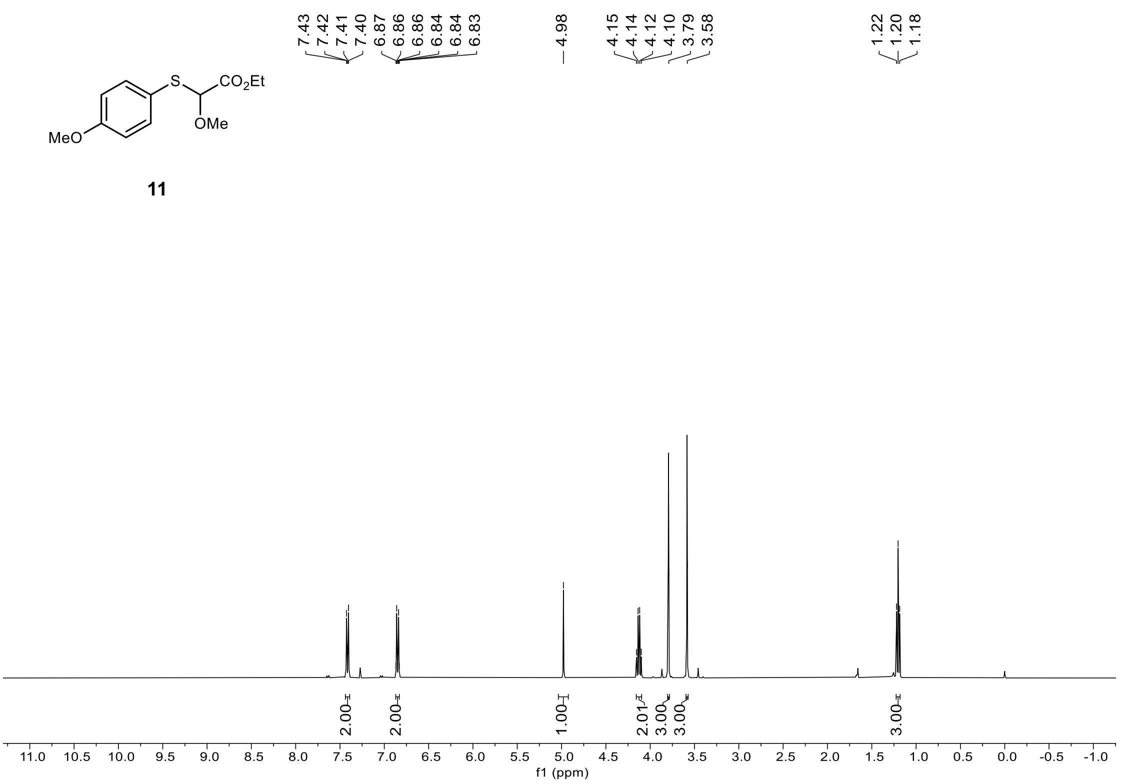

Supplementary Figure 31. ^{1}**H NMR** spectrum of compound **11** (400 MHz, r.t., $CDCl_3$)

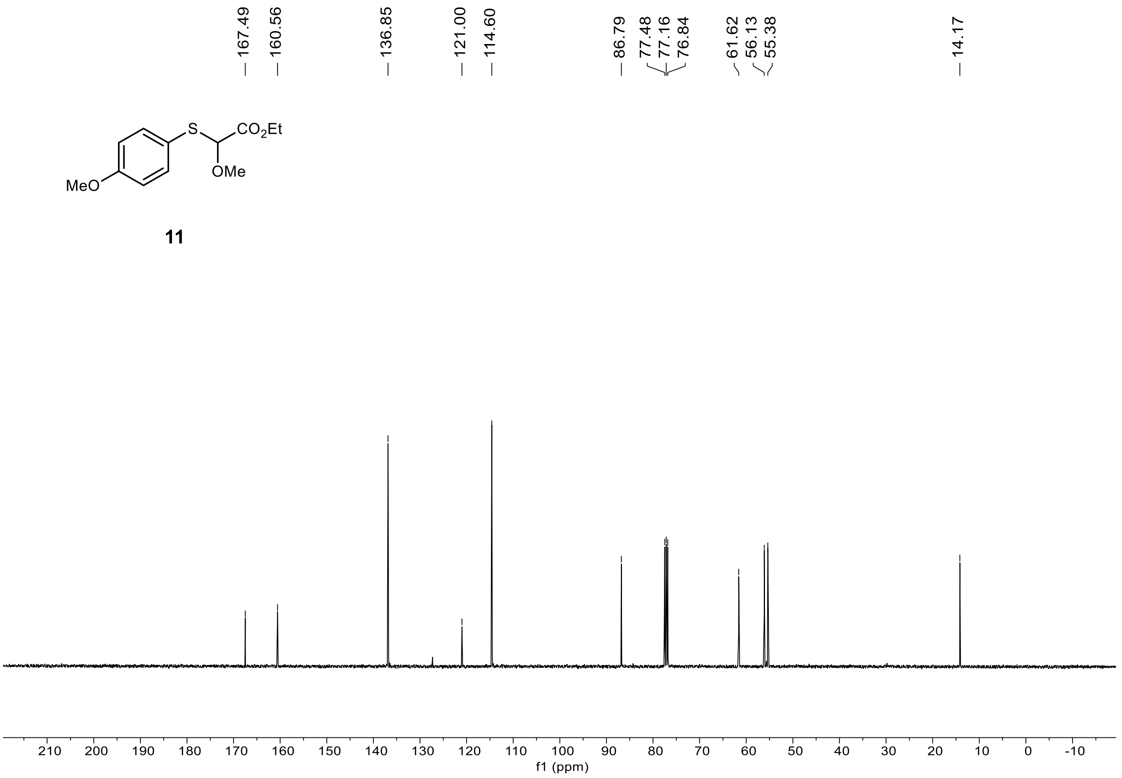

Supplementary Figure 32. ^{13}**C NMR** spectrum of compound **11** (101 MHz, r.t., $CDCl_3$)

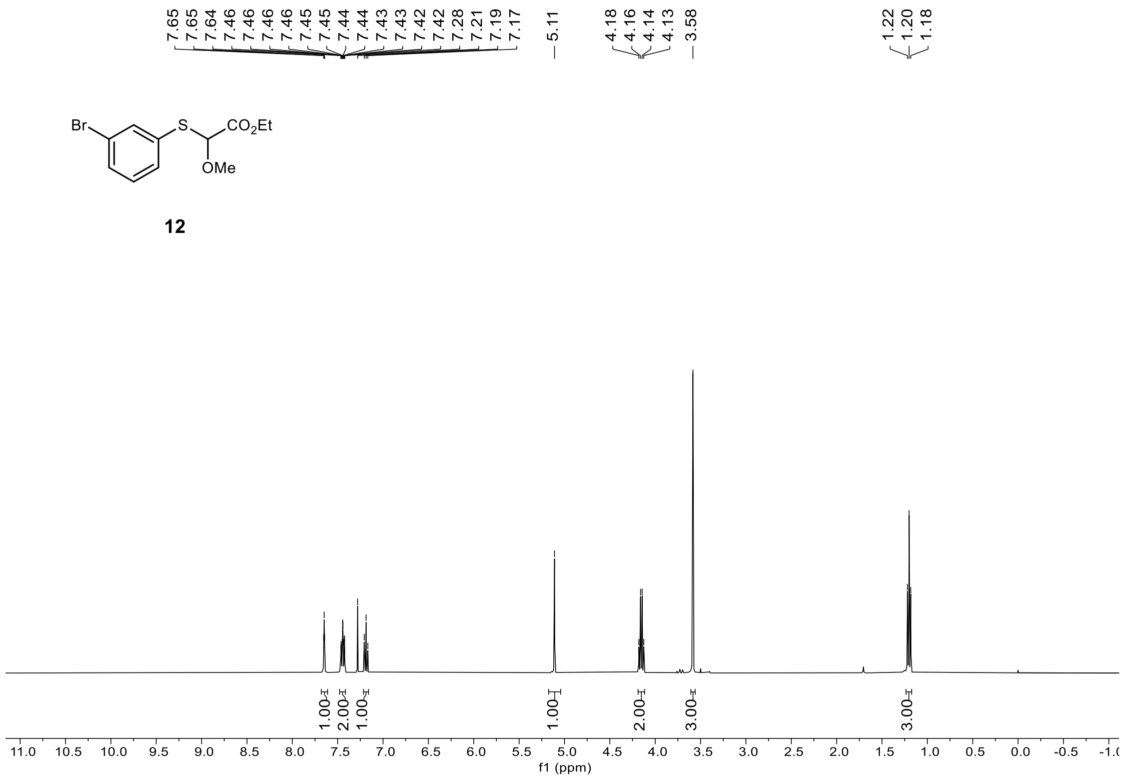

Supplementary Figure 33. ^{1}H NMR spectrum of compound **12** (400 MHz, r.t., $CDCl_3$)

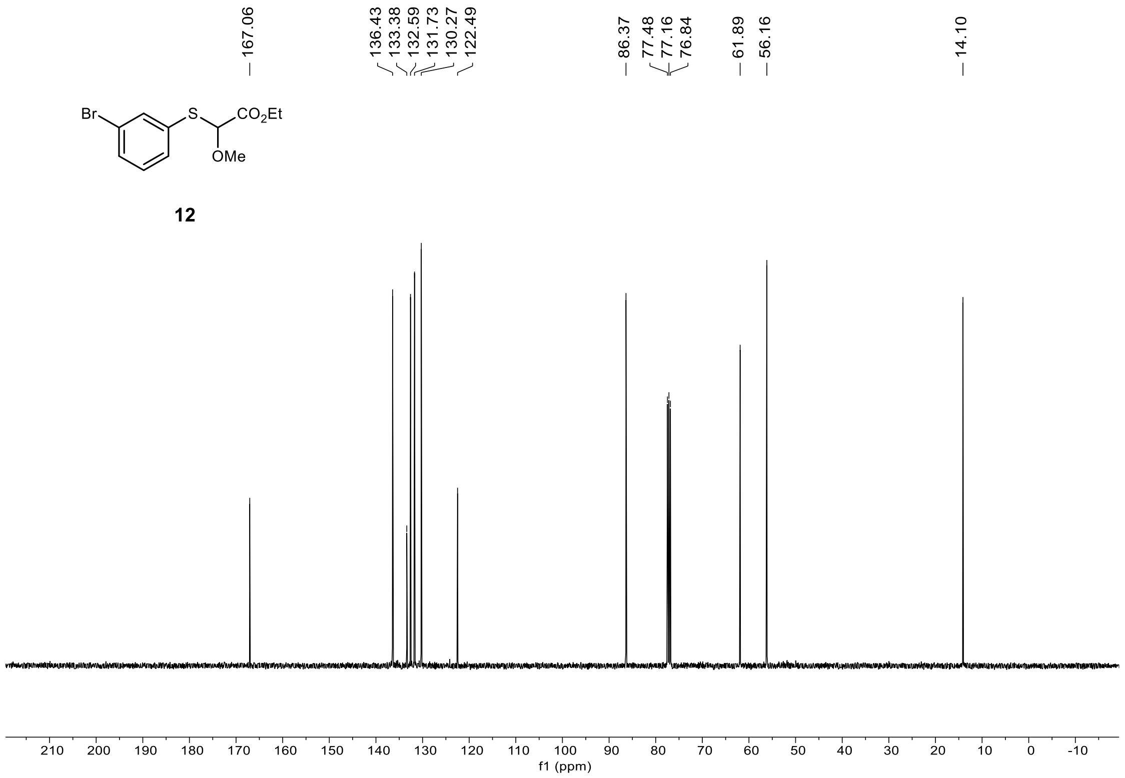

Supplementary Figure 34. ^{13}C NMR spectrum of compound **12** (101 MHz, r.t., $CDCl_3$)

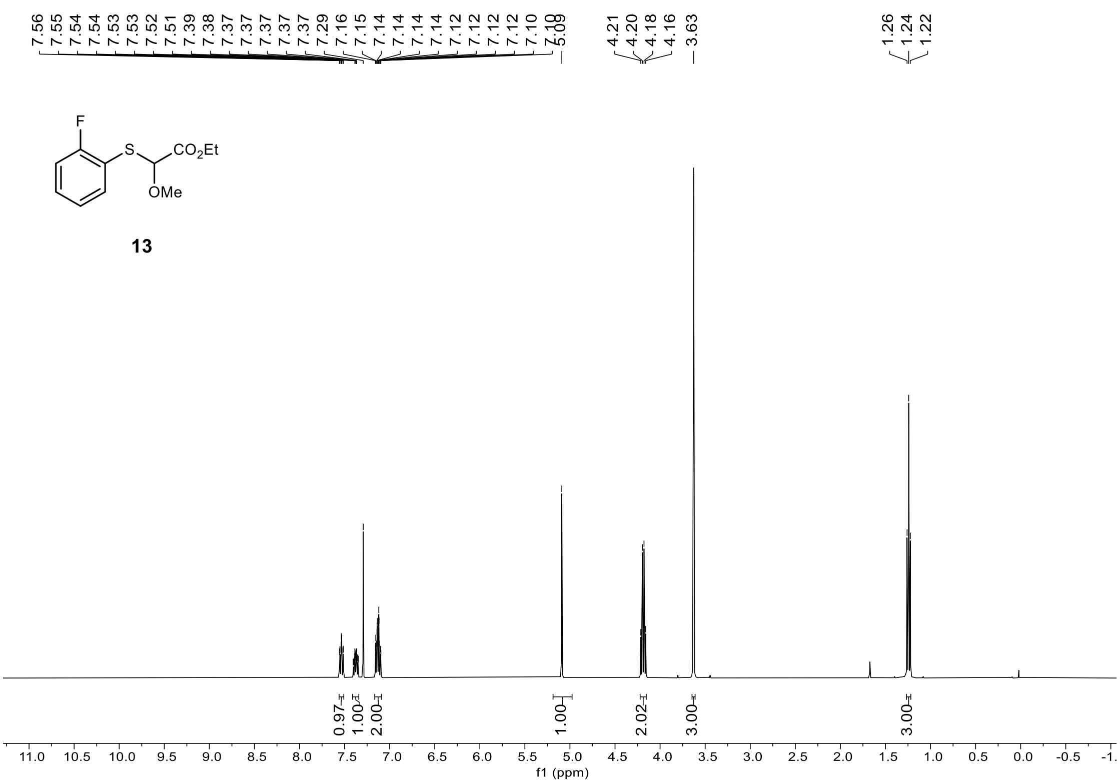

Supplementary Figure 35. ^{1}**H NMR** spectrum of compound **13** (400 MHz, r.t., $CDCl_3$)

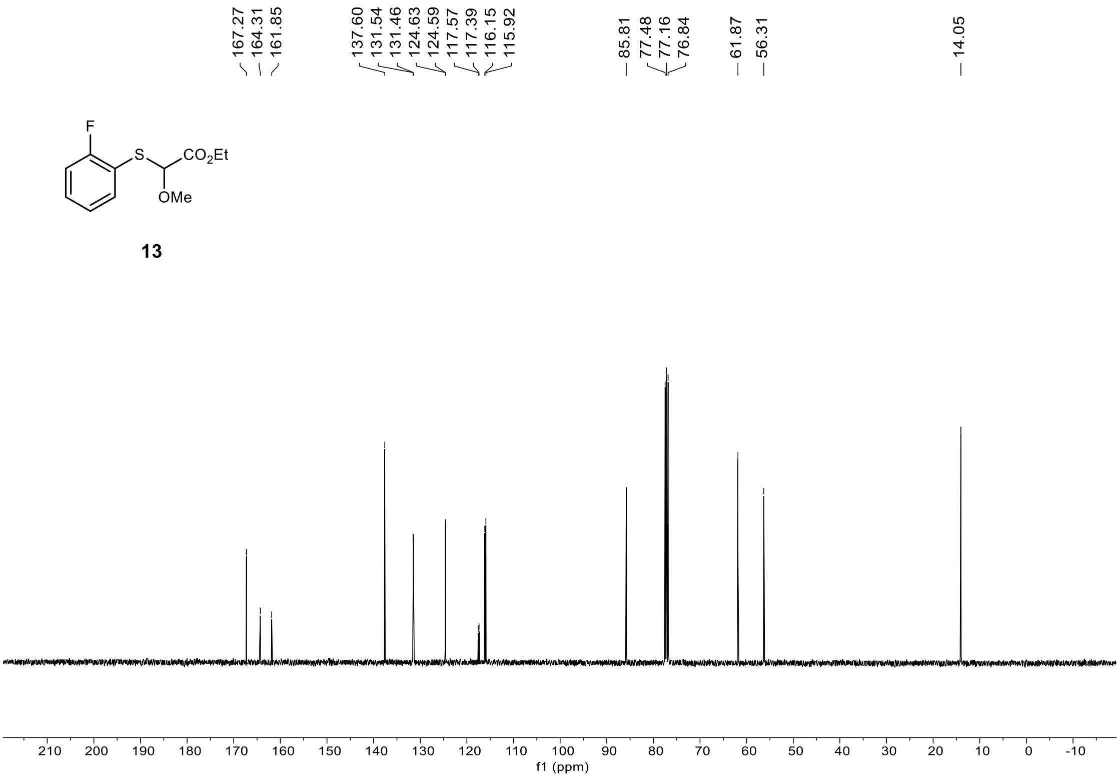

Supplementary Figure 36. ^{13}**C NMR** spectrum of compound **13** (101 MHz, r.t., $CDCl_3$)

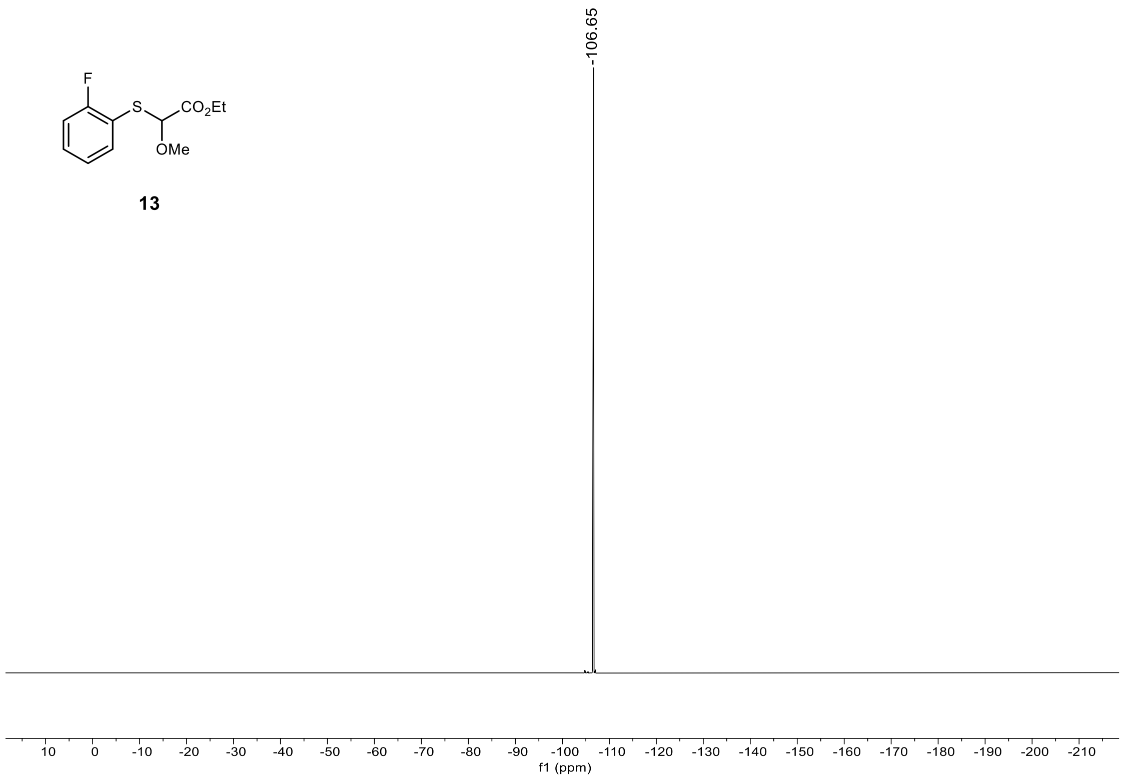

Supplementary Figure 37. ^{19}F NMR spectrum of compound **13** (377 MHz, r.t., $CDCl_3$)

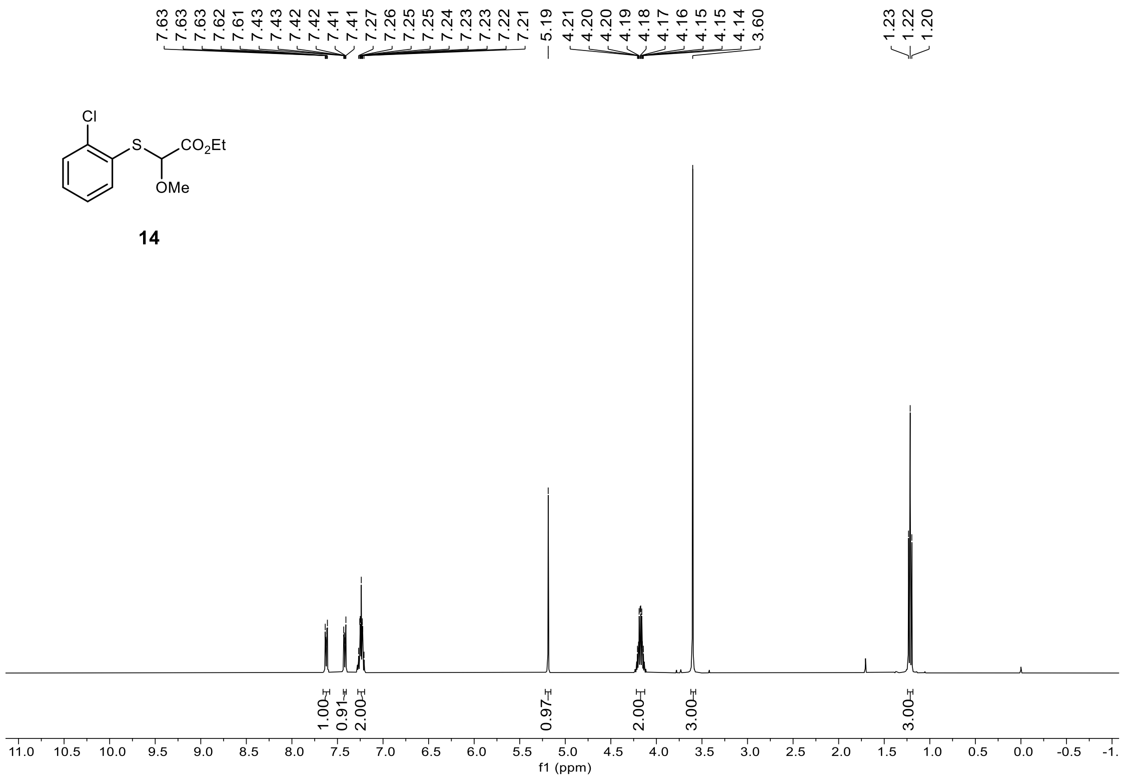

Supplementary Figure 38. ^{1}**H NMR** spectrum of compound **14** (400 MHz, r.t., $CDCl_3$)

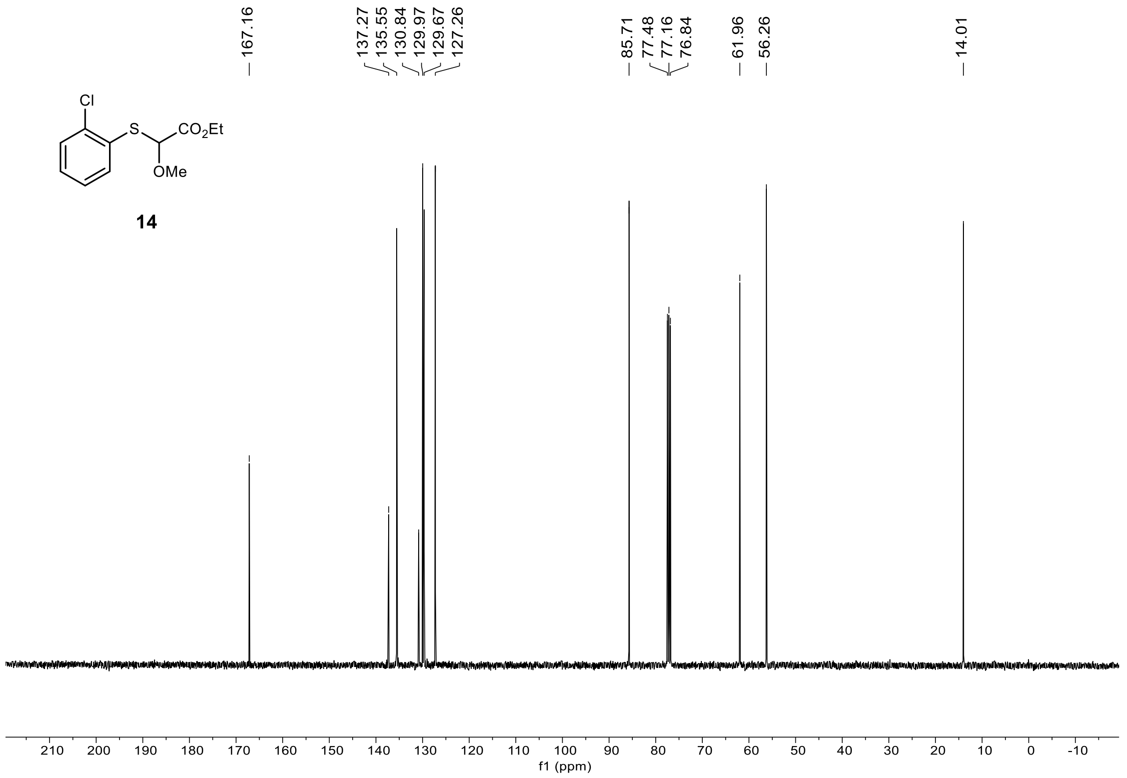

Supplementary Figure 39. ^{13}**C NMR** spectrum of compound **14** (101 MHz, r.t., $CDCl_3$)

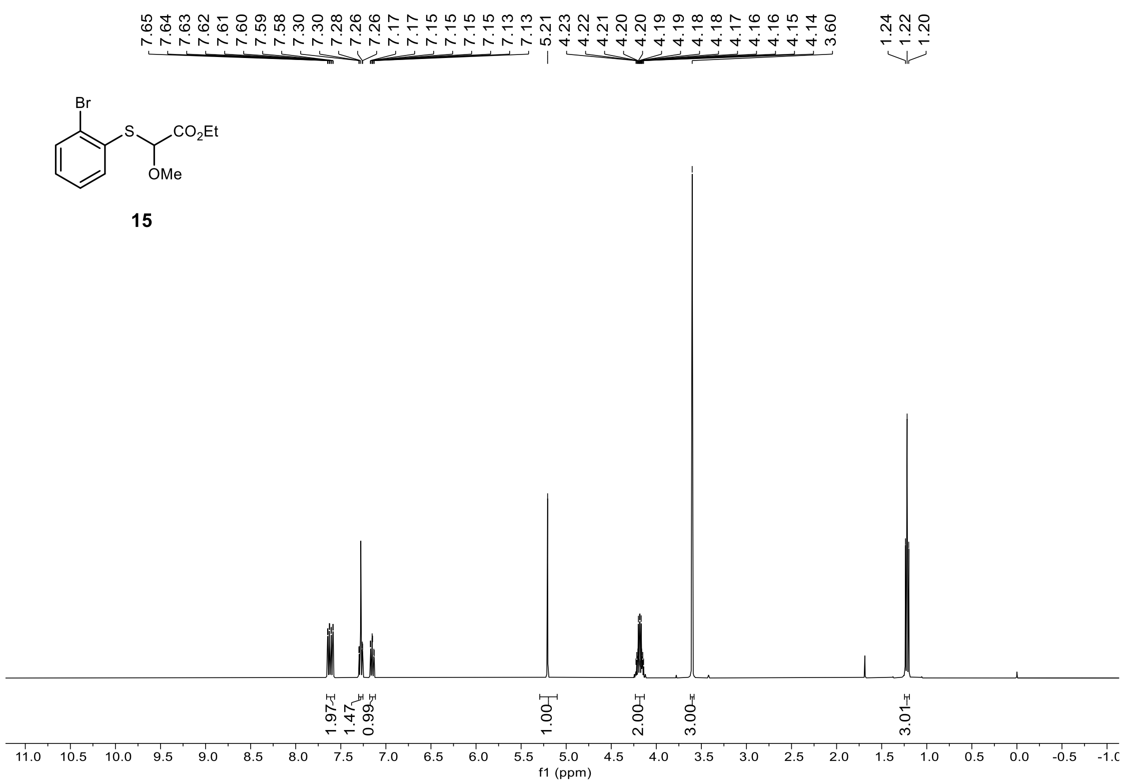

Supplementary Figure 40. ^{1}**H NMR** spectrum of compound **15** (400 MHz, r.t., $CDCl_3$)

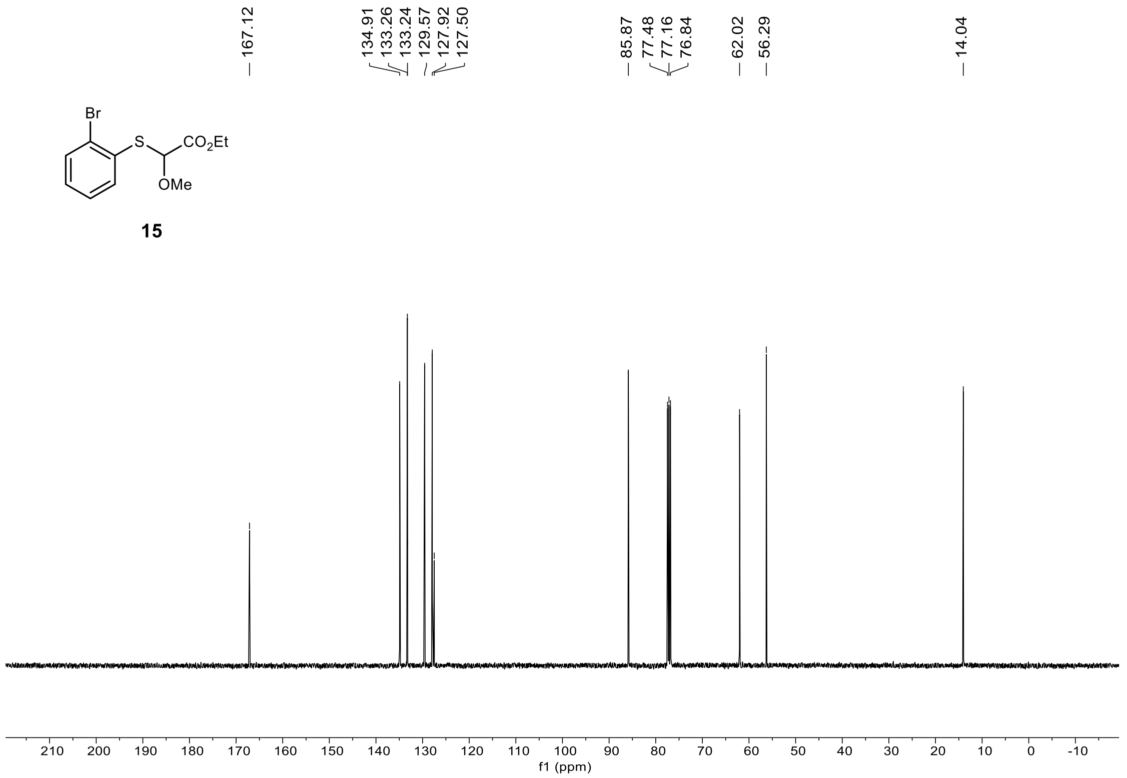

Supplementary Figure 41. ^{13}**C NMR** spectrum of compound **15** (101 MHz, r.t., $CDCl_3$)

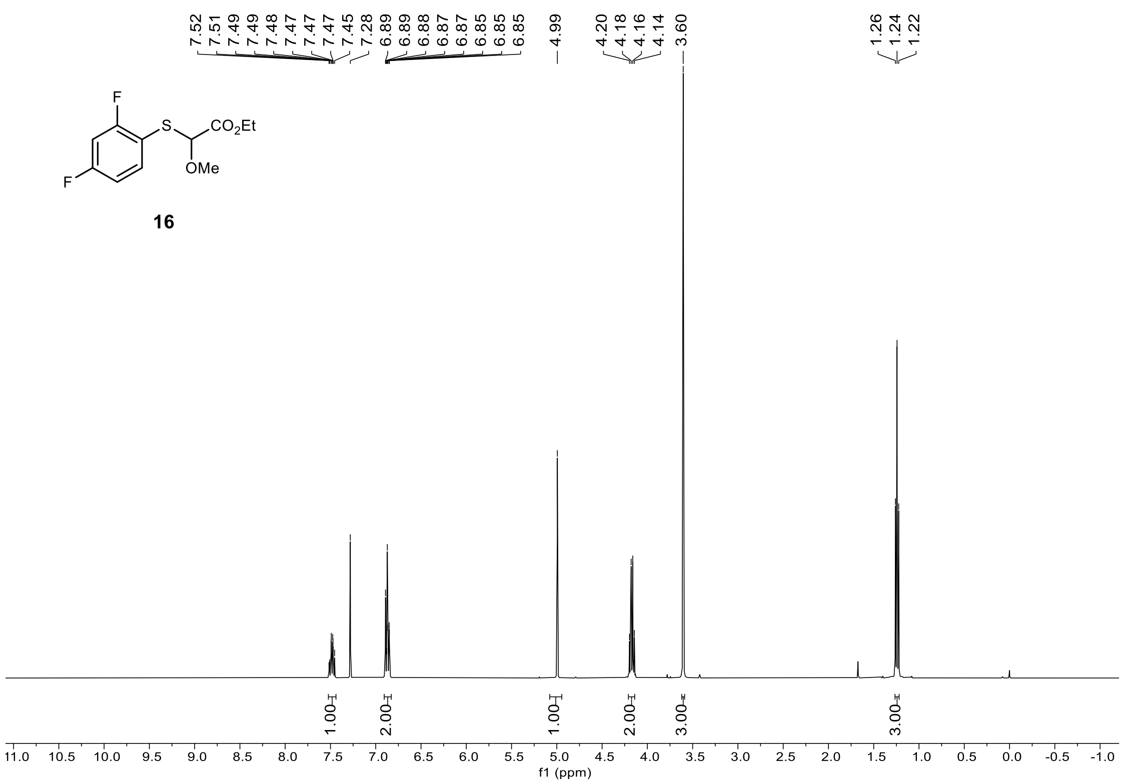

Supplementary Figure 42. **^{1}H NMR** spectrum of compound **16** (400 MHz, r.t., $CDCl_3$)

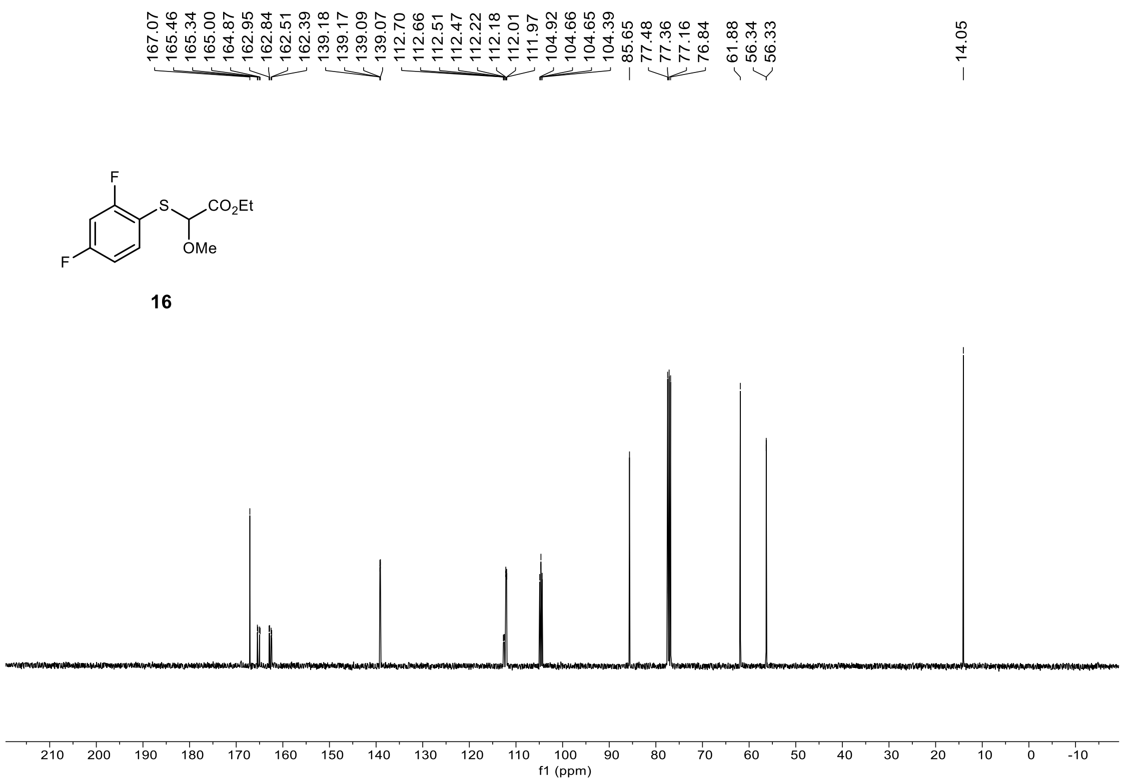

Supplementary Figure 43. **^{13}C NMR** spectrum of compound **16** (101 MHz, r.t., $CDCl_3$)

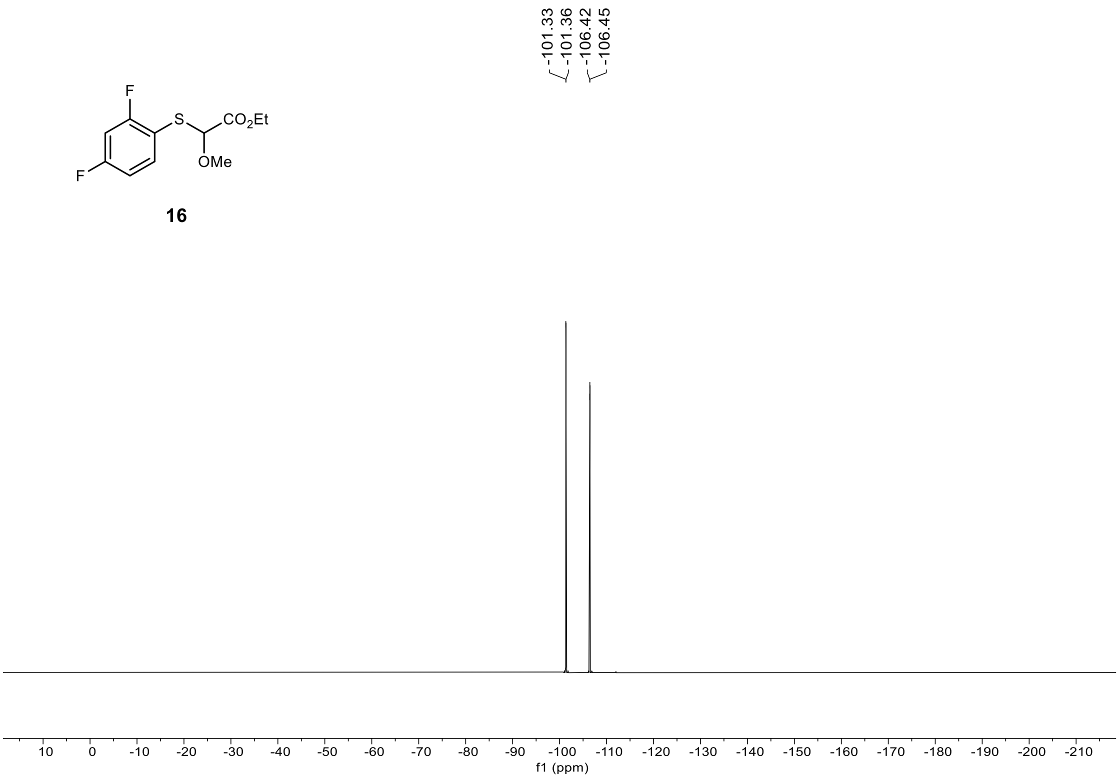

Supplementary Figure 44. ^{19}**F NMR** spectrum of compound **16** (377 MHz, r.t., $CDCl_3$)

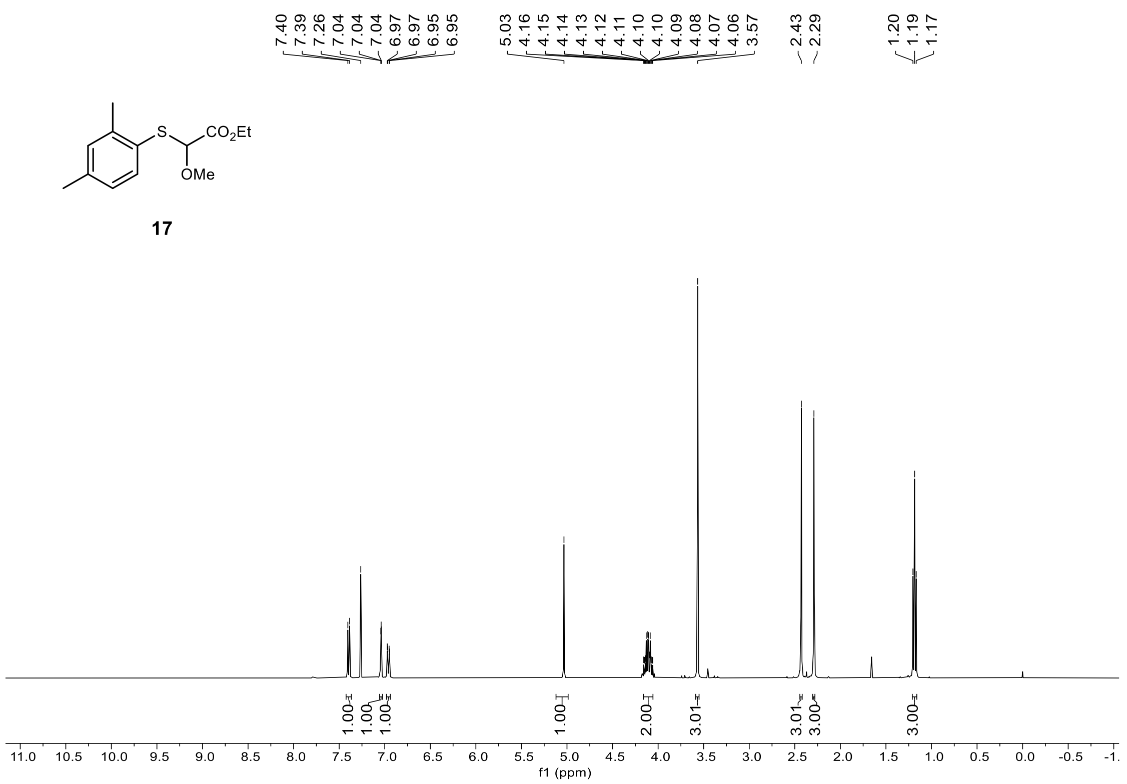

Supplementary Figure 45. ^{1}**H NMR** spectrum of compound **17** (400 MHz, r.t., $CDCl_3$)

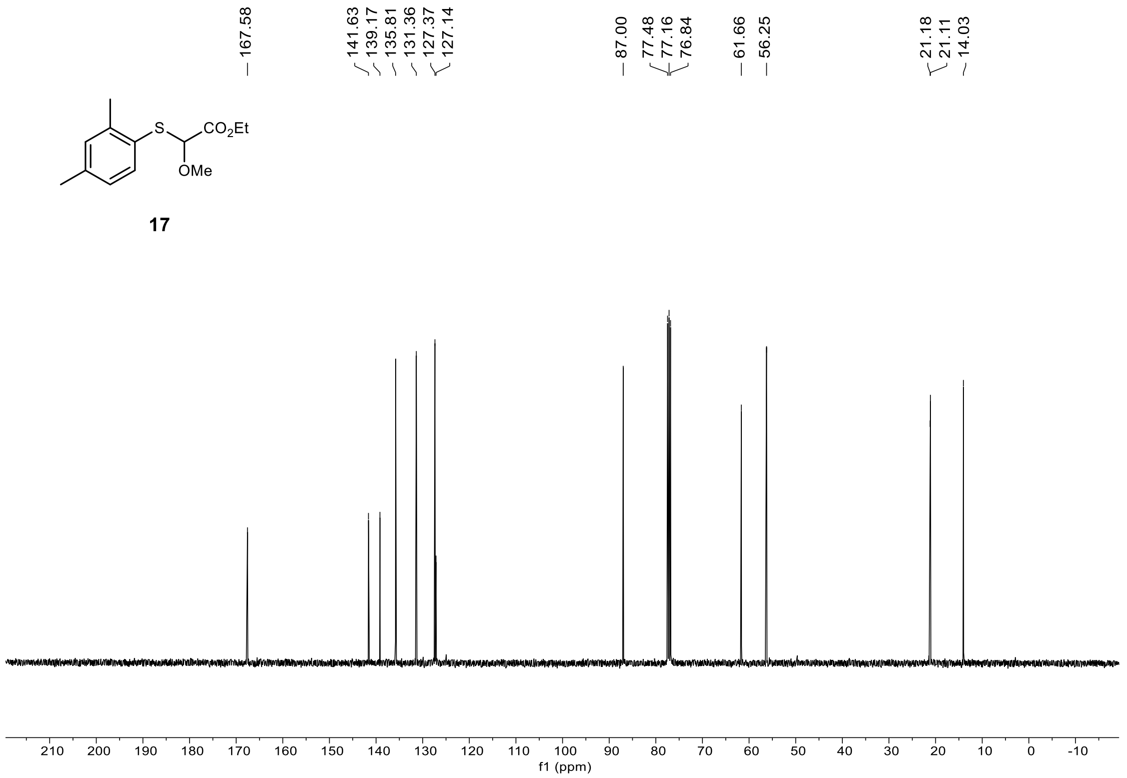

Supplementary Figure 46. ^{13}**C NMR** spectrum of compound **17** (101 MHz, r.t., $CDCl_3$)

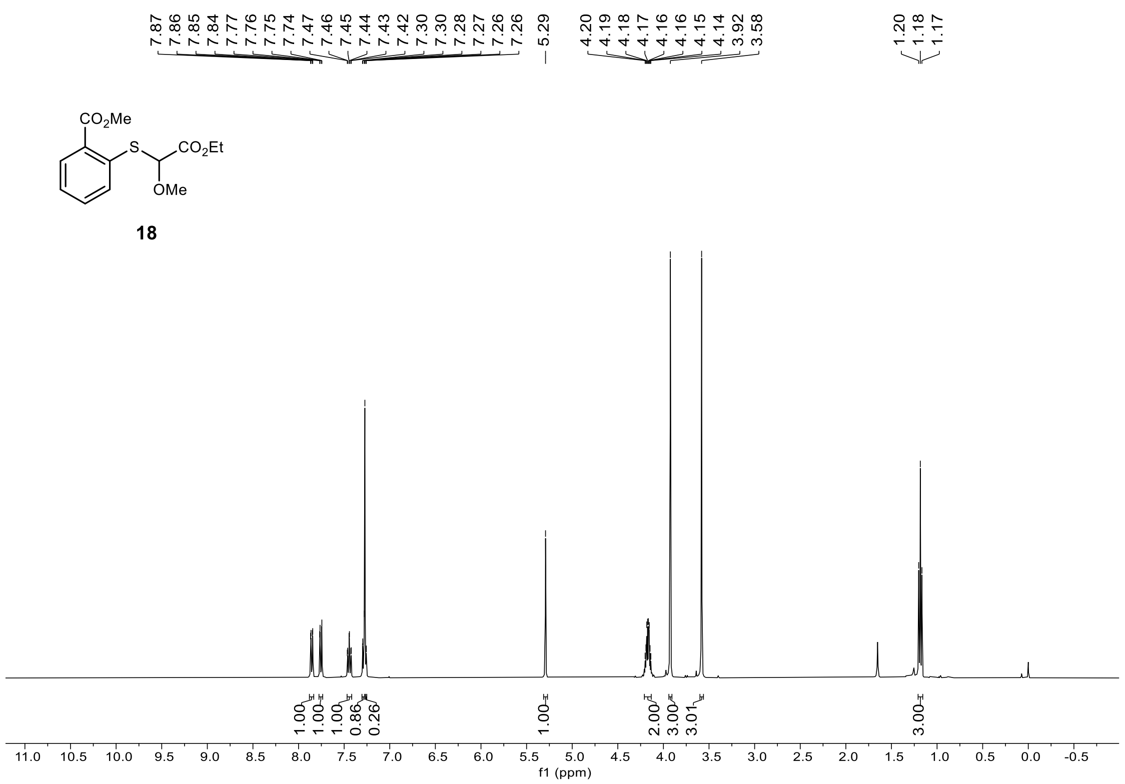

Supplementary Figure 47. ^{1}**H NMR** spectrum of compound **18** (400 MHz, r.t., $CDCl_3$)

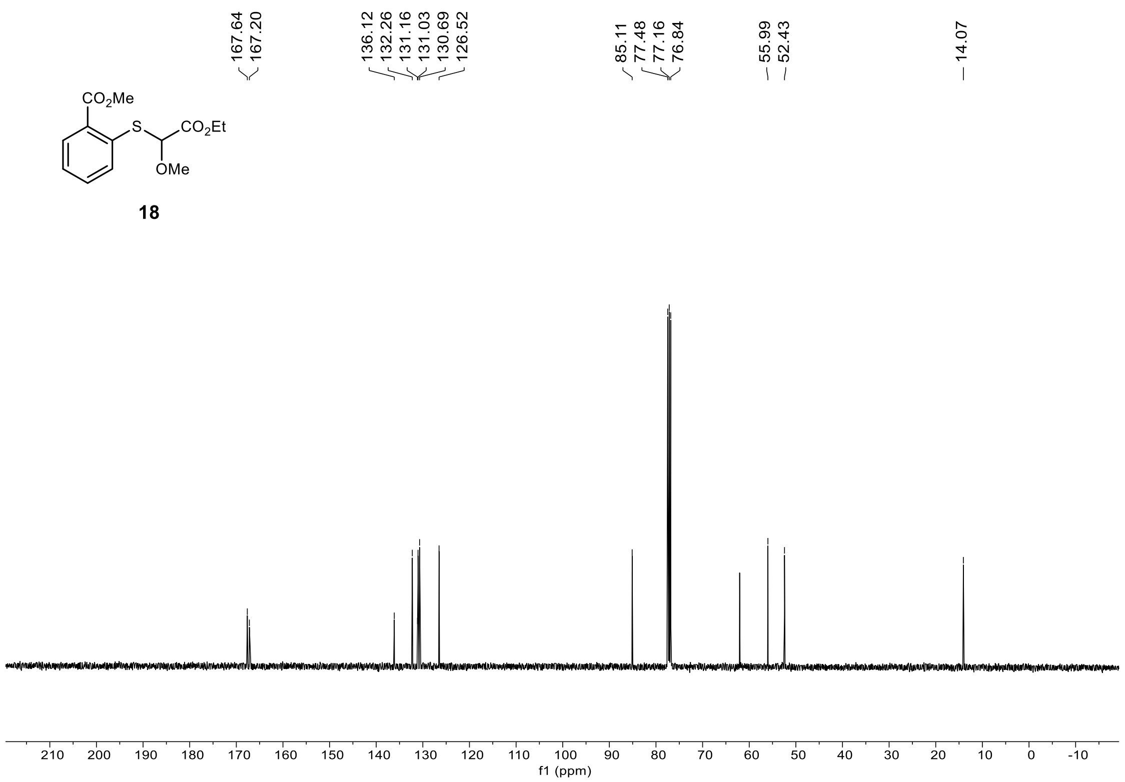

Supplementary Figure 48. ^{13}**C NMR** spectrum of compound **18** (101 MHz, r.t., $CDCl_3$)

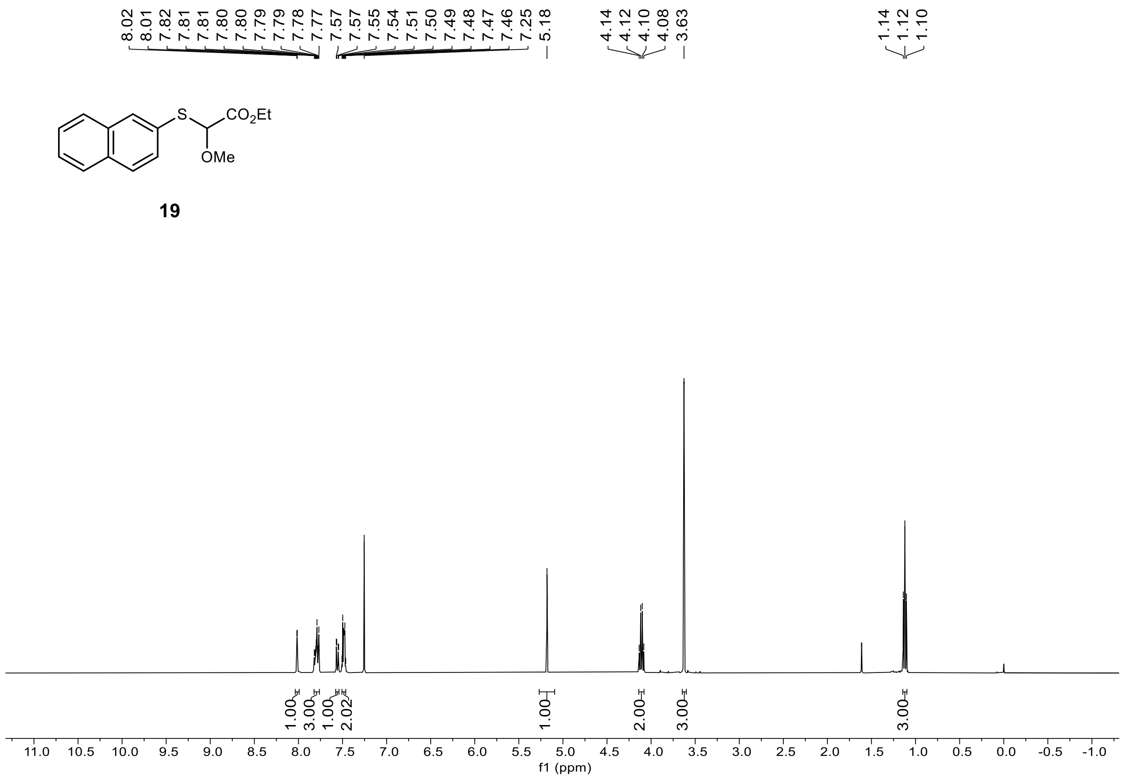

Supplementary Figure 49. ^{1}**H NMR** spectrum of compound **19** (400 MHz, r.t., $CDCl_3$)

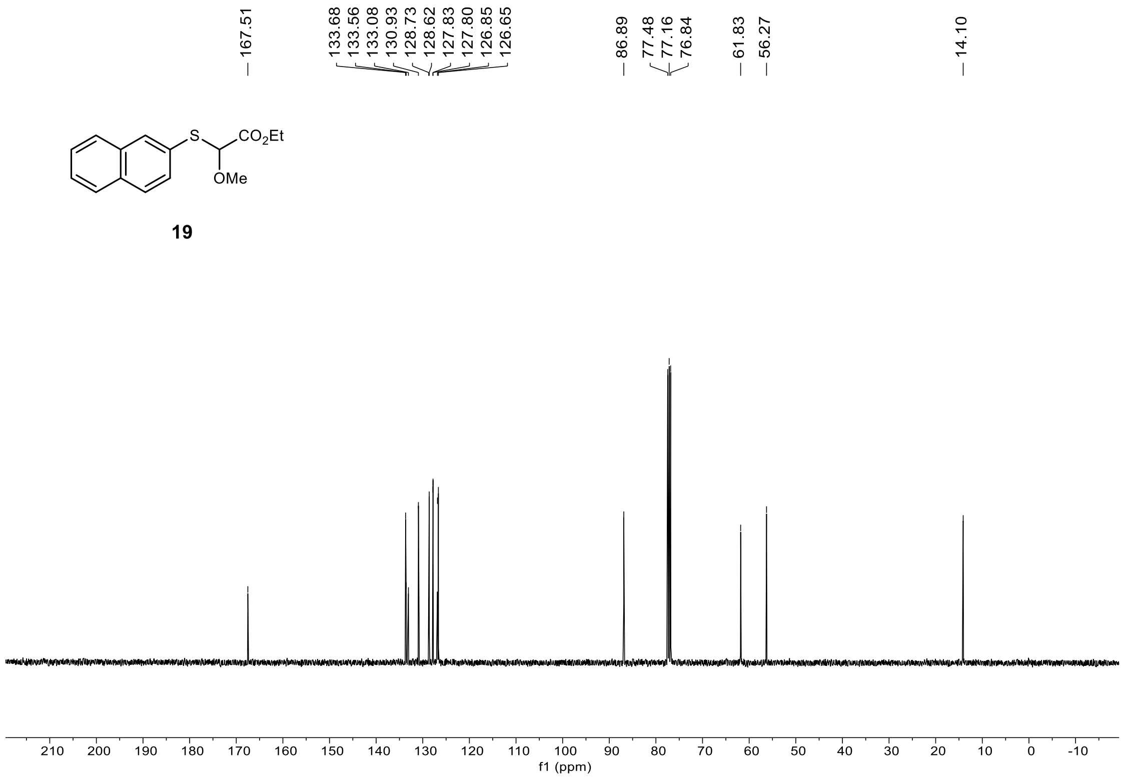

Supplementary Figure 50. ^{13}**C NMR** spectrum of compound **19** (101 MHz, r.t., $CDCl_3$)

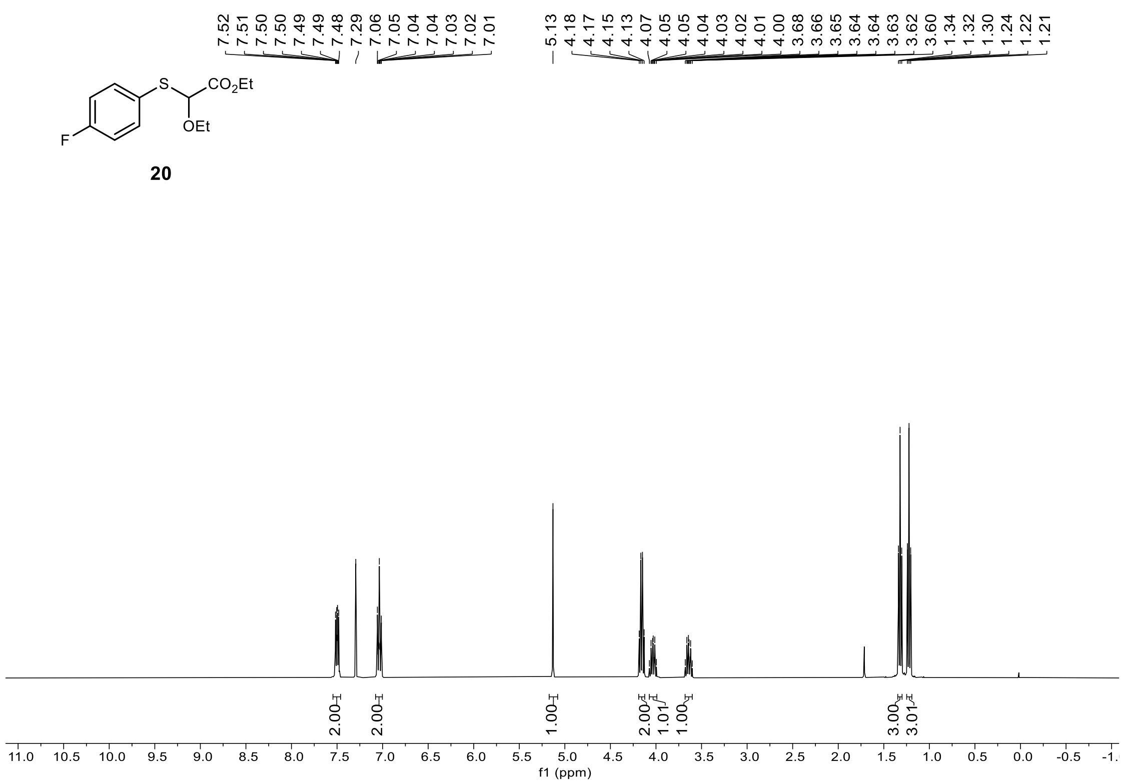

Supplementary Figure 51. ^{1}**H NMR** spectrum of compound **20** (400 MHz, r.t., $CDCl_3$)

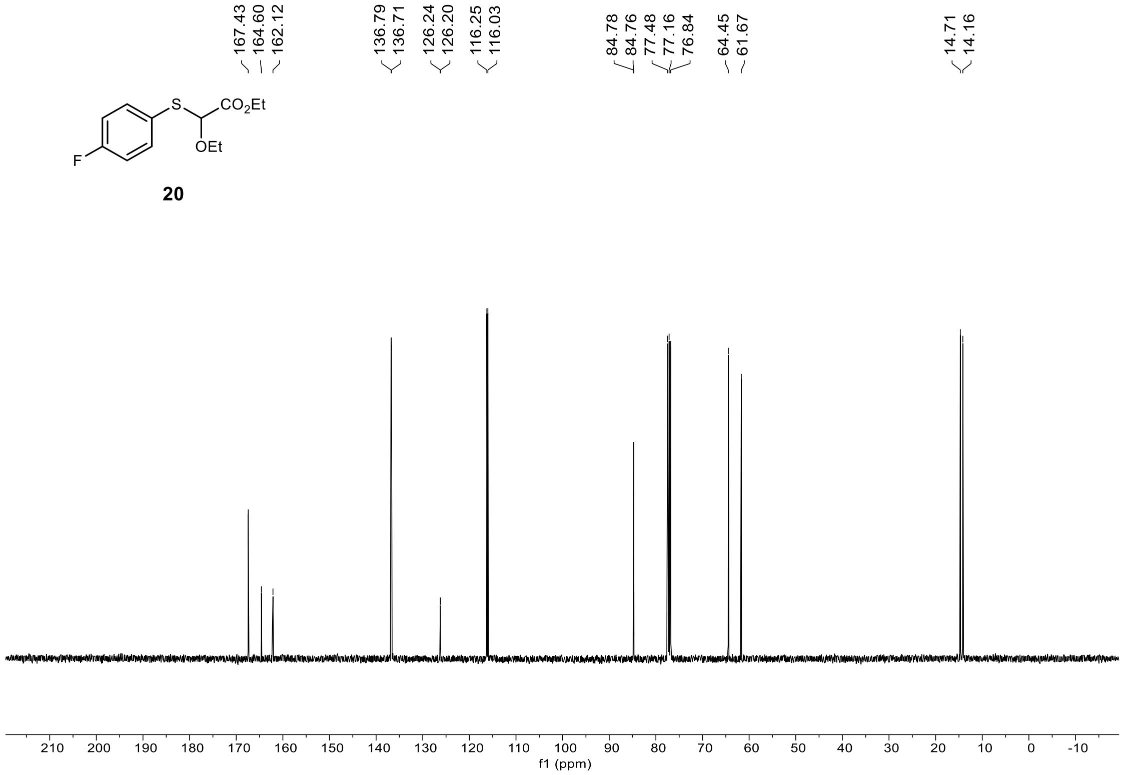

Supplementary Figure 52. ^{13}**C NMR** spectrum of compound **20** (101 MHz, r.t., $CDCl_3$)

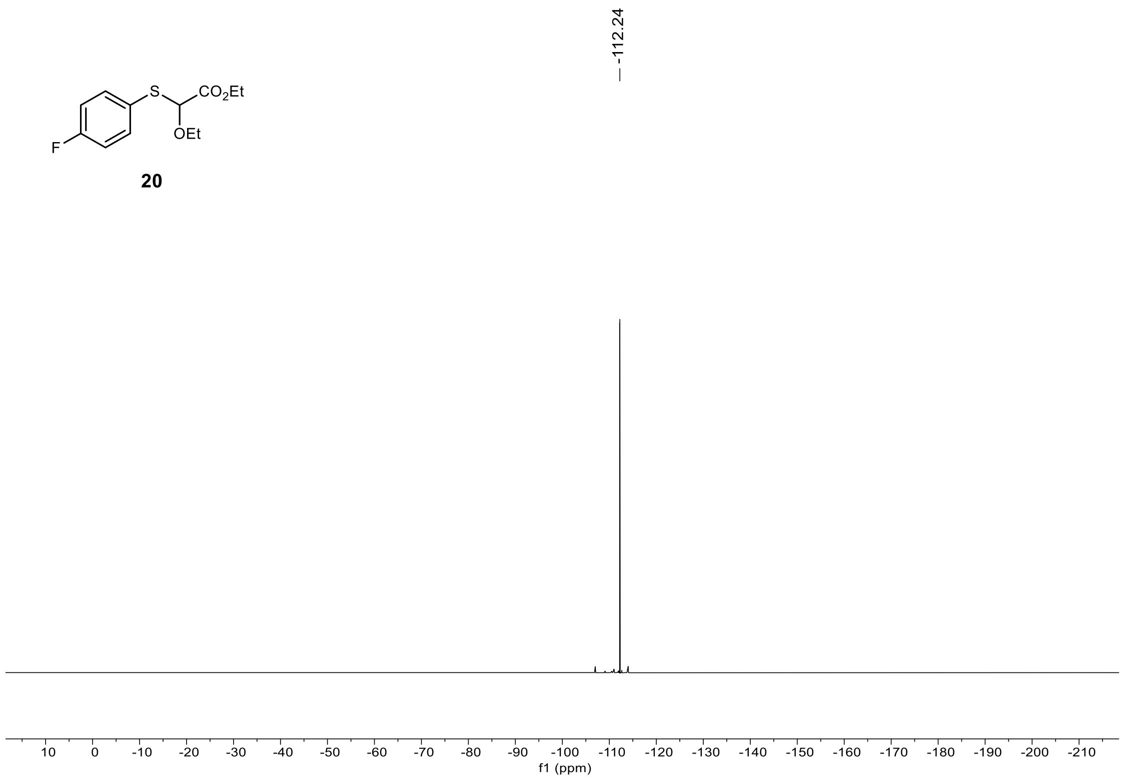

Supplementary Figure 53. **^{19}F NMR** spectrum of compound **20** (377 MHz, r.t., $CDCl_3$)

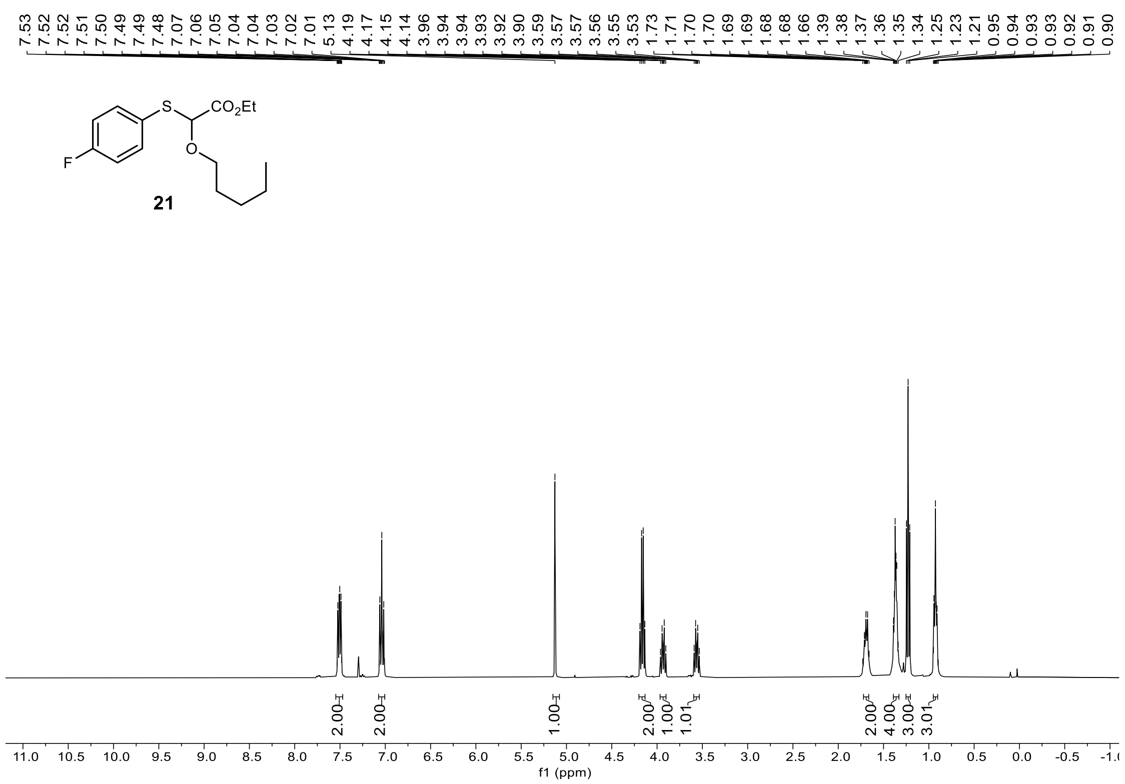

Supplementary Figure 54. **^{1}H NMR** spectrum of compound **21** (400 MHz, r.t., $CDCl_3$)

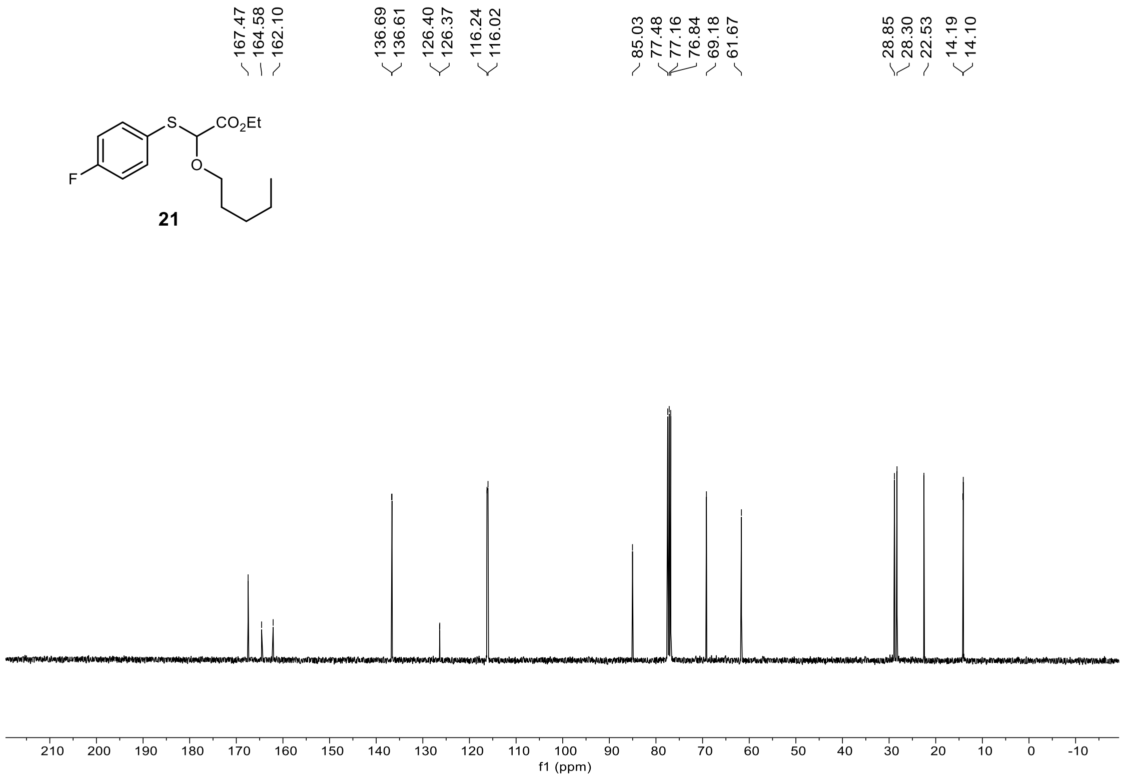

Supplementary Figure 55. **^{13}C NMR** spectrum of compound **21** (101 MHz, r.t., $CDCl_3$)

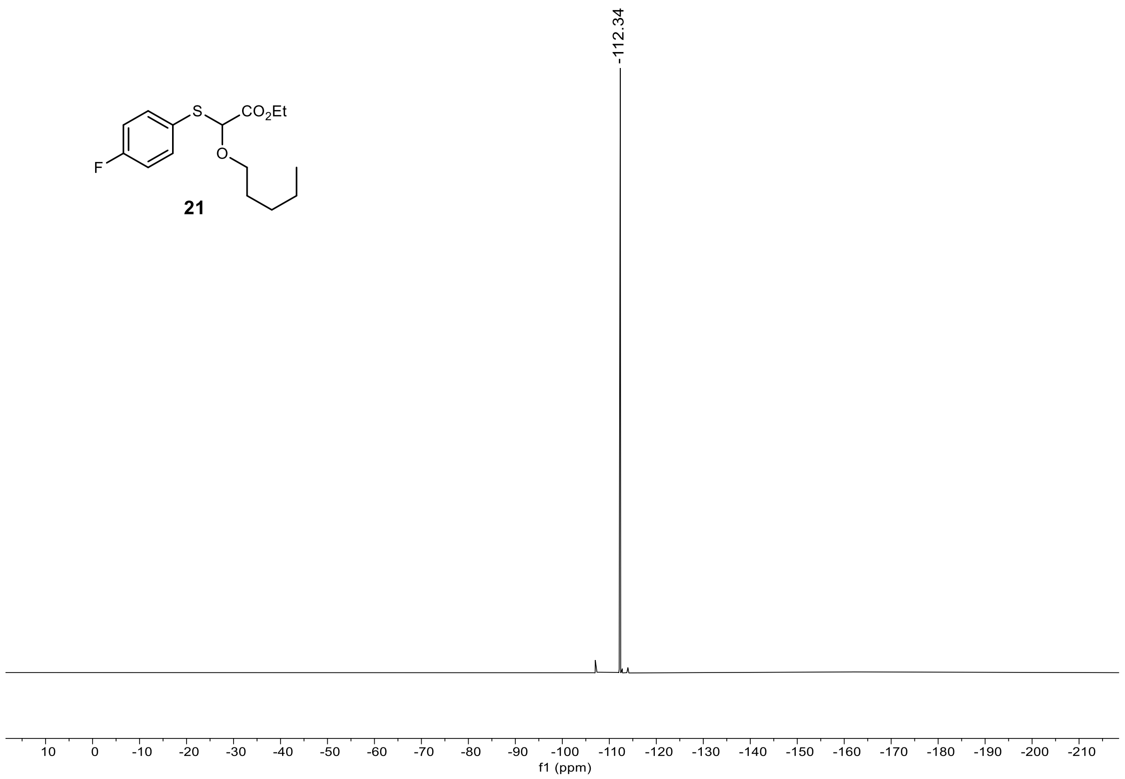

Supplementary Figure 56. ^{19}F NMR spectrum of compound **21** (377 MHz, r.t., $CDCl_3$)

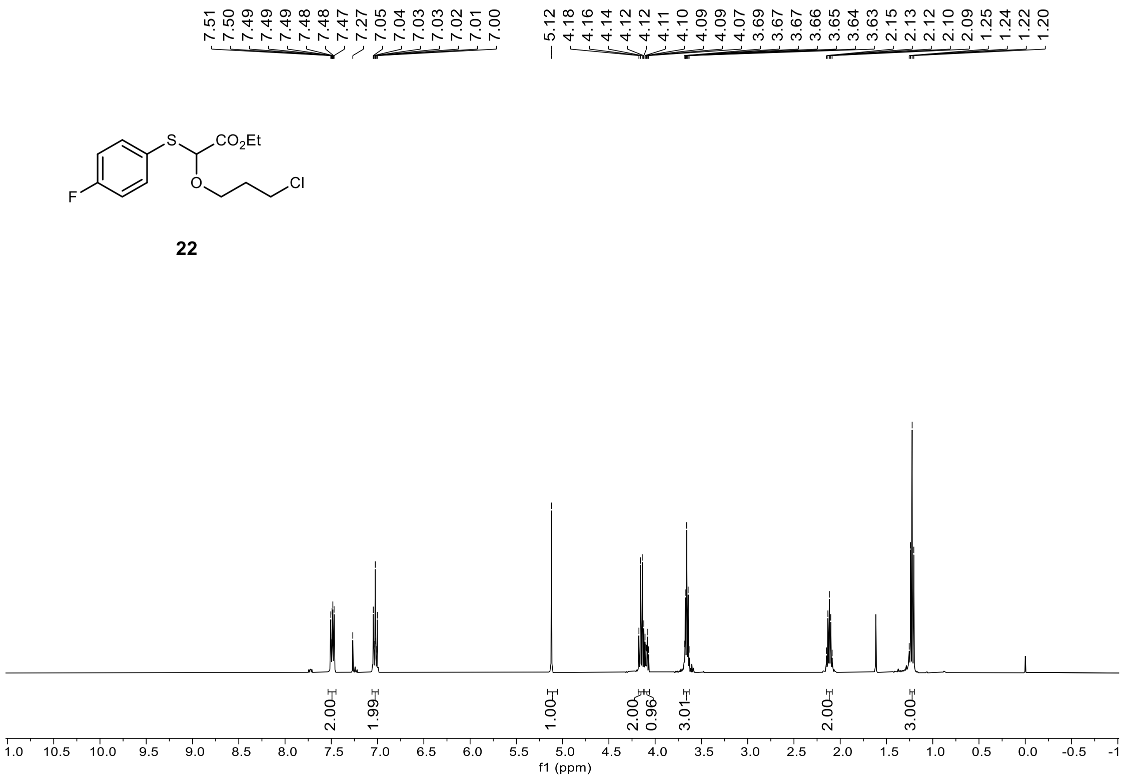

Supplementary Figure 57. ^{1}**H NMR** spectrum of compound **22** (400 MHz, r.t., $CDCl_3$)

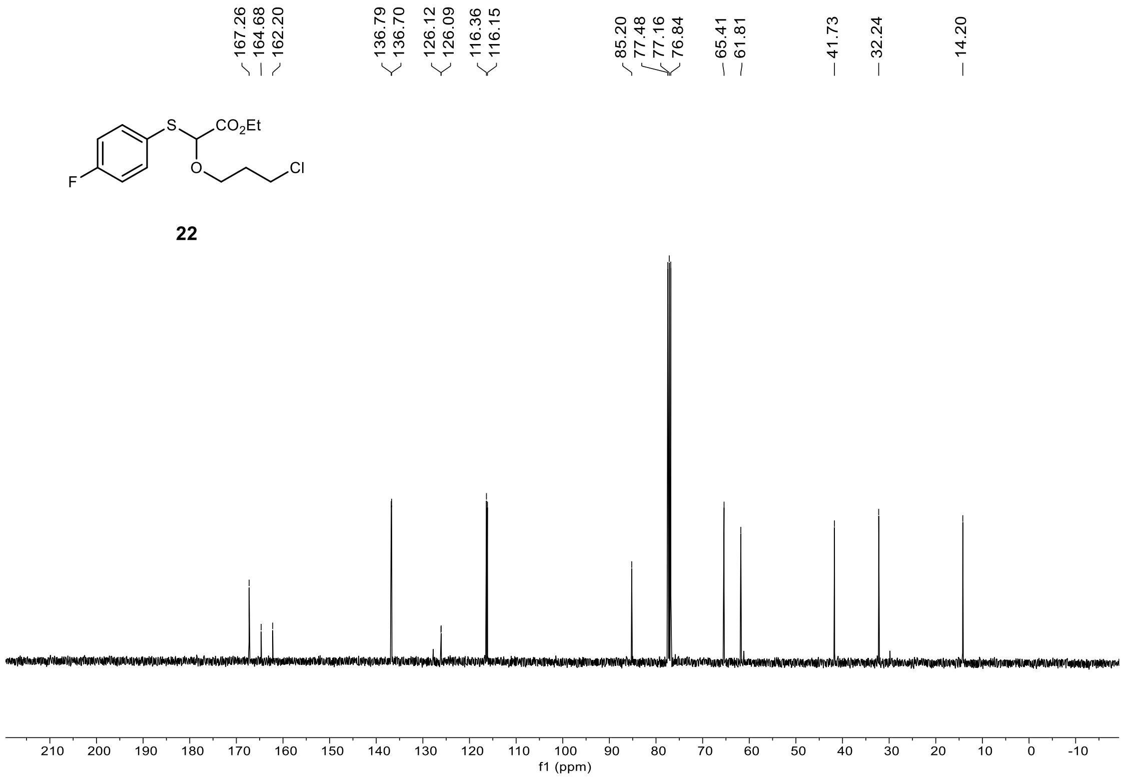

Supplementary Figure 58. ^{13}**C NMR** spectrum of compound **22** (101 MHz, r.t., $CDCl_3$)

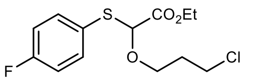

22

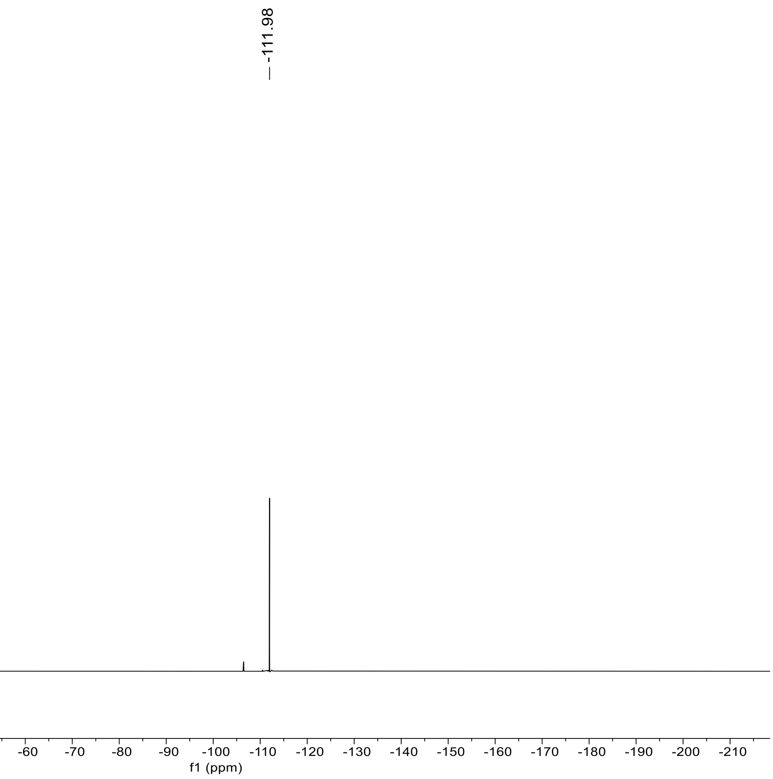

Supplementary Figure 59. ^{19}F NMR spectrum of compound **22** (377 MHz, r.t., $CDCl_3$)

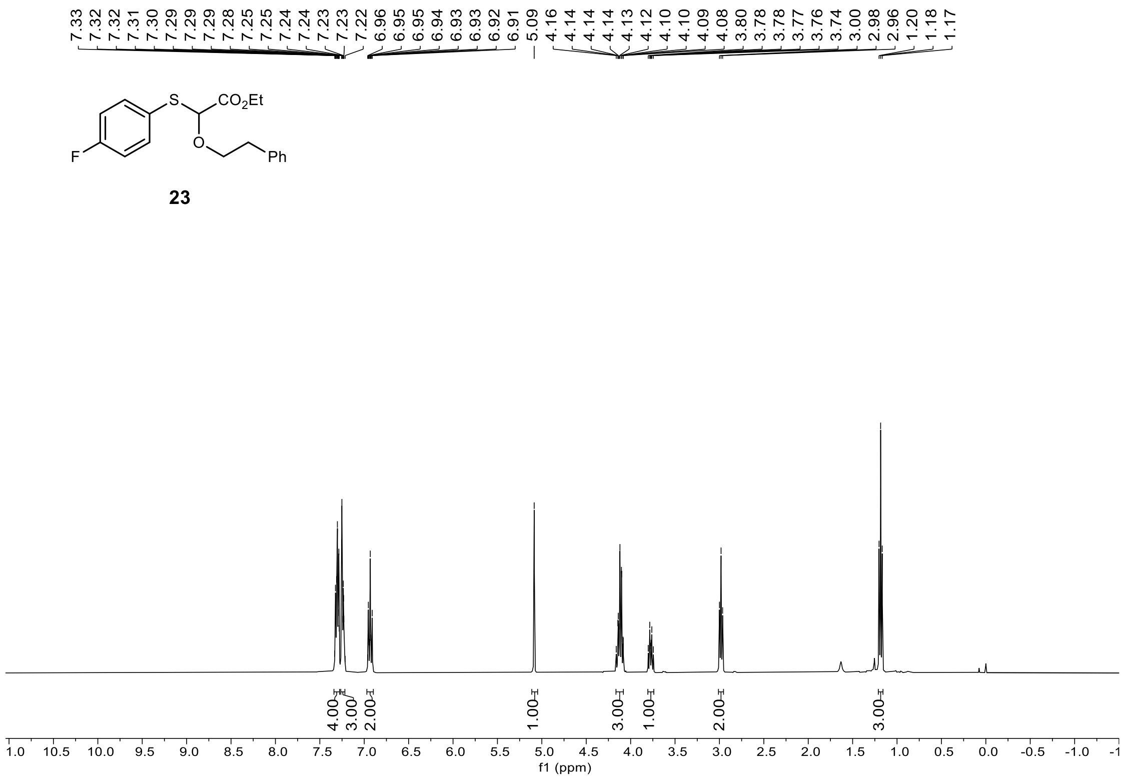

Supplementary Figure 60. **^{1}H NMR** spectrum of compound **23** (400 MHz, r.t., $CDCl_3$)

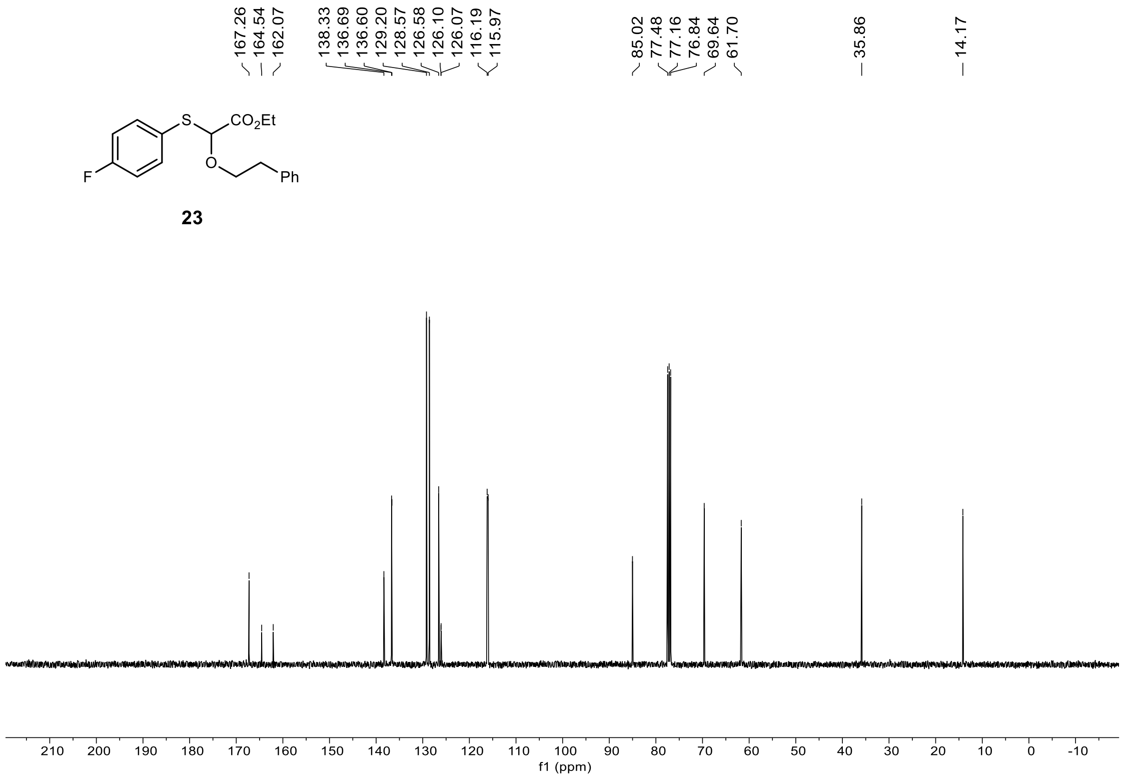

Supplementary Figure 61. **^{13}C NMR** spectrum of compound **23** (101 MHz, r.t., $CDCl_3$)

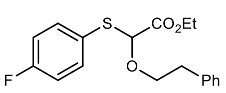

23

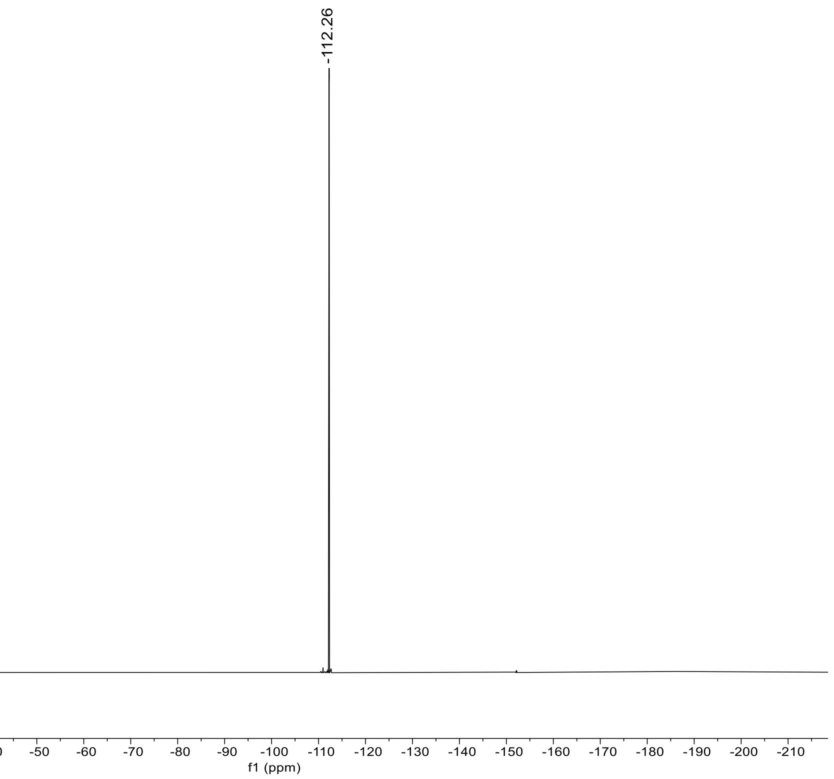

Supplementary Figure 62. **^{19}F NMR** spectrum of compound **23**(377 MHz, r.t., $CDCl_3$)

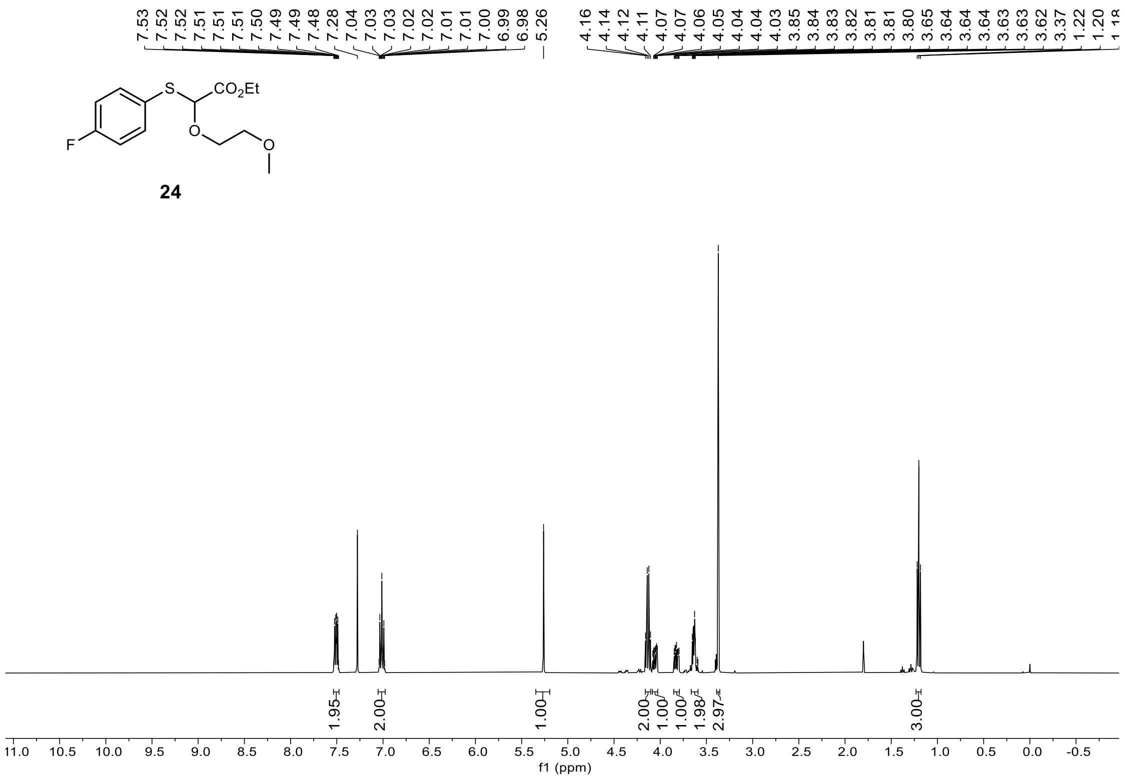

Supplementary Figure 63. ^{1}**H NMR** spectrum of compound **24** (400 MHz, r.t., $CDCl_3$)

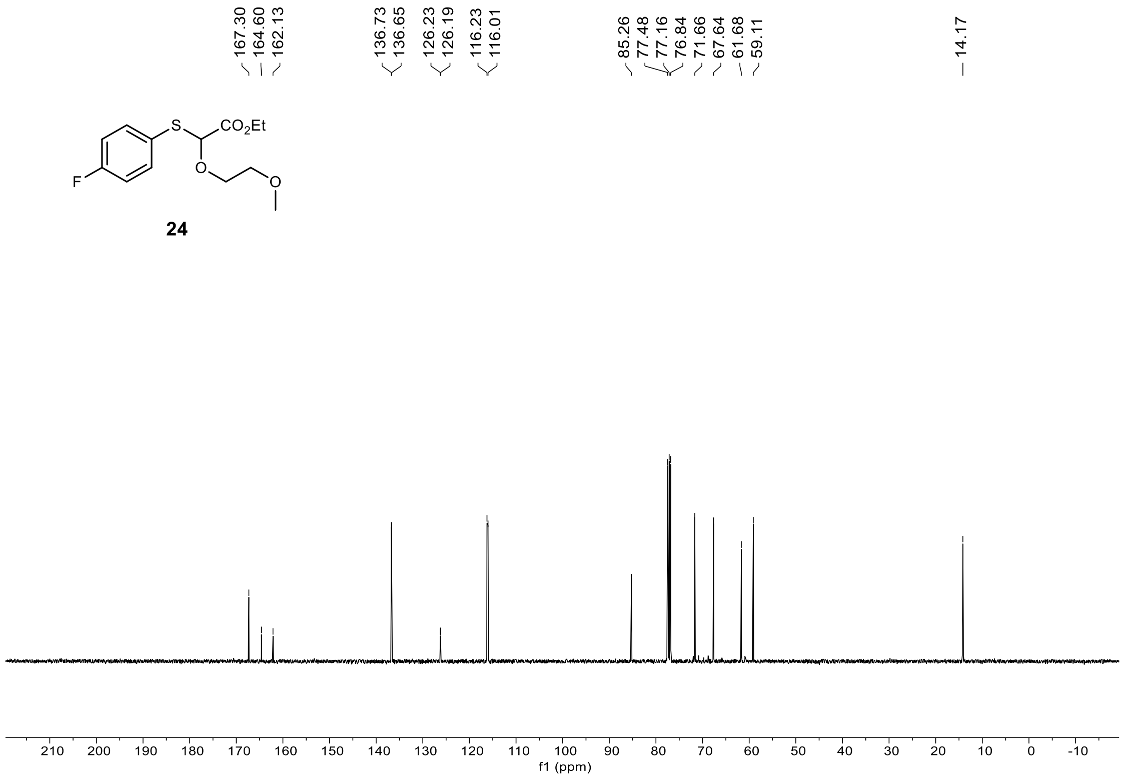

Supplementary Figure 64. ^{13}**C NMR** spectrum of compound **24** (101 MHz, r.t., $CDCl_3$)

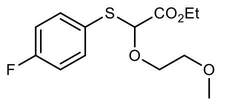

24

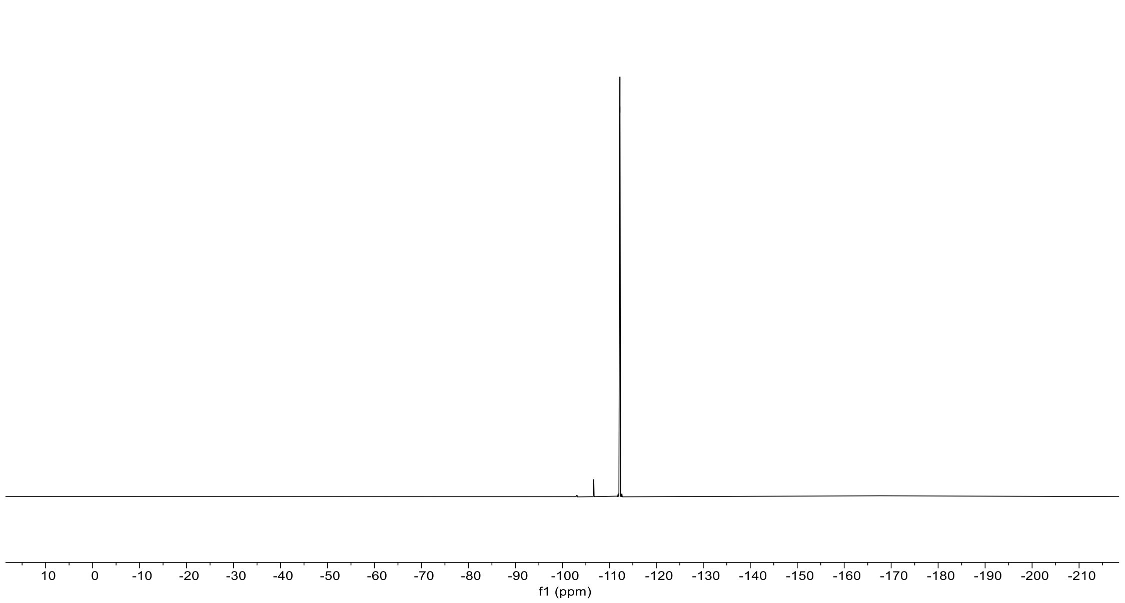

Supplementary Figure 65. ^{19}F NMR spectrum of compound **24** (377 MHz, r.t., $CDCl_3$)

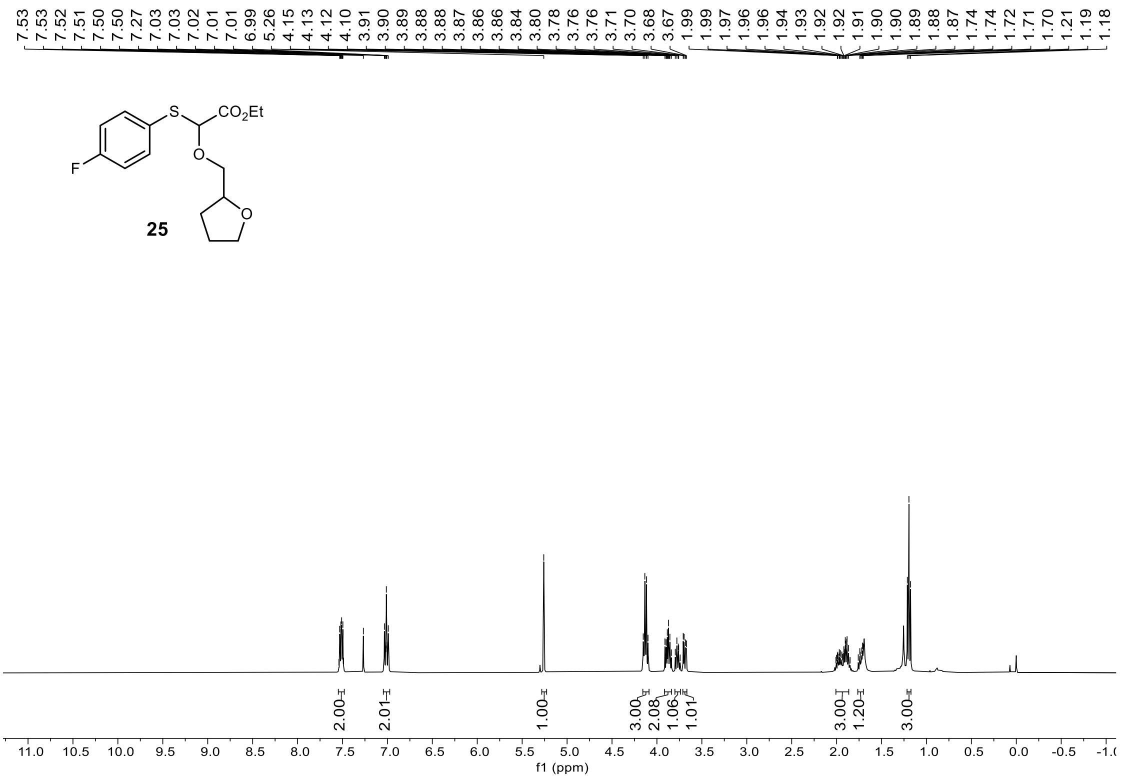

Supplementary Figure 66. ^{1}H NMR spectrum of compound **25** (400 MHz, r.t., $CDCl_3$)

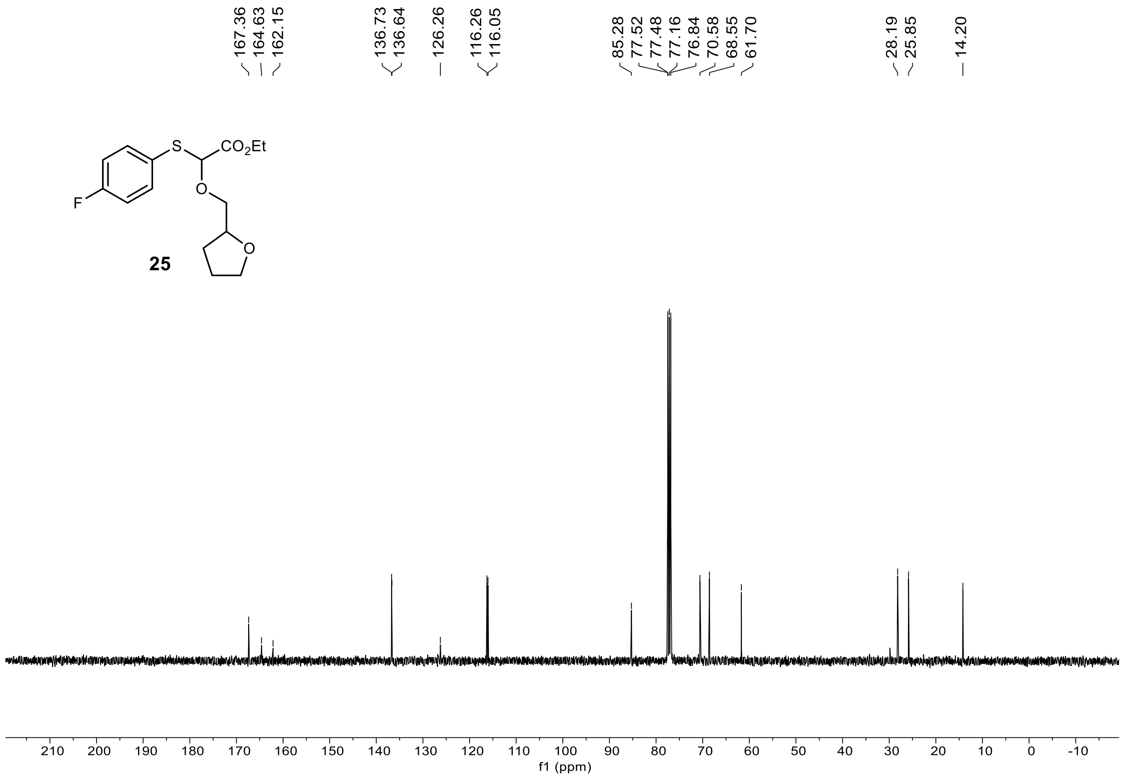

Supplementary Figure 67. ^{13}C NMR spectrum of compound **25** (101 MHz, r.t., $CDCl_3$)

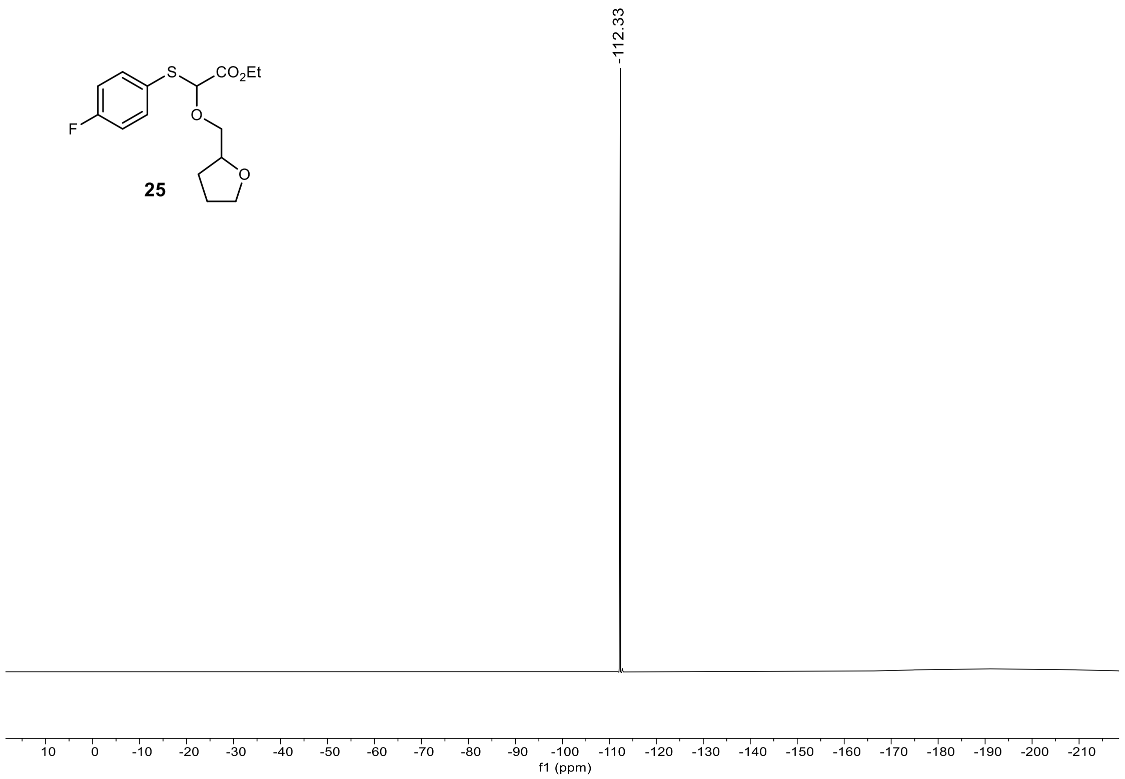

Supplementary Figure 68. ^{19}F NMR spectrum of compound **25** (377 MHz, r.t., $CDCl_3$)

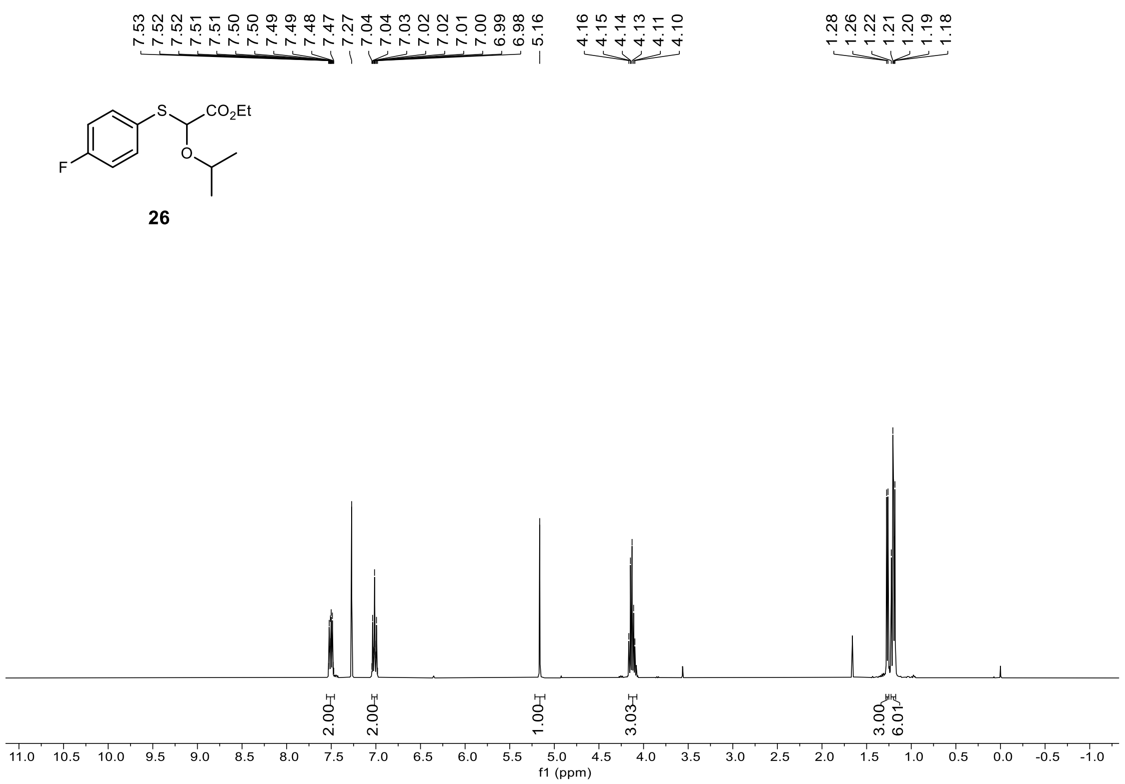

Supplementary Figure 69. ^{1}**H NMR** spectrum of compound **26** (400 MHz, r.t., $CDCl_3$)

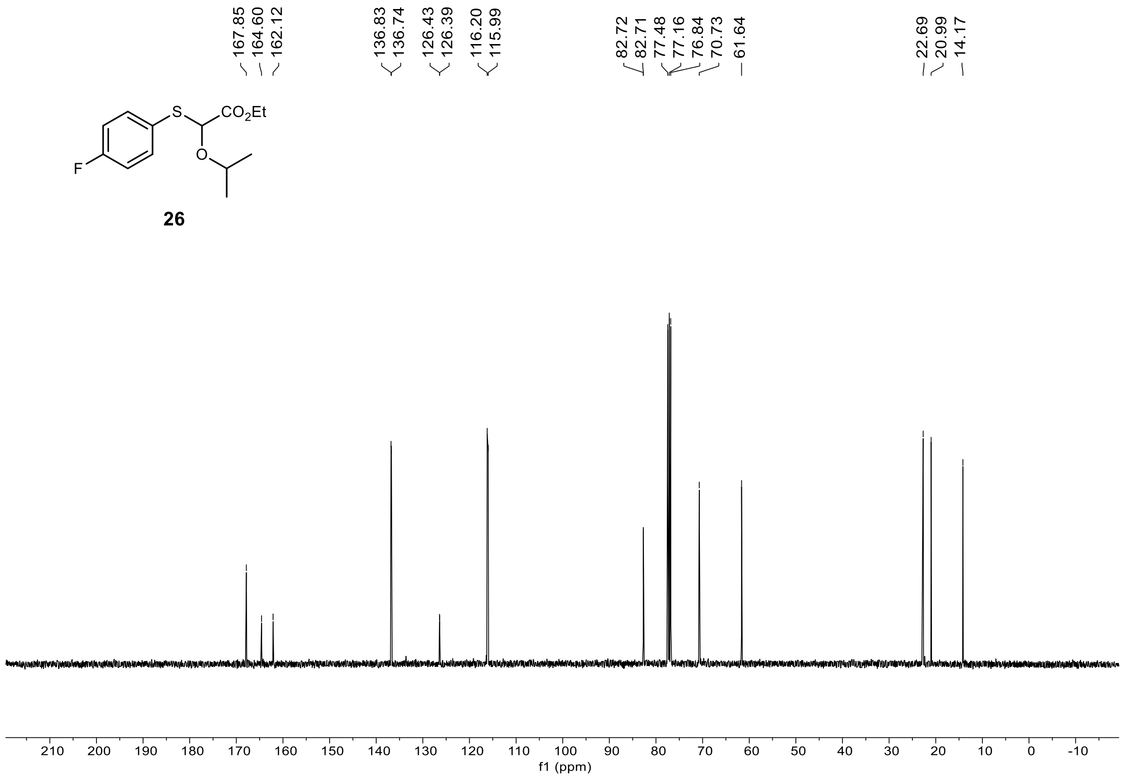

Supplementary Figure 70. ^{13}**C NMR** spectrum of compound **26** (101 MHz, r.t., $CDCl_3$)

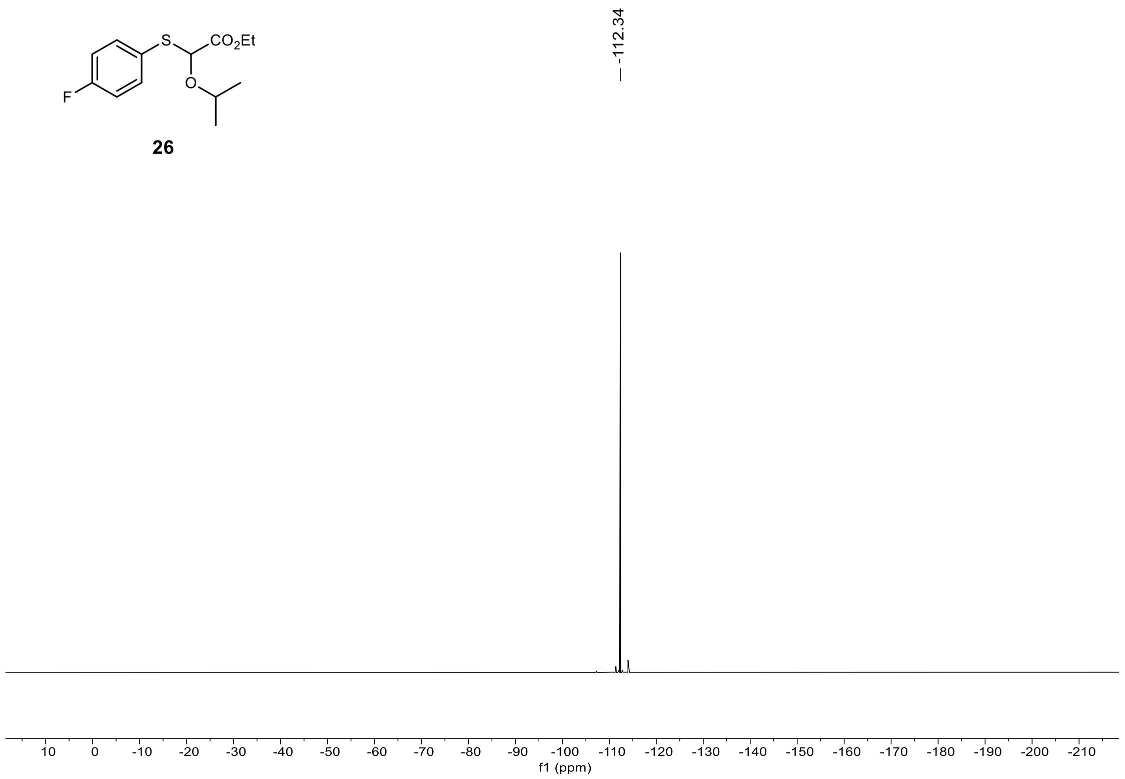

Supplementary Figure 71. ^{19}**F NMR** spectrum of compound **26** (377 MHz, r.t., $CDCl_3$)

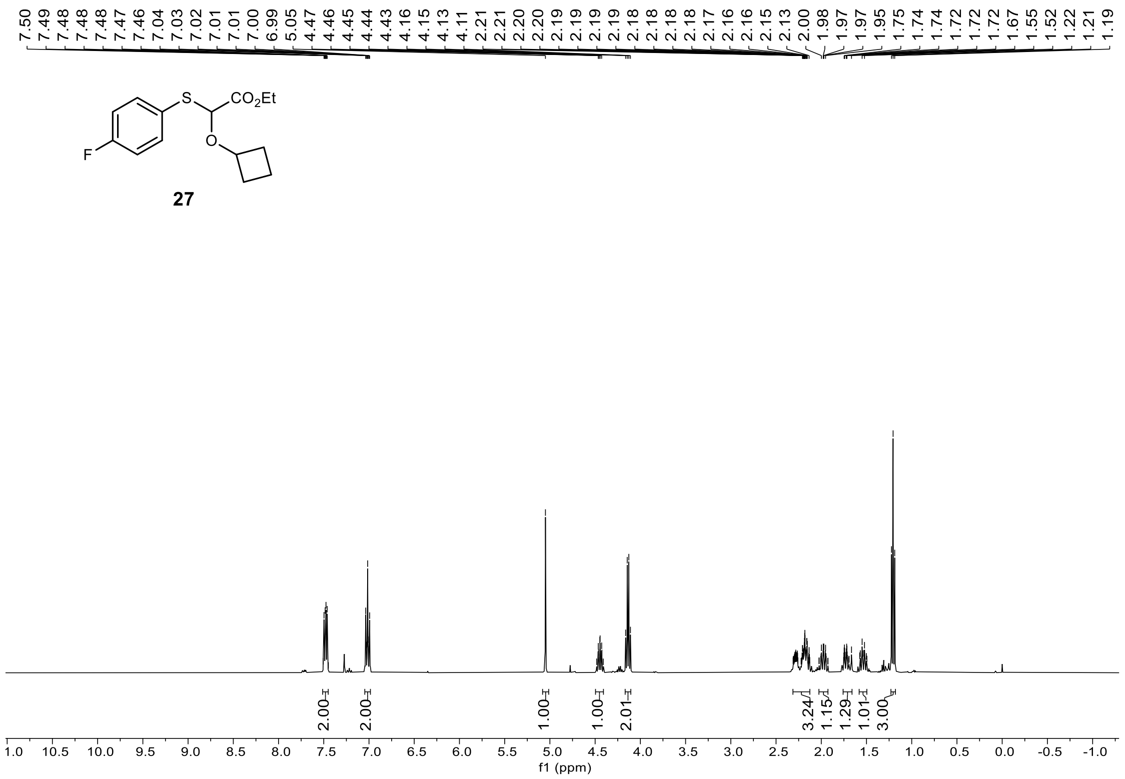

Supplementary Figure 72. ^{1}**H NMR** spectrum of compound **27** (400 MHz, r.t., $CDCl_3$)

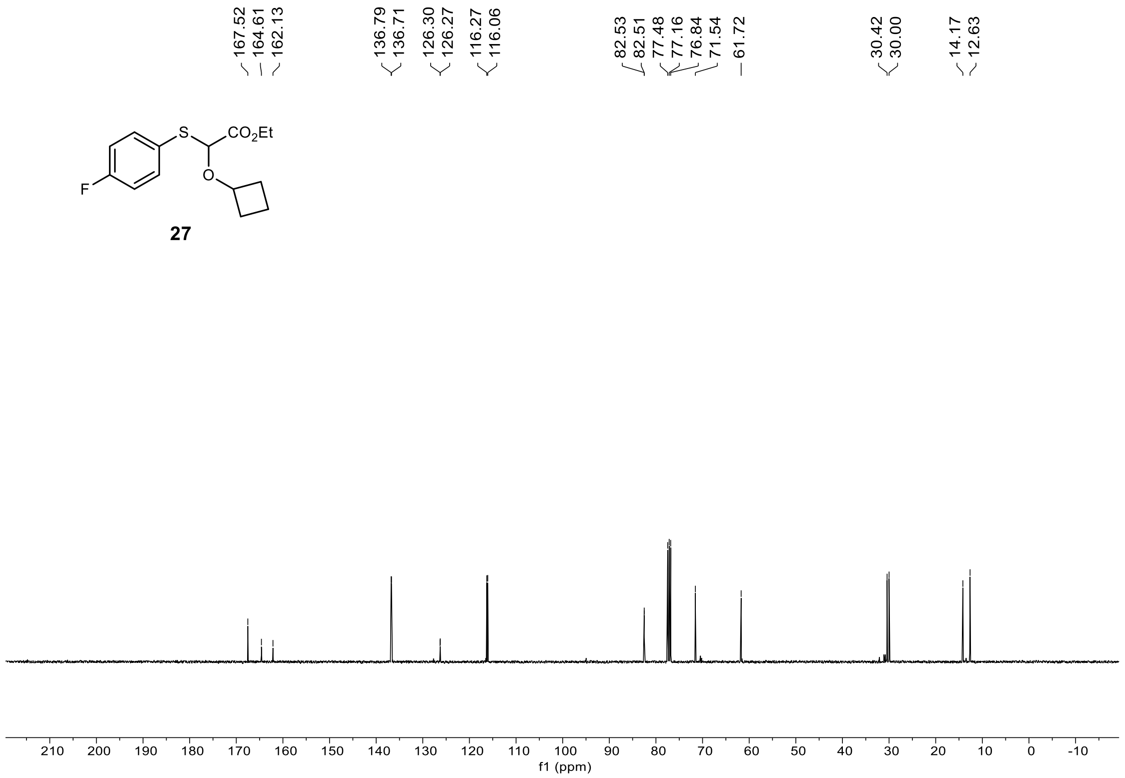

Supplementary Figure 73. ^{13}**C NMR** spectrum of compound **27** (101 MHz, r.t., $CDCl_3$)

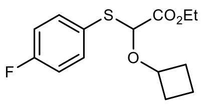

27

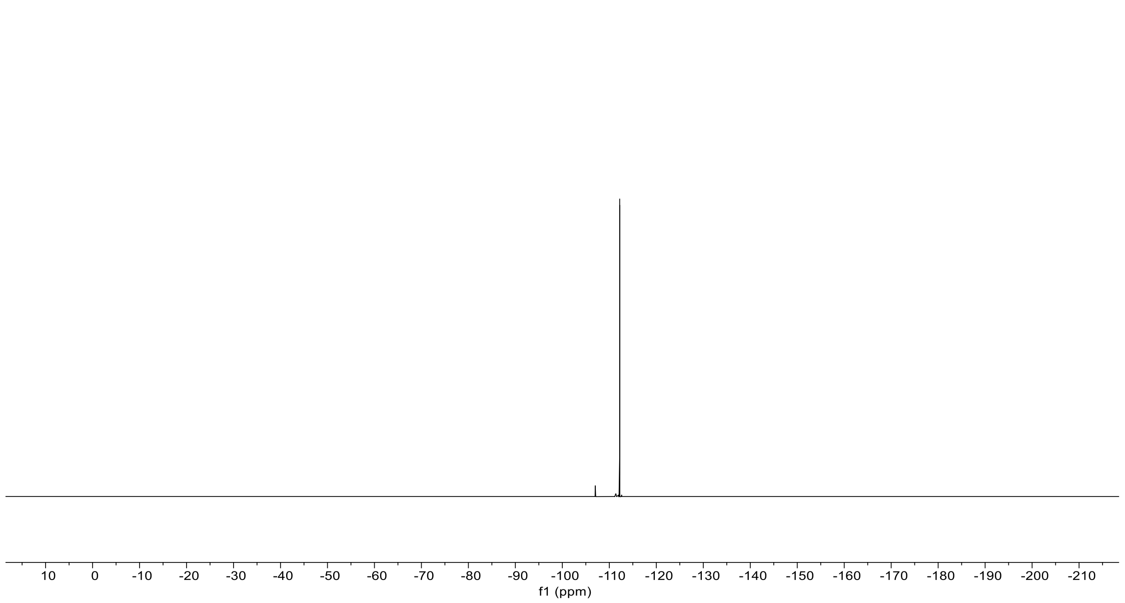

Supplementary Figure 74. ^{19}**F NMR** spectrum of compound **27** (377 MHz, r.t., $CDCl_3$)

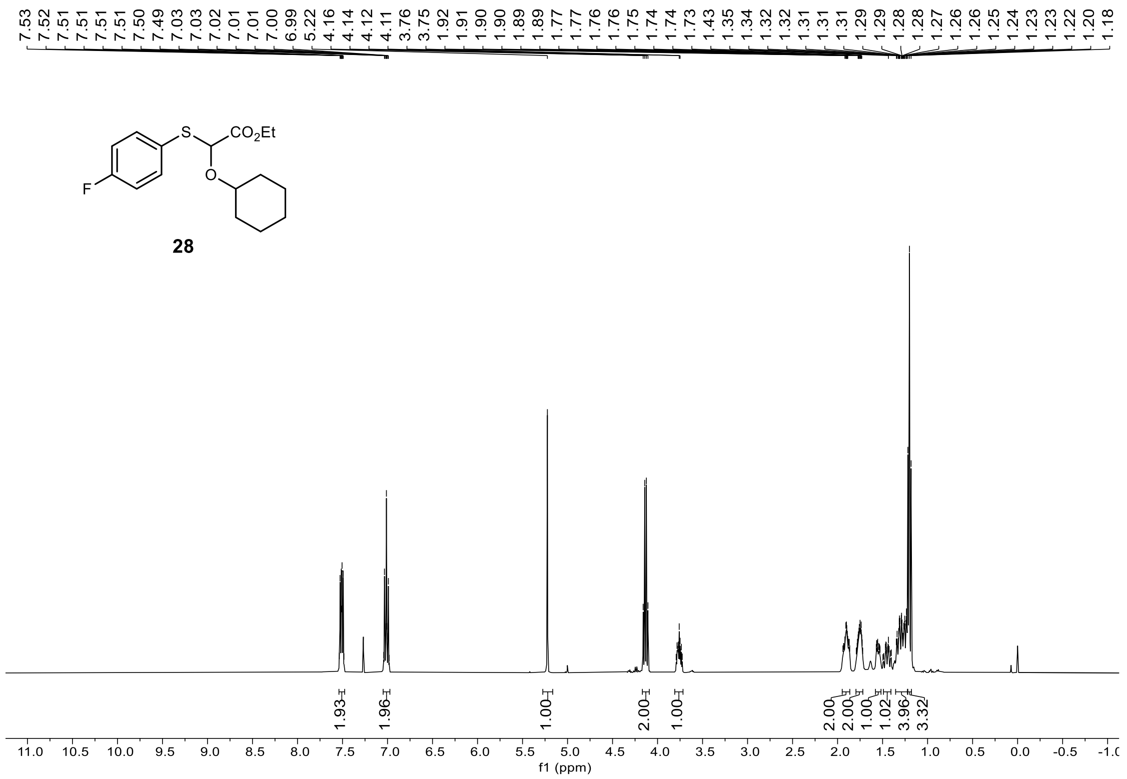

Supplementary Figure 75. **^{1}H NMR** spectrum of compound **28** (400 MHz, r.t., $CDCl_3$)

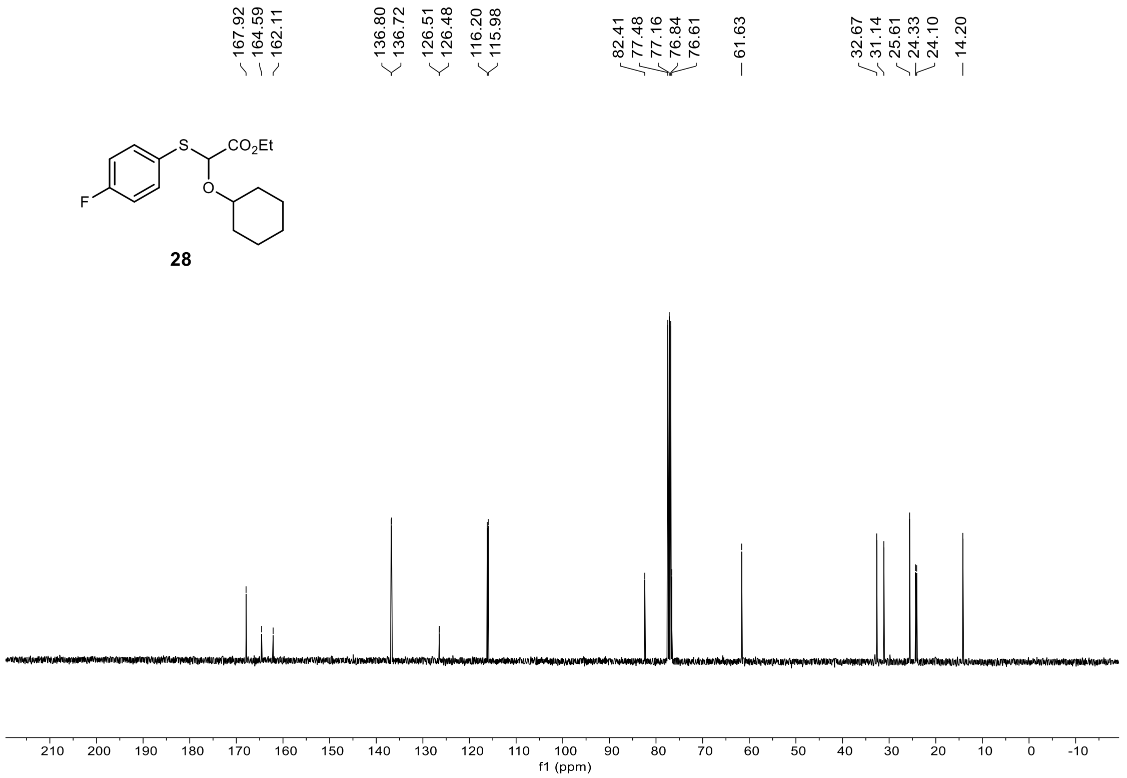

Supplementary Figure 76. **^{13}C NMR** spectrum of compound **28** (101 MHz, r.t., $CDCl_3$)

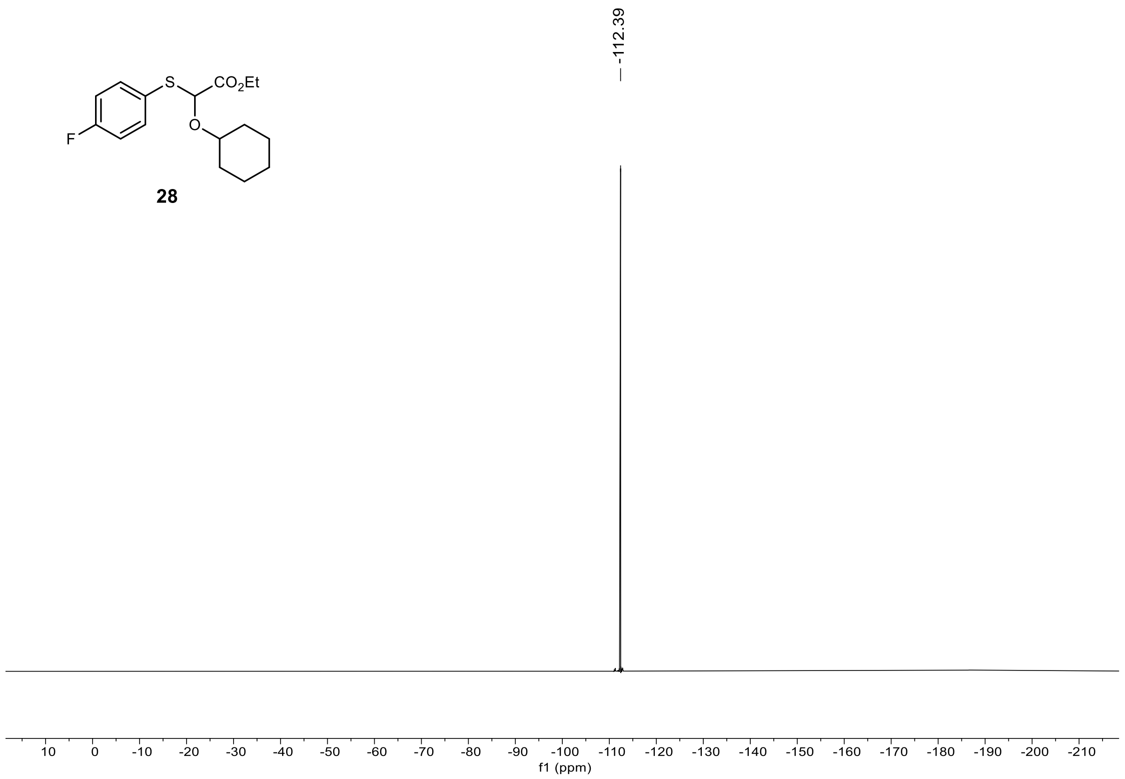

Supplementary Figure 77. ^{19}F NMR spectrum of compound **28** (377 MHz, r.t., $CDCl_3$)

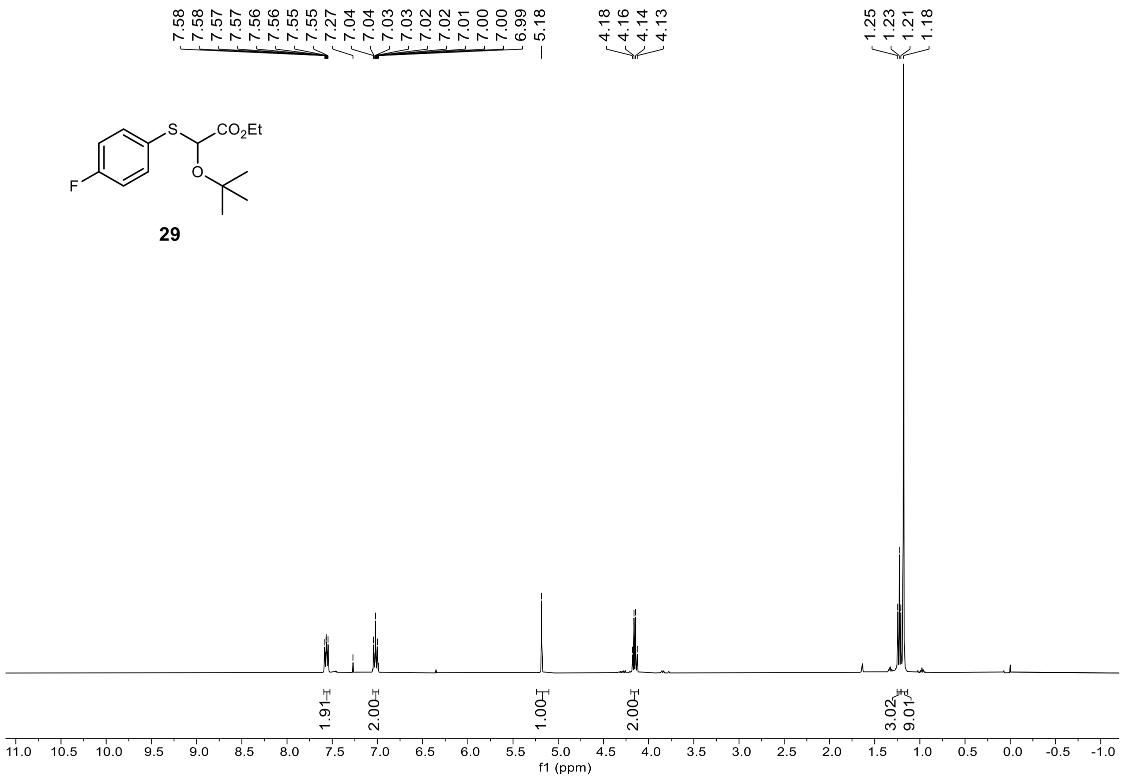

Supplementary Figure 78. ^{1}**H NMR** spectrum of compound **29** (400 MHz, r.t., $CDCl_3$)

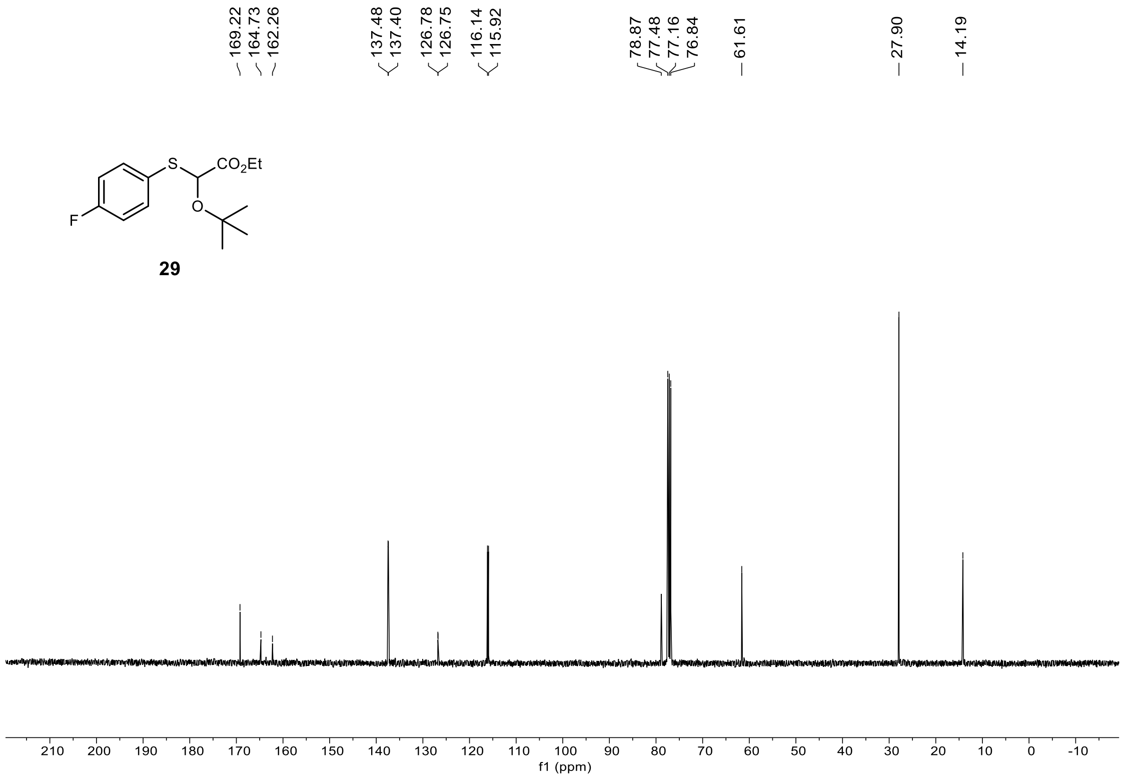

Supplementary Figure 79. ^{13}**C NMR** spectrum of compound **29** (101 MHz, r.t., $CDCl_3$)

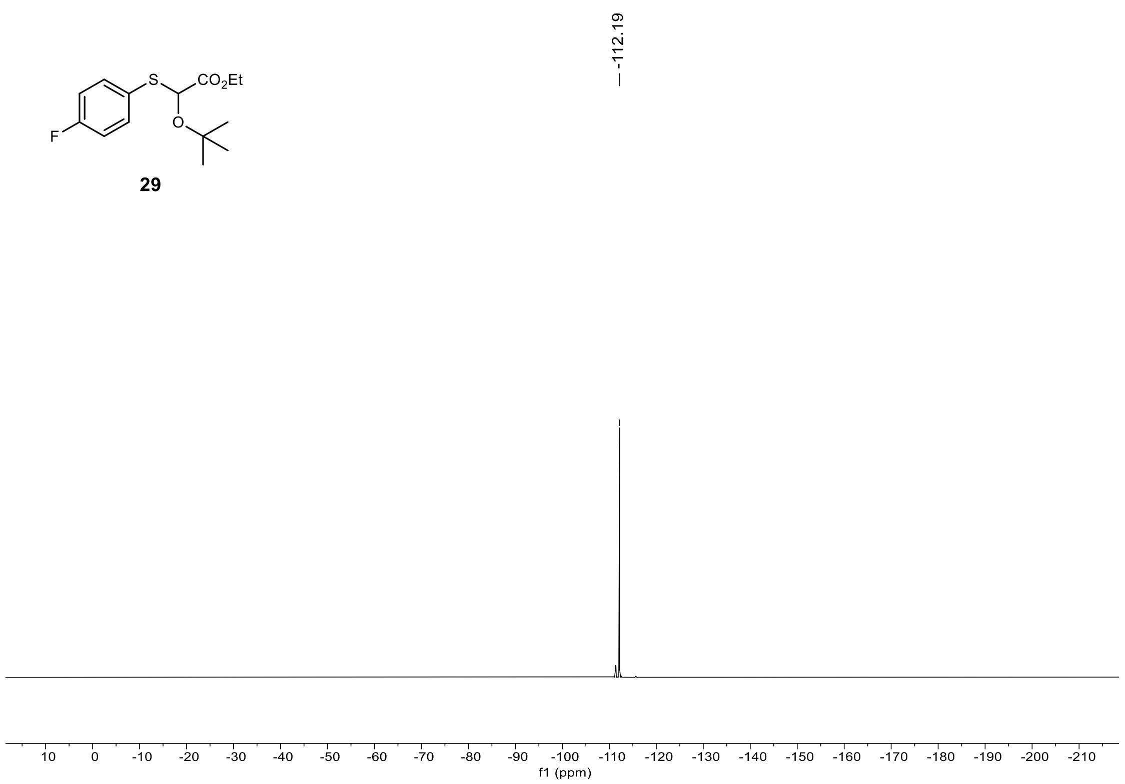

Supplementary Figure 80. ^{19}F NMR spectrum of compound **29** (377 MHz, r.t., $CDCl_3$)

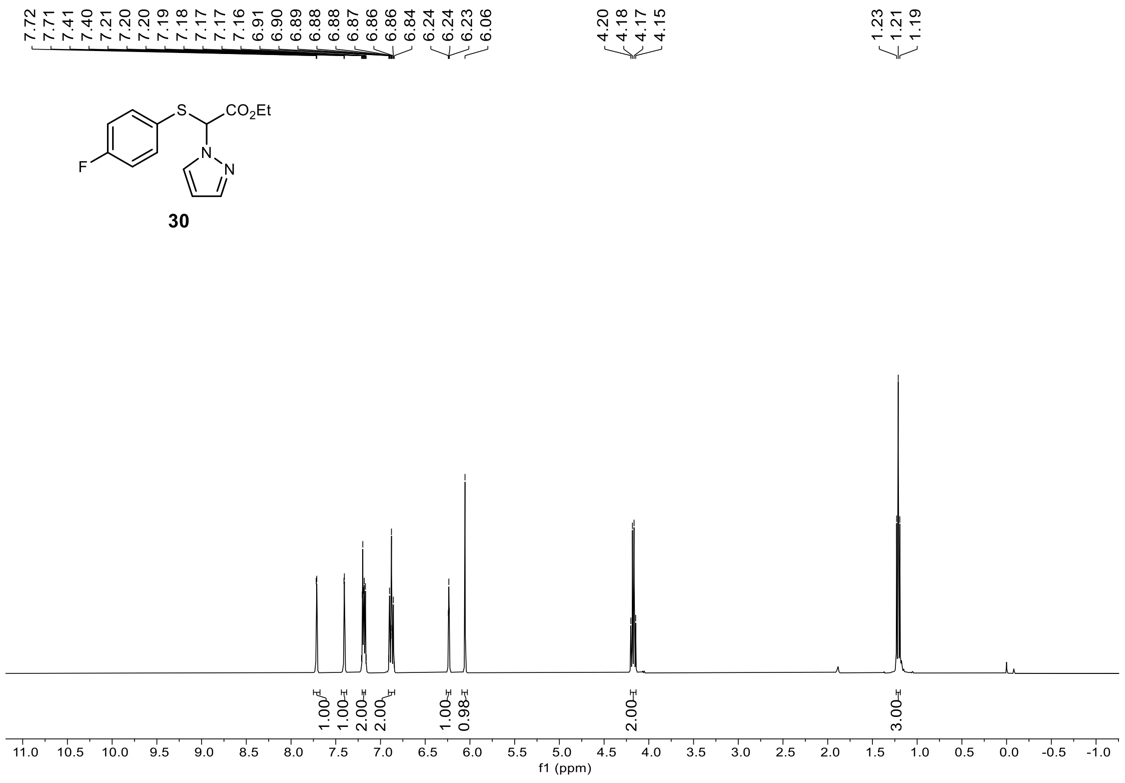

Supplementary Figure 81. ^{1}**H NMR** spectrum of compound **30** (400 MHz, r.t., $CDCl_3$)

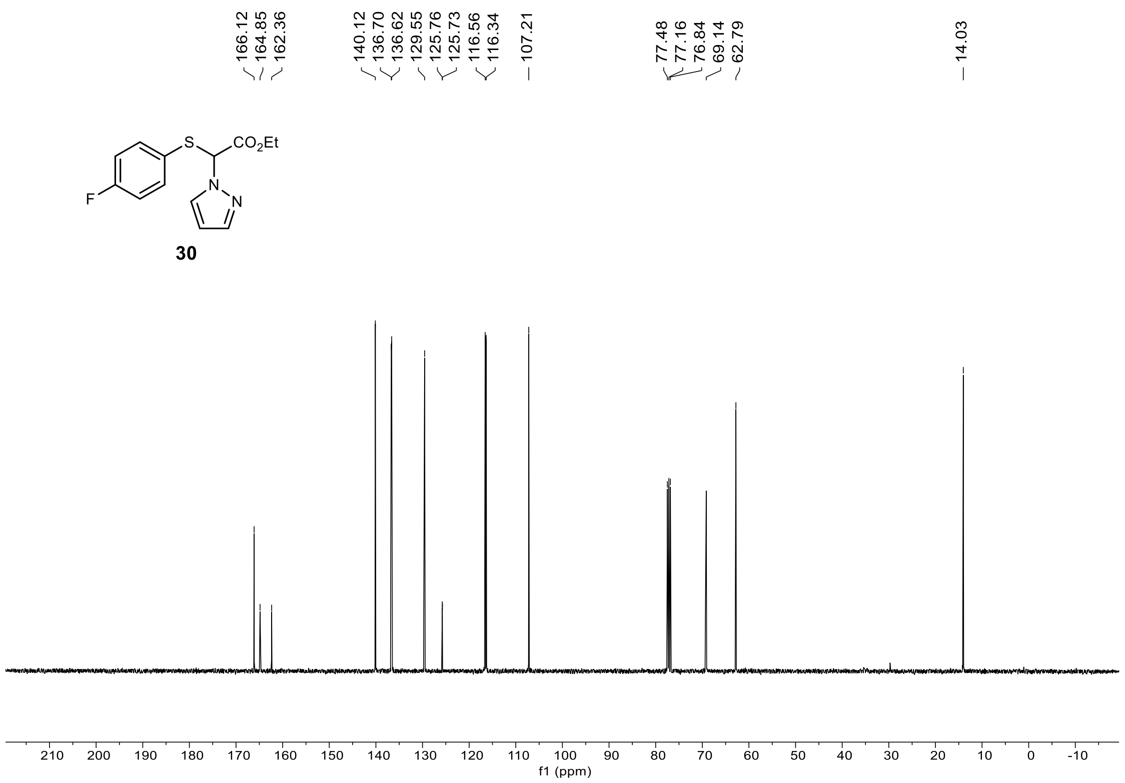

Supplementary Figure 82. ^{13}**C NMR** spectrum of compound **30** (101 MHz, r.t., $CDCl_3$)

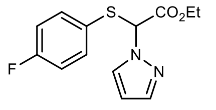

30

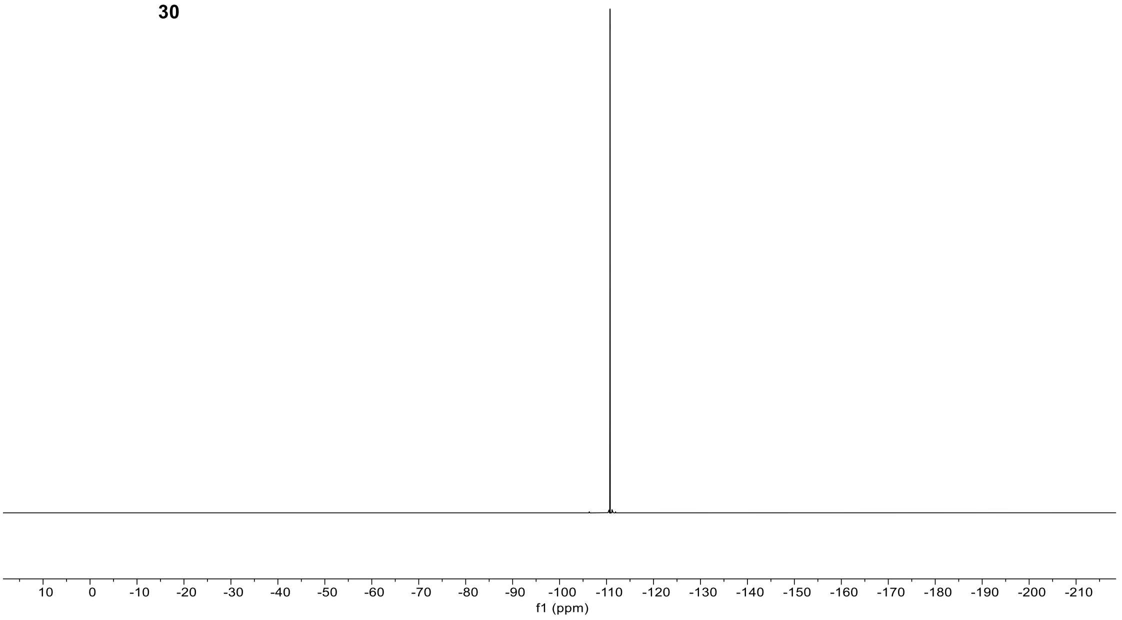

Supplementary Figure 83. ^{19}**F NMR** spectrum of compound **30** (377 MHz, r.t., $CDCl_3$)

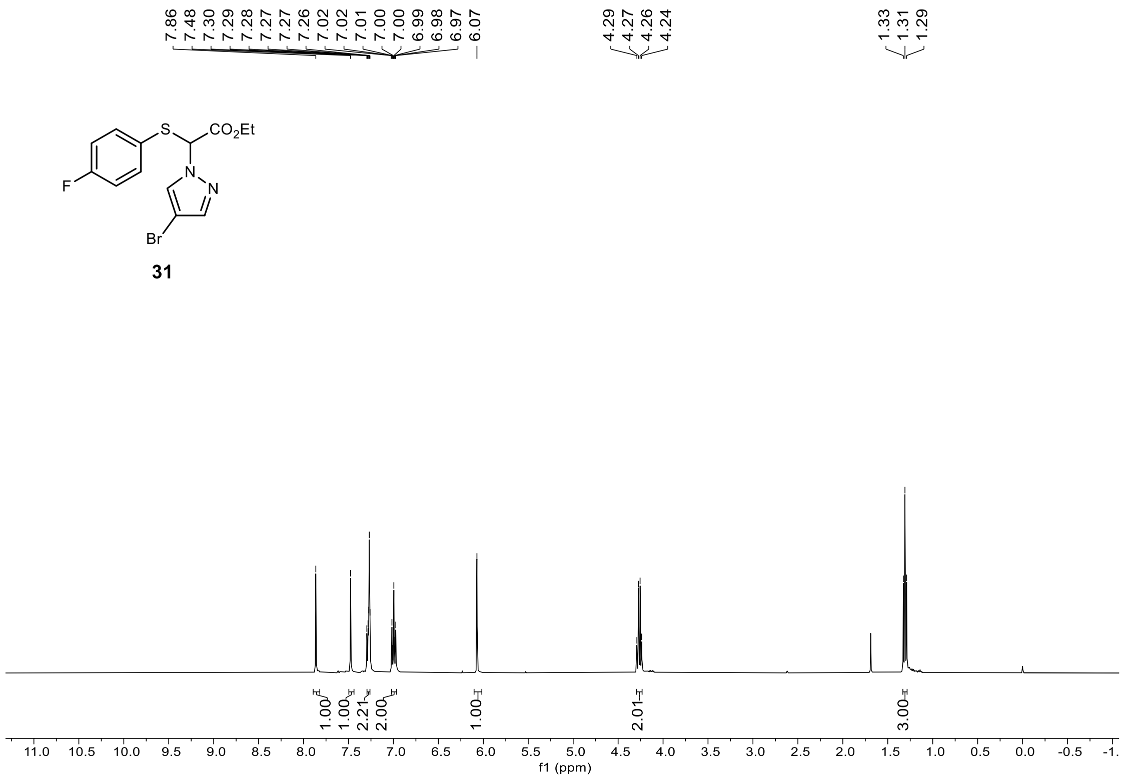

Supplementary Figure 84. ^{1}**H NMR** spectrum of compound **31** (400 MHz, r.t., $CDCl_3$)

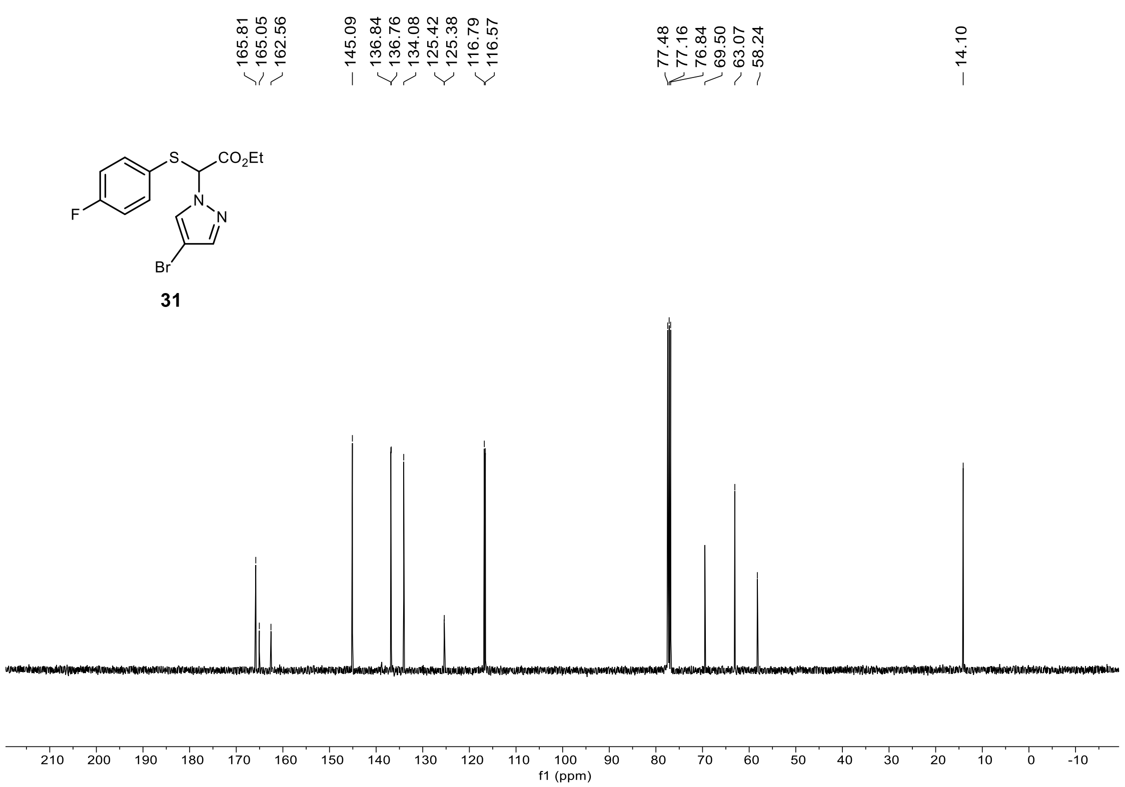

Supplementary Figure 85. ^{13}**C NMR** spectrum of compound **31** (101 MHz, r.t., $CDCl_3$)

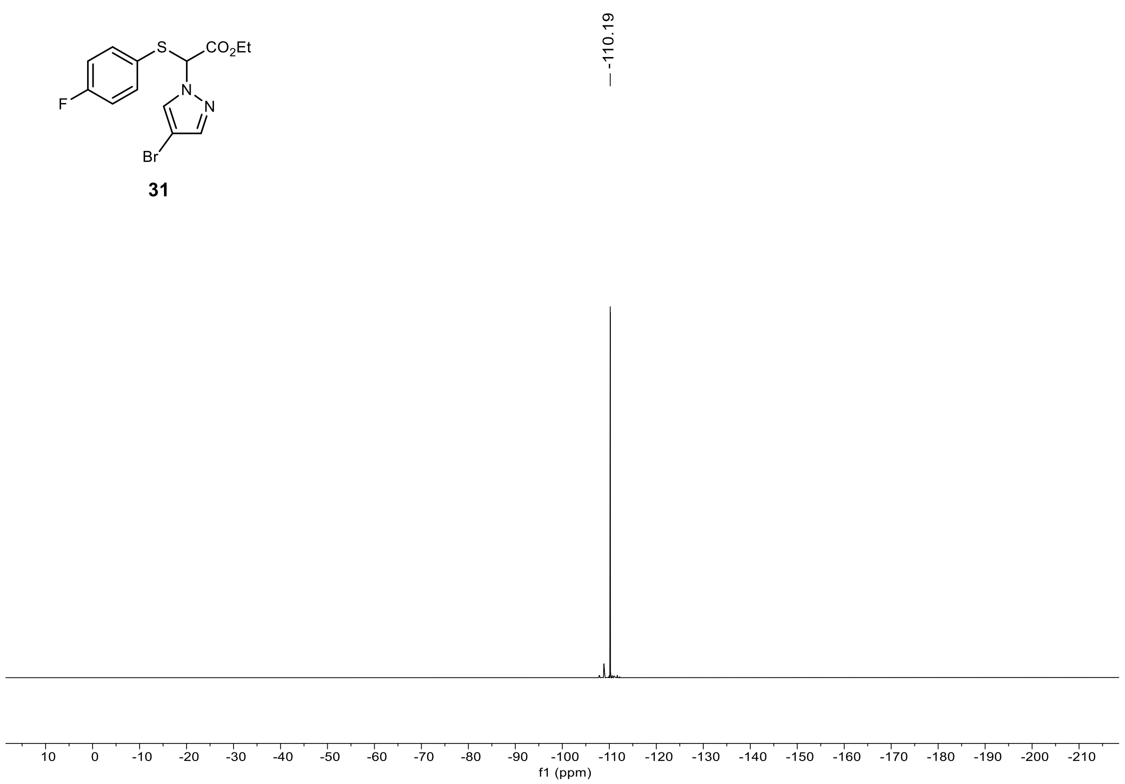

Supplementary Figure 86. ^{19}**F NMR** spectrum of compound **31** (377 MHz, r.t., $CDCl_3$)

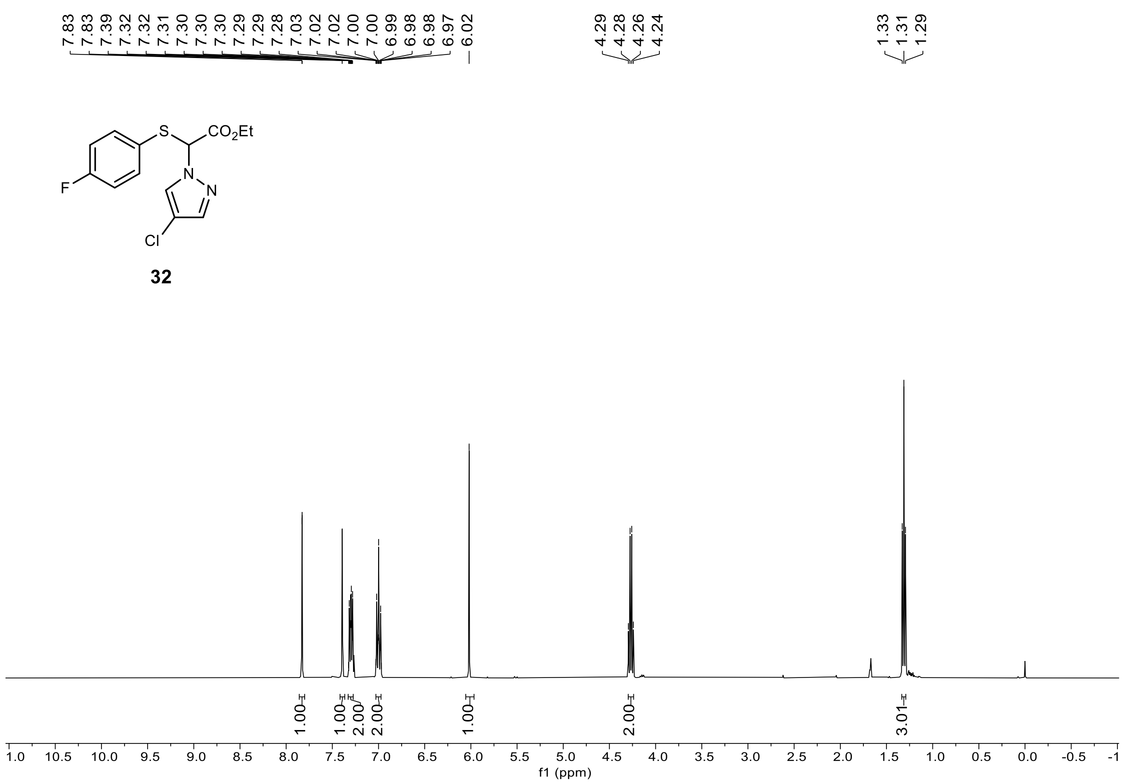

Supplementary Figure 87. ^{1}**H NMR** spectrum of compound **32** (400 MHz, r.t., $CDCl_3$)

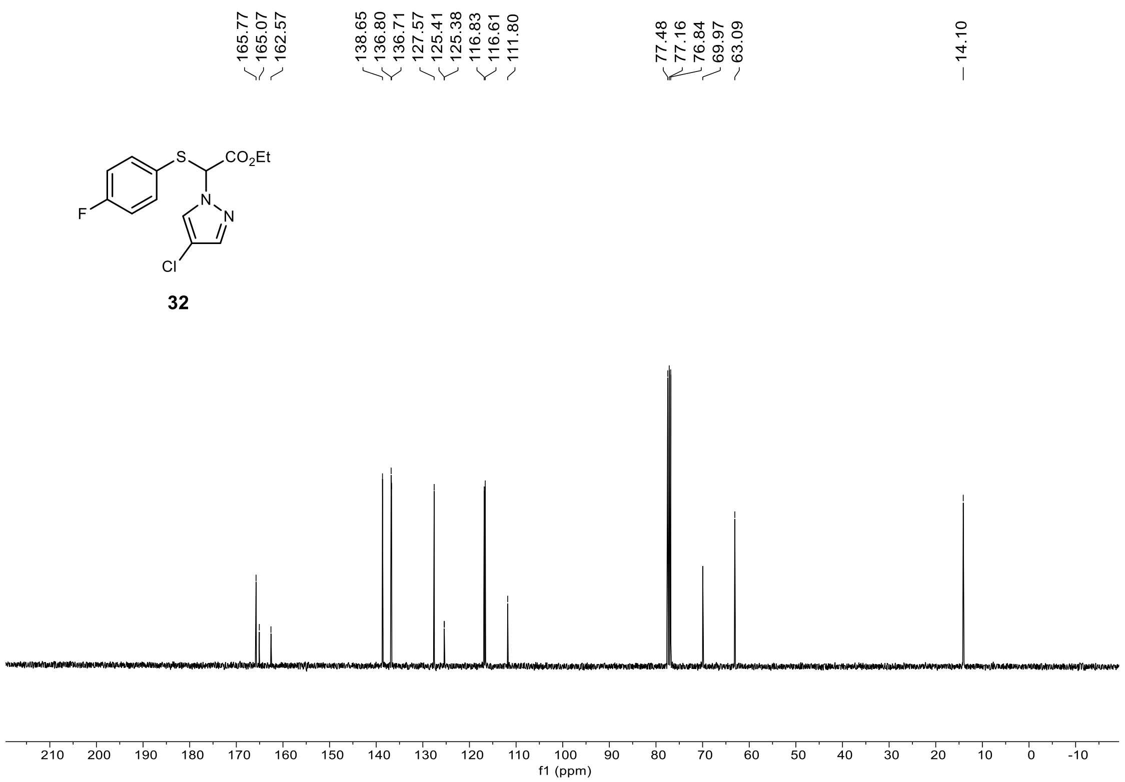

Supplementary Figure 88. ^{13}**C NMR** spectrum of compound **32** (101 MHz, r.t., $CDCl_3$)

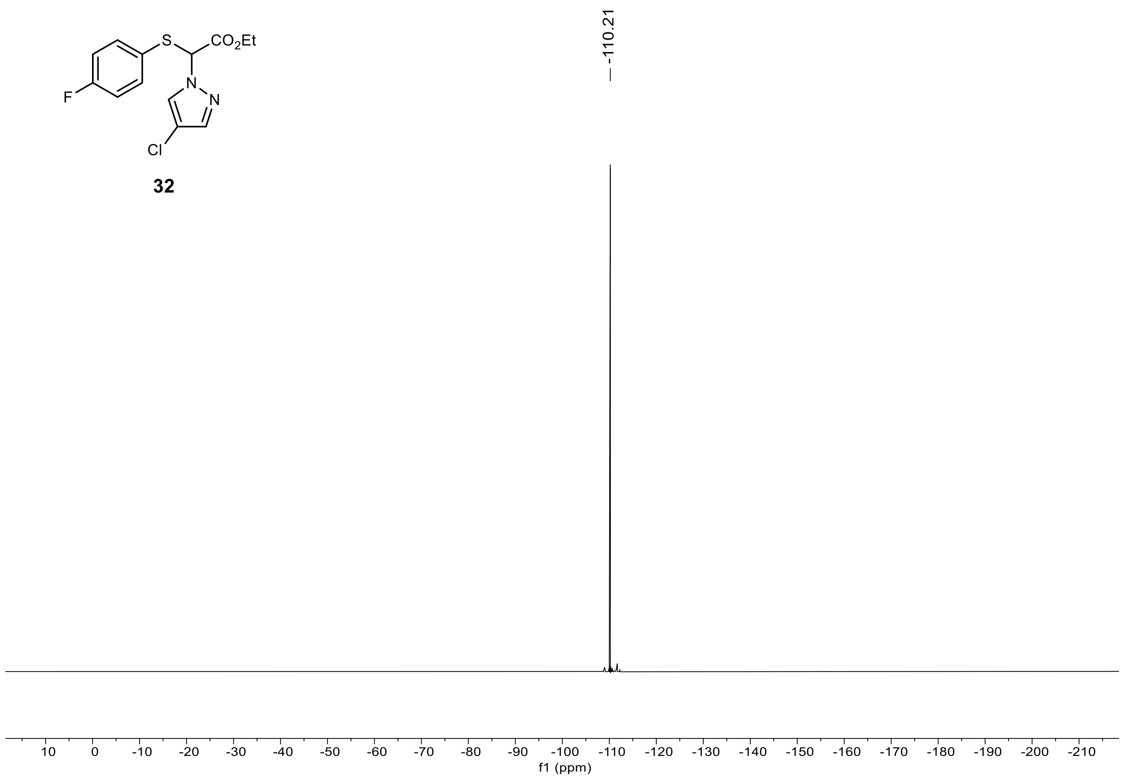

Supplementary Figure 89. ^{19}**F NMR** spectrum of compound **32** (377 MHz, r.t., $CDCl_3$)

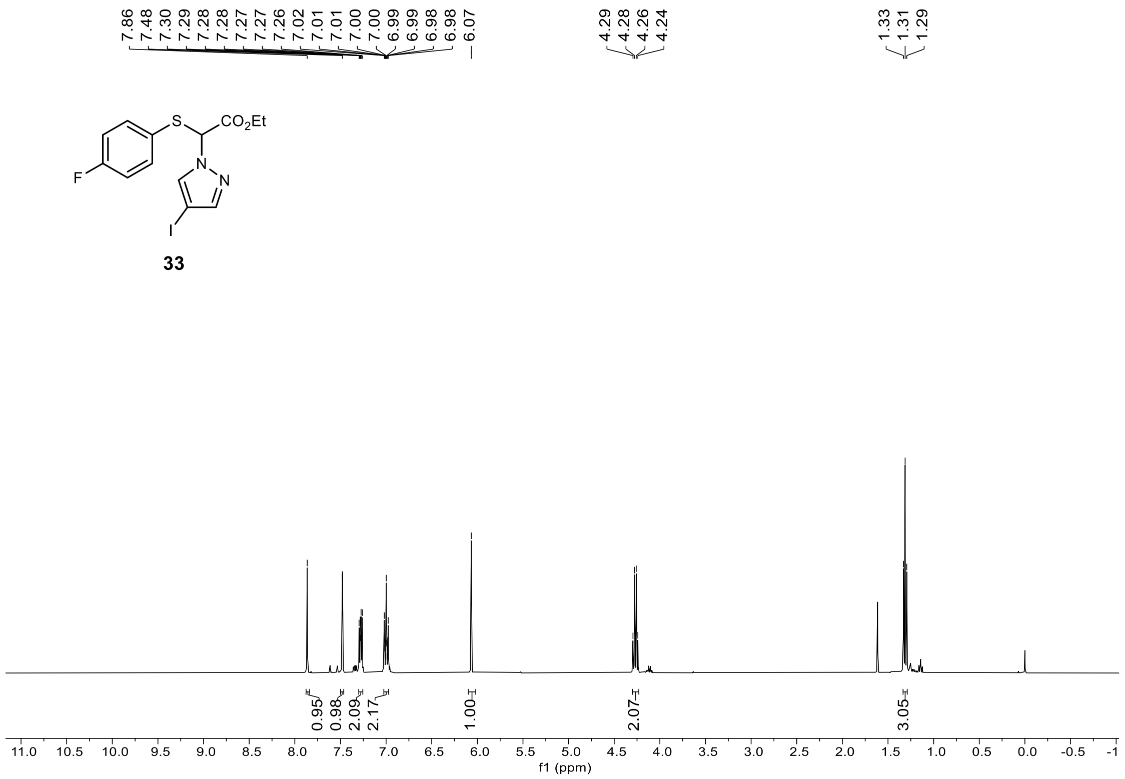

Supplementary Figure 90. ^{1}**H NMR** spectrum of compound **33** (400 MHz, r.t., $CDCl_3$)

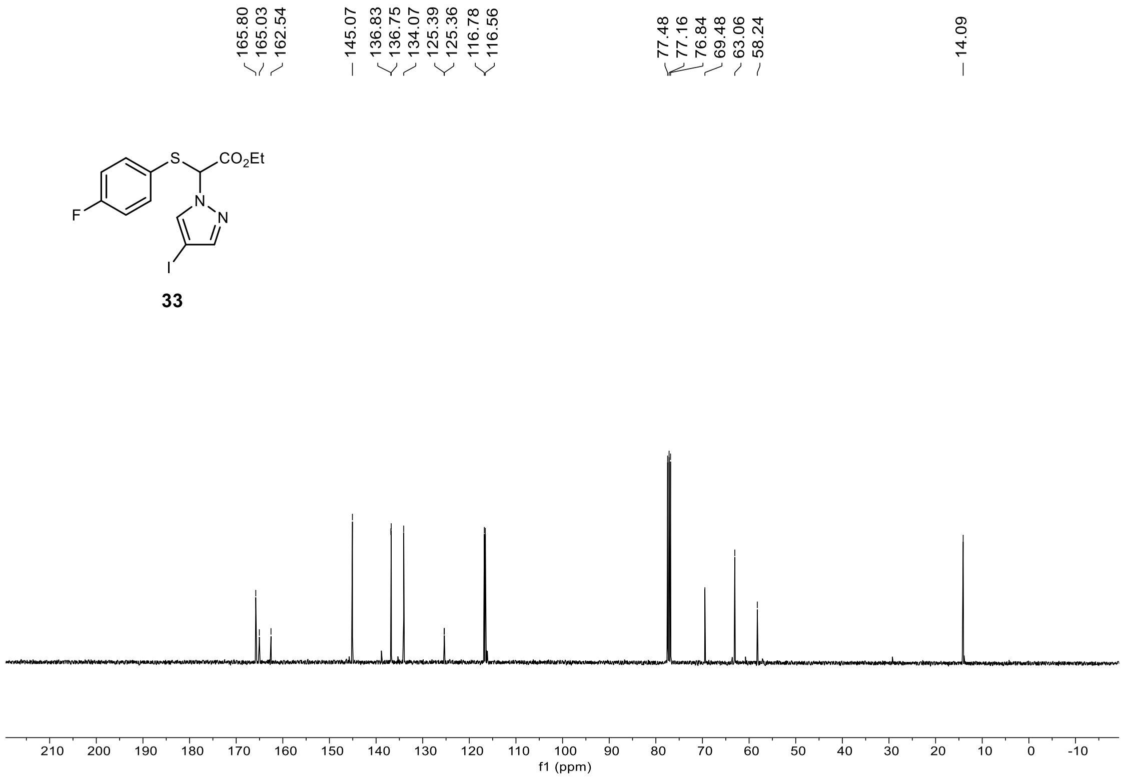

Supplementary Figure 91. ^{13}**C NMR** spectrum of compound **33** (101 MHz, r.t., $CDCl_3$)

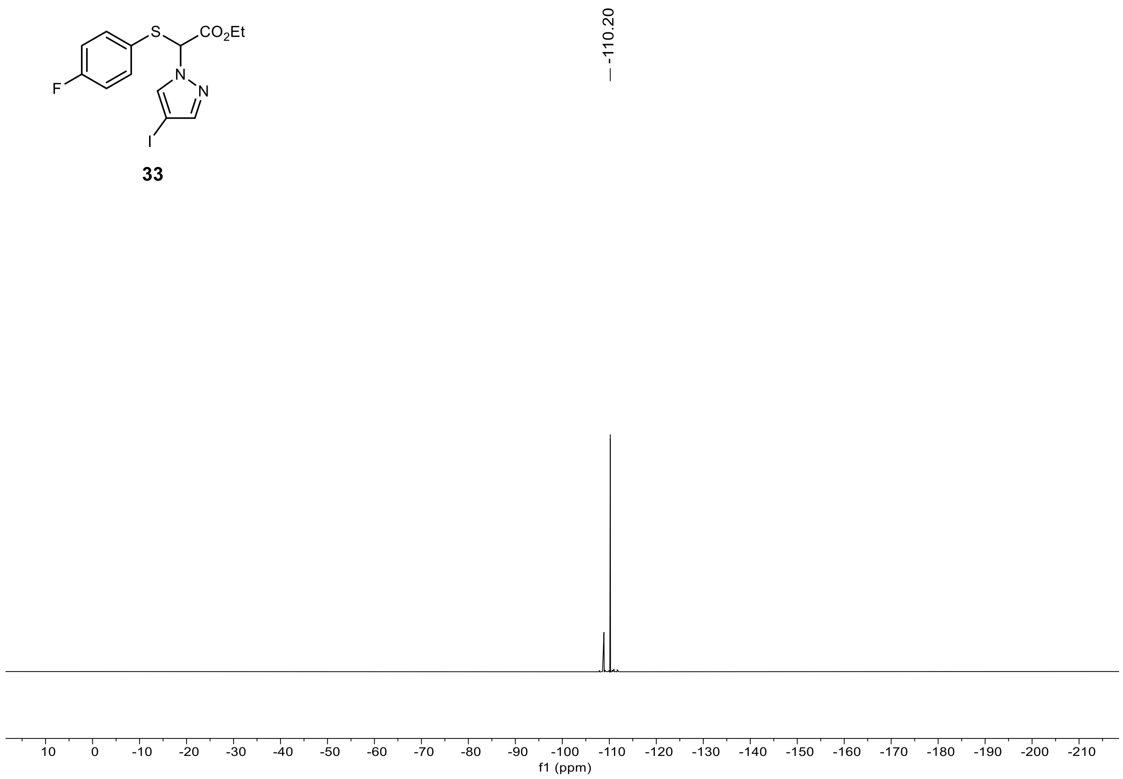

Supplementary Figure 92. ^{19}**F NMR** spectrum of compound **33** (377 MHz, r.t., $CDCl_3$)

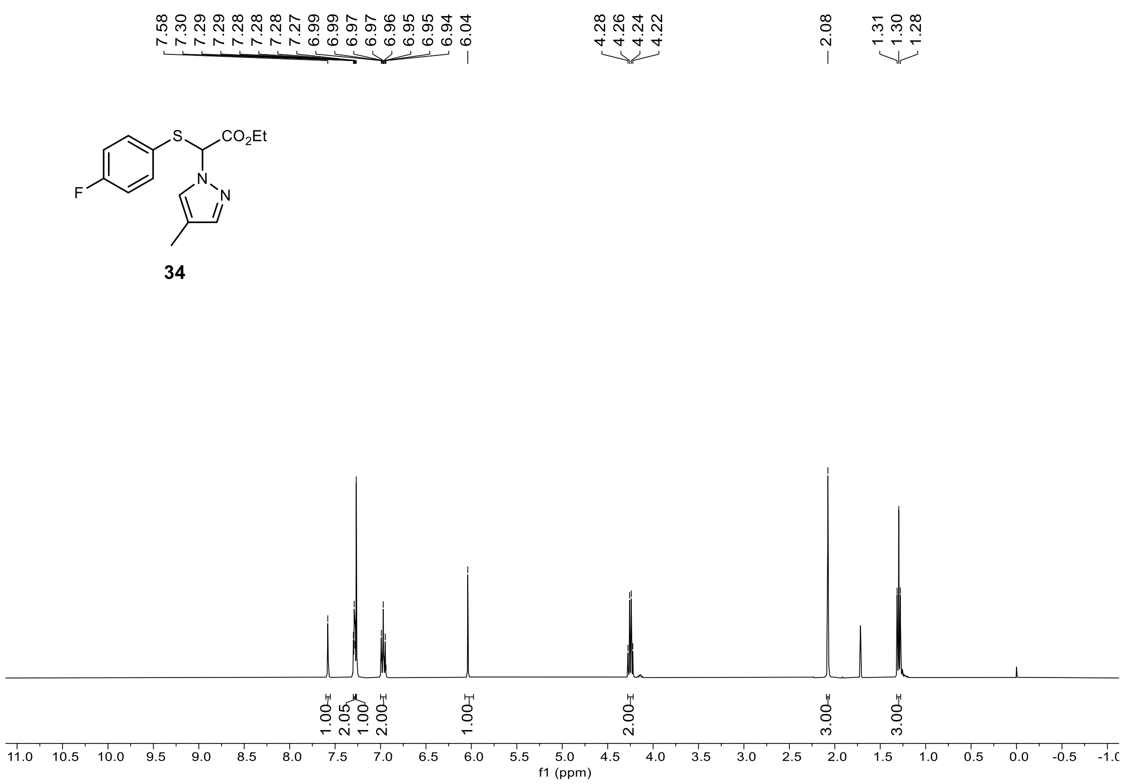

Supplementary Figure 93. ^{1}**H NMR** spectrum of compound **34** (400 MHz, r.t., $CDCl_3$)

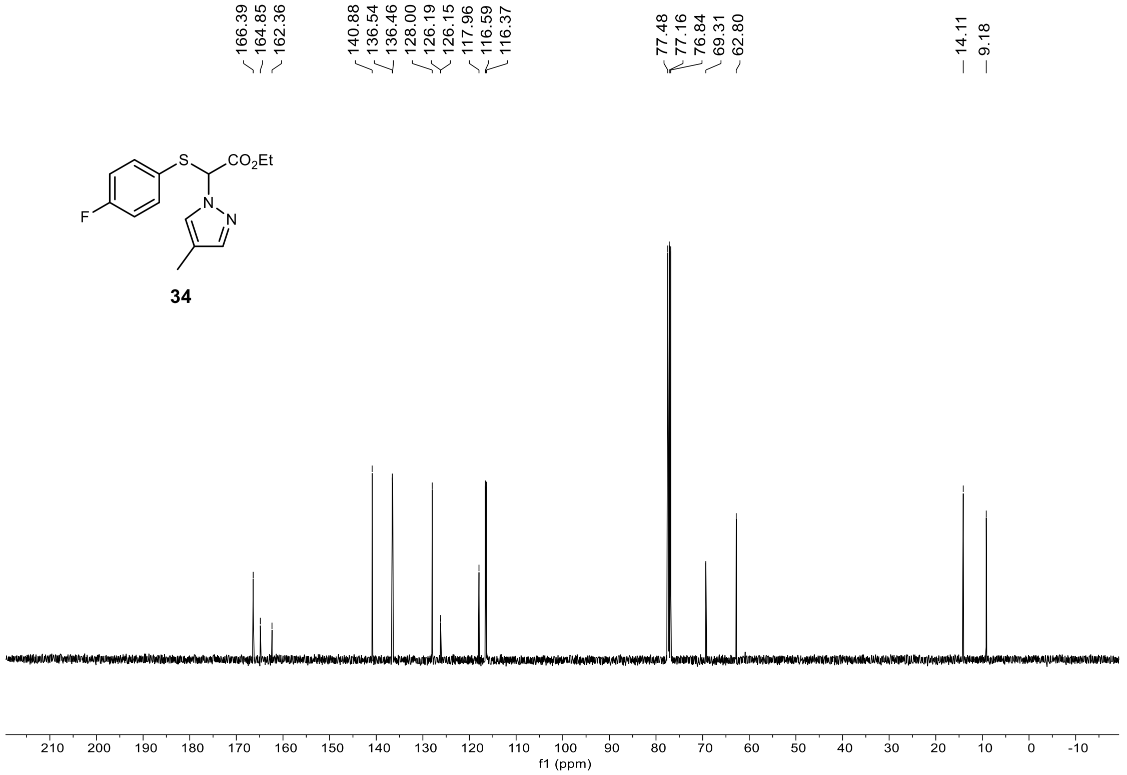

Supplementary Figure 94. ^{13}**C NMR** spectrum of compound **34** (101 MHz, r.t., $CDCl_3$)

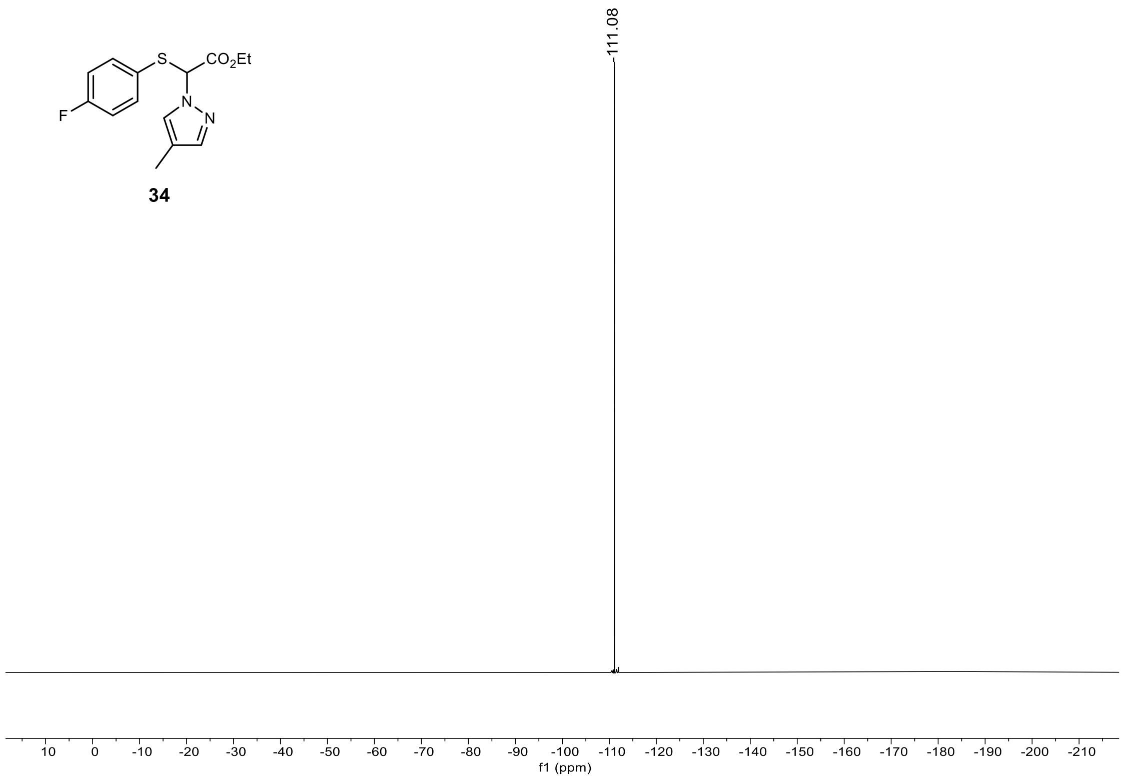

Supplementary Figure 95. ^{19}**F NMR** spectrum of compound **34** (377 MHz, r.t., $CDCl_3$)

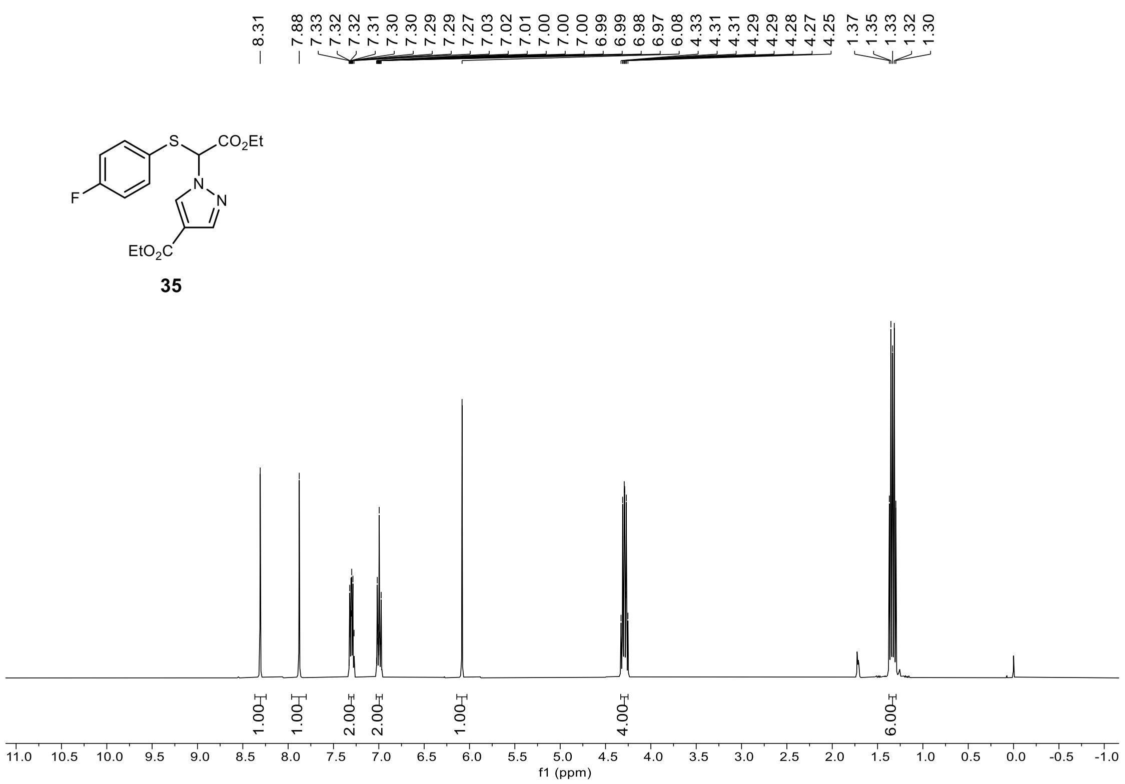

Supplementary Figure 96. ^{1}**H NMR** spectrum of compound **35** (400 MHz, r.t., $CDCl_3$)

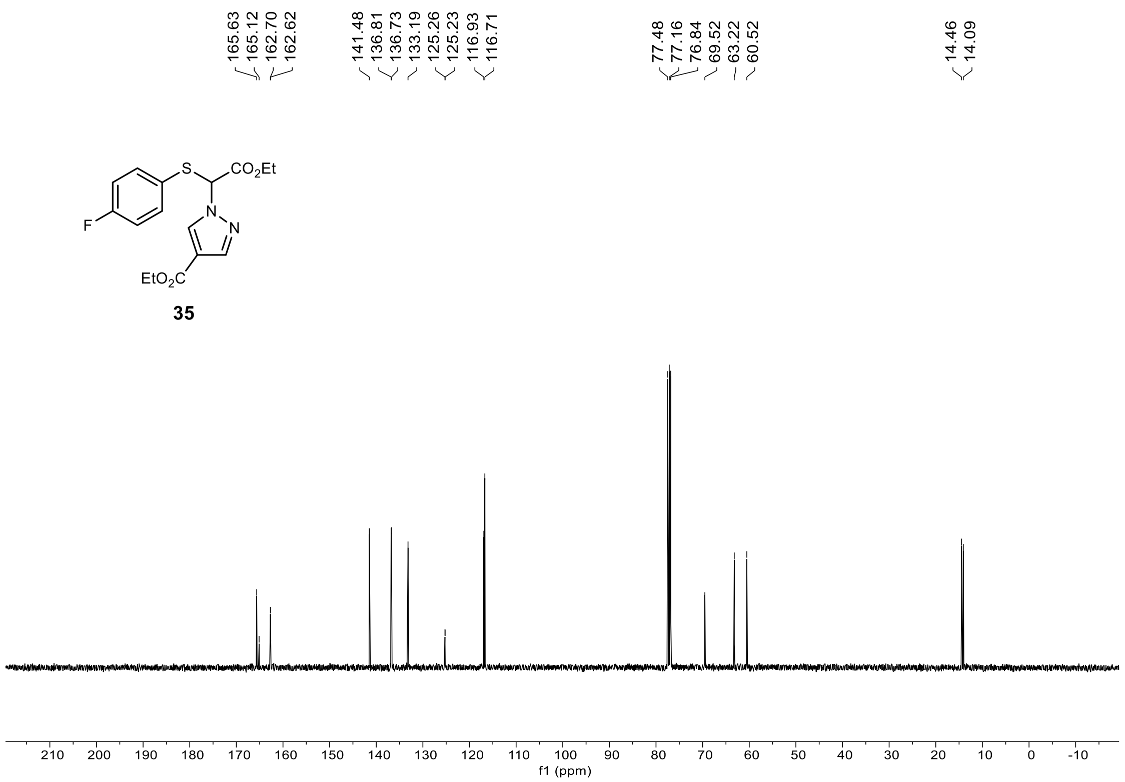

Supplementary Figure 97. ^{13}**C NMR** spectrum of compound **35** (101 MHz, r.t., $CDCl_3$)

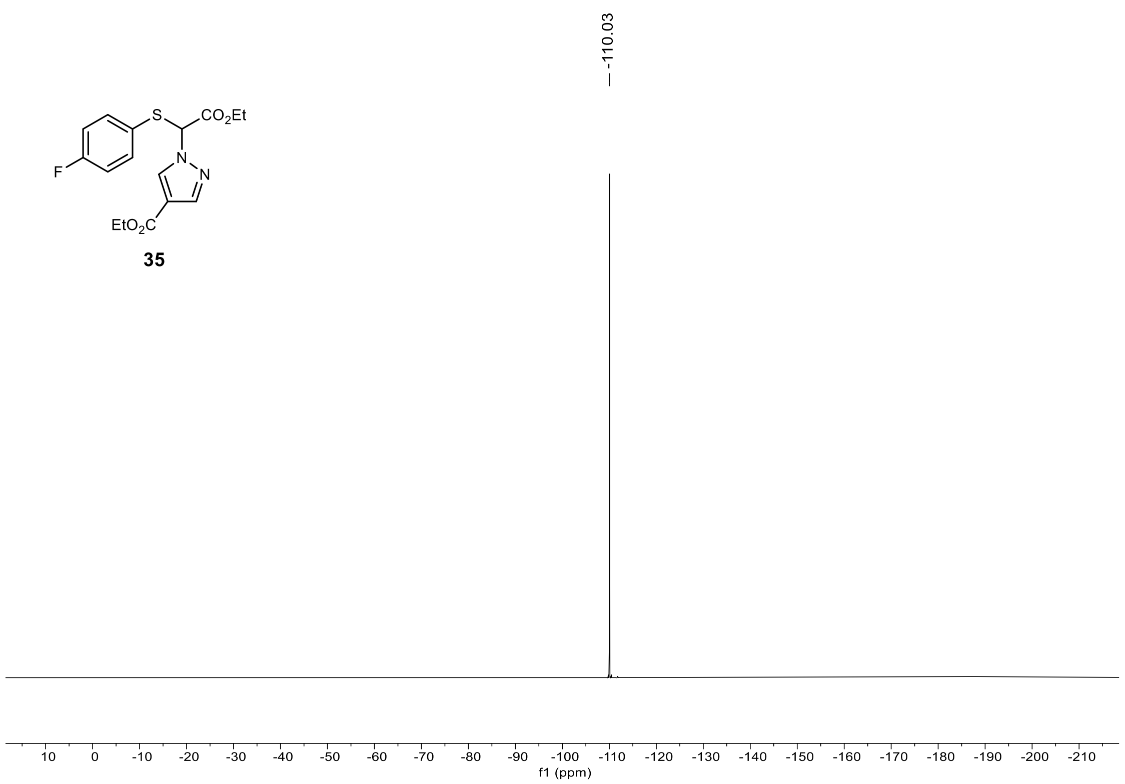

Supplementary Figure 98. **^{19}F NMR** spectrum of compound **35** (377 MHz, r.t., $CDCl_3$)

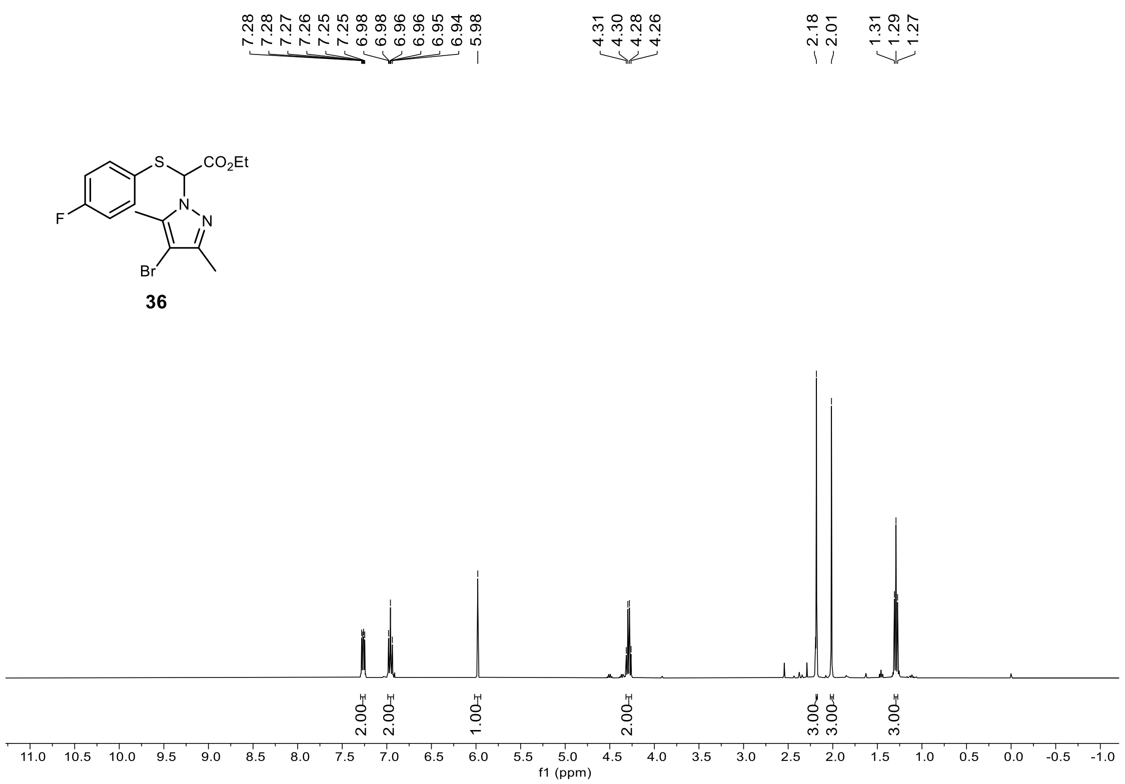

Supplementary Figure 99. **^{1}H NMR** spectrum of compound **36** (400 MHz, r.t., $CDCl_3$)

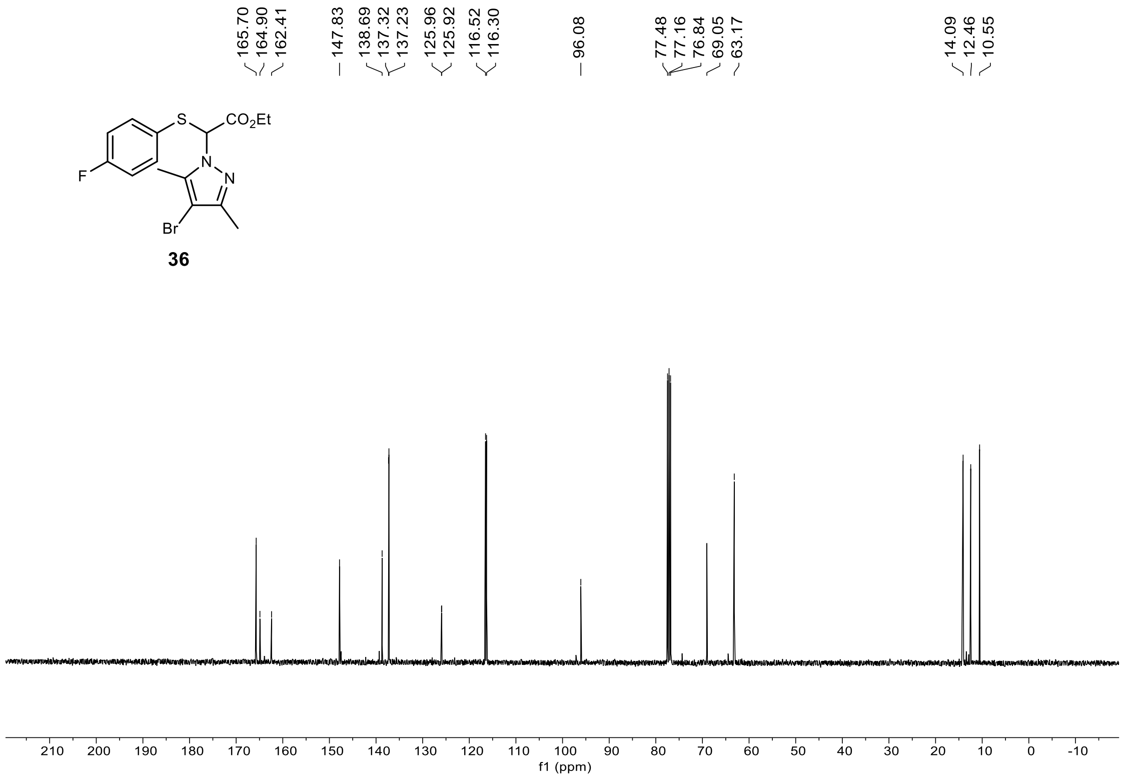

Supplementary Figure 100. **^{13}C NMR** spectrum of compound **36** (101 MHz, r.t., $CDCl_3$)

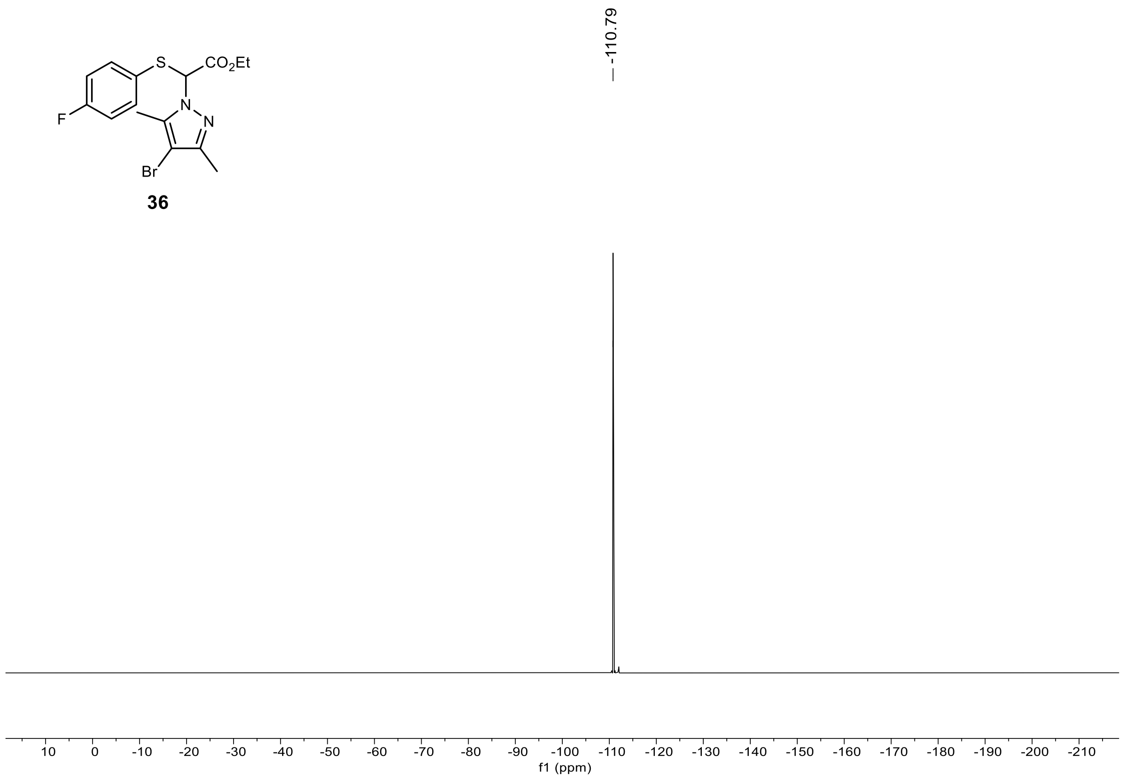

Supplementary Figure 101. ^{19}**F NMR** spectrum of compound **36** (377 MHz, r.t., $CDCl_3$)

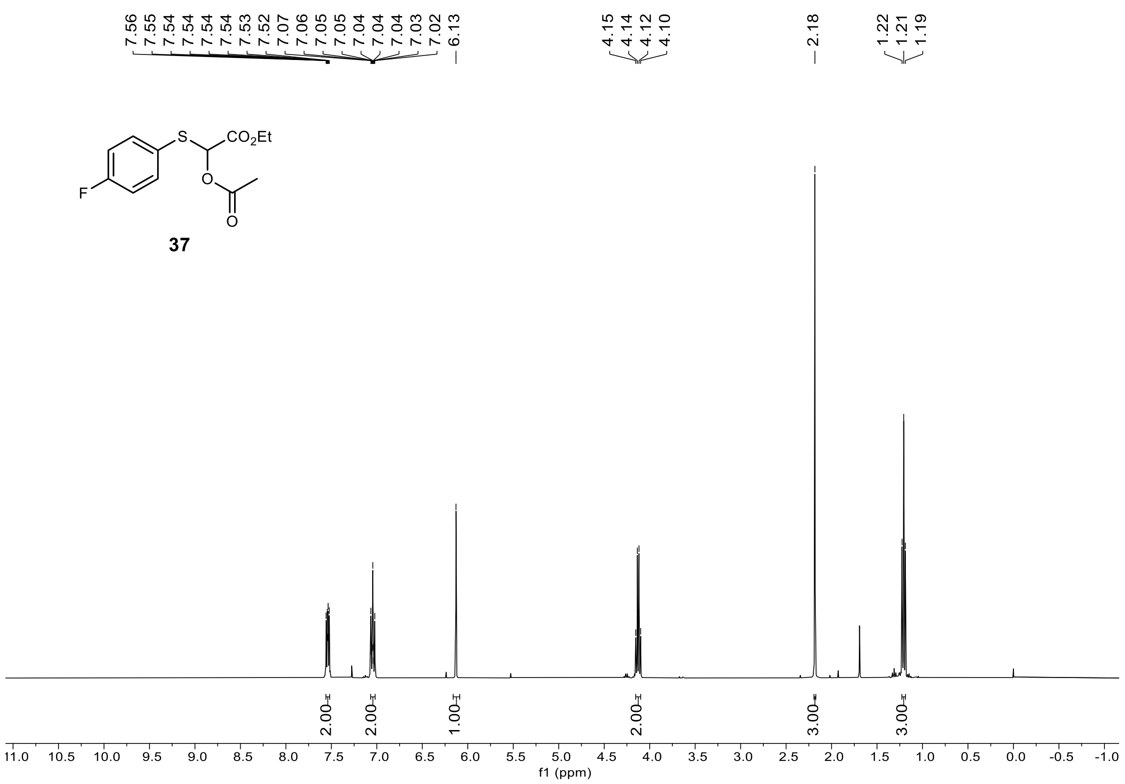

Supplementary Figure 102. **^{1}H NMR** spectrum of compound **37** (400 MHz, r.t., $CDCl_3$)

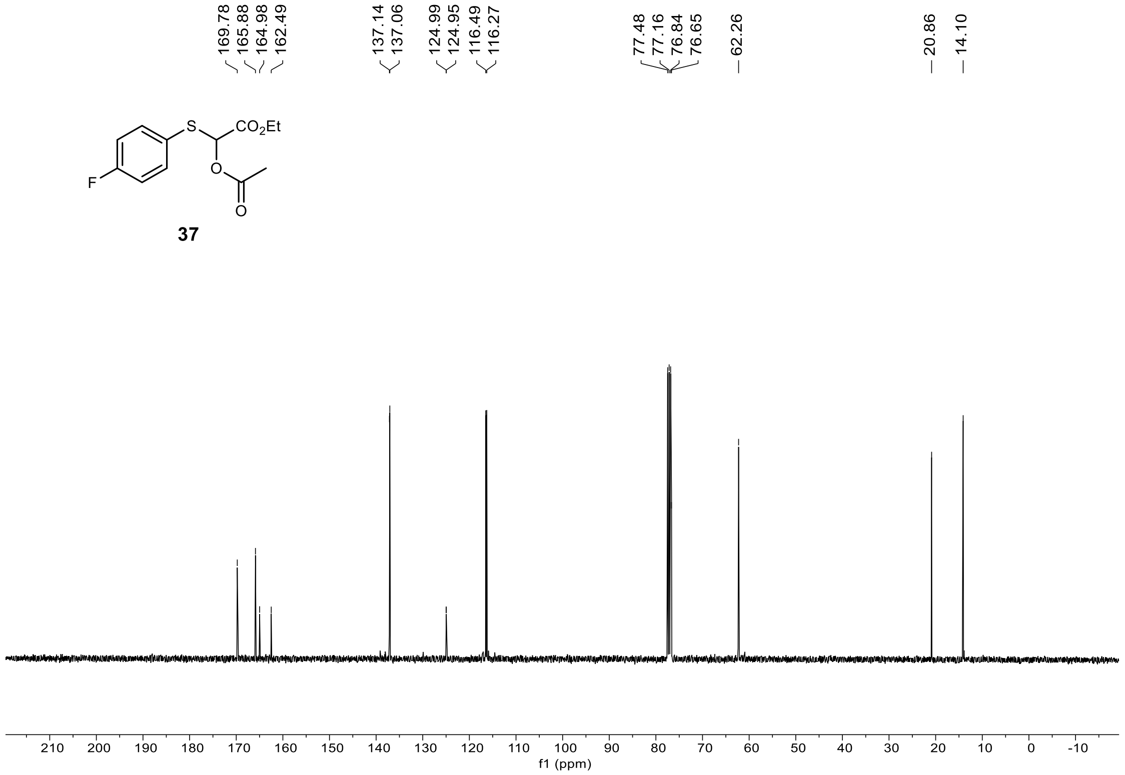

Supplementary Figure 103. **^{13}C NMR** spectrum of compound **37** (101 MHz, r.t., $CDCl_3$)

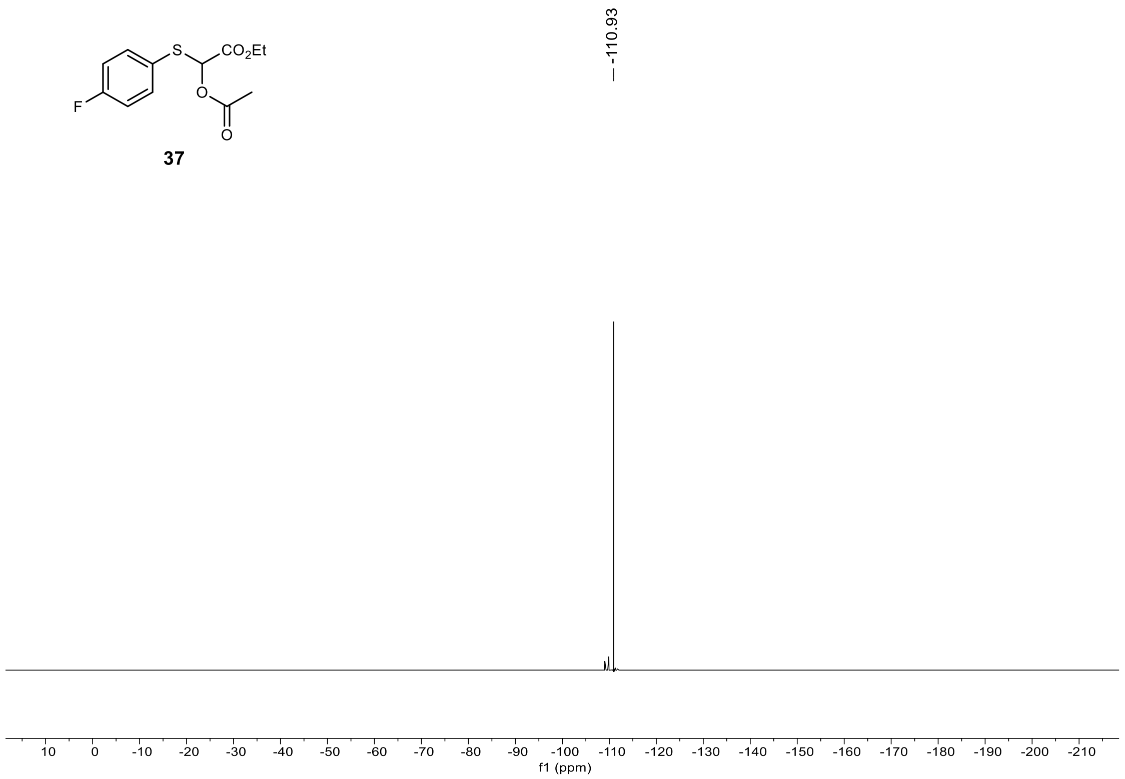

Supplementary Figure 104. **^{19}F NMR** spectrum of compound **37** (377 MHz, r.t., $CDCl_3$)

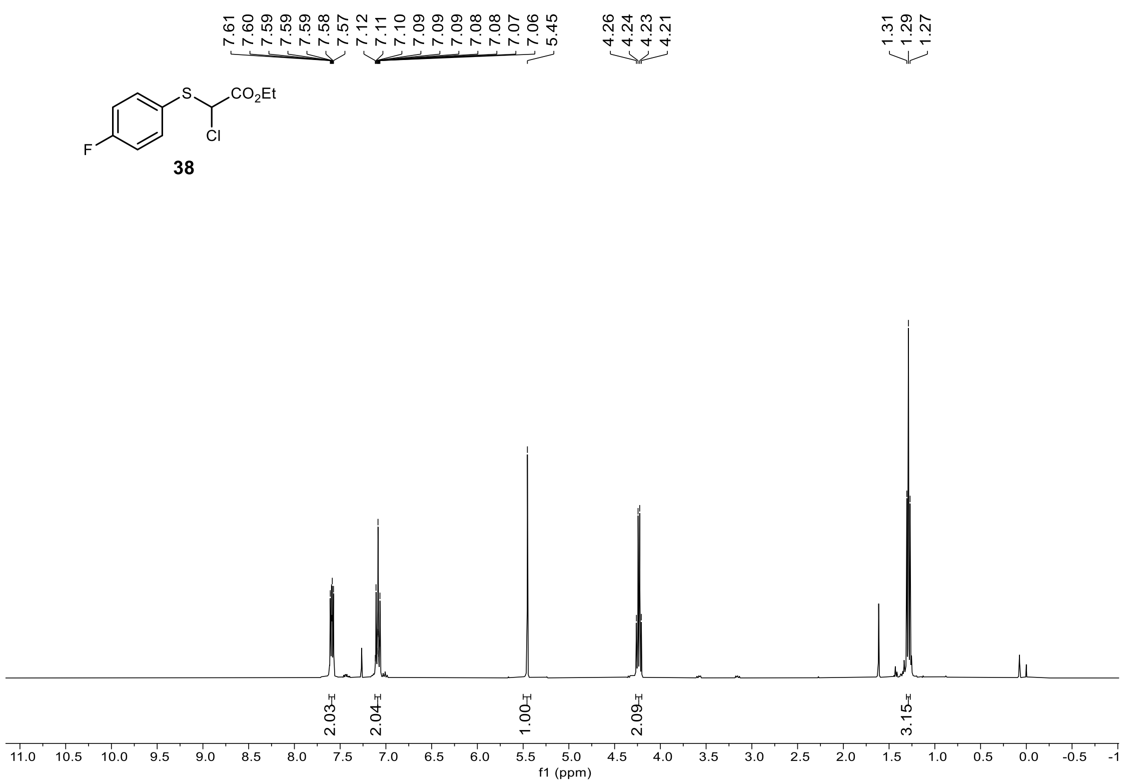

Supplementary Figure 105. ^{1}**H NMR** spectrum of compound **38** (400 MHz, r.t., $CDCl_3$)

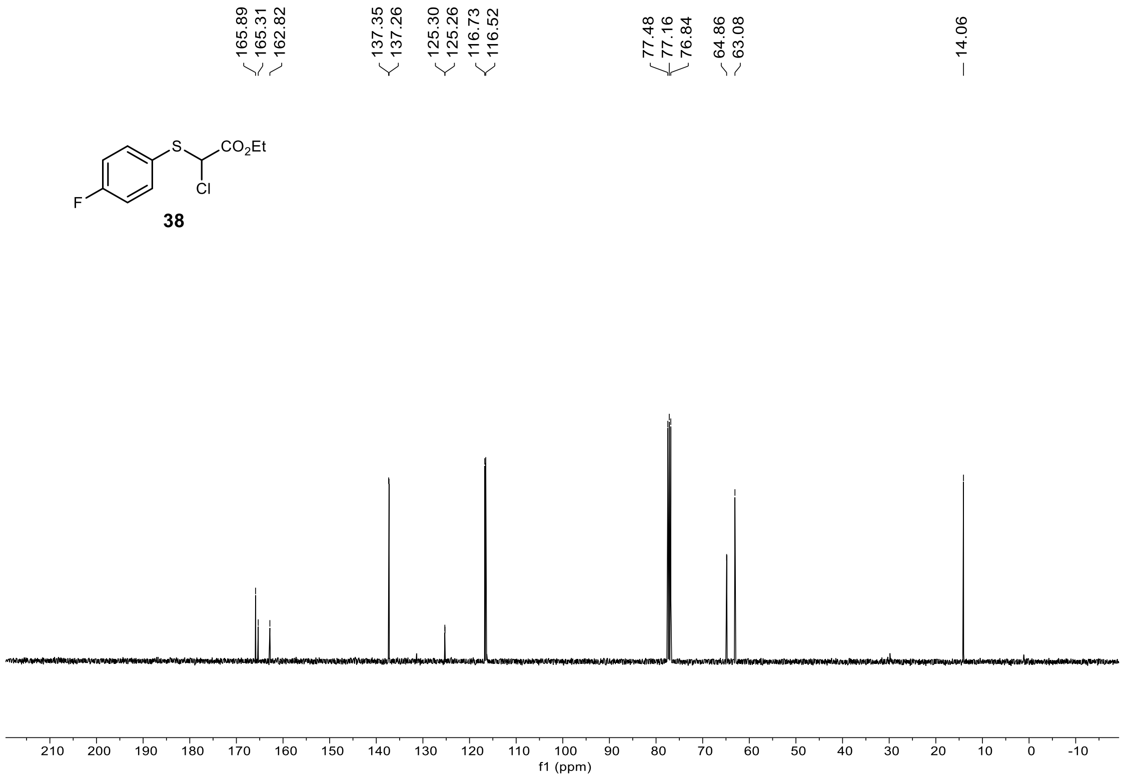

Supplementary Figure 106. ^{13}**C NMR** spectrum of compound **38** (101 MHz, r.t., $CDCl_3$)

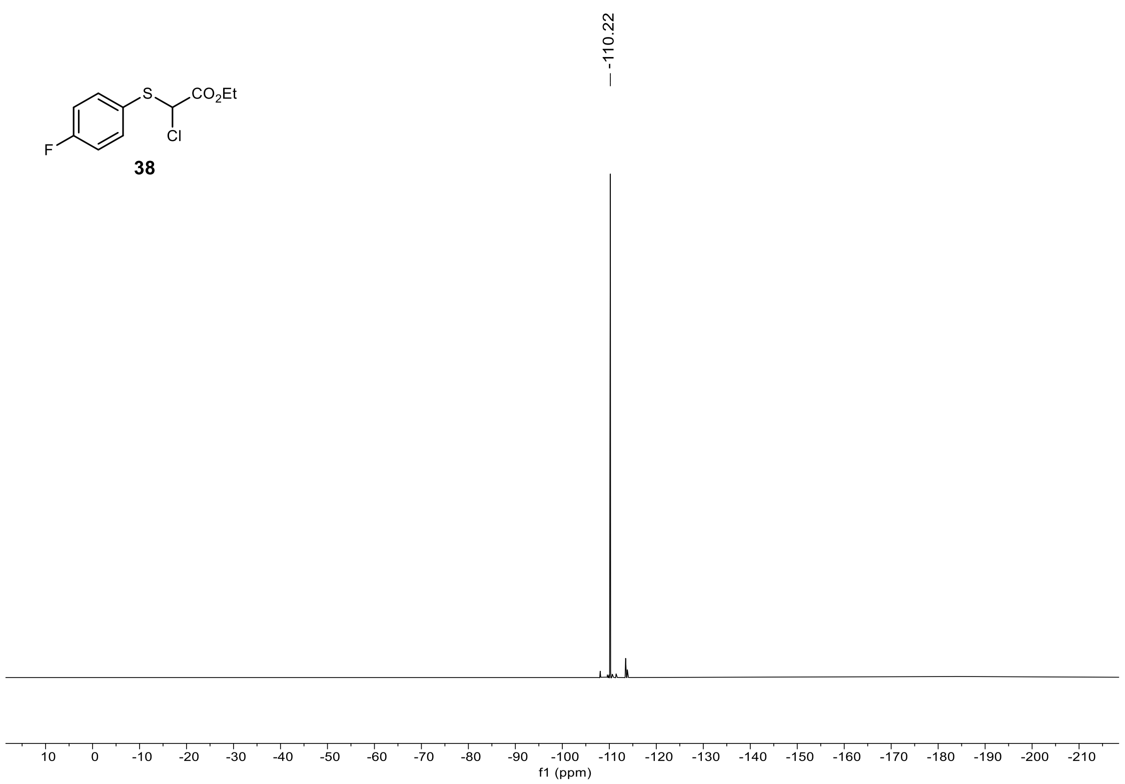

Supplementary Figure 107. ^{19}**F NMR** spectrum of compound **38** (377 MHz, r.t., $CDCl_3$)

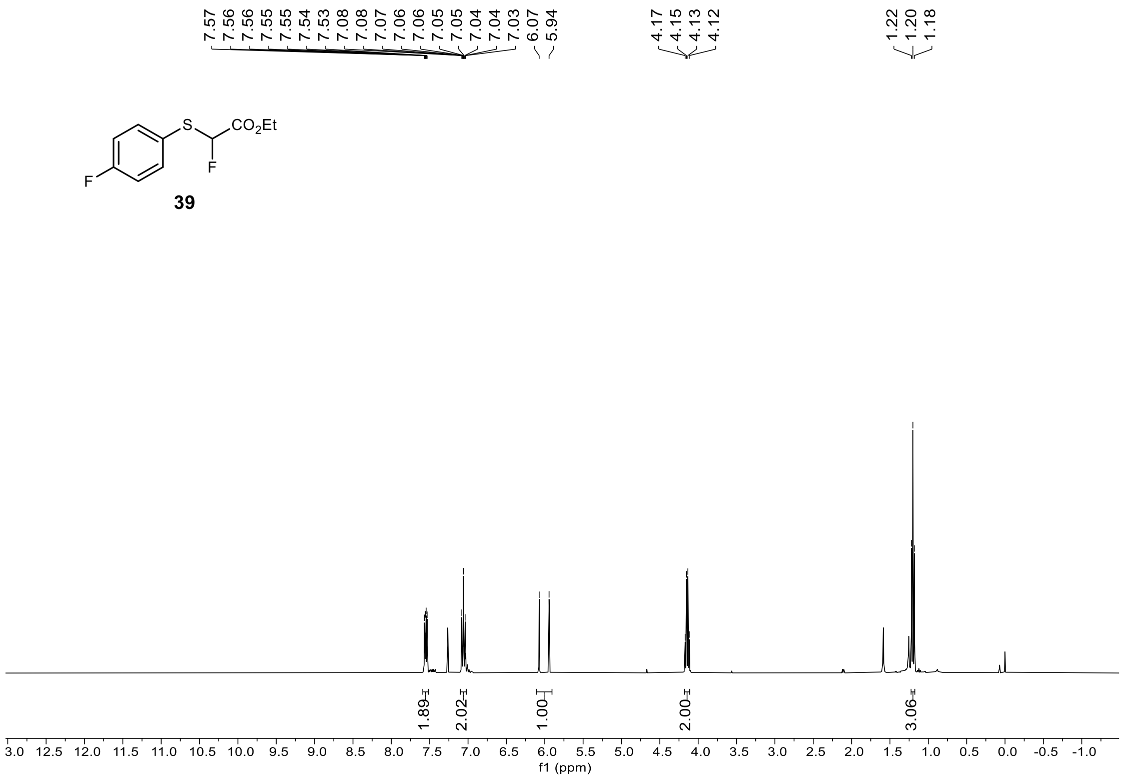

Supplementary Figure 108. ^{1}**H NMR** spectrum of compound **39** (400 MHz, r.t., $CDCl_3$)

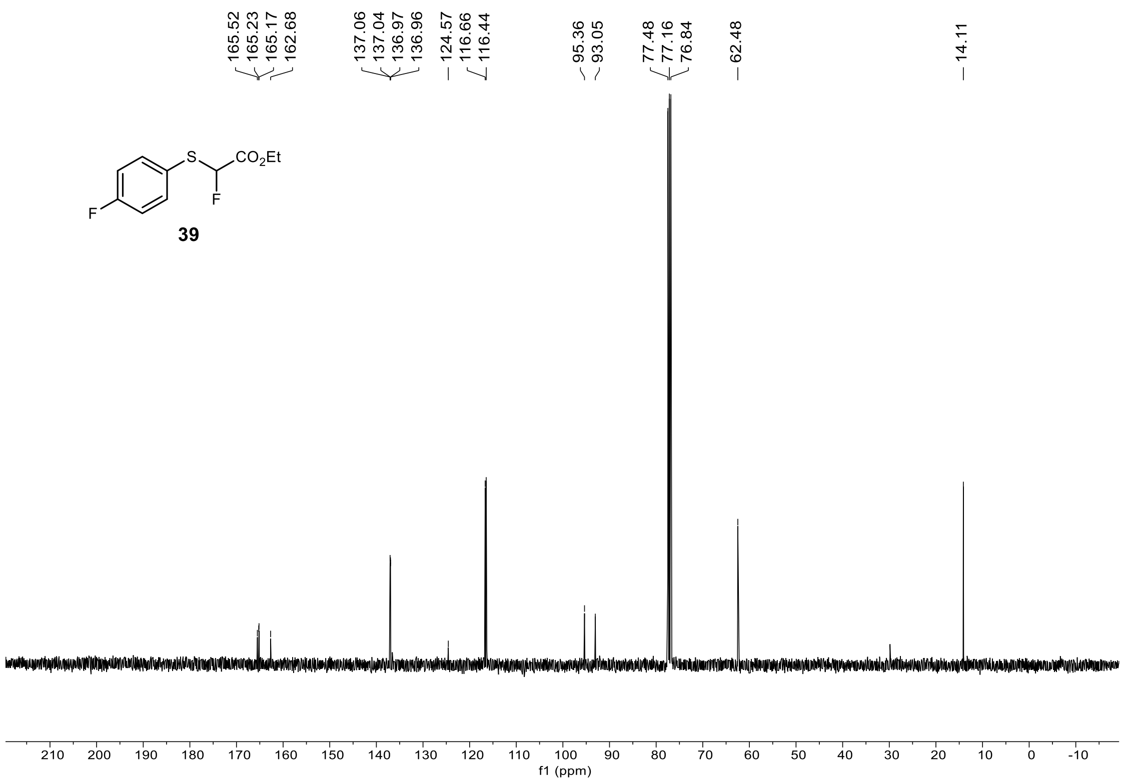

Supplementary Figure 109. ^{13}**C NMR** spectrum of compound **39** (101 MHz, r.t., $CDCl_3$)

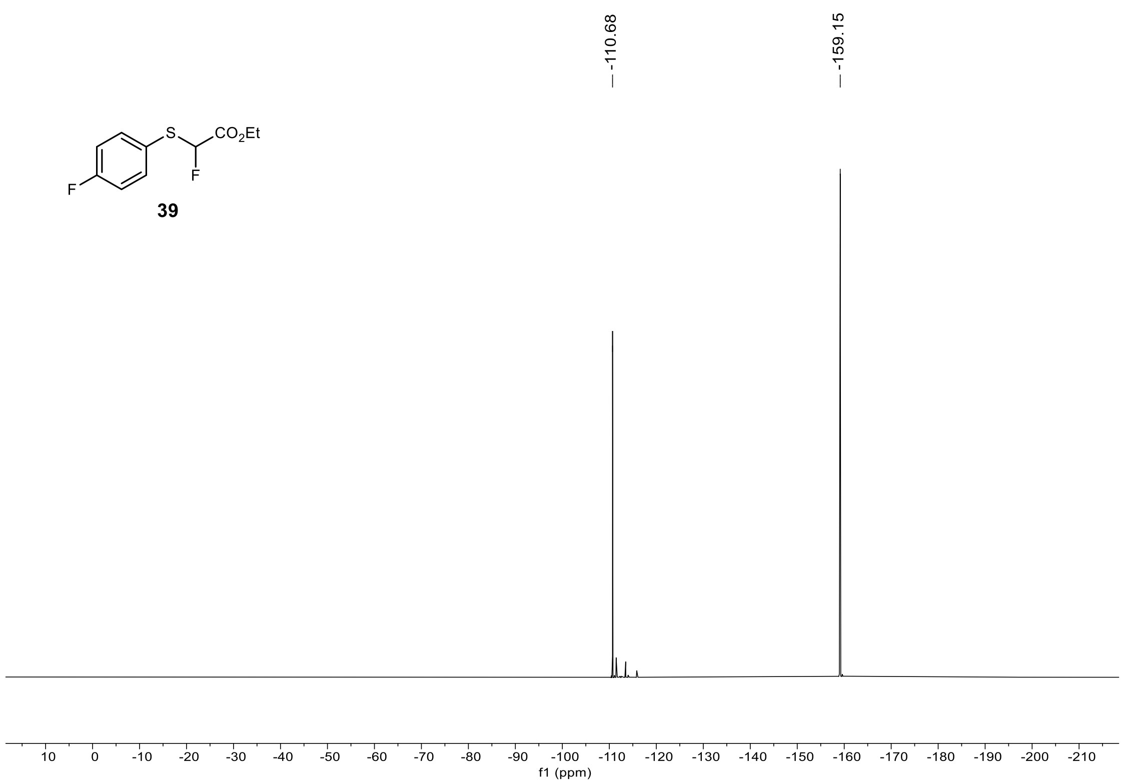

Supplementary Figure 110. ^{19}**F NMR** spectrum of compound **39** (377 MHz, r.t., $CDCl_3$)

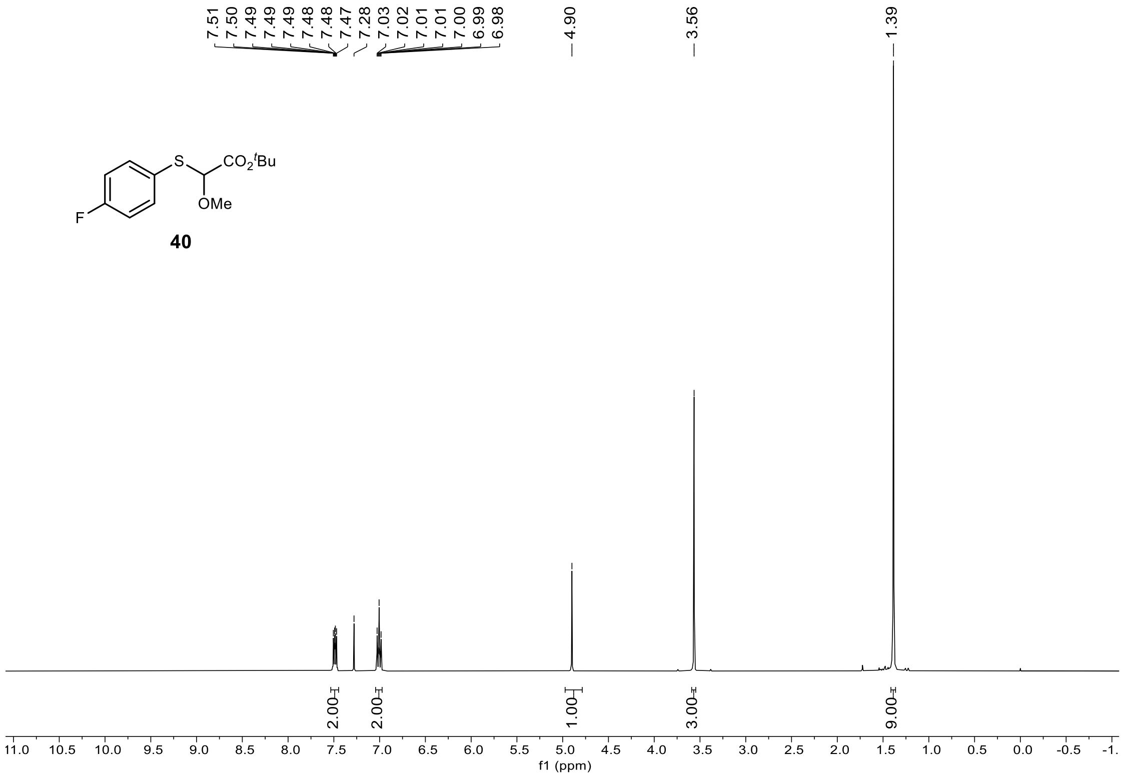

Supplementary Figure 111. ^{1}**H NMR** spectrum of compound **40** (400 MHz, r.t., $CDCl_3$)

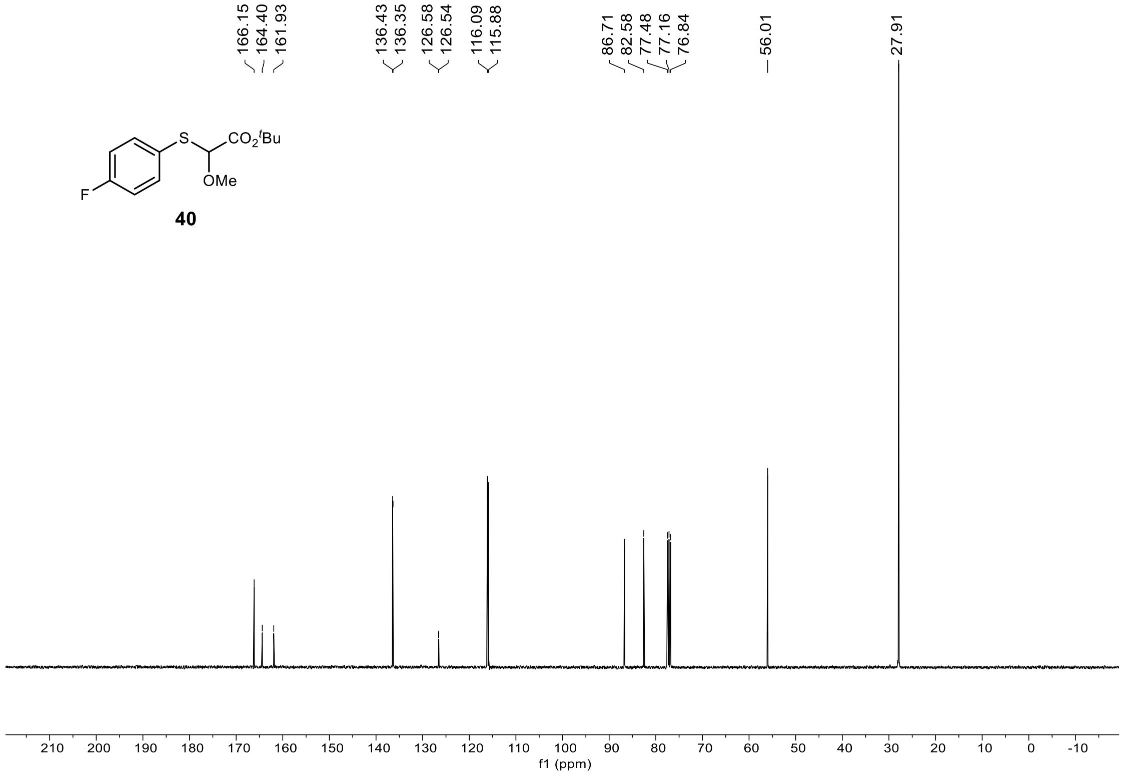

Supplementary Figure 112. ^{13}**C NMR** spectrum of compound **40** (101 MHz, r.t., $CDCl_3$)

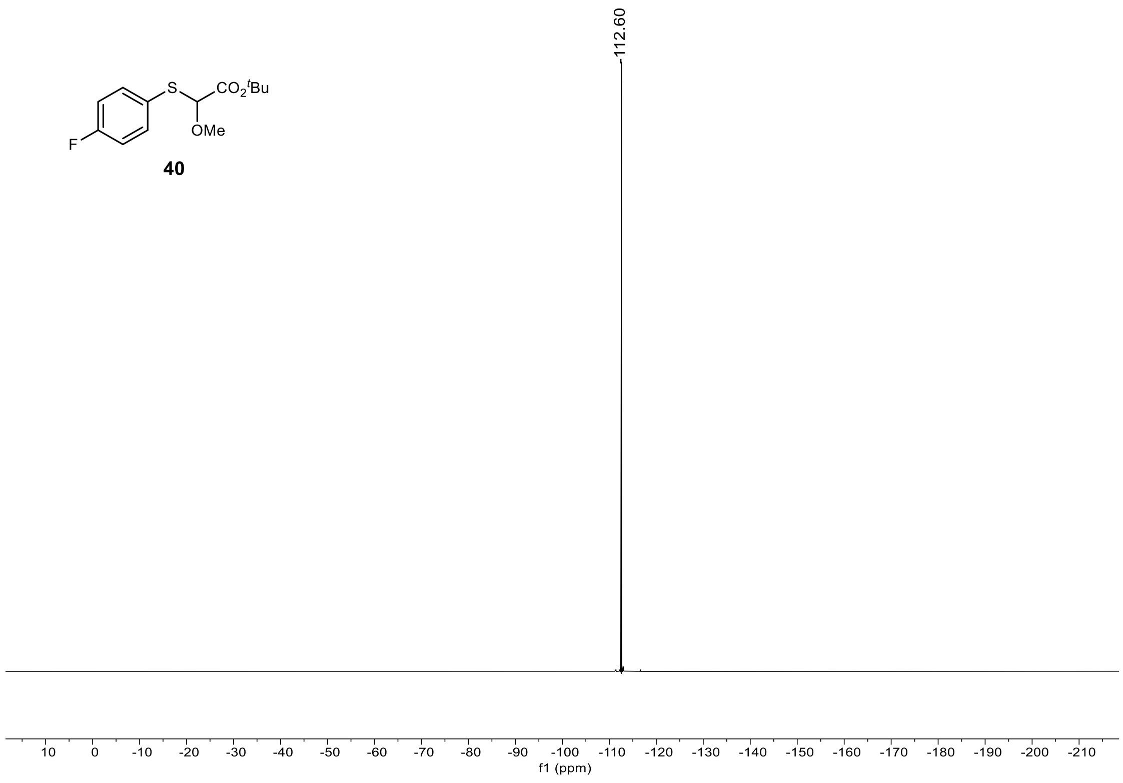

Supplementary Figure 113. ^{19}**F NMR** spectrum of compound **40** (377 MHz, r.t., $CDCl_3$)

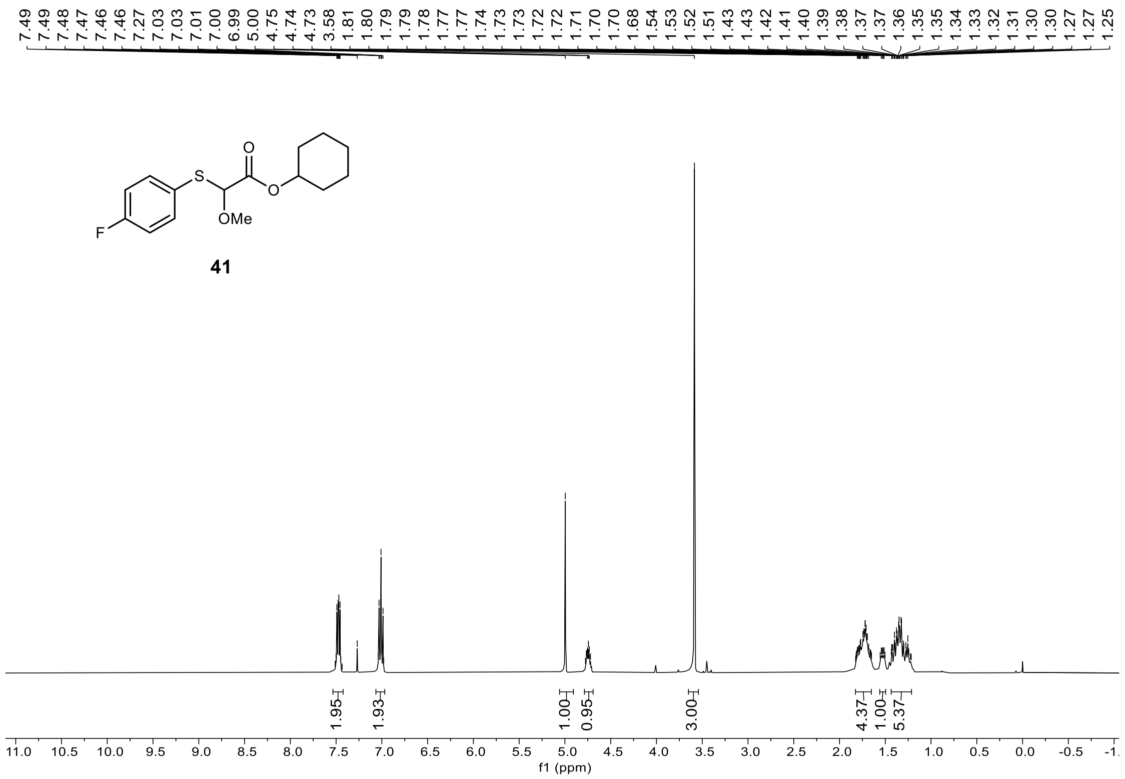

Supplementary Figure 114. ^{1}**H NMR** spectrum of compound **41** (400 MHz, r.t., $CDCl_3$)

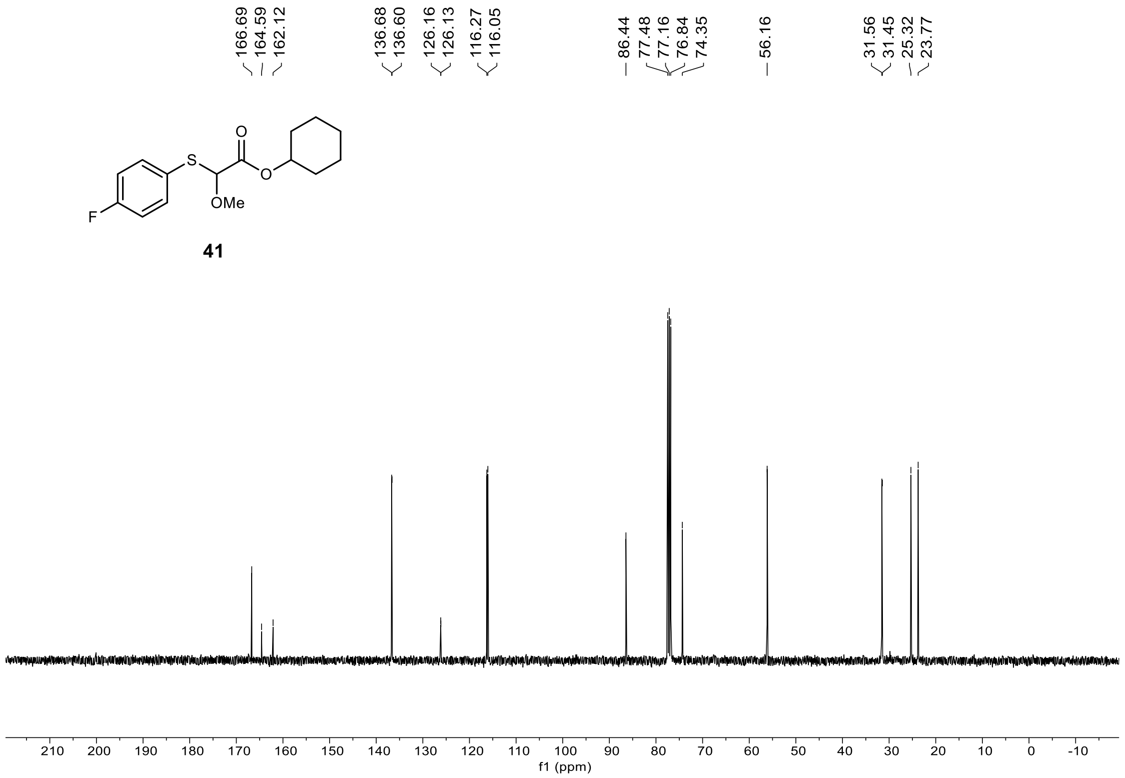

Supplementary Figure 115. ^{13}**C NMR** spectrum of compound **41** (101 MHz, r.t., $CDCl_3$)

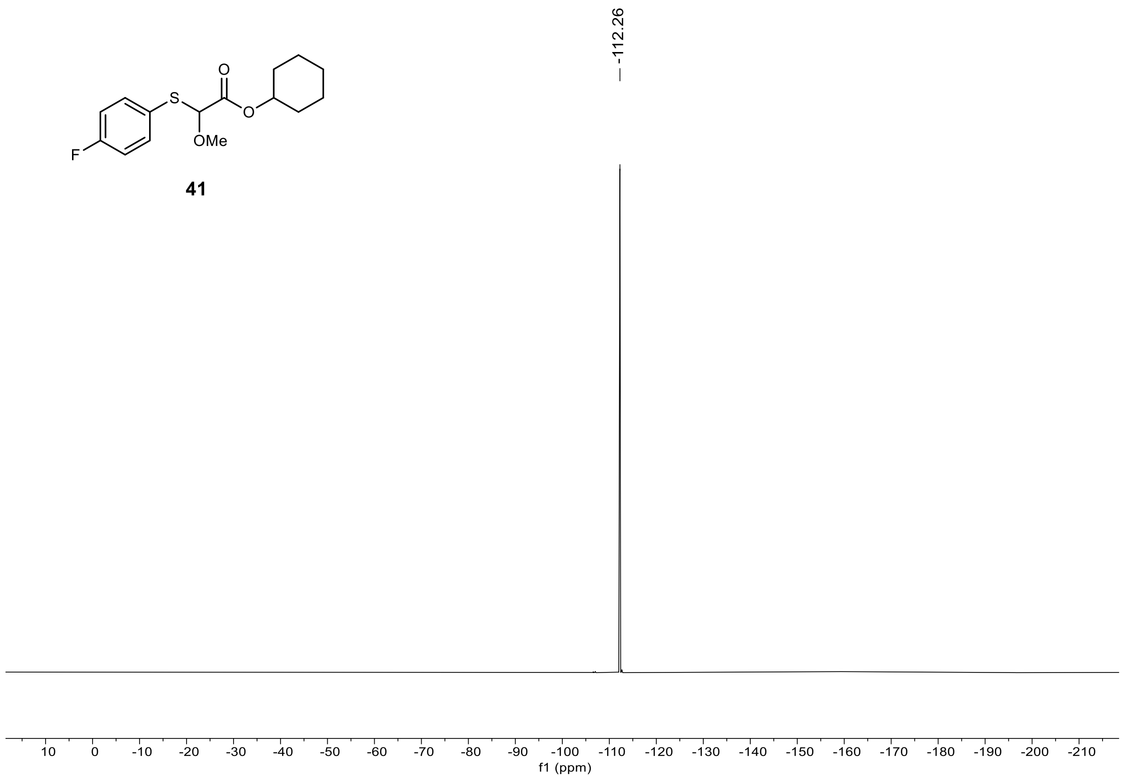

Supplementary Figure 116. ^{19}**F NMR** spectrum of compound **41** (377 MHz, r.t., $CDCl_3$)

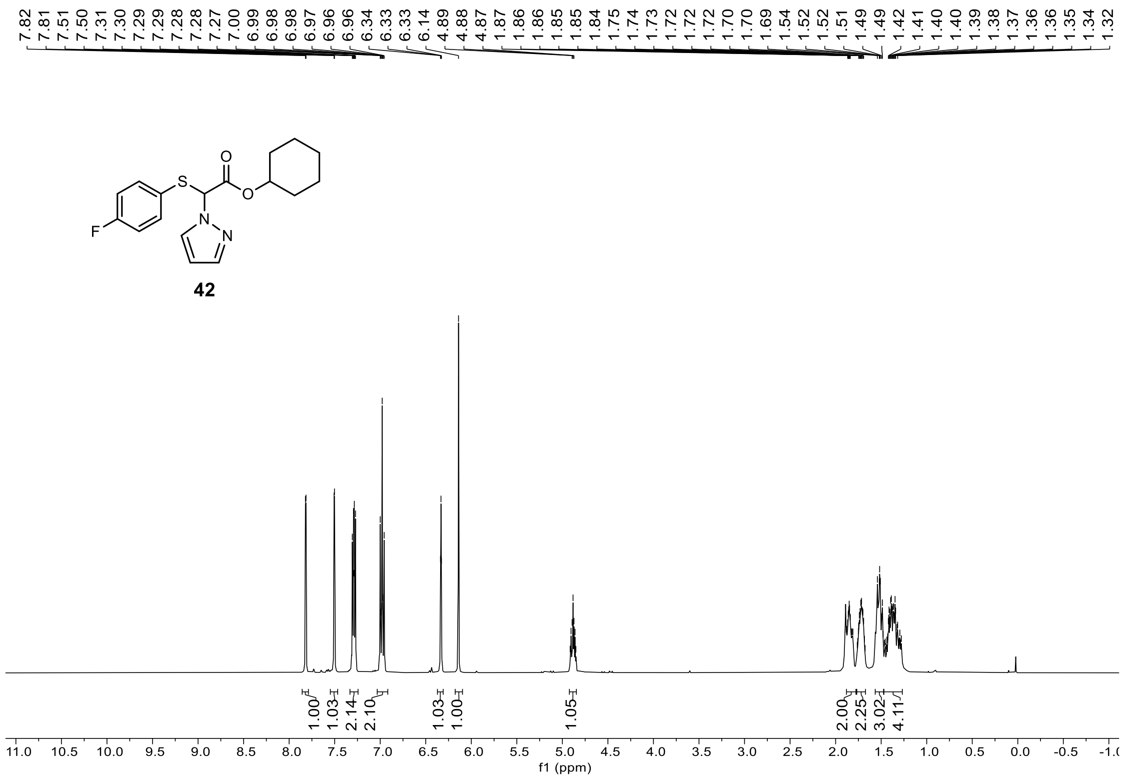

Supplementary Figure 117. ^{1}**H NMR** spectrum of compound **42** (400 MHz, r.t., $CDCl_3$)

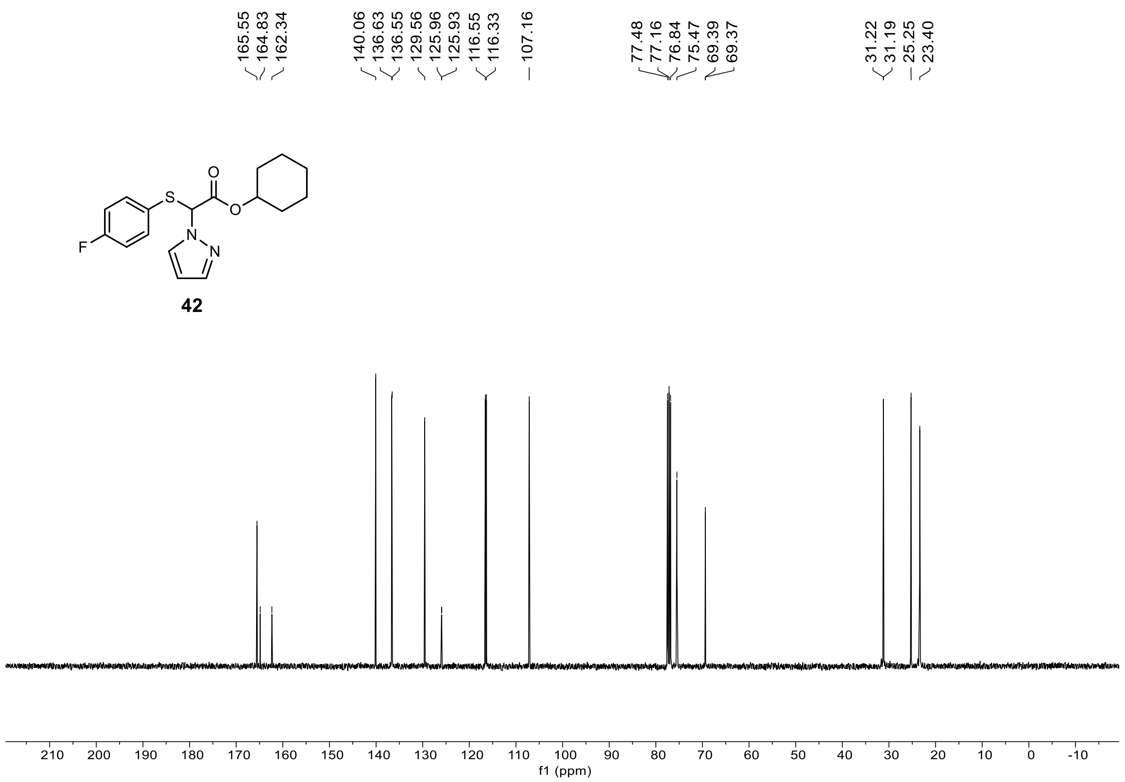

Supplementary Figure 118. ^{13}**C NMR** spectrum of compound **42** (101 MHz, r.t., $CDCl_3$)

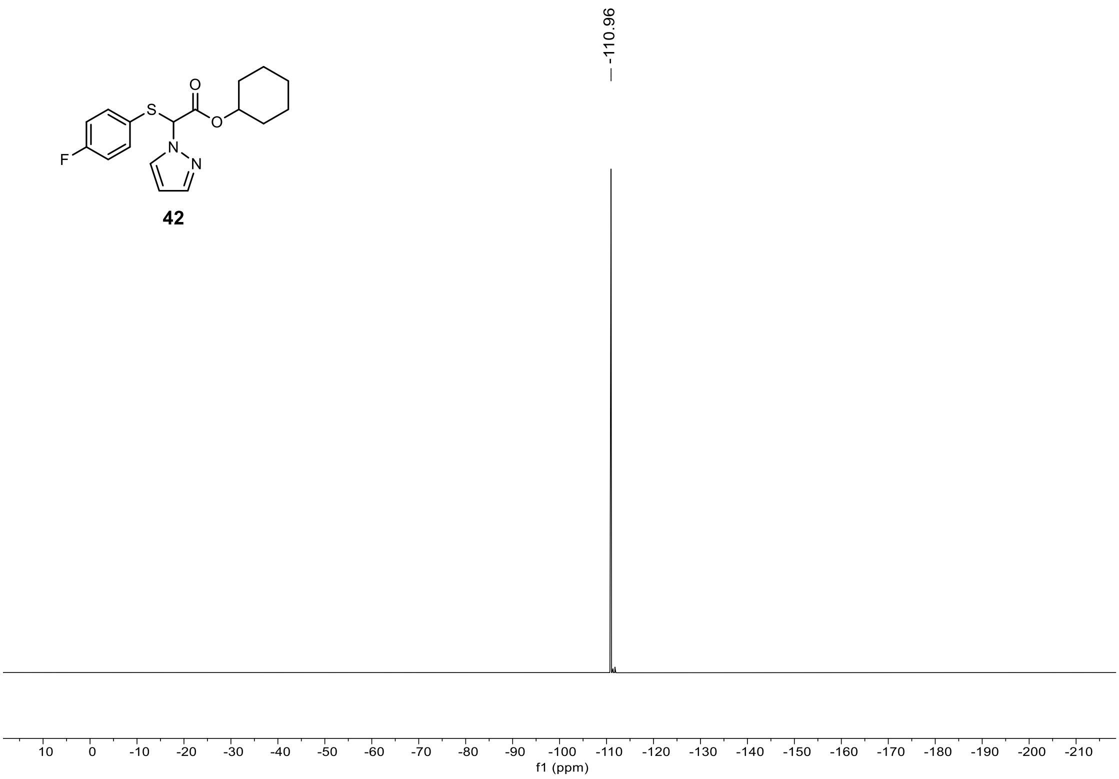

Supplementary Figure 119. ^{19}F NMR spectrum of compound **42** (377 MHz, r.t., $CDCl_3$)

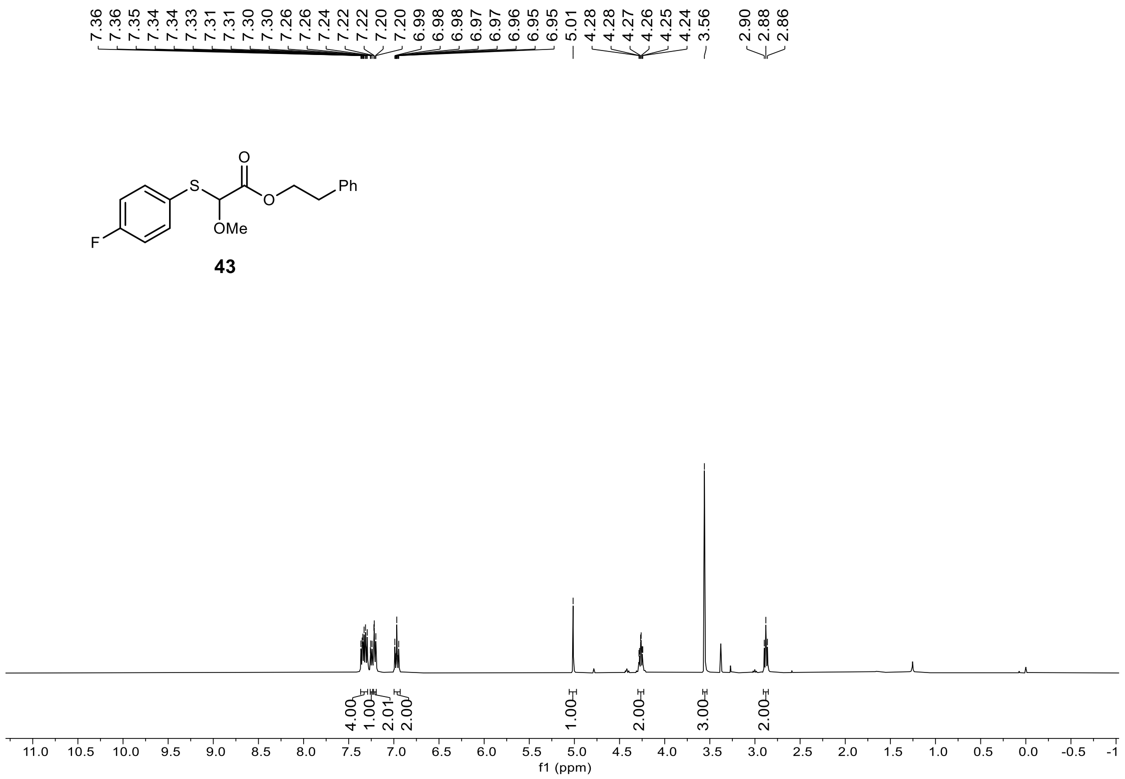

Supplementary Figure 120. ^{1}H NMR spectrum of compound **43** (400 MHz, r.t., $CDCl_3$)

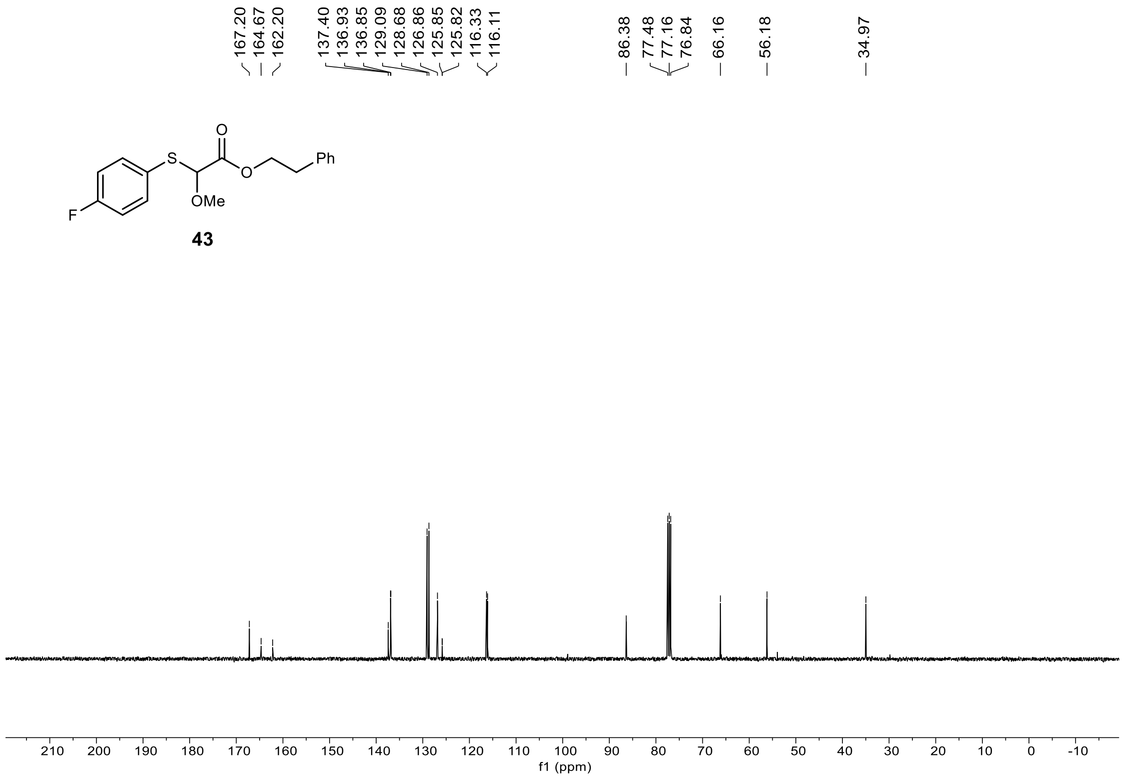

Supplementary Figure 121. ^{13}C NMR spectrum of compound **43** (101 MHz, r.t., $CDCl_3$)

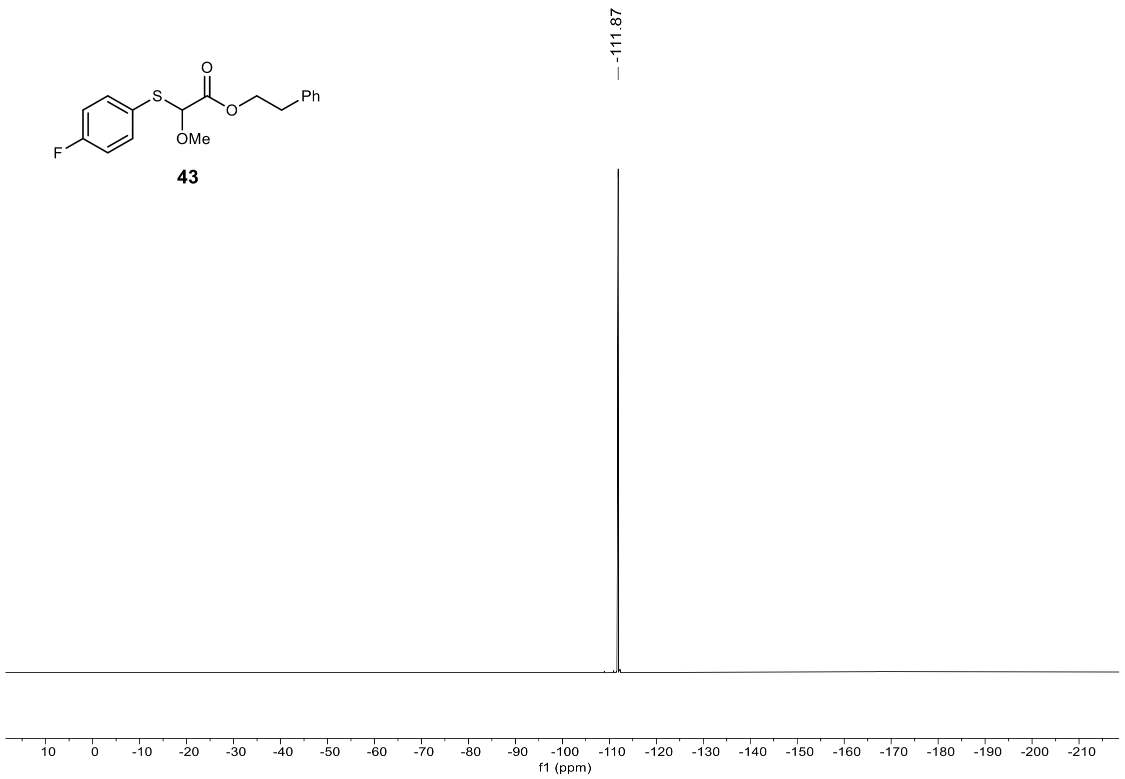

Supplementary Figure 122. **^{19}F NMR** spectrum of compound **43** (377 MHz, r.t., $CDCl_3$)

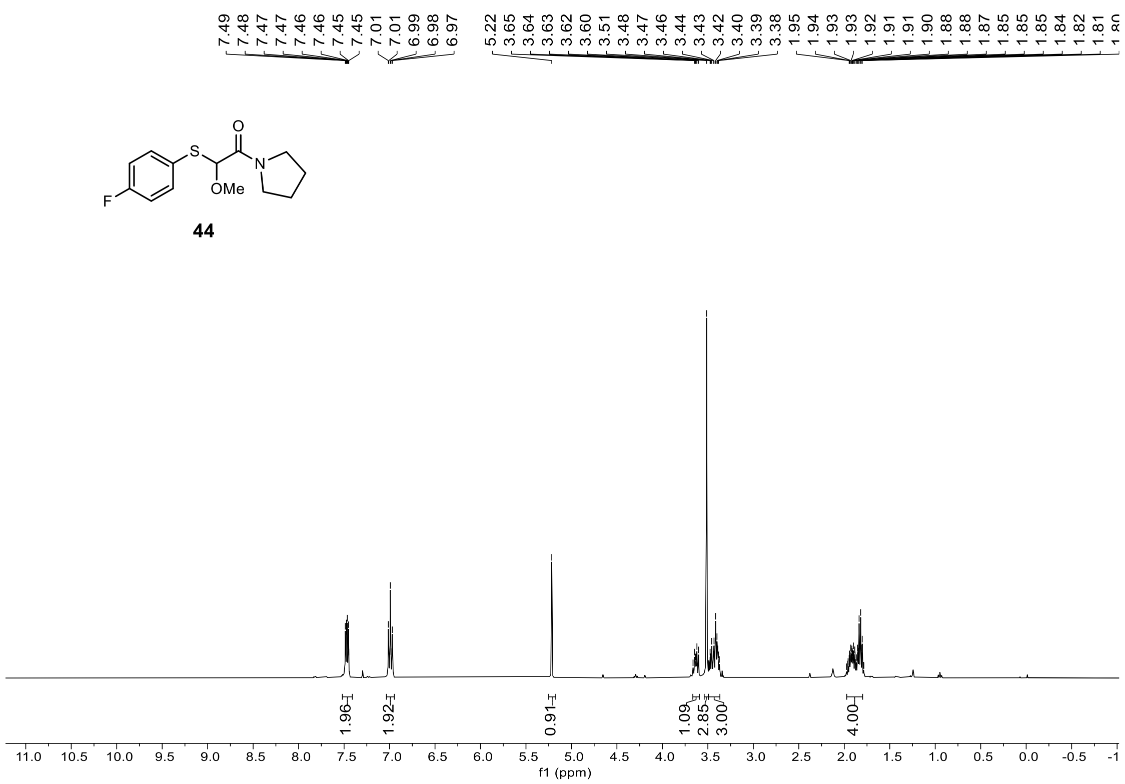

Supplementary Figure 123. ^{1}**H NMR** spectrum of compound **44** (400 MHz, r.t., $CDCl_3$)

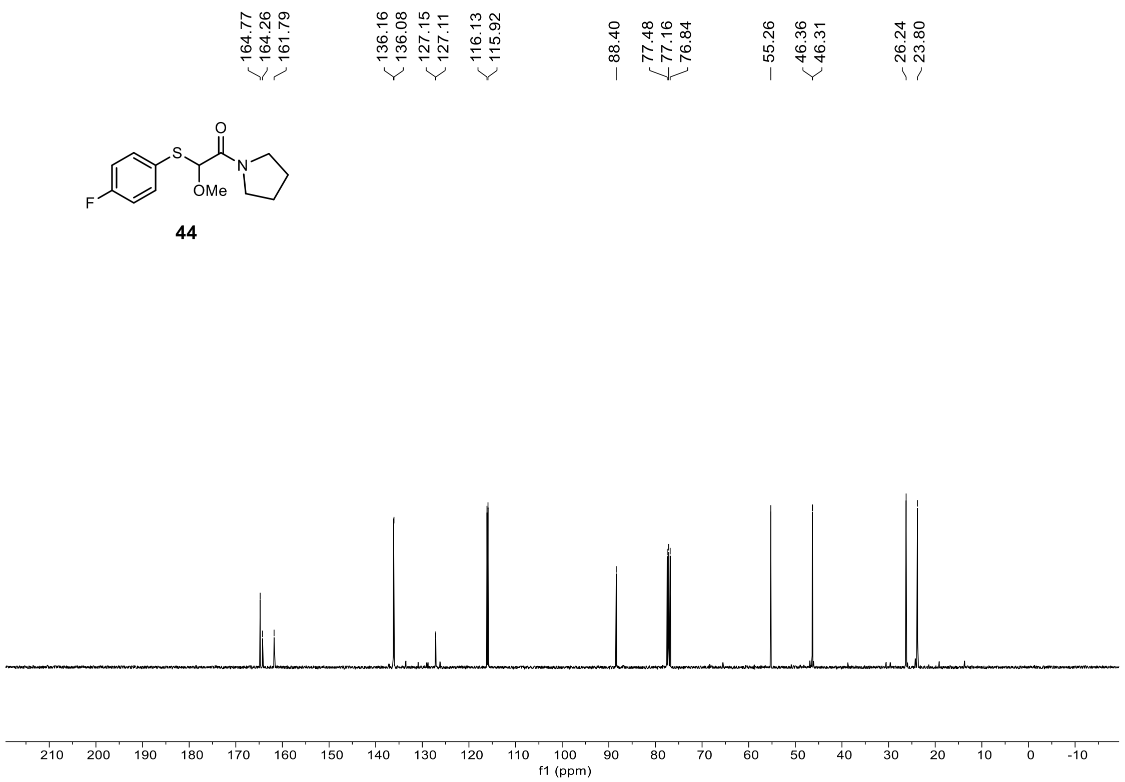

Supplementary Figure 124. ^{13}**C NMR** spectrum of compound **44** (101 MHz, r.t., $CDCl_3$)

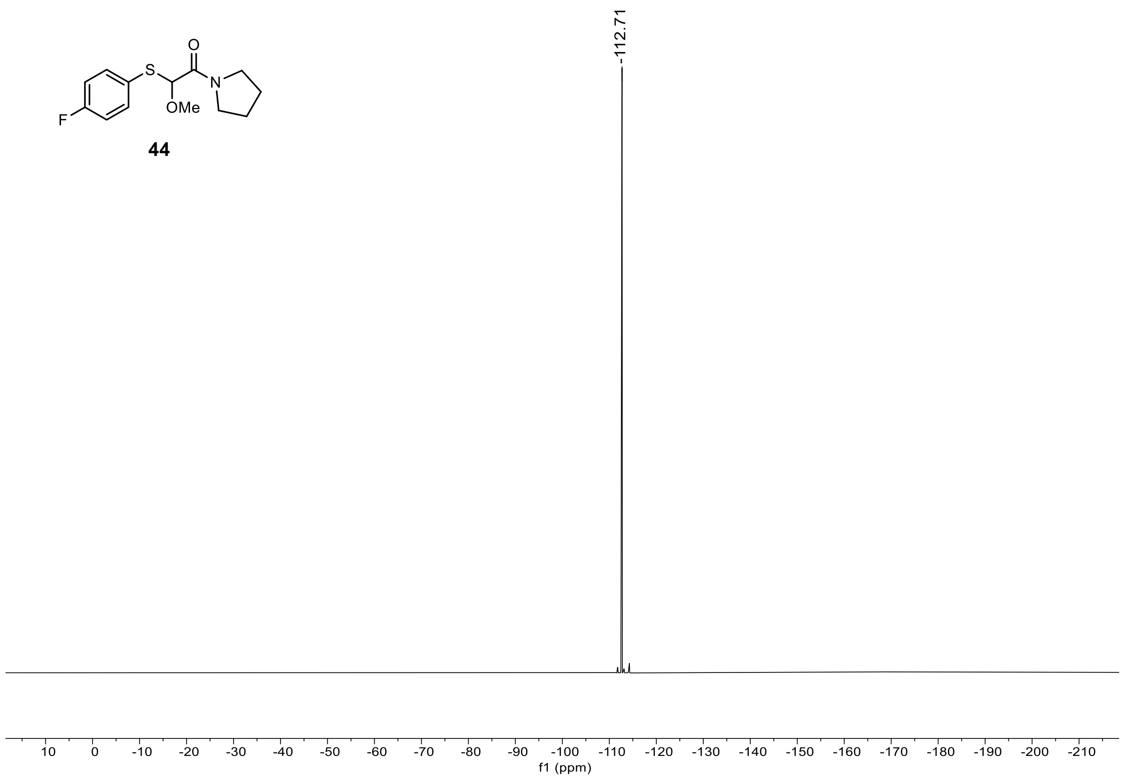

Supplementary Figure 125. ^{19}F NMR spectrum of compound **44** (377 MHz, r.t., $CDCl_3$)

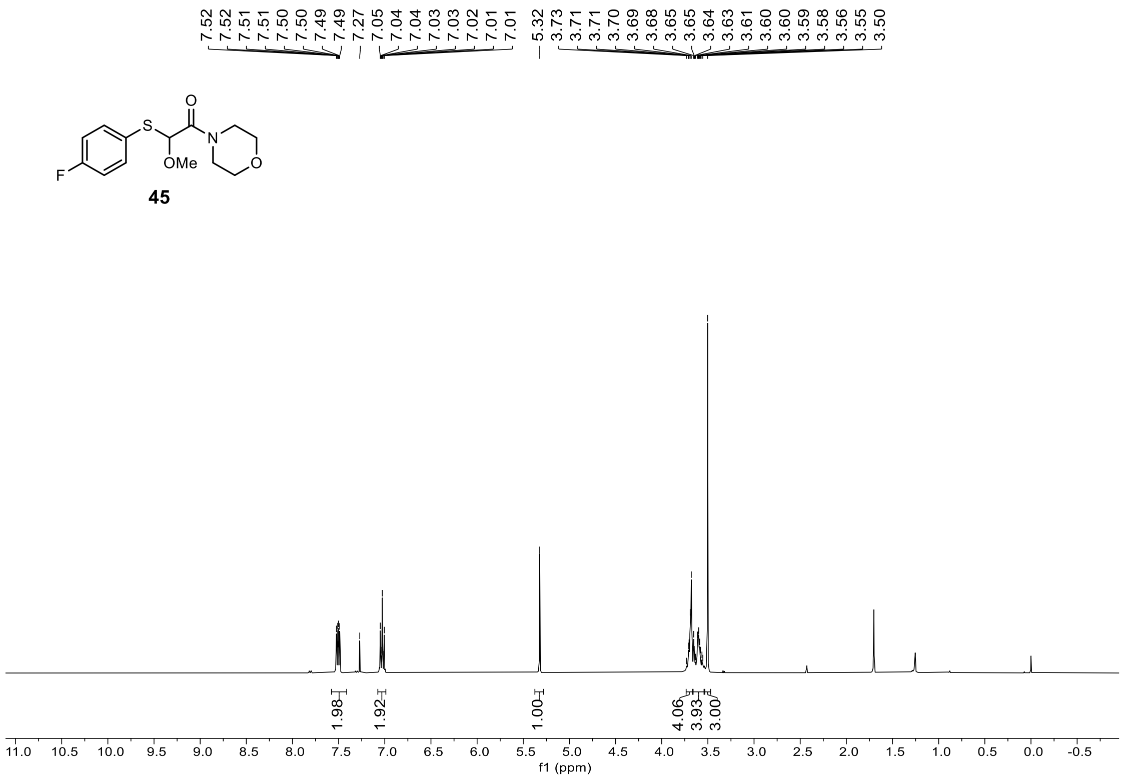

Supplementary Figure 126. ^{1}**H NMR** spectrum of compound **45** (400 MHz, r.t., $CDCl_3$)

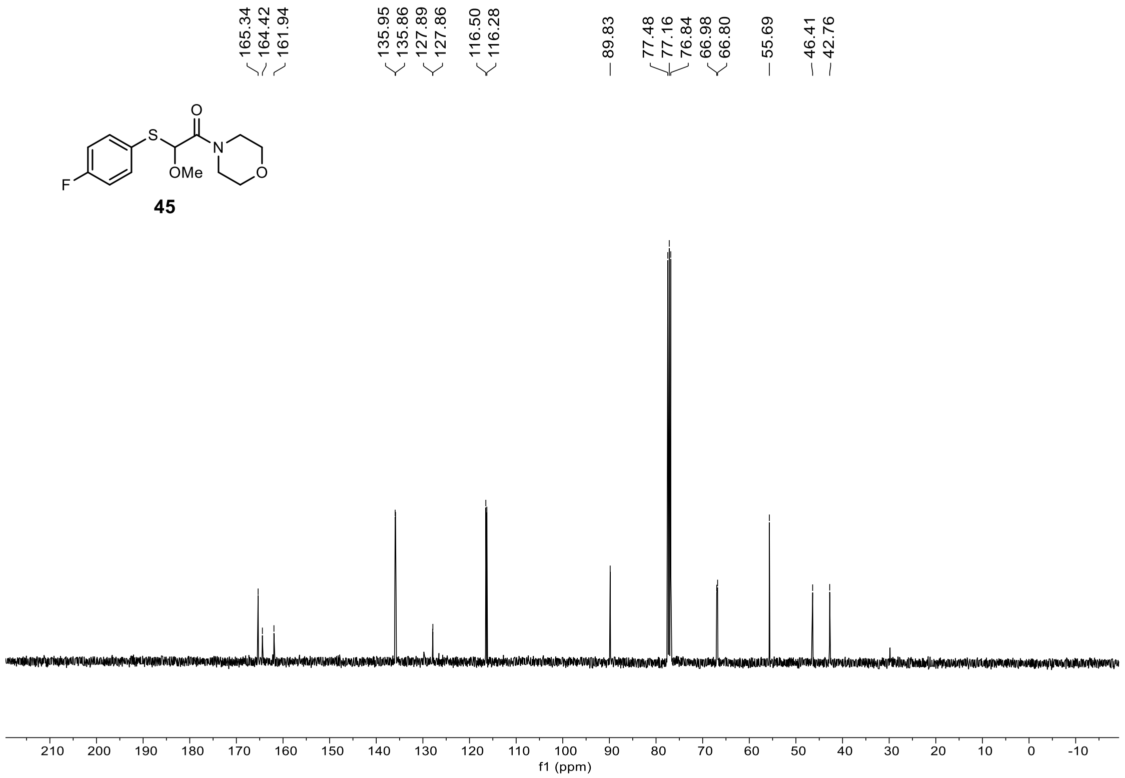

Supplementary Figure 127. ^{13}**C NMR** spectrum of compound **45** (101 MHz, r.t., $CDCl_3$)

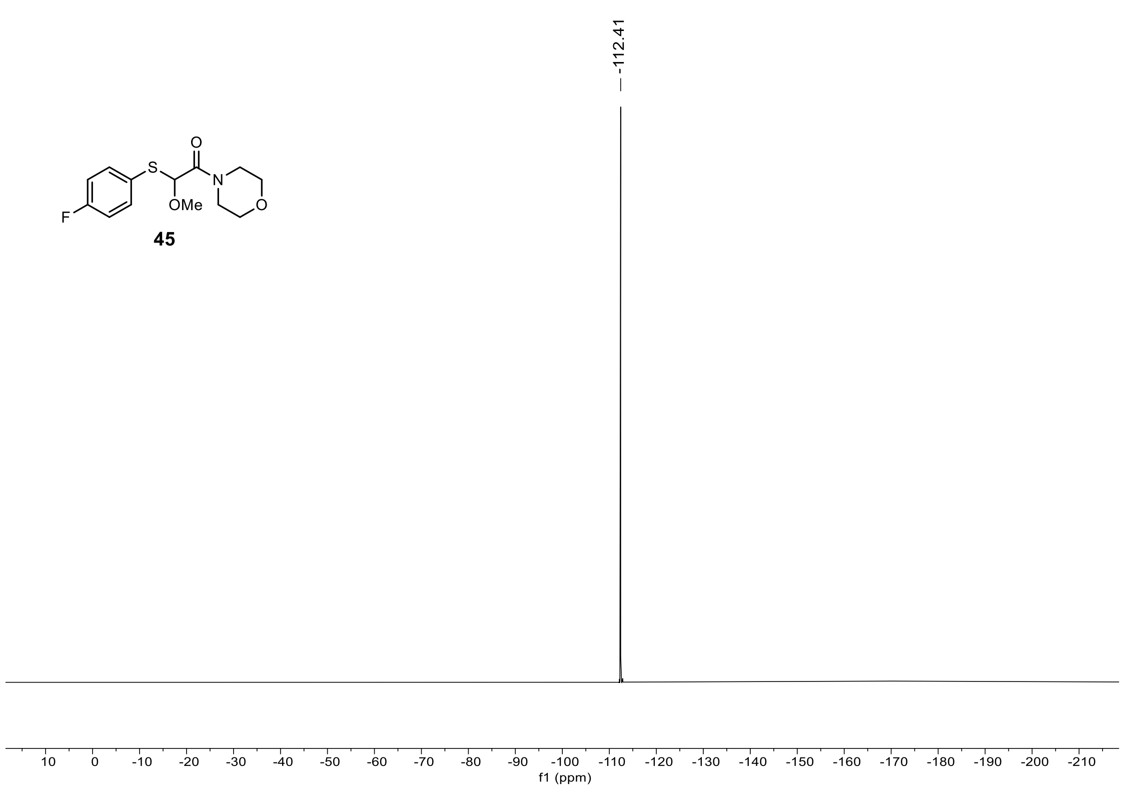

Supplementary Figure 128. ^{19}F NMR spectrum of compound **45** (377 MHz, r.t., $CDCl_3$)

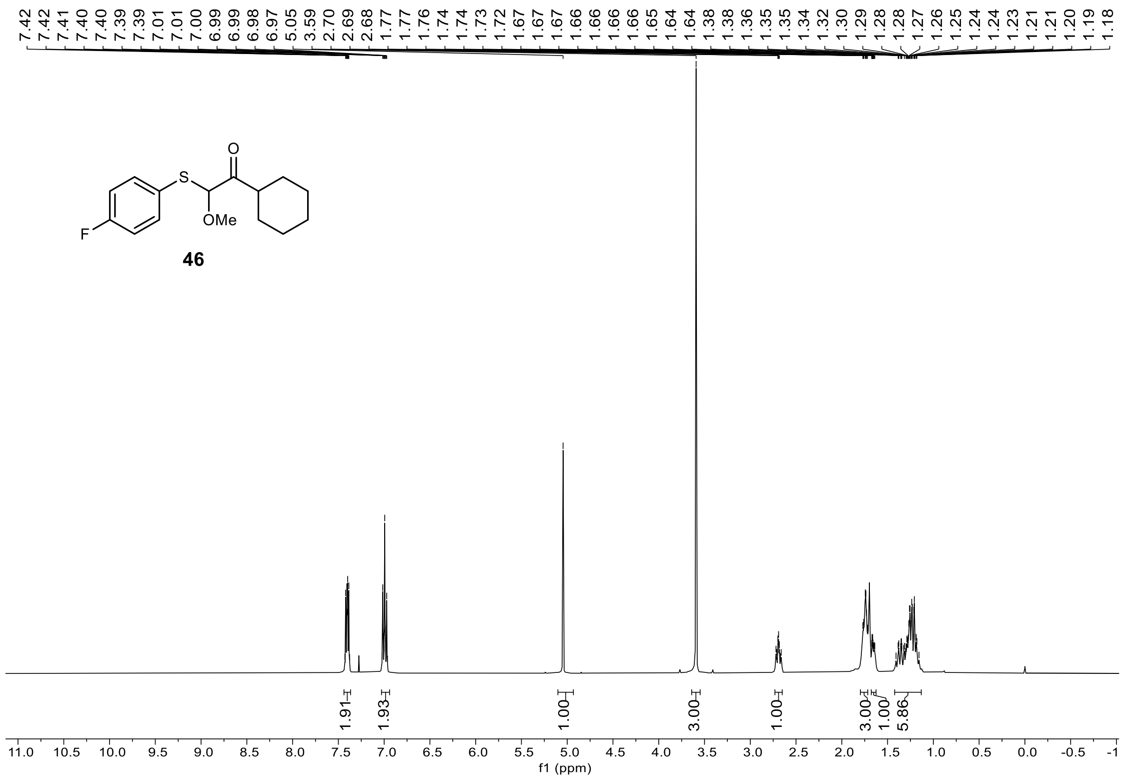

Supplementary Figure 129. ^{1}**H NMR** spectrum of compound **46** (400 MHz, r.t., $CDCl_3$)

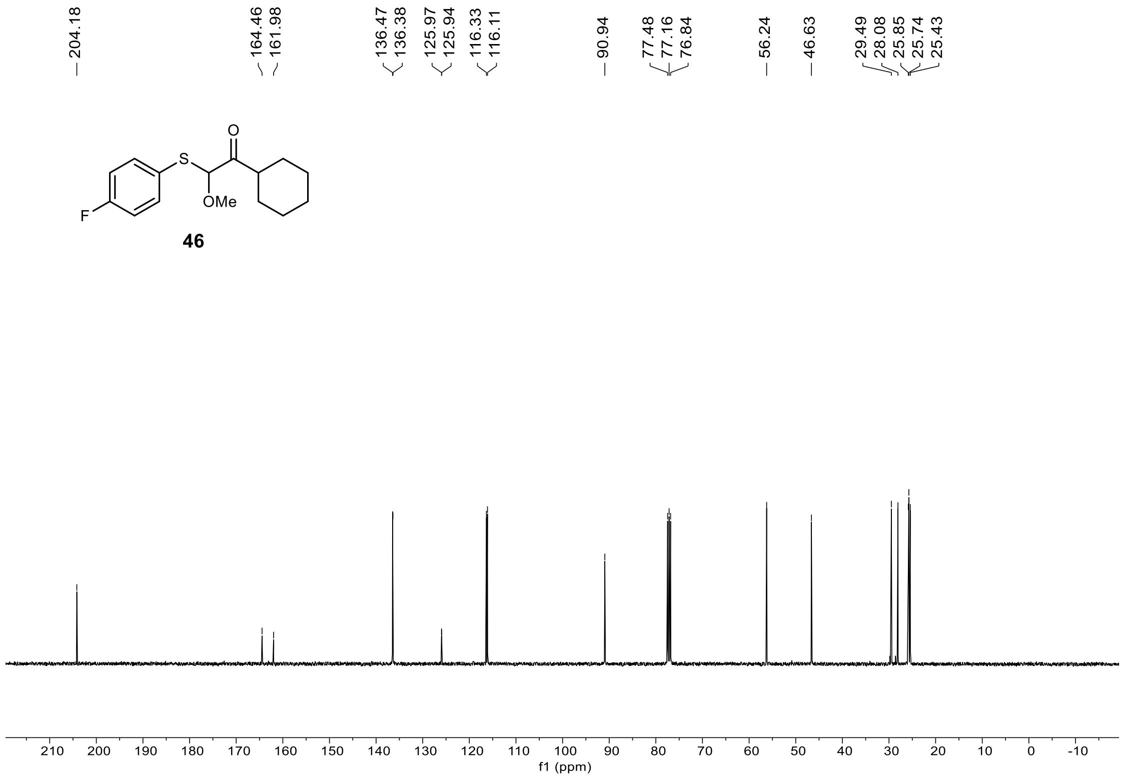

Supplementary Figure 130. ^{13}**C NMR** spectrum of compound **46** (101 MHz, r.t., $CDCl_3$)

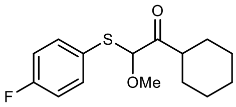

46

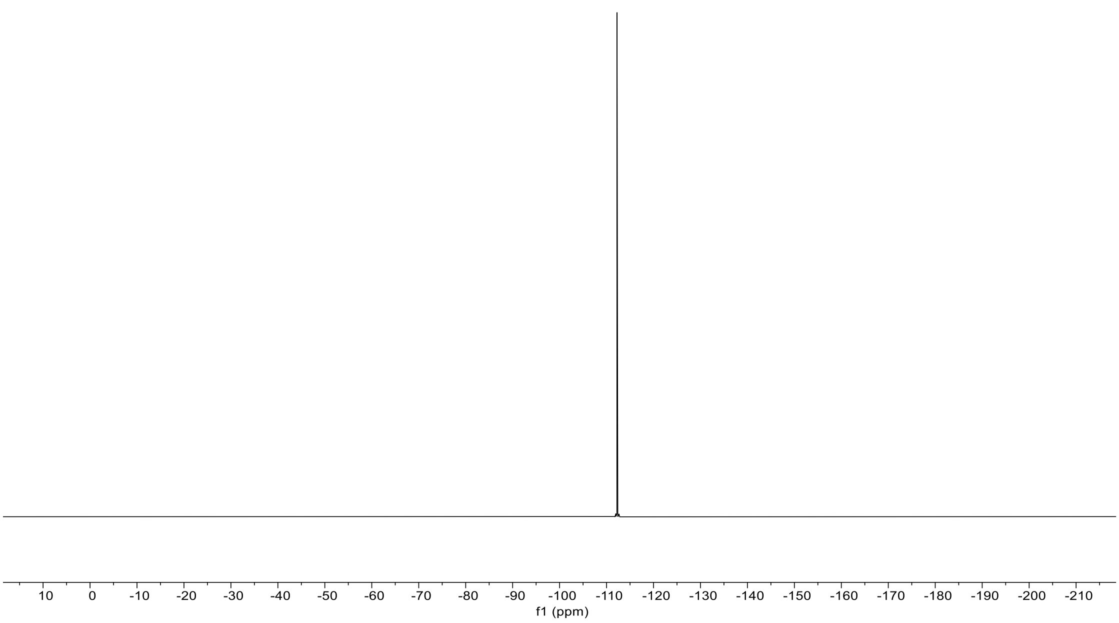

Supplementary Figure 131. ^{19}F NMR spectrum of compound **46** (377 MHz, r.t., $CDCl_3$)

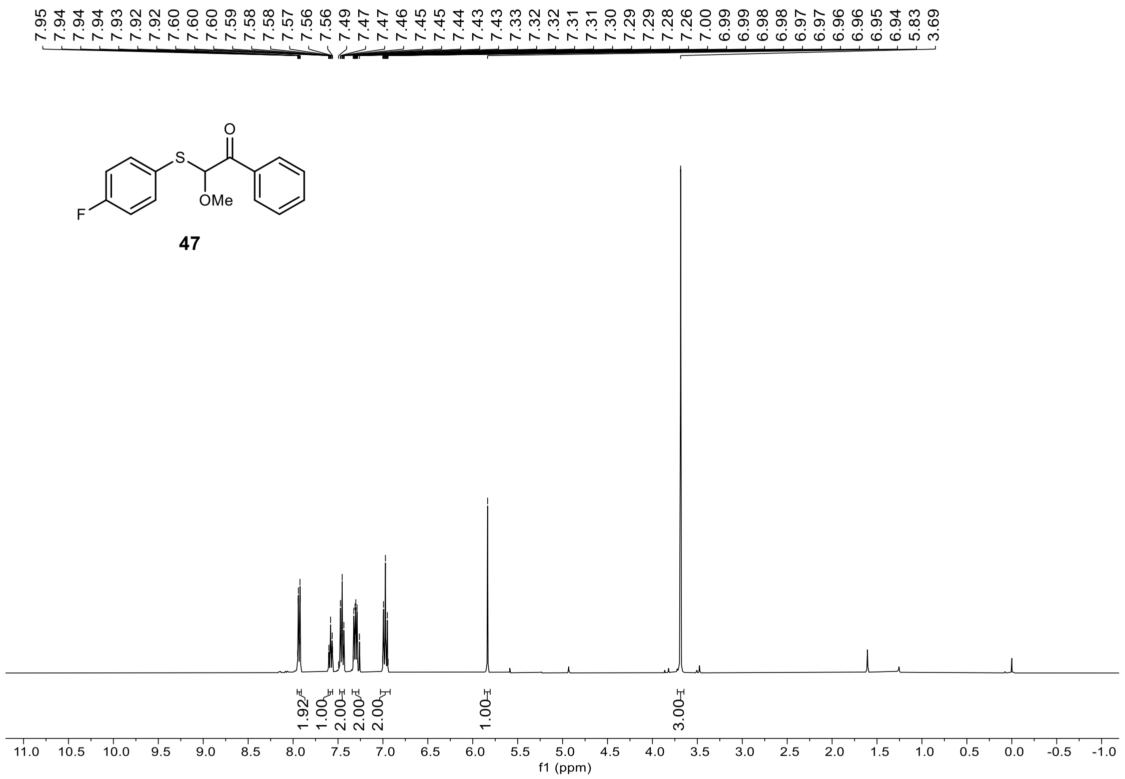

Supplementary Figure 132. **^{1}H NMR** spectrum of compound **47** (400 MHz, r.t., $CDCl_3$)

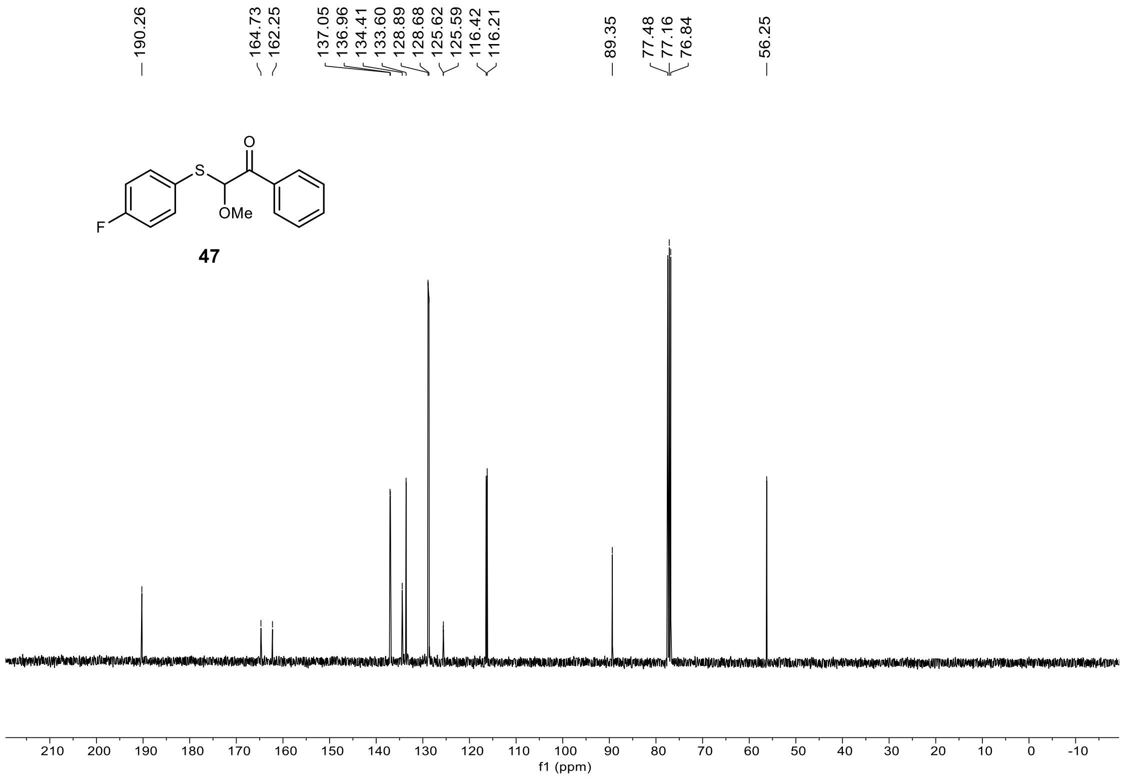

Supplementary Figure 133. **^{13}C NMR** spectrum of compound **47** (101 MHz, r.t., $CDCl_3$)

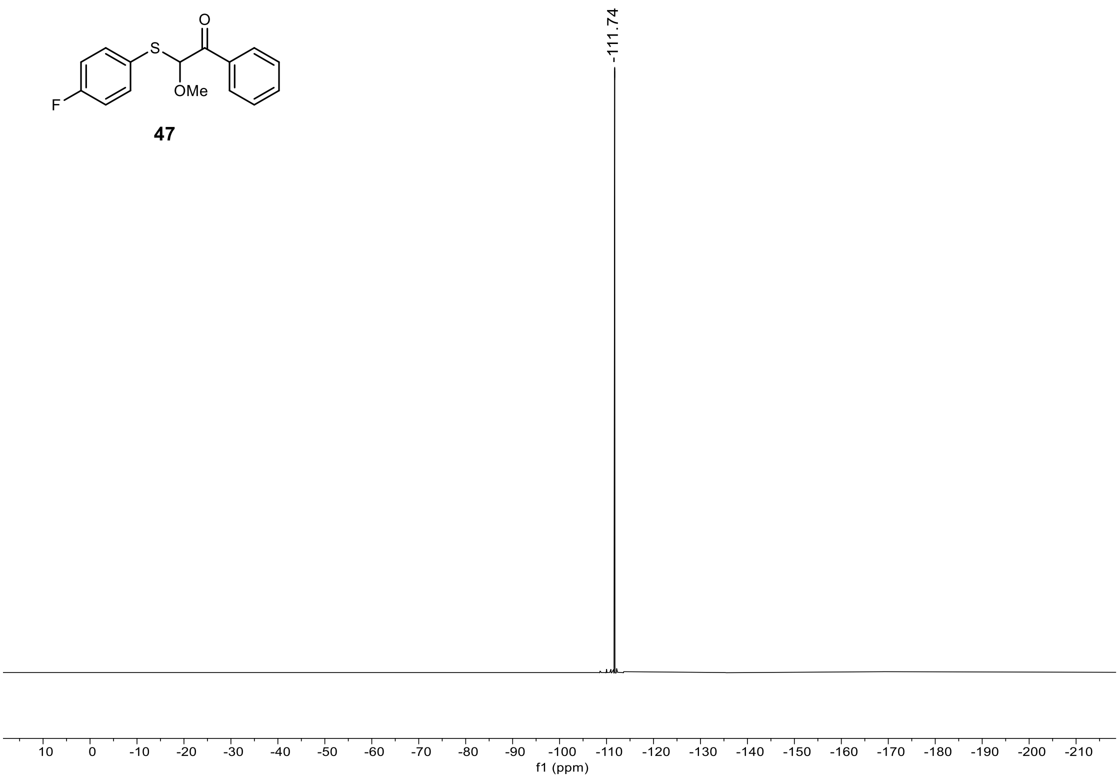

Supplementary Figure 134. ^{19}**F NMR** spectrum of compound **47** (377 MHz, r.t., $CDCl_3$)

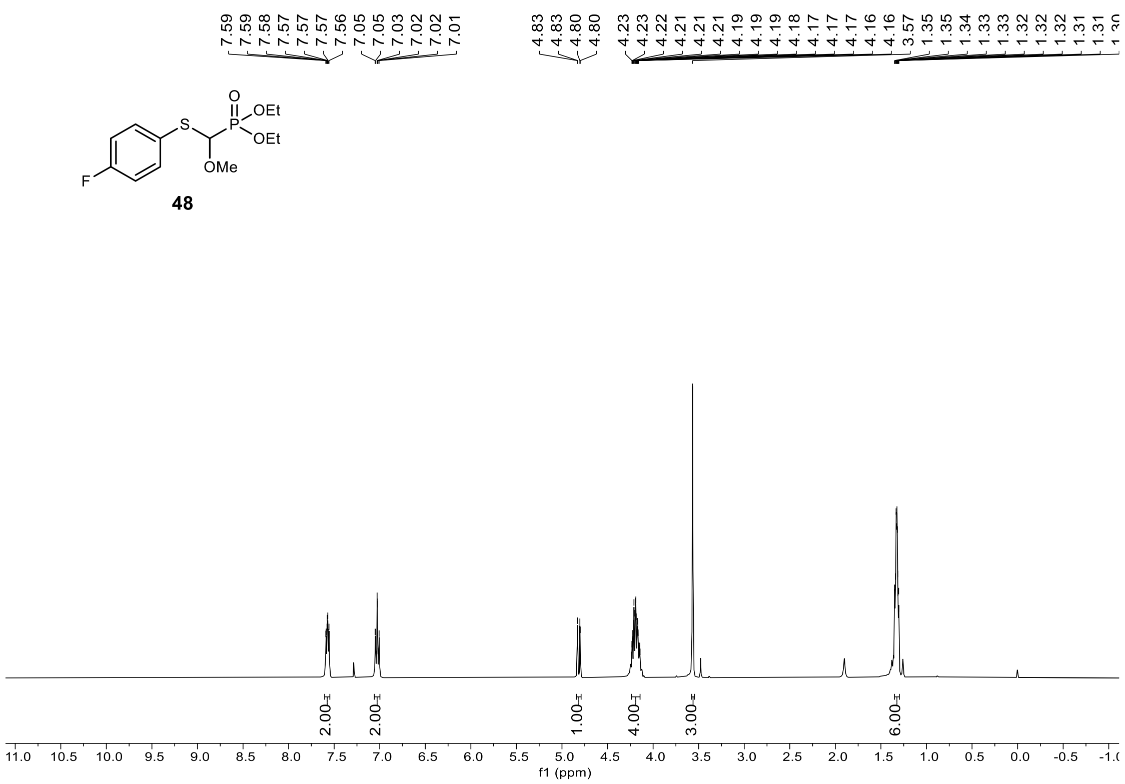

Supplementary Figure 135. ^{1}**H NMR** spectrum of compound **48** (400 MHz, r.t., $CDCl_3$)

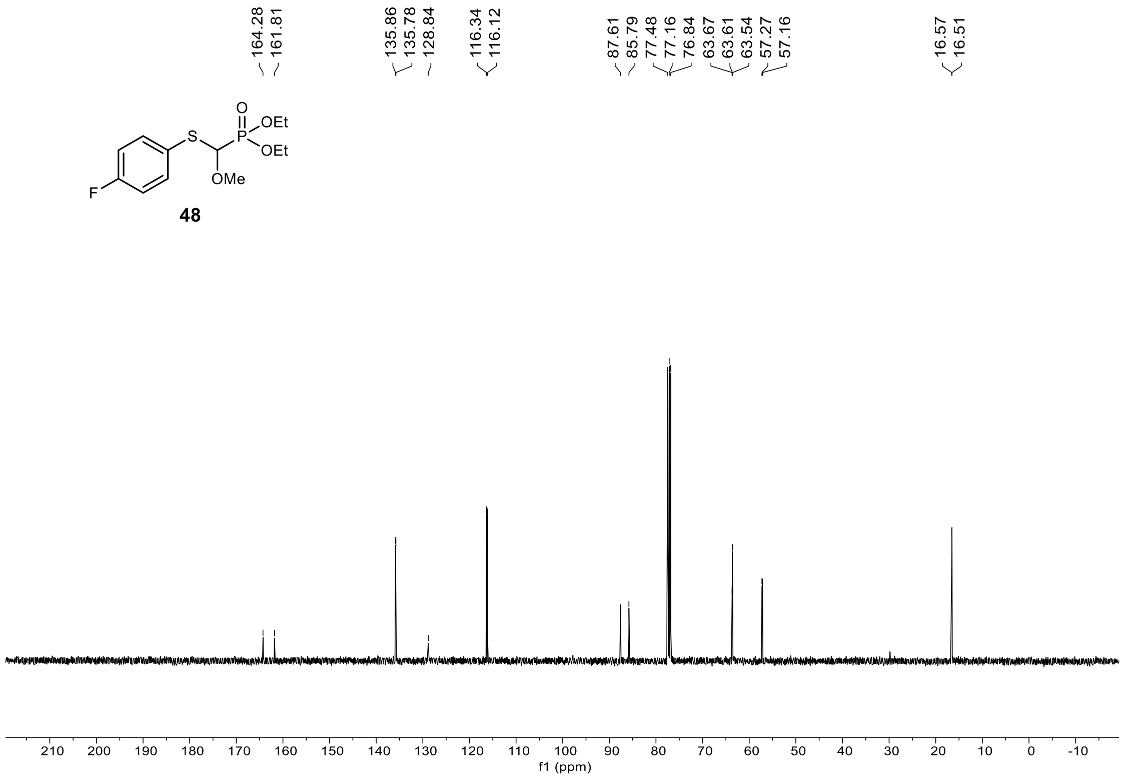

Supplementary Figure 136. ^{13}**C NMR** spectrum of compound **48** (101 MHz, r.t., $CDCl_3$)

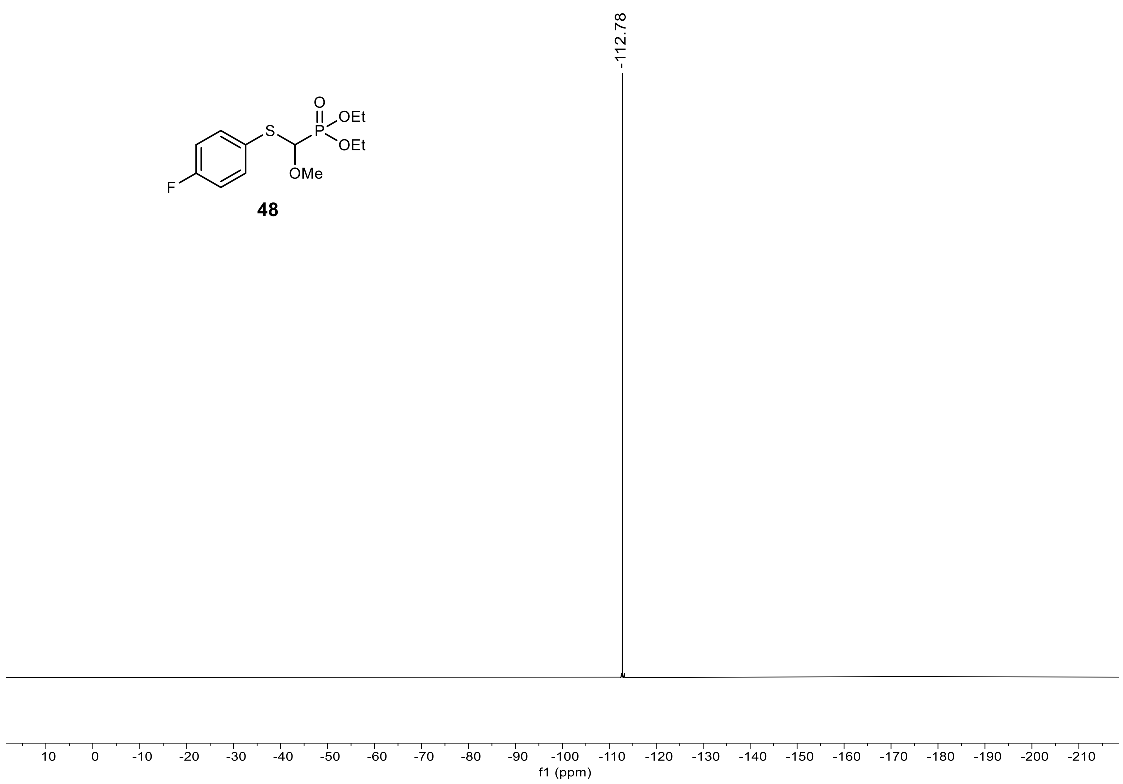

Supplementary Figure 137. ^{19}F NMR spectrum of compound **48** (377 MHz, r.t., $CDCl_3$)

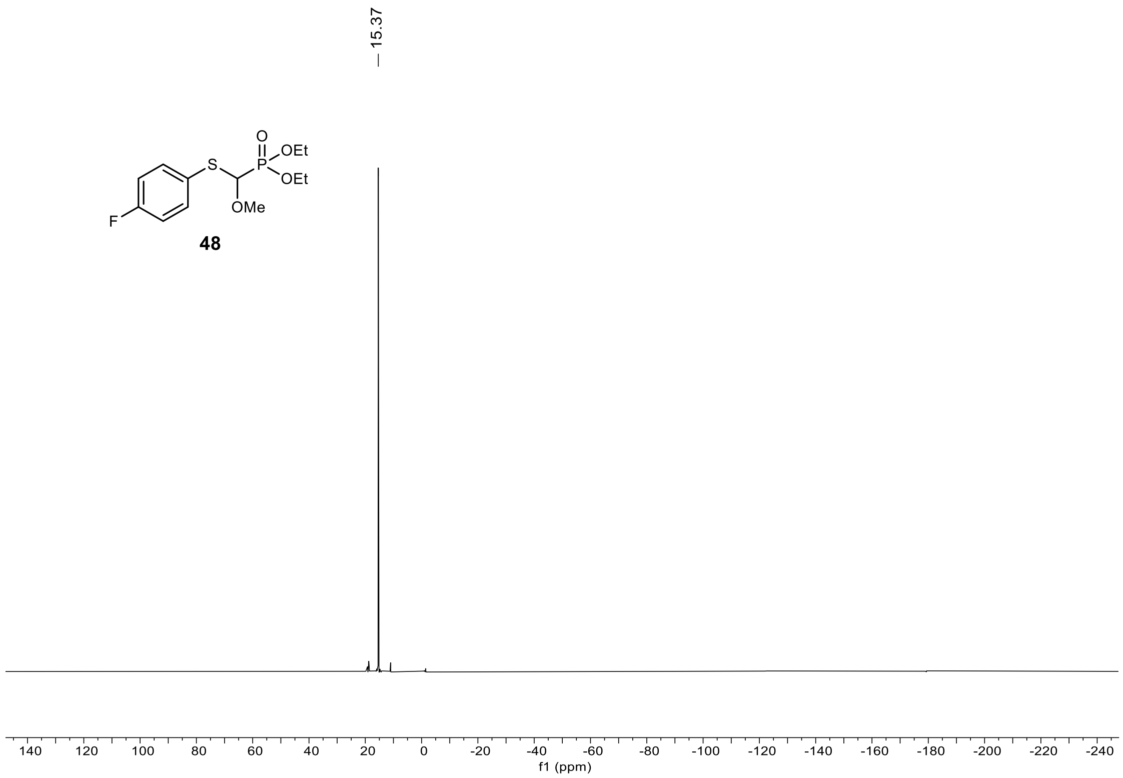

Supplementary Figure 138. ^{31}P NMR spectrum of compound **48** (162 MHz, r.t., $CDCl_3$)

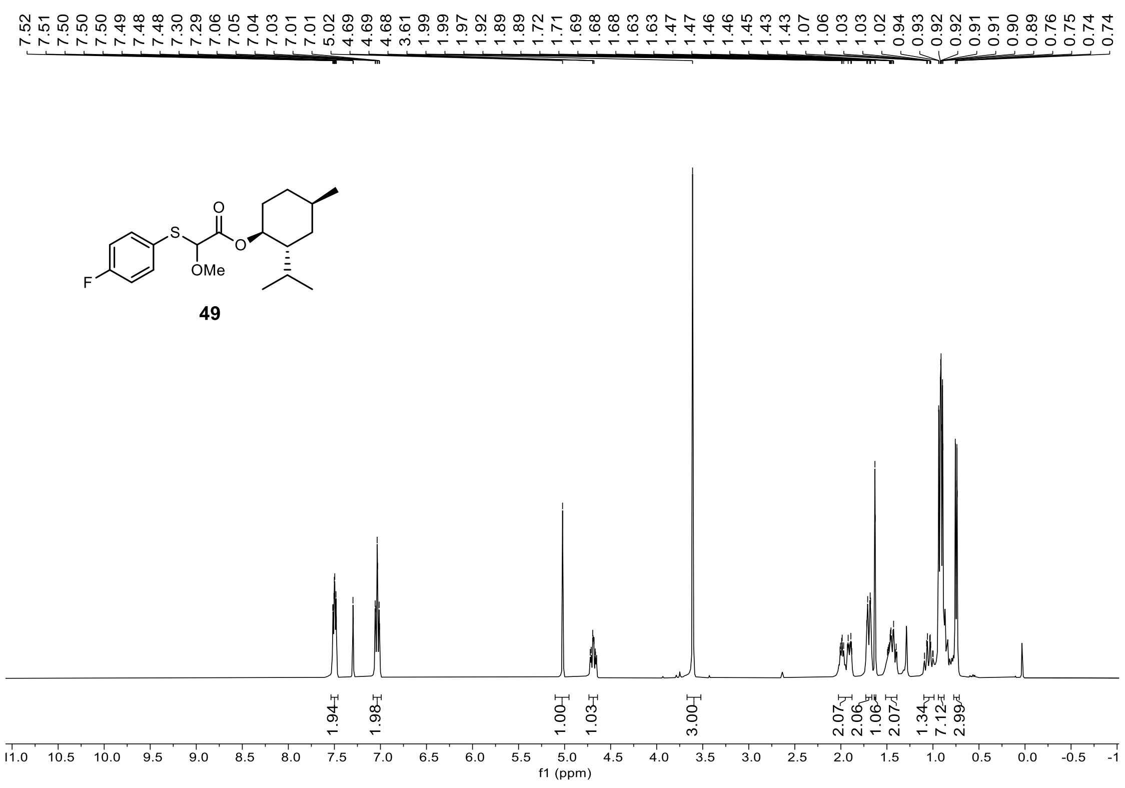

Supplementary Figure 139. ^{1}H NMR spectrum of compound **49** (400 MHz, r.t., $CDCl_3$)

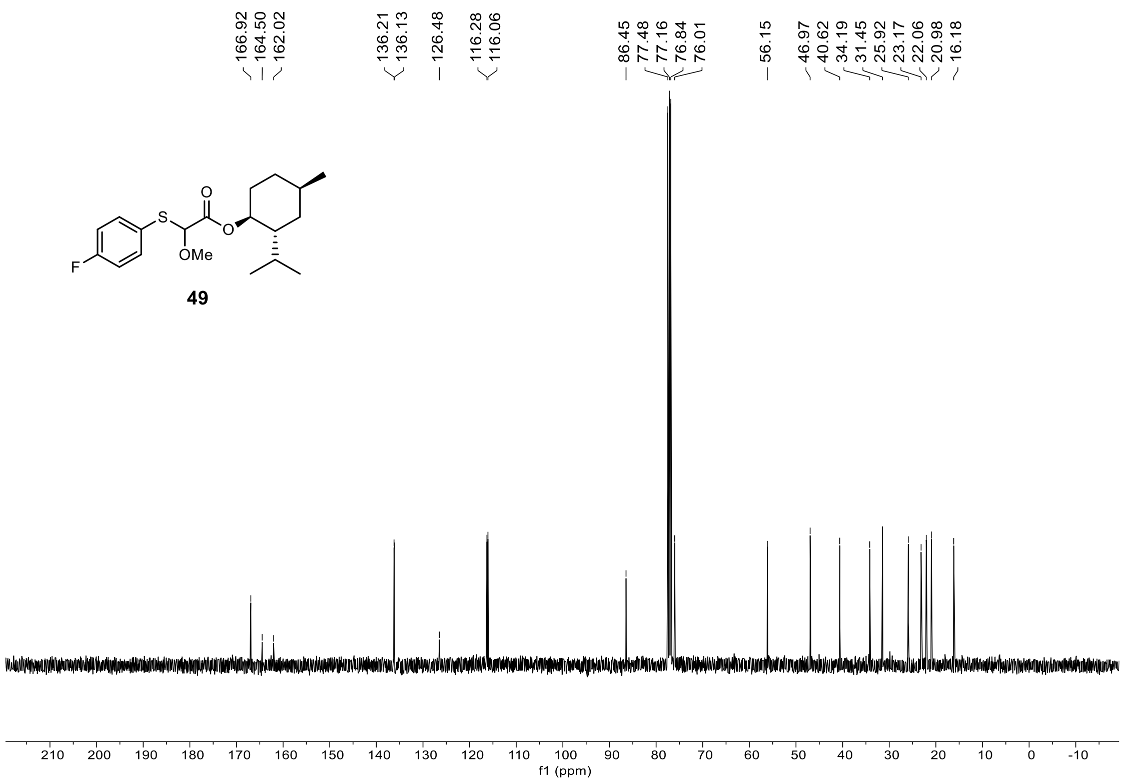

Supplementary Figure 140. ^{13}C NMR spectrum of compound **49** (101 MHz, r.t., $CDCl_3$)

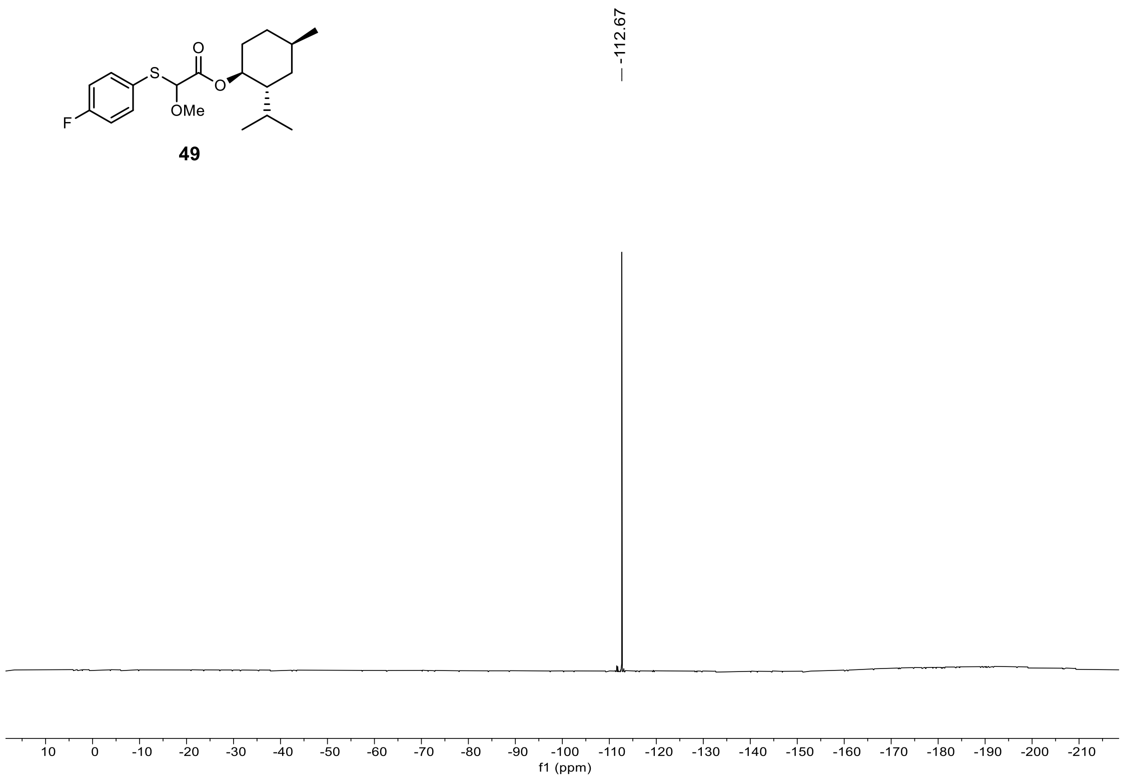

Supplementary Figure 141. ^{19}**F NMR** spectrum of compound **49** (377 MHz, r.t., $CDCl_3$)

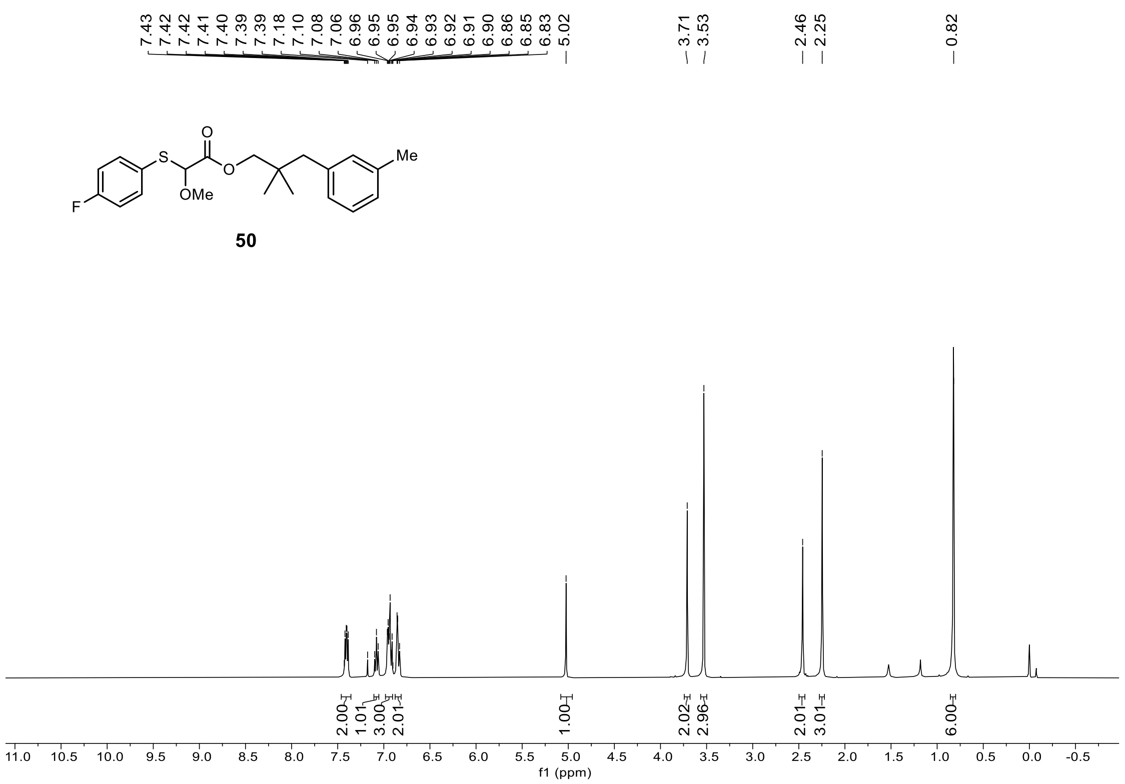

Supplementary Figure 142. **^{1}H NMR** spectrum of compound **50** (400 MHz, r.t., $CDCl_3$)

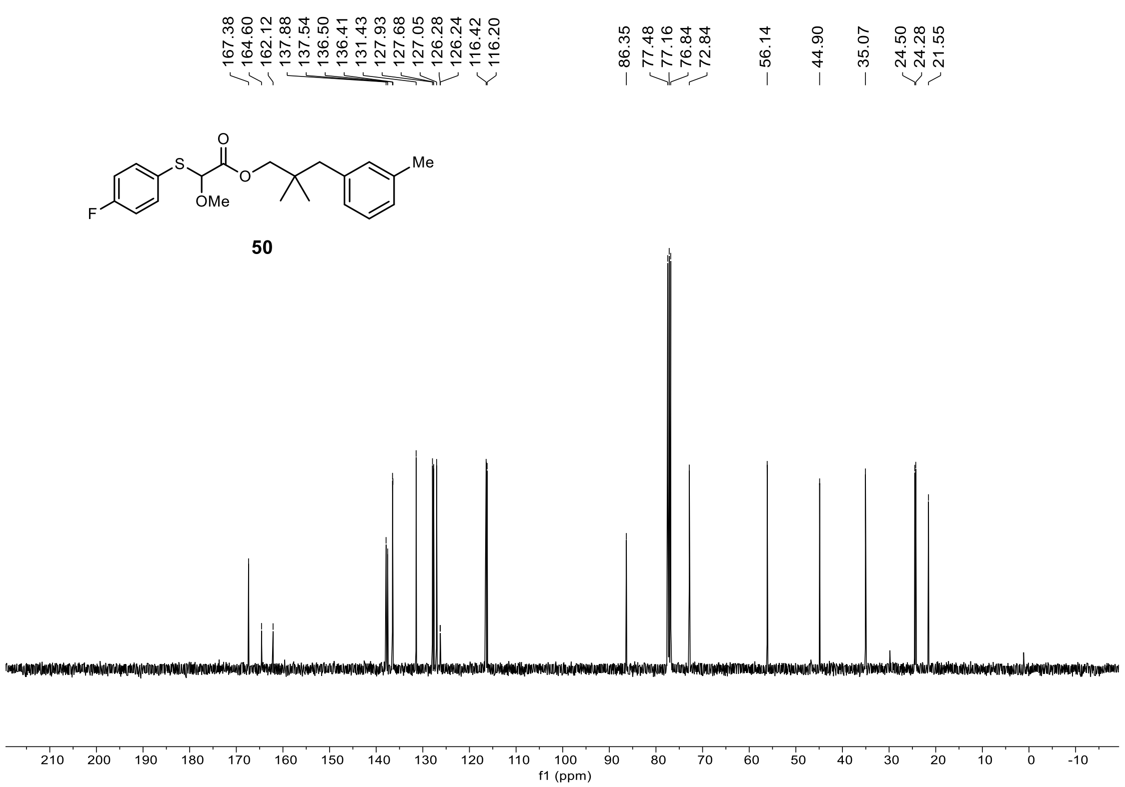

Supplementary Figure 143. **^{13}C NMR** spectrum of compound **50** (101 MHz, r.t., $CDCl_3$)

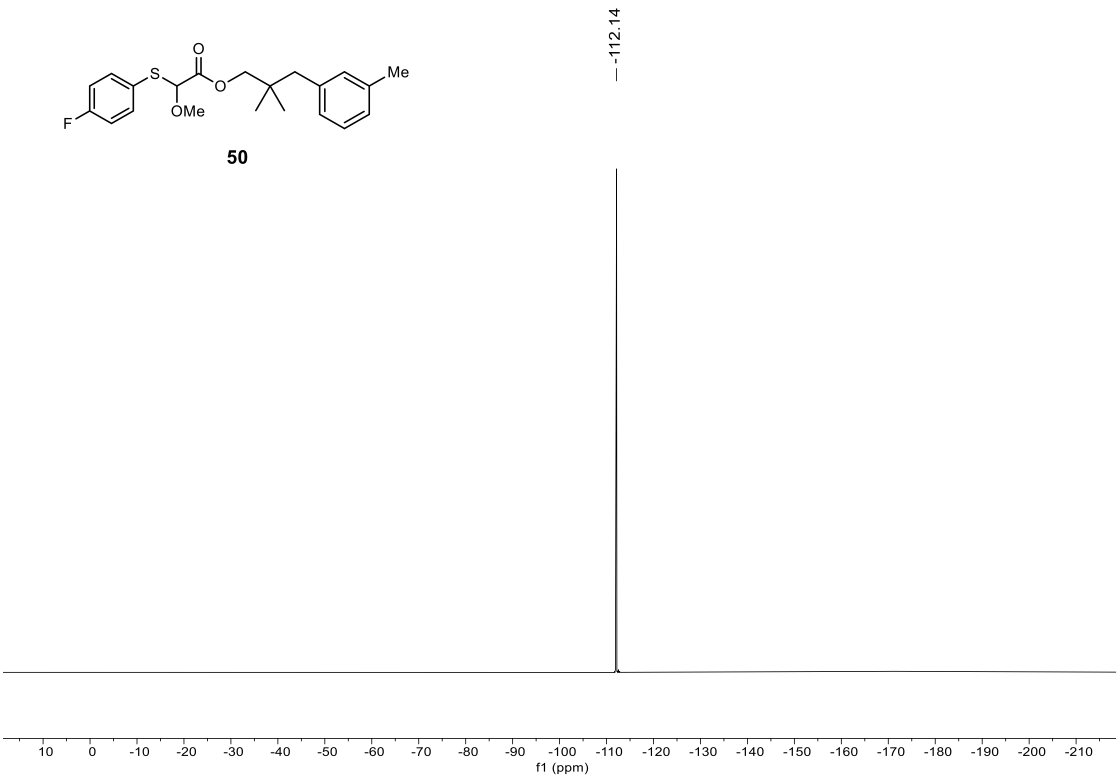

Supplementary Figure 144. ^{19}F NMR spectrum of compound **50** (377 MHz, r.t., $CDCl_3$)

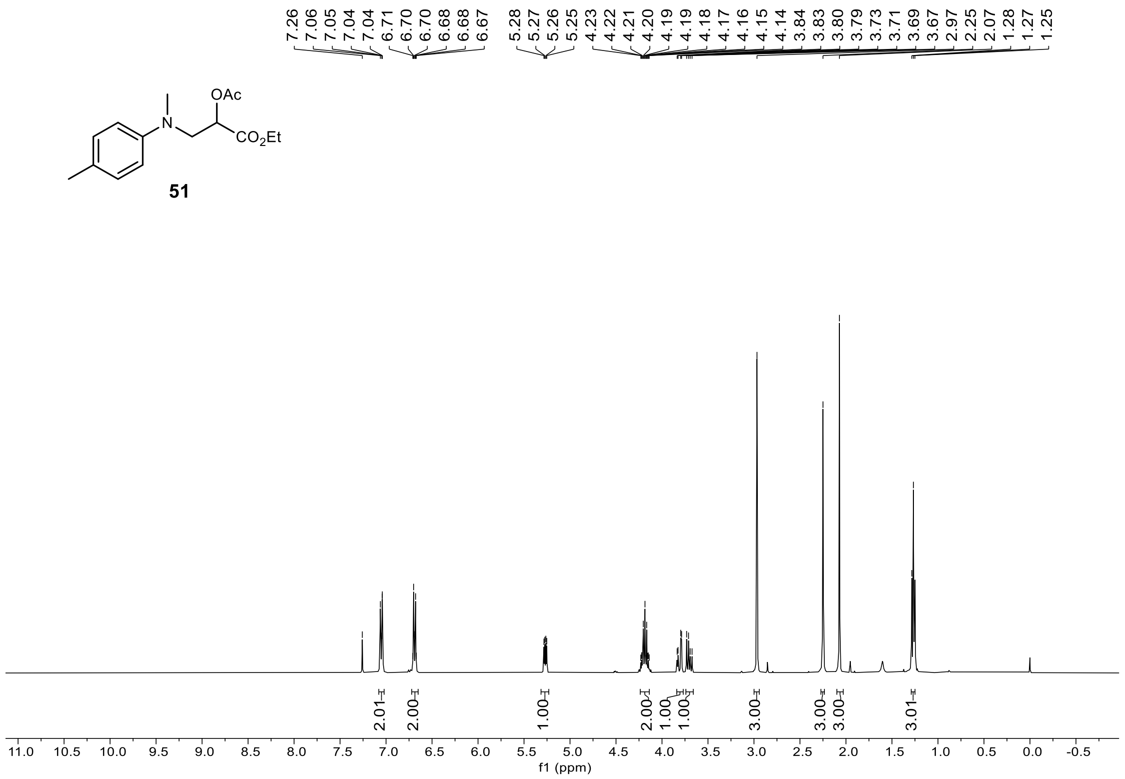

Supplementary Figure 145. ^{1}**H NMR** spectrum of compound **51** (400 MHz, r.t., $CDCl_3$)

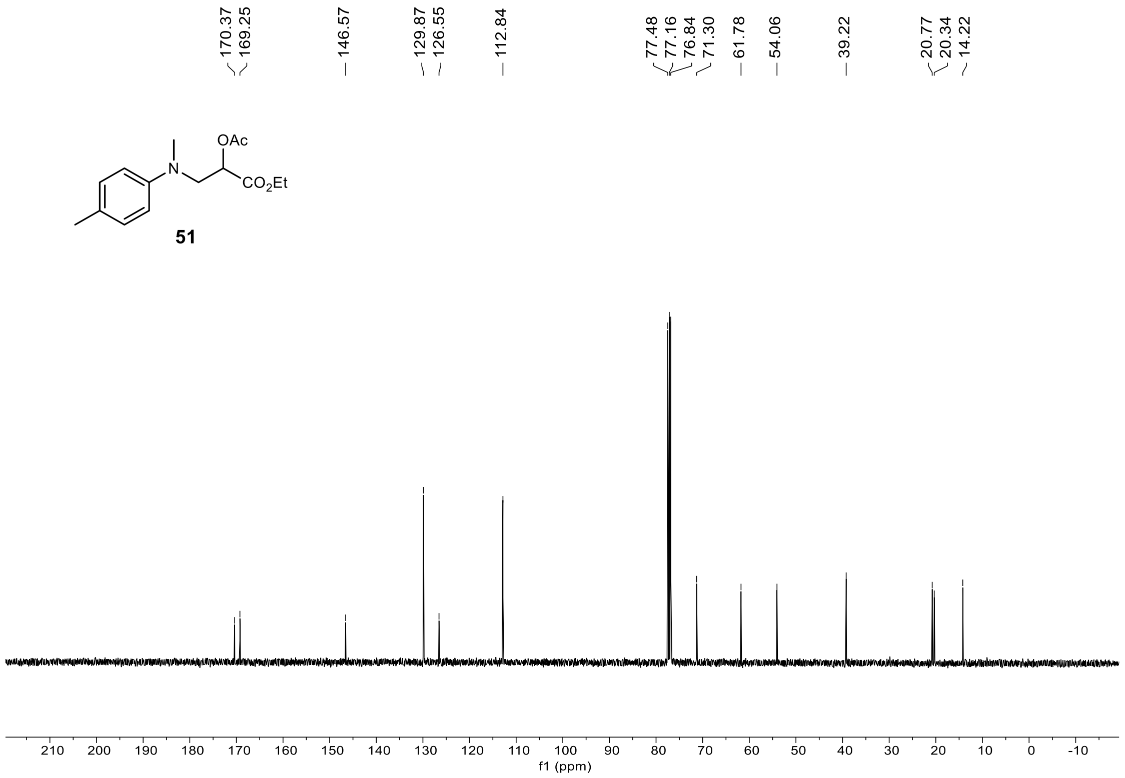

Supplementary Figure 146. ^{13}**C NMR** spectrum of compound **51** (101 MHz, r.t., $CDCl_3$)

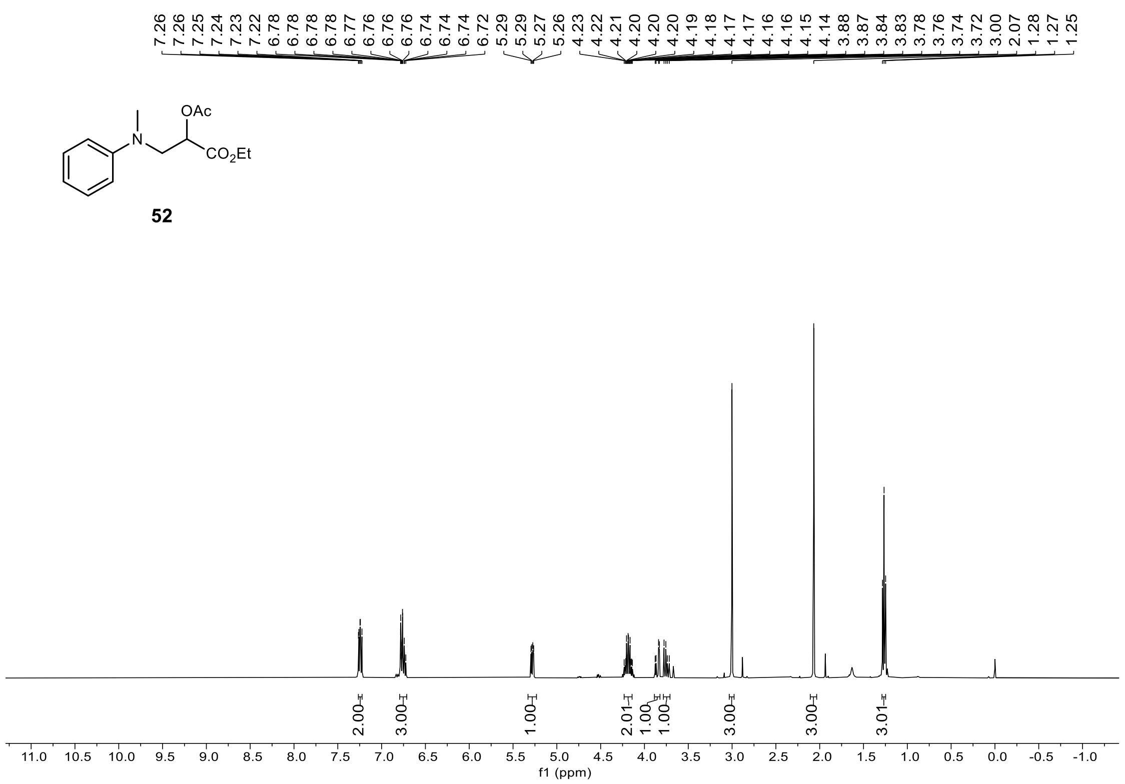

Supplementary Figure 147. **^{1}H NMR** spectrum of compound **52** (400 MHz, r.t., $CDCl_3$)

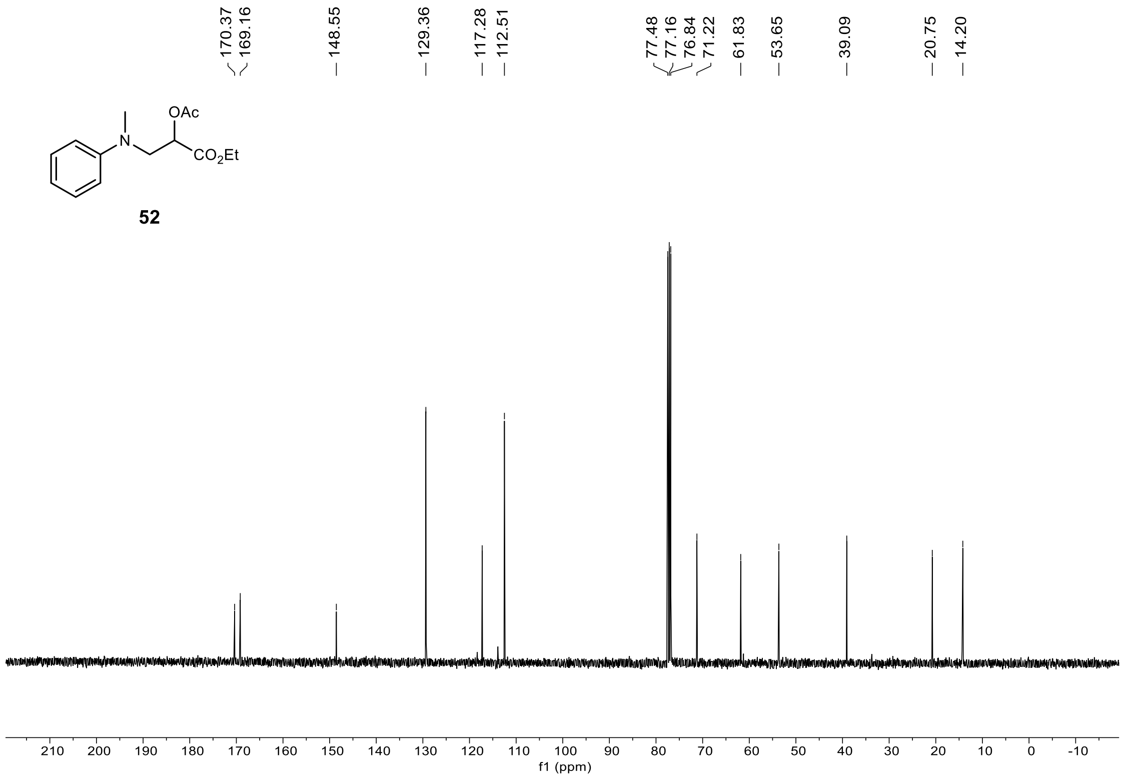

Supplementary Figure 148. **^{13}C NMR** spectrum of compound **52** (101 MHz, r.t., $CDCl_3$)

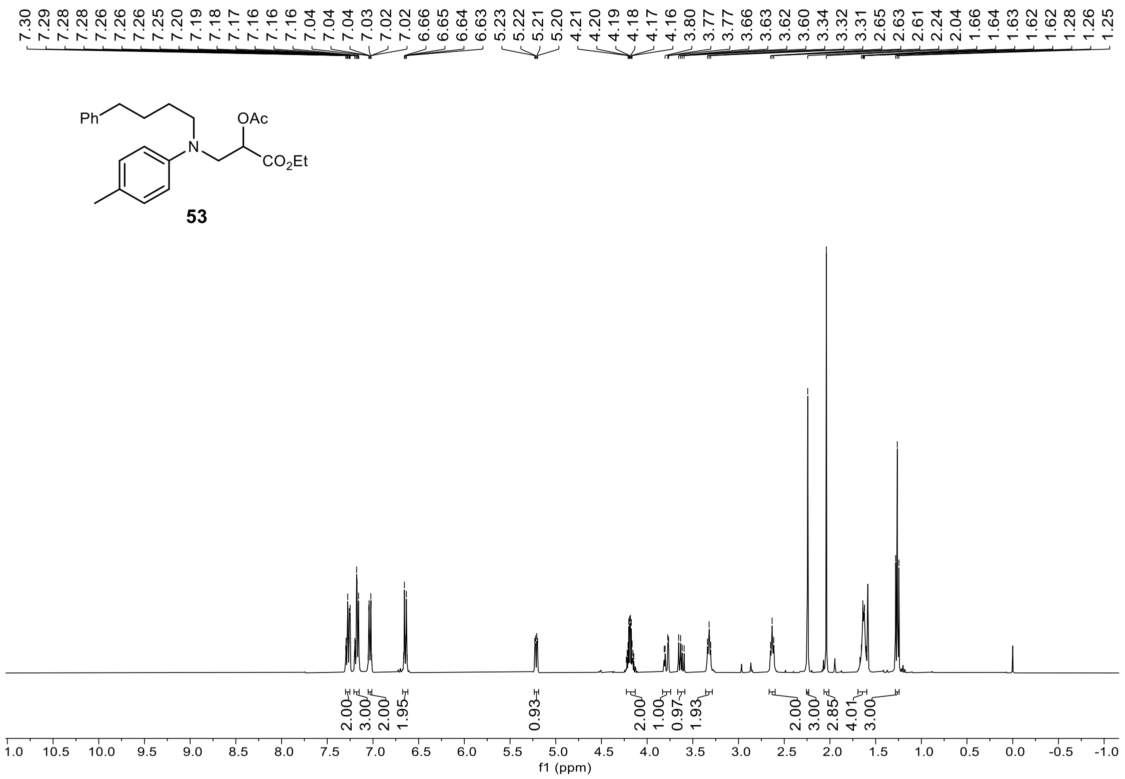

Supplementary Figure 149. ^{1}**H NMR** spectrum of compound **53** (400 MHz, r.t., $CDCl_3$)

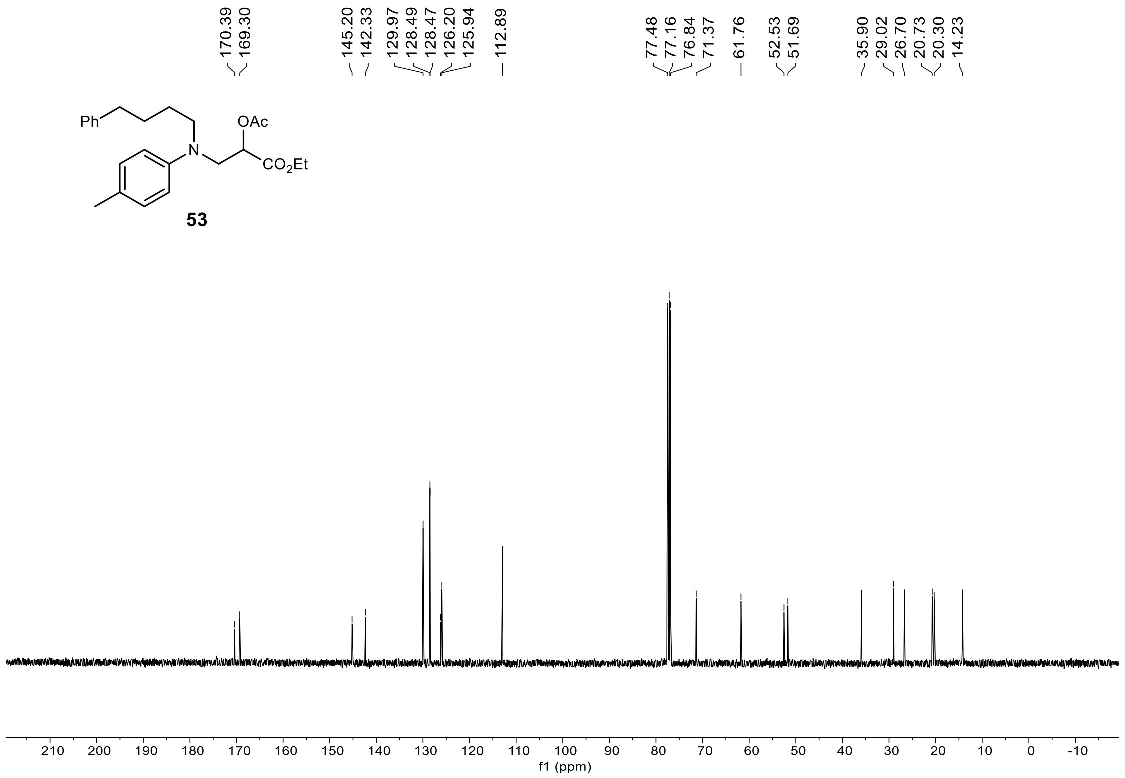

Supplementary Figure 150. ^{13}**C NMR** spectrum of compound **53** (101 MHz, r.t., $CDCl_3$)

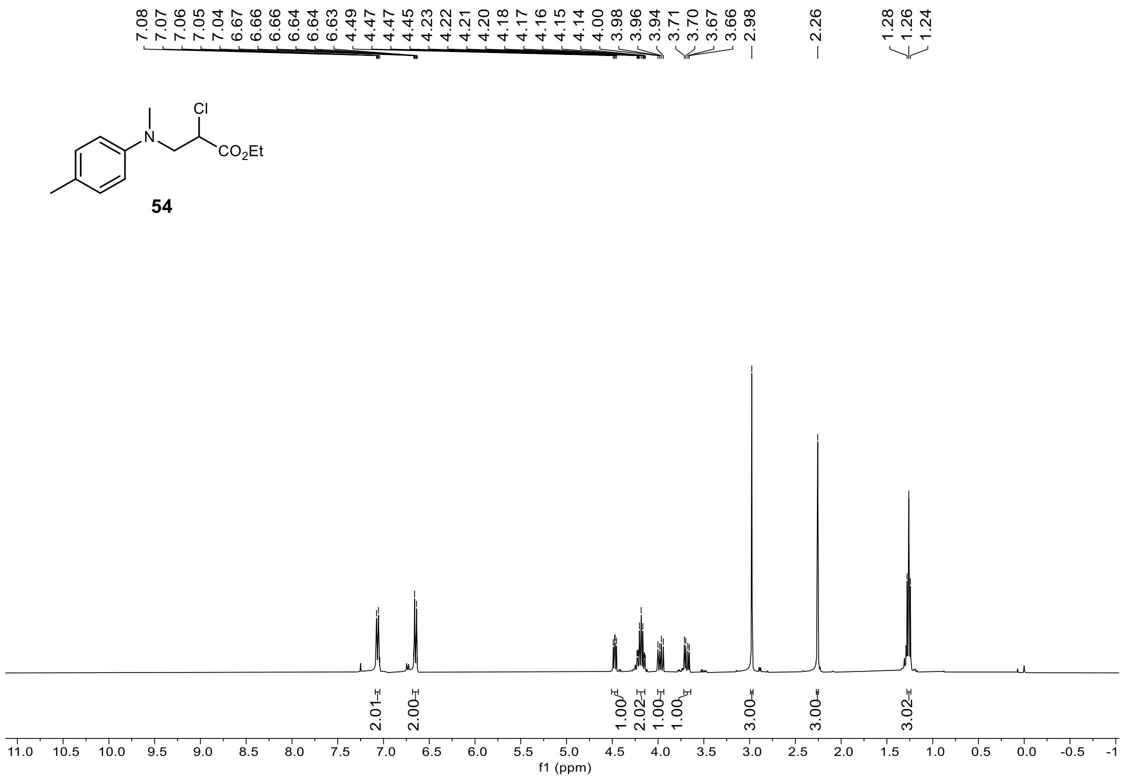

Supplementary Figure 151. ^{1}H NMR spectrum of compound **54** (400 MHz, r.t., $CDCl_3$)

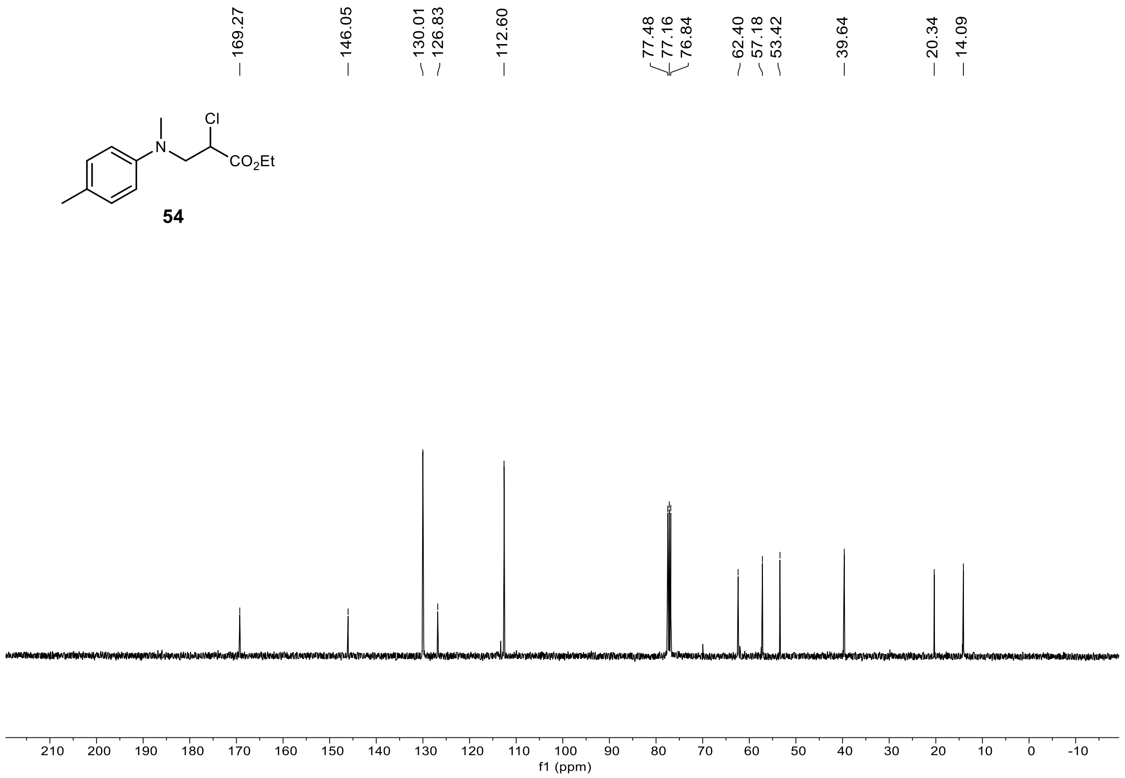

Supplementary Figure 152. ^{13}C NMR spectrum of compound **54** (101 MHz, r.t., $CDCl_3$)

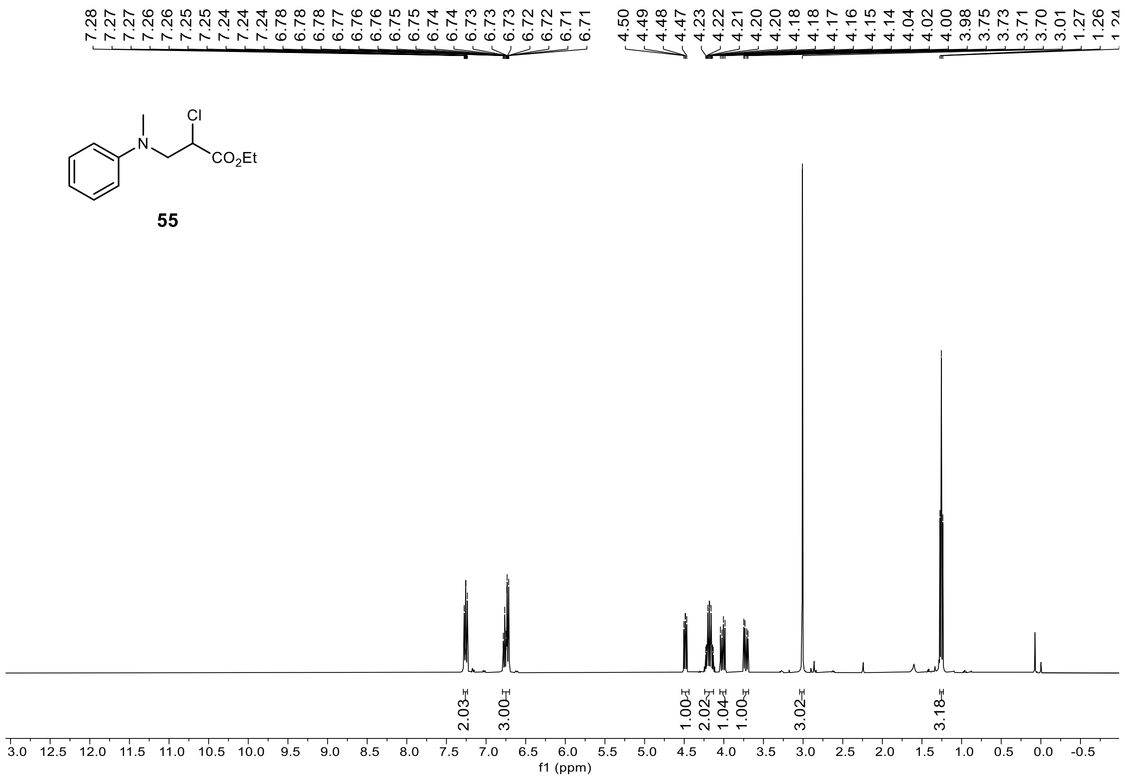

Supplementary Figure 153. ^{1}**H NMR** spectrum of compound **55** (400 MHz, r.t., $CDCl_3$)

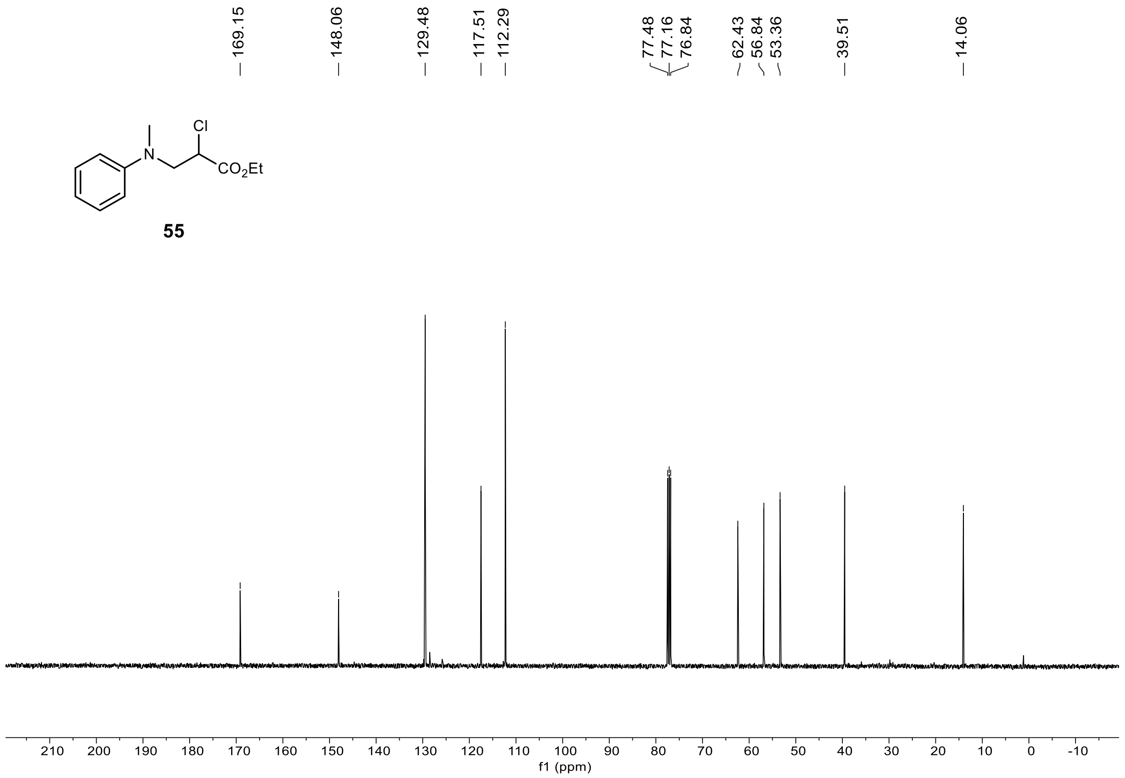

Supplementary Figure 154. ^{13}**C NMR** spectrum of compound **55** (101 MHz, r.t., $CDCl_3$)

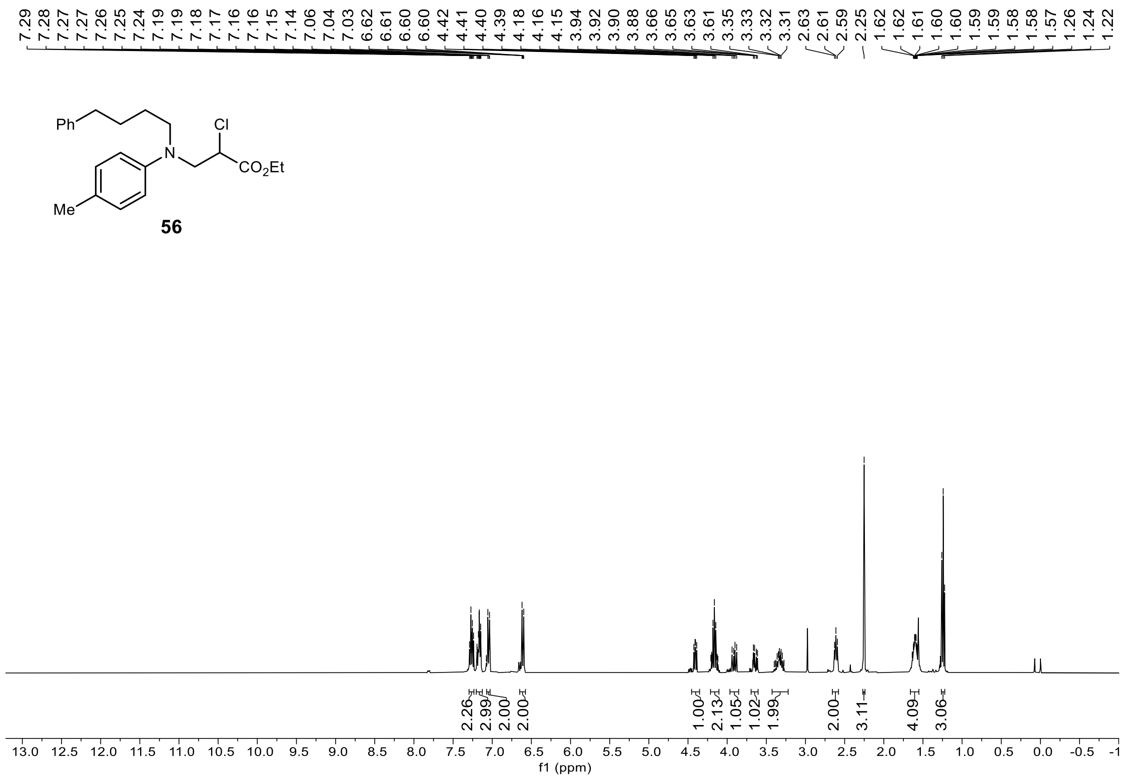

Supplementary Figure 155. **^{1}H NMR** spectrum of compound **56** (400 MHz, r.t., $CDCl_3$)

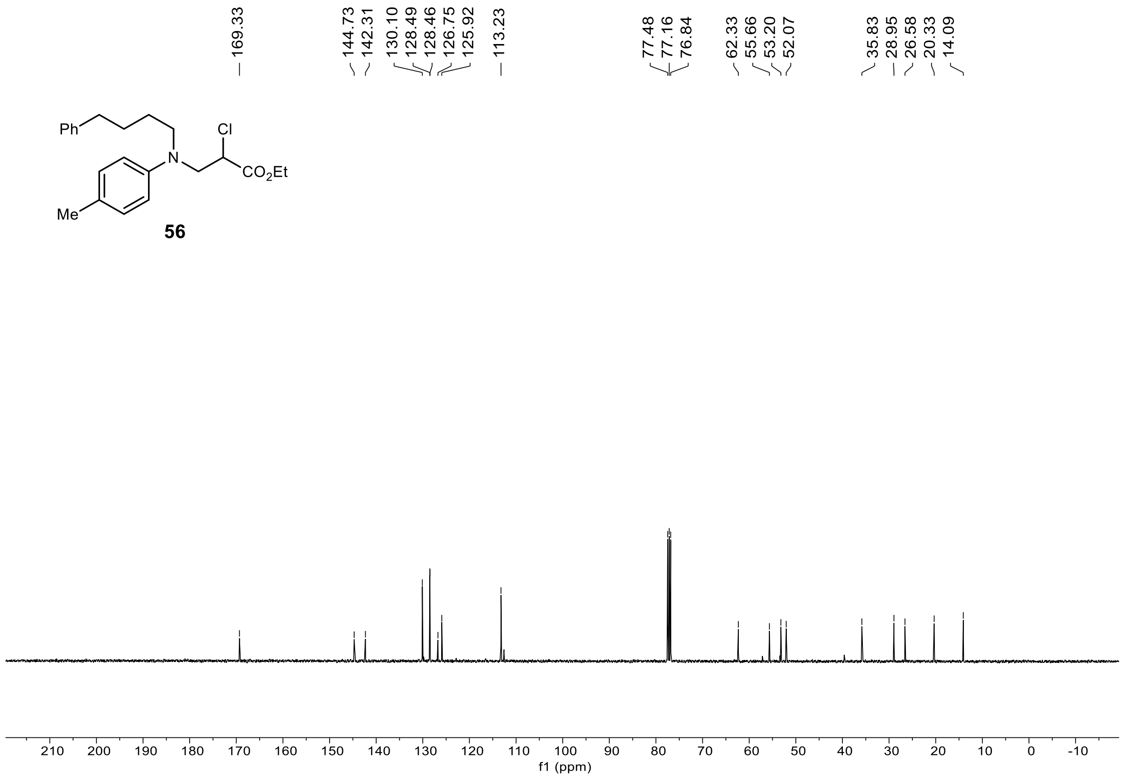

Supplementary Figure 156. **^{13}C NMR** spectrum of compound **56** (101 MHz, r.t., $CDCl_3$)

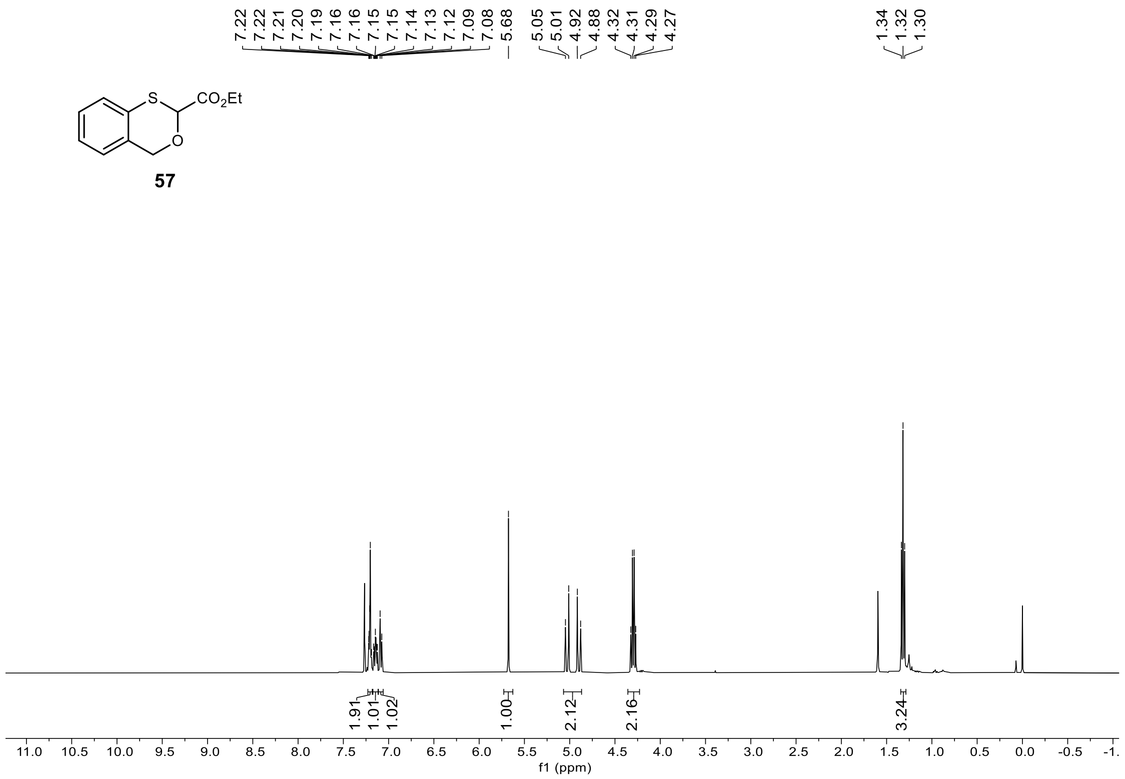

Supplementary Figure 157. ^{1}**H NMR** spectrum of compound **57** (400 MHz, r.t., $CDCl_3$)

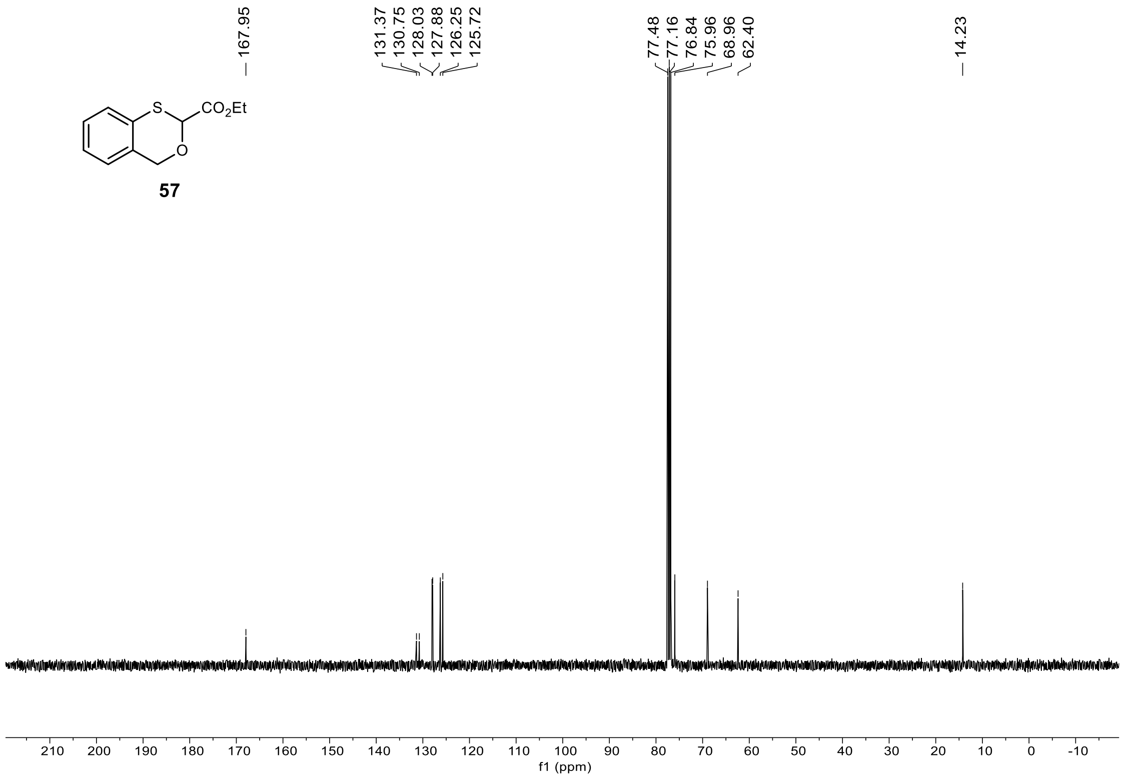

Supplementary Figure 158. ^{13}**C NMR** spectrum of compound **57** (101 MHz, r.t., $CDCl_3$)

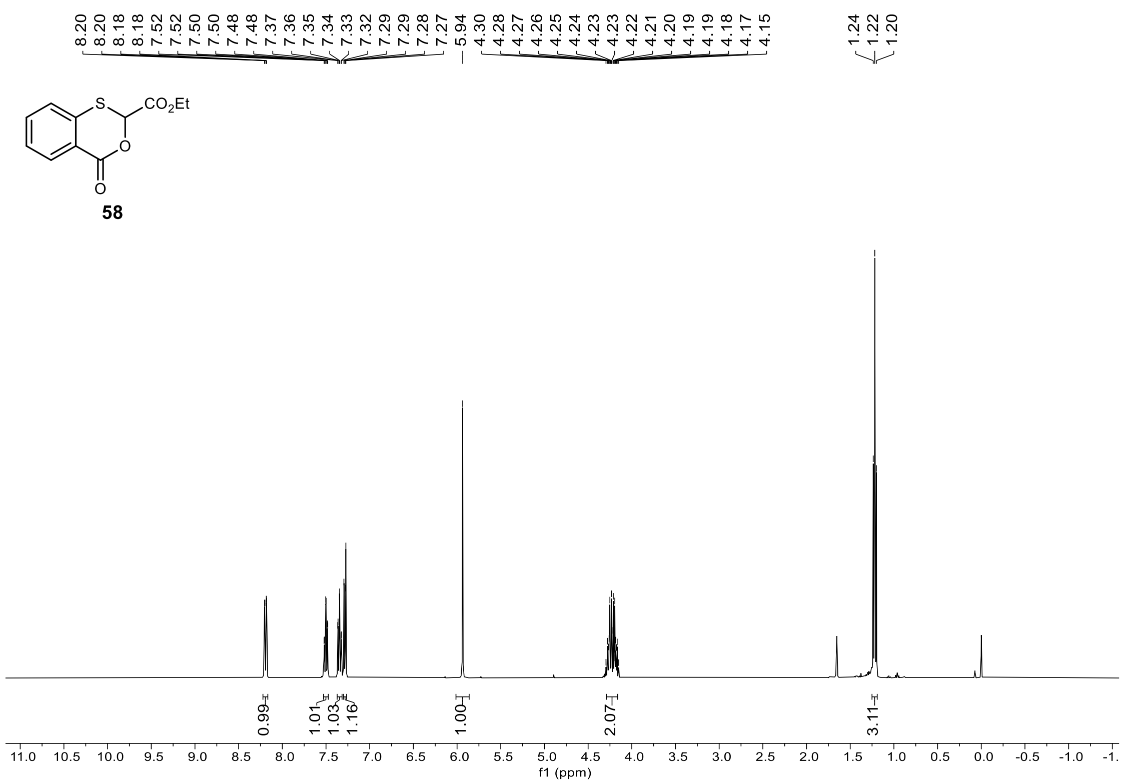

Supplementary Figure 159. ^{1}**H NMR** spectrum of compound **58** (400 MHz, r.t., $CDCl_3$)

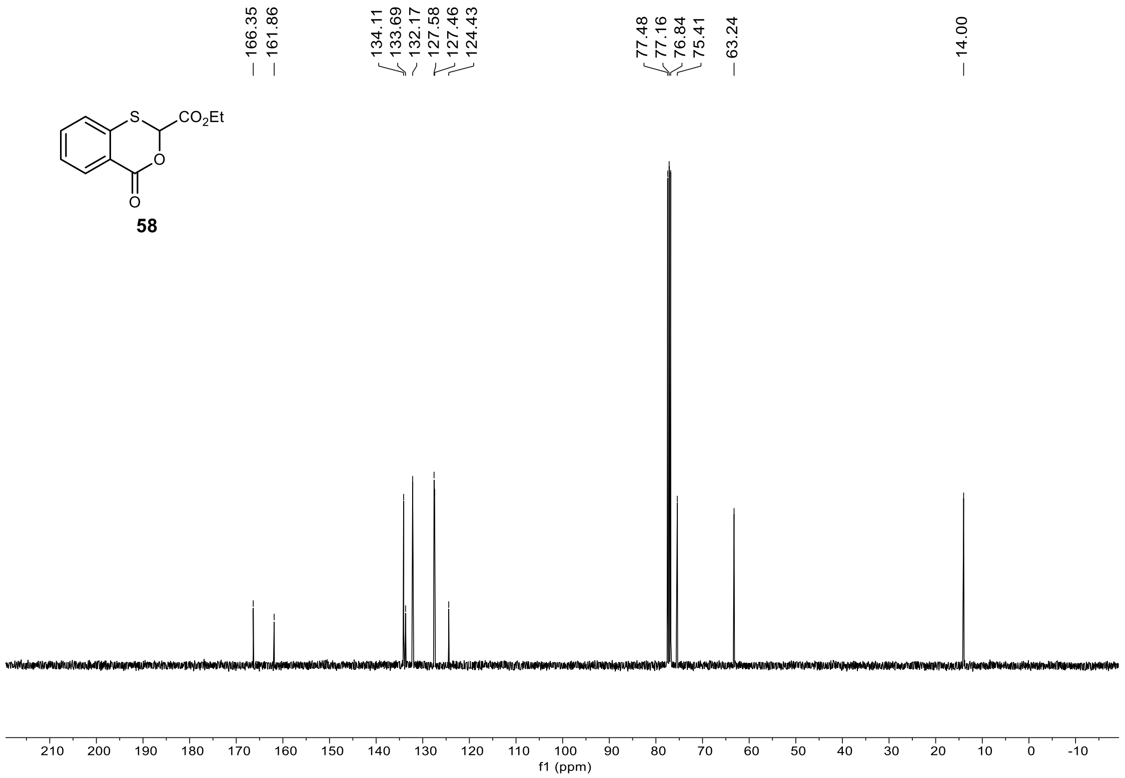

Supplementary Figure 160. ^{13}**C NMR** spectrum of compound **58** (101 MHz, r.t., $CDCl_3$)

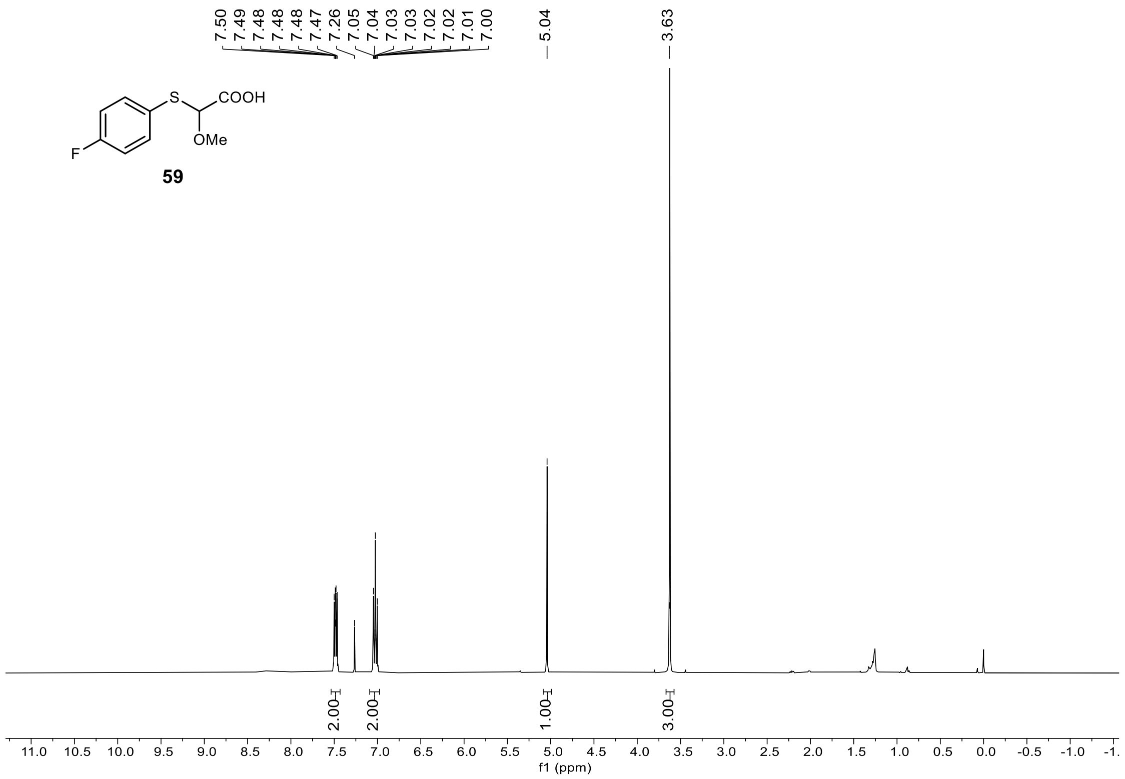

Supplementary Figure 161.[1]**H NMR** spectrum of compound **59** (400 MHz, r.t., $CDCl_3$)

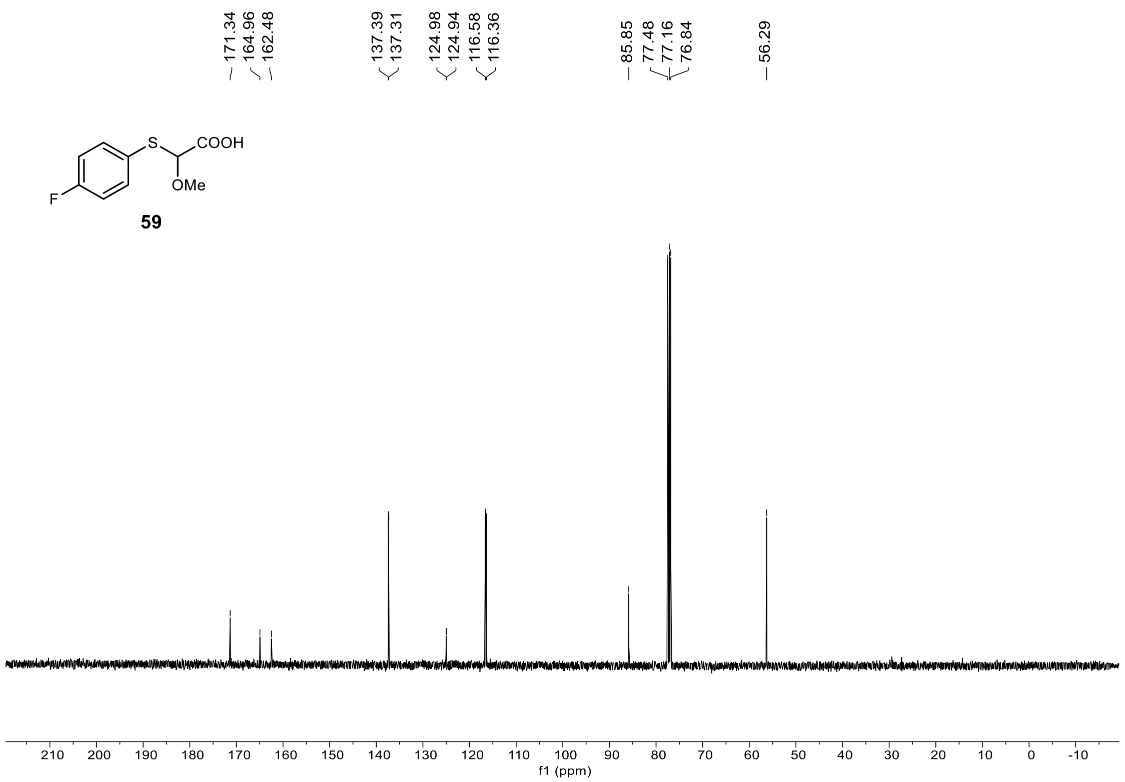

Supplementary Figure 162. [13]**C NMR** spectrum of compound **59** (101 MHz, r.t., $CDCl_3$)

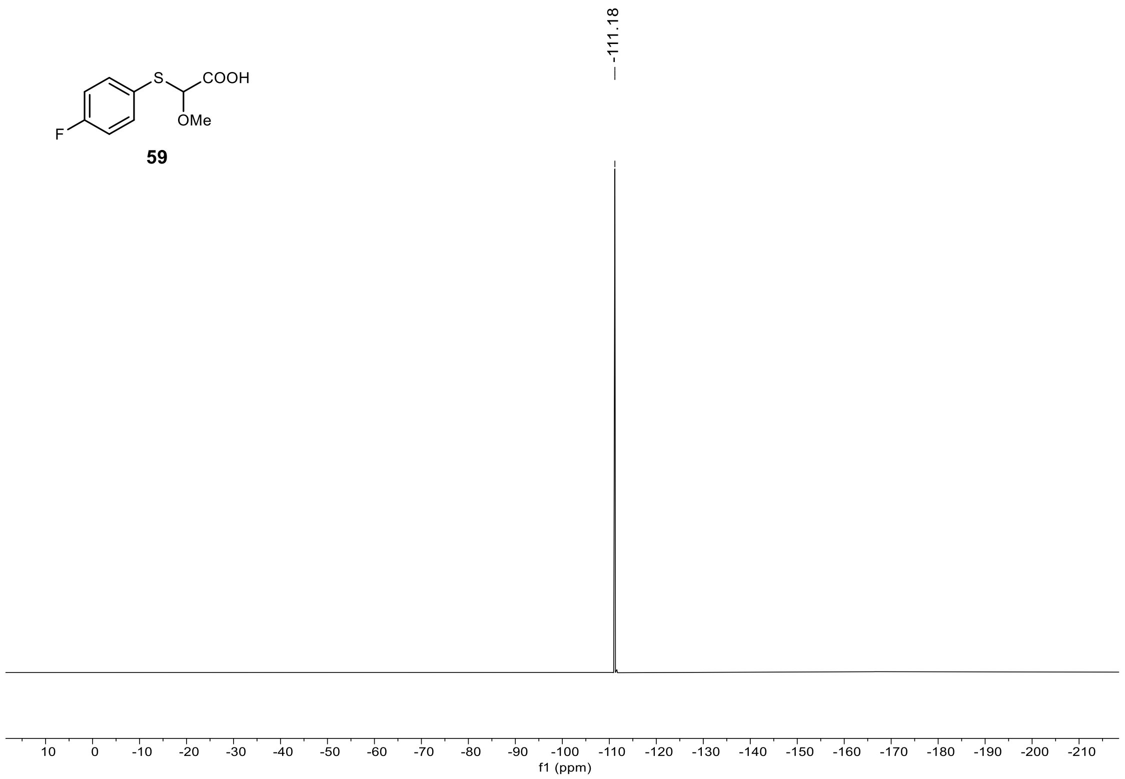

Supplementary Figure 163. ^{19}**F NMR** spectrum of compound **59** (377 MHz, r.t., $CDCl_3$)

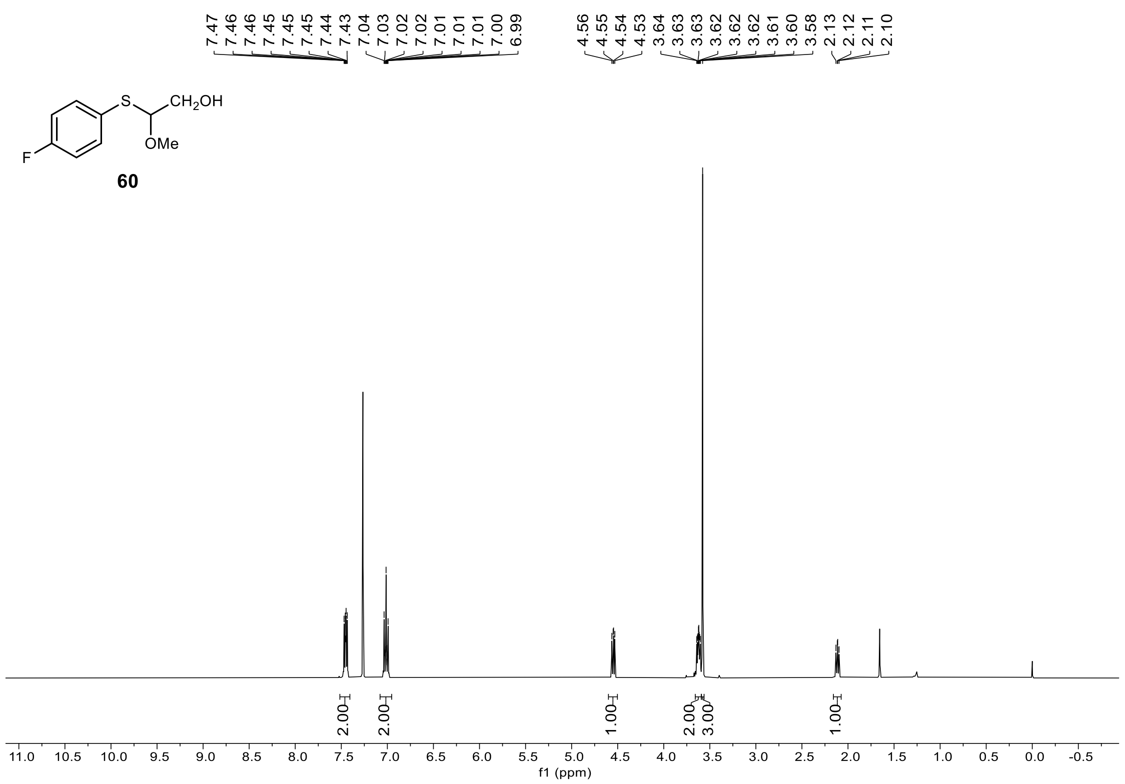

Supplementary Figure 164. ^{1}**H NMR** spectrum of compound **60** (400 MHz, r.t., $CDCl_3$)

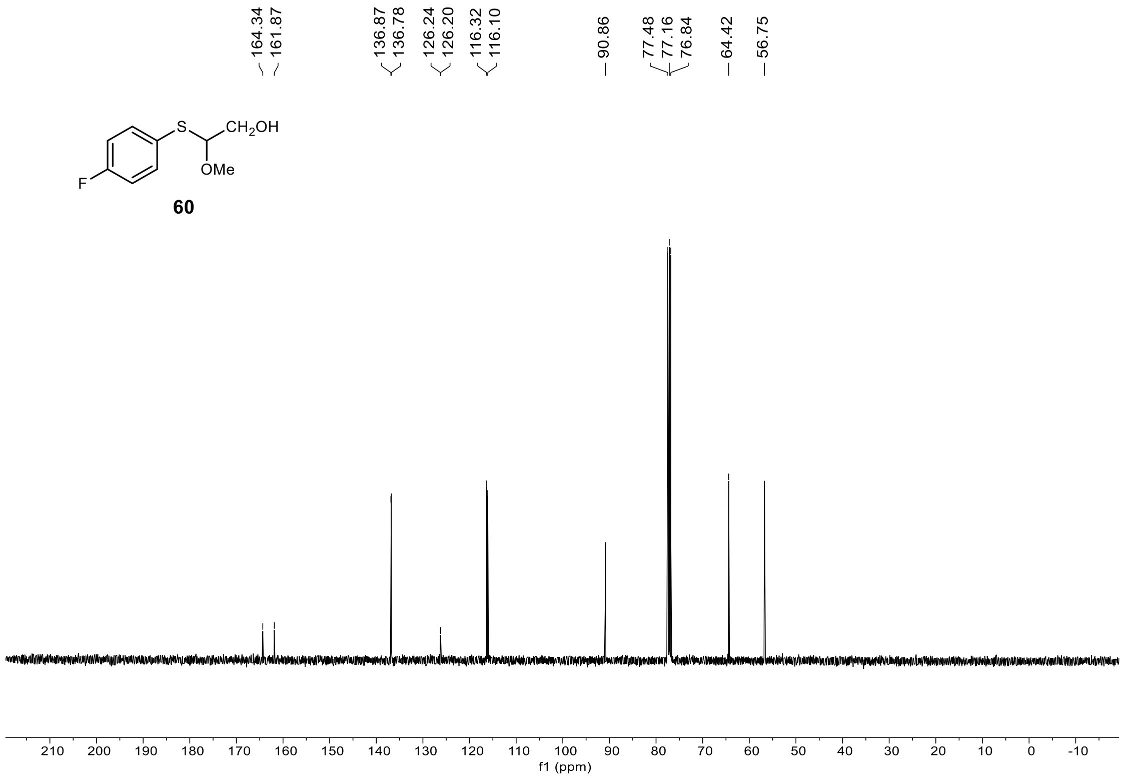

Supplementary Figure 165. ^{13}**C NMR** spectrum of compound **60** (101 MHz, r.t., $CDCl_3$)

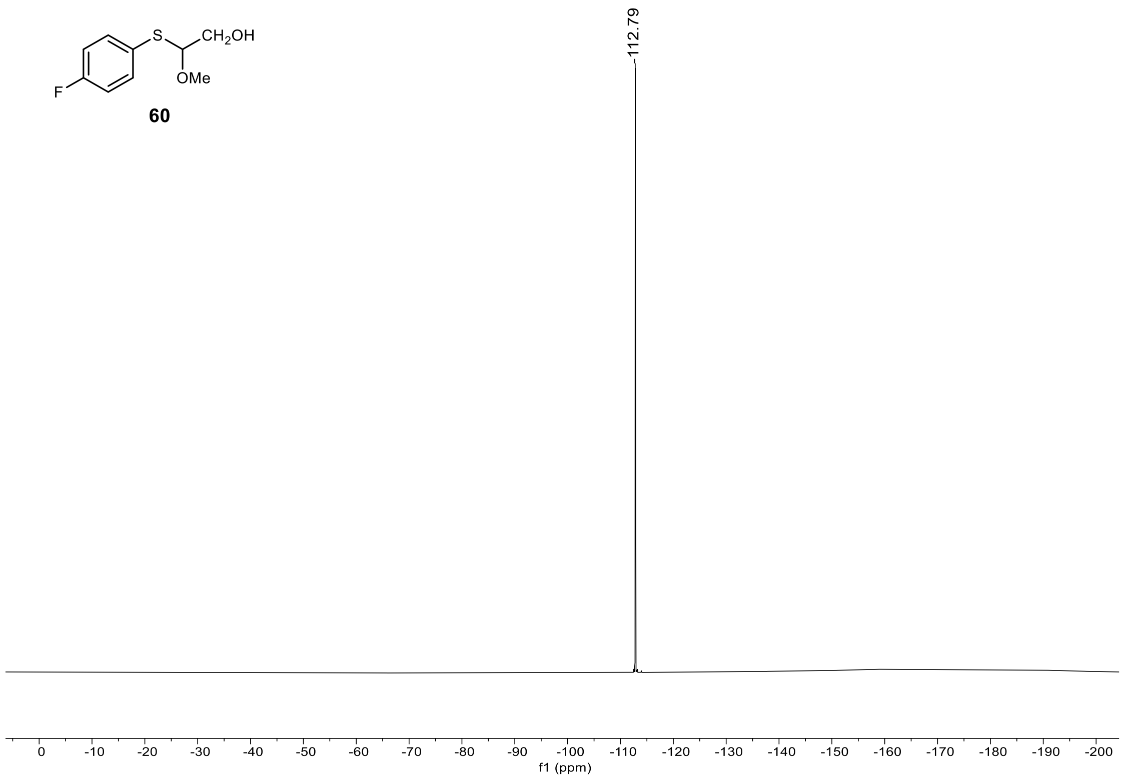

Supplementary Figure 166. ^{19}**F NMR** spectrum of compound **60** (377 MHz, r.t., $CDCl_3$)

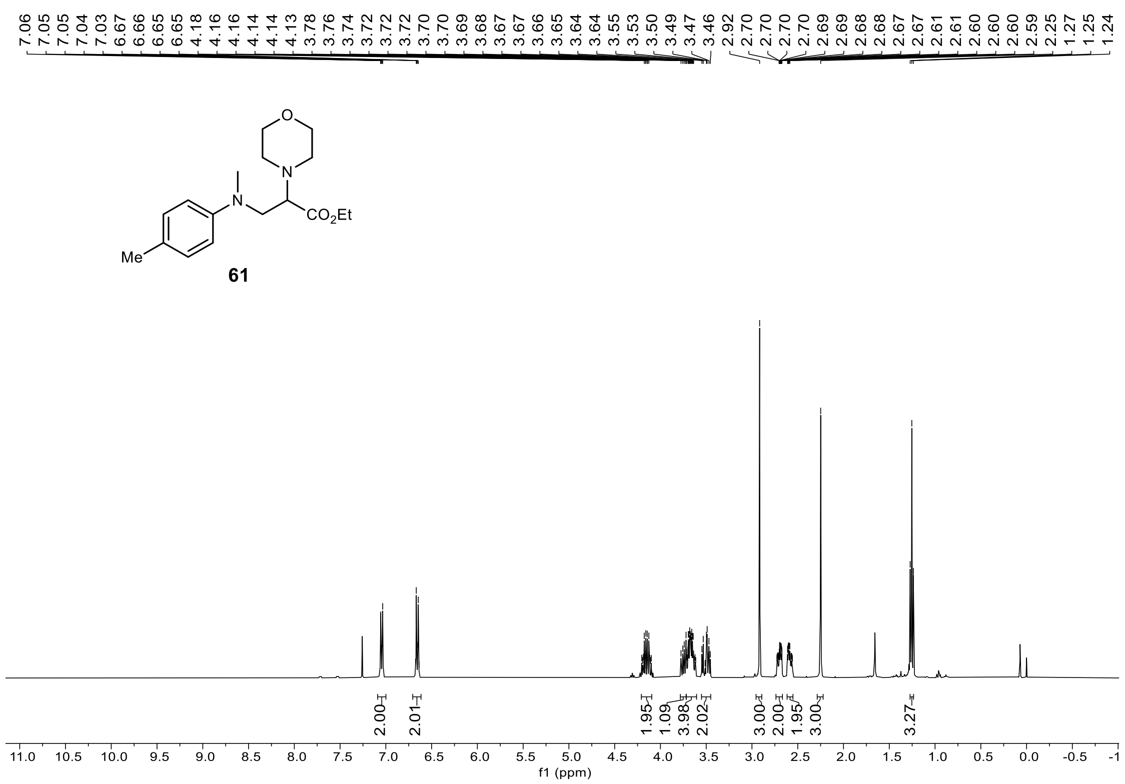

Supplementary Figure 167. **^{1}H NMR** spectrum of compound **61** (400 MHz, r.t., $CDCl_3$)

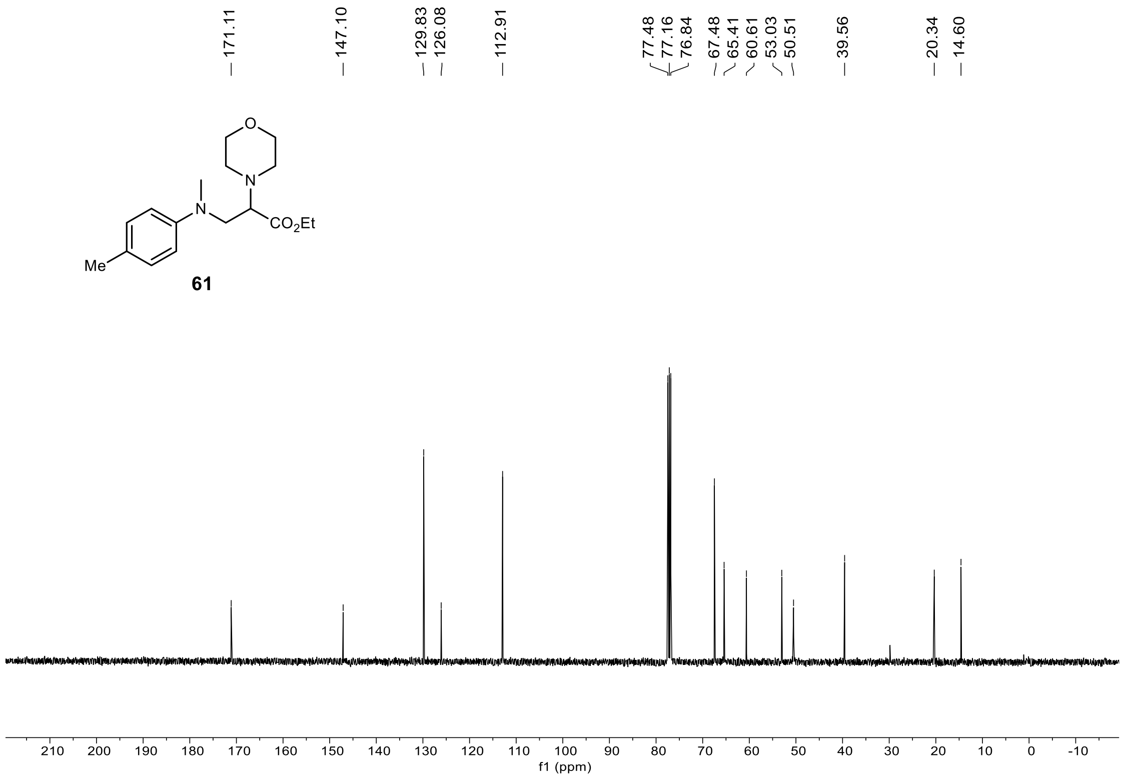

Supplementary Figure 168. **^{13}C NMR** spectrum of compound **61** (101 MHz, r.t., $CDCl_3$)

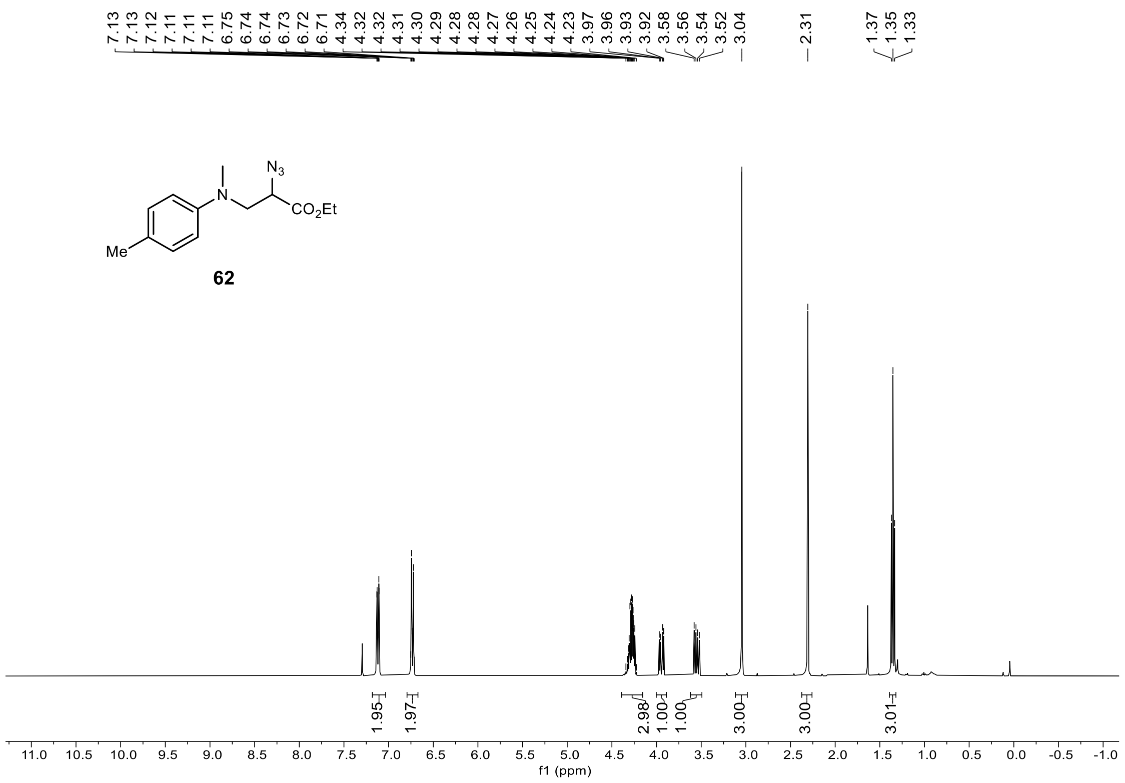

Supplementary Figure 169. **^{1}H NMR** spectrum of compound **62** (400 MHz, r.t., $CDCl_3$)

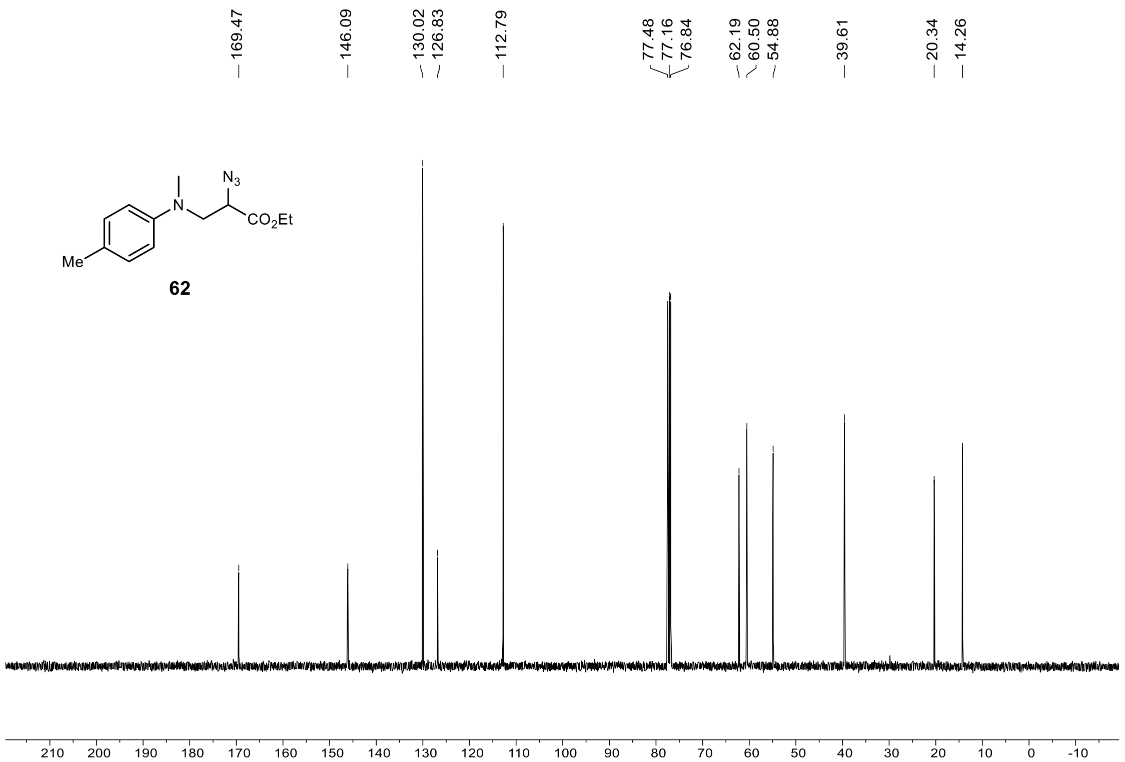

Supplementary Figure 170. **^{13}C NMR** spectrum of compound **62** (101 MHz, r.t., $CDCl_3$)

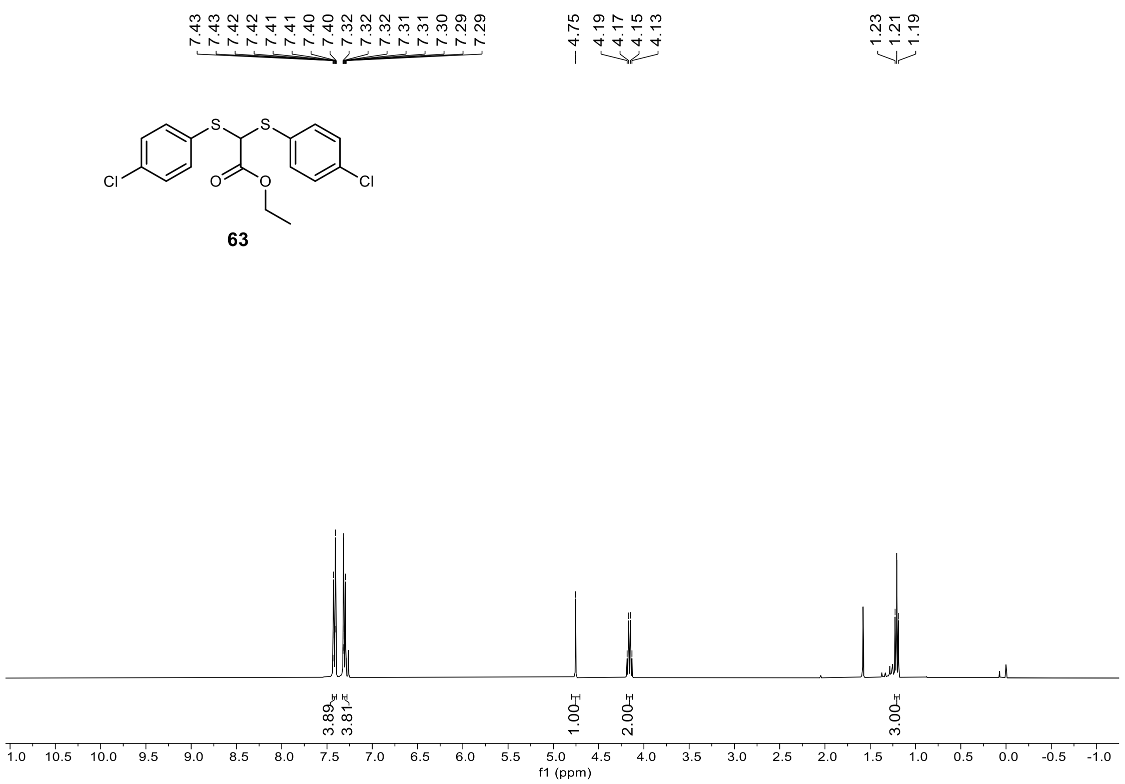

Supplementary Figure 171. ^{1}**H NMR** spectrum of compound **63** (400 MHz, r.t., $CDCl_3$)

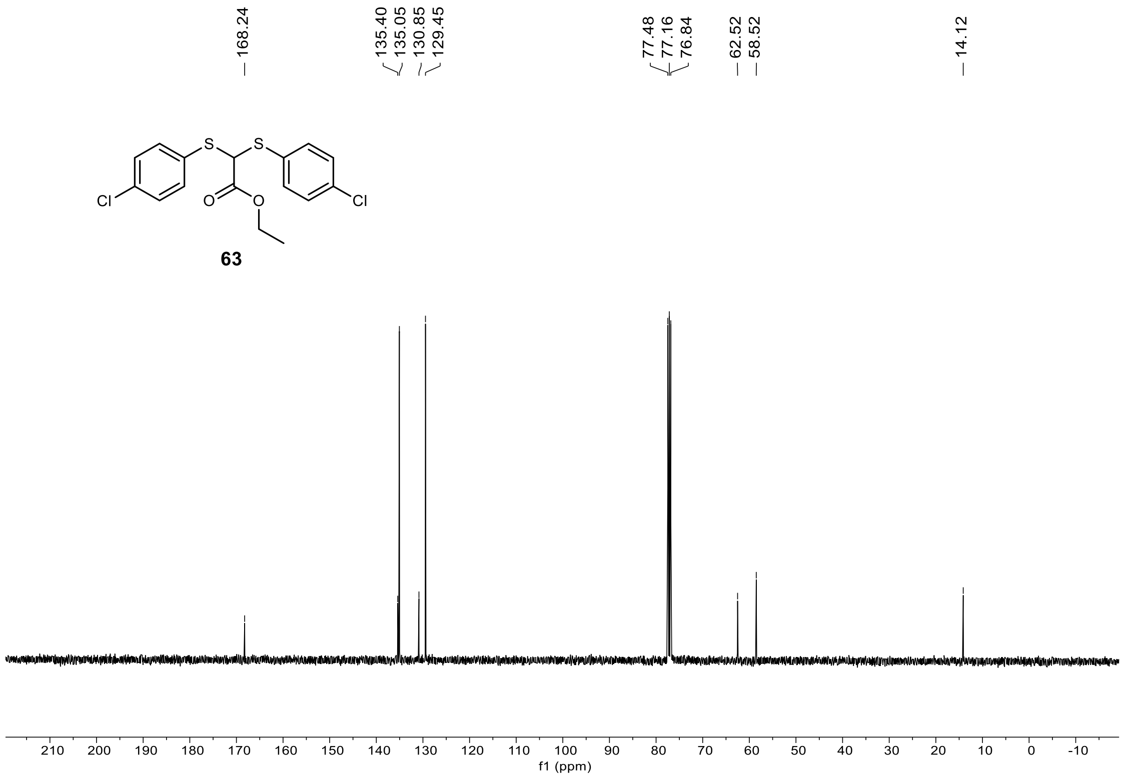

Supplementary Figure 172. ^{13}**C NMR** spectrum of compound **63** (101 MHz, r.t., $CDCl_3$)
